# Inhibition of Dickkopf-1 enhances the anti-tumor efficacy of sorafenib via inhibition of the PI3K/Akt and Wnt/β-catenin pathways in hepatocellular carcinoma

**DOI:** 10.1186/s12964-023-01355-2

**Published:** 2023-11-27

**Authors:** Sang Hyun Seo, Kyung Joo Cho, Hye Jung Park, Hye Won Lee, Beom Kyung Kim, Jun Yong Park, Do Young Kim, Sang Hoon Ahn, Jae Hee Cheon, Jong In Yook, Man-Deuk Kim, Dong Jin Joo, Seung Up Kim

**Affiliations:** 1 Department of Internal Medicine, Graduate School of Medical Science, Brain Korea 21 Project, Yonsei University College of Medicine, Seoul, Korea; 2 Yonsei Liver Center, Severance Hospital, Seoul, Korea; 3 Department of Internal Medicine, Institute of Gastroenterology, Yonsei University College of Medicine, 50-1 Yonsei-ro, Seodaemun–gu, Seoul, 120–752 Korea; 4 Severance Biomedical Science Institute, Yonsei University College of Medicine, Seoul, Korea; 5 Department of Oral Pathology, Yonsei University College of Dentistry, Seoul, Korea; 6 Department of Radiology, Severance Hospital, Yonsei University College of Medicine, Seoul, Korea; 7 Department of Surgery, Yonsei University of College of Medicine, Seoul, Korea

**Keywords:** Dickkopf-1, Sorafenib, Hepatocellular carcinoma, Phosphatidylinositol-3-kinase/protein kinase B pathway, Wnt/β-catenin pathway, GSK3β

## Abstract

**Background:**

Sorafenib improves the overall survival in patients with advanced hepatocellular carcinoma (HCC). Dickkopf-1 (DKK1) is commonly overexpressed in HCC. In this study, we investigated whether the inhibition of DKK1 enhances the anti-tumor efficacy of sorafenib in HCC.

**Methods:**

HCC cells were treated with sorafenib and WAY-262611, which is an inhibitor of DKK1. Transgenic mouse models were also developed using hydrodynamic tail vein injection. Mice were orally administered with sorafenib (32 mg/kg), WAY-262611 (16 mg/kg), or sorafenib + WAY-262611 for 10 days. Mechanisms of sorafenib and WAY-262611 were explored via western blotting, immunostaining, and RNA sequencing.

**Results:**

DKK1 was significantly overexpressed in patients with HCC than in the healthy controls and patients with liver diseases except HCC (all *P* < 0.05). Compared with sorafenib alone, sorafenib + WAY-262611 significantly inhibited the cell viability, invasion, migration, and colony formation by promoting apoptosis and altering the cell cycles in HCC cells (all *P* < 0.05). Moreover, sorafenib + WAY-262611 decreased the p110α, phospho-Akt (all *P* < 0.05), active β-catenin (all *P* < 0.05) and phospho-GSK-3β (Ser9) expression levels, while increasing the phospho-GSK-3β (Tyr216) expression levels compared with those in the sorafenib alone in vitro and in vivo. In addition, sorafenib + WAY-262611 inhibited tumor progression by regulating cell proliferation and apoptosis, significantly better than sorafenib alone in mouse models.

**Conclusions:**

Our results indicate that DKK1 inhibition significantly enhances the anti-tumor efficacy of sorafenib by inhibiting the PI3K/Akt and Wnt/β-catenin pathways via regulation of GSK3β activity, suggesting a novel therapeutic strategy for HCC.

Video Abstract

**Supplementary Information:**

The online version contains supplementary material available at 10.1186/s12964-023-01355-2.

## Background

Hepatocellular carcinoma (HCC) is the most common type of primary liver malignancy and is the fourth leading cause of cancer deaths worldwide [1, 2]. Chronic infection with hepatitis B virus or hepatitis C virus, diabetes, obesity, and toxicity (aflatoxins and alcohol) are the main risk factors for HCC [3–8]. The Food and Drug Administration has approved sorafenib (SOR), lenvatinib (LEN), atezolizumab + bevacizumab, and durvalumab + tremelimumab as first-line standards of systemic therapy for patients with unresectable HCC [9–11].

SOR was the first tyrosine kinase inhibitor (TKI) approved for the treatment of advanced HCC [12, 13]. SOR decreases tumor cell proliferation and angiogenesis by inhibiting vascular endothelial growth factor receptor (VEGFR), fibroblast growth factor receptor, platelet-derived growth factor receptor, and Ras/Raf/MEK signaling cascade [14]. Meta-analyses and multicenter studies have reported that SOR increases the patient survival and delays the progression of HCC [15]. However, the clinical application of SOR is limited by its insensitivity, resistance, and side effects [16].

Dickkopf-1 (DKK1), a secretory inhibitor of the Wnt/β-catenin pathway, plays a crucial role in the induction of head formation during vertebrate development [17–19]. DKK1 is rarely detected in normal human adult tissues, except the placental and embryonic tissues, but it is commonly upregulated in pancreatic cancer, breast cancer, multiple myeloma, and HCC [20–22]. Upregulation of DKK1 expression is observed with vascular or lymphatic invasion and correlates with poor prognosis in patients with HCC.

Mutations are commonly observed in the Wnt/β-catenin pathway components, which result in constitutively activated β-catenin in HCC [23]. Although DKK1 is a well-characterized inhibitor of the Wnt/β-catenin pathway, some studies have reported that DKK1 and β-catenin are positively correlated, suggesting that the Wnt/β-catenin pathway is activated by DKK1 in HCC cells [24, 25]. Besides canonical Wnt/β-catenin pathway, abnormal regulation of DKK1 contributes to other pathways, including the DKK1/cytoskeleton associated protein 4/phosphoinositide 3-kinase (PI3K), β-catenin/matrix metallopeptidase 7, and VEGFR2-mediated PI3K/protein kinase B (Akt) pathways [26–29].

Identification of synergistic agents for SOR is important to increase its sensitivity and therapeutic efficacy, however the association between SOR and DKK1 remain ambiguous [30–32]. Therefore, in this study, we investigated whether the inhibition of DKK1 using WAY-262611 (WAY), a small molecule DKK1 inhibitor, enhances the anti-tumor efficacy of SOR in HCC.

## Methods

### Data analysis using The Cancer Genome Atlas (TCGA) datasets

DKK1 mRNA expression was analyzed using publicly available databases TCGA projects-HCC datasets. In total, 373 HCC and 50 non-tumor samples were obtained from TCGA website [33].

### Patients and specimens

All human samples were obtained with the approval of the Institutional Review Board (IRB) of Severance Hospital (Seoul, Korea). This study adhered to the ethical guidelines of the Declaration of Helsinki 1964. Human serum samples were obtained from the Yonsei Liver Blood Bank of the Division of Gastroenterology and Hepatology, Department of Internal Medicine (IRB numbers 4-2018-0537 and 4-2018-1036). Liver tissues were obtained during liver transplantation from the Department of Transplant Surgery, Yonsei University College of Medicine, Severance Hospital, Seoul, Korea (IRB number 4-2016-0323).

### Enzyme-linked immunosorbent assay (ELISA)

Serum DKK1 concentrations were measured using ELISA kits (R&D Systems, Minneapolis, MN, USA), according to the manufacturer’s instructions.

### Immunohistochemistry (IHC) and immunofluorescence (IF) staining

IHC and IF staining were performed as previously described [28]. Detailed information on the primary and secondary antibodies used in this study is provided in Supplementary Table 1. Protein expression after IHC staining was analyzed under a light microscope (Olympus, Tokyo, Japan) at 200 × and 400 × magnifications. Protein expression after IF staining was observed using Zeiss LSM 700 and 780 confocal microscopes (Carl Zeiss, Oberkochen, Germany) at 200 × and 400 × magnification.

### Mice

All experiments involving animals were performed in accordance with the Guidelines and Regulations for the Care and Use of Laboratory Animals at AAALAC-accredited facilities. This study was approved by the Animal Policy and Welfare Committee of the Yonsei University College of Medicine (Permit number: 2020-0290 and 2020-0299). Four-week-old C57BL/6 and Balb/c nude male mice were purchased from Orient Bio, Inc. (Seongnam, Korea) and Central Lab. Animal, Inc. (Seoul, Korea), respectively. The mice were randomly treated with vehicle (75% ethanol/cremophor EL: DMSO: polyethylene glycol: saline, 1:1:1.5:6.5), SOR (32 mg/kg) (LC Laboratories, Woburn, MA, USA), WAY (16 mg/kg) (Axon Medchem, Groningen, The Netherlands), and SOR + WAY for 10 d. SOR was dissolved in a stock solution containing 75% ethanol and Cremophor EL (Sigma-Aldrich, St Louis, MO, USA) (1:1). Subsequently, the stock solution was dissolved in a saline solution (1:9). WAY was dissolved in DMSO (0.02 g/mL) as a stock solution, which was dissolved in polyethylene glycol (Sigma-Aldrich) and saline water.

### Recombinant DNA and hydrodynamic tail-vein injection

The vector pT2/Hras^G12V^, pT2/short-hairpin RNA down-regulation p53 (shp53), pT2/c-Myc, pT3/EF5a TAZ^S89A^, pT2/EGFP, pT2/Smad7, pT3-EF1a Hras^G12V^-microRNA down-regulating p53 (miRp53), and pT2/PI3K^E545K^ as previously described [34–36].

DNA mixtures (pT2- or pT3 vectors) and transposase-encoding plasmids (SB Transposase) were obtained using the Endo-free Maxi Kit (Qiagen, Hilden, Germany). The DNA mixtures were suspended in a saline solution and intravenously injected into 5-week-old C57BL/6 male mice less than 7 s [37].

### Xenograft mouse model

Hep3B cells were injected subcutaneously into the right flank of Balb/c nude mice (5 × 10^6^ cells/mouse). When the average tumor size reached at 100 mm^3^, the mice were randomly assigned to four groups: Control, SOR (32 mg/kg), WAY (16 mg/kg), and SOR + WAY groups. The doses of sorafenib and WAY were determined according to previous studies [38–41]. We used sorafenib 32 mg/kg and WAY 16 mg/kg as the final working solutions to minimize problems, such as drug dissolution and solvent amount, during the process of preparing the working solution from the concentrated stock solution. SOR and WAY were orally administered for 10 d. Tumor volumes were monitored twice a week using calipers (Tumor volume = length × width^2^/2).

### Cell lines

HCC cell lines (Huh7 and Hep3B) were maintained, and stable cell lines were constructed using the clustered regularly interspaced short palindromic repeats-associated nuclease 9-based DKK1 knockout system in Hep3B cells (Hep3B DKK1 KO), as previously described [28].

### Cell viability assay

Cell viability assays were performed using EZ-Cytox (DoGen, DAEILLAB, Seoul, Korea). Cells were seeded at a density of 3 × 10^3^ cells/150 μL per well in a 96-well plates. After 24 h, cells were treated with varying concentrations of SOR (0.2 nM to 20 μM) (Selleckchem, Houston, TX, USA), LEN (2 nM to 200 μM) (Selleckchem) or WAY (0.2 nM to 20 μM) (Calbiochem, La Jolla, CA, USA) for 24 h or 48 h. After the required incubation, 15 μL of WST-1 reagent was added to each well for 1 h 30 m. Subsequently, cell viability was confirmed by measuring the absorbance at 450 nm using a microplate reader (Molecular Devices, CA, USA). Half-maximal inhibitory concentration (IC_50_) values were calculated using the GraphPad Prism software (GraphPad Software, Inc., CA, USA).

### Invasion, migration, and long-term colony formation assays

Invasion assays were performed using a 24-well transwell permeable plates (Corning Costar, Cambridge, MA, USA). Cells (1 × 10^5^ cells/well) were seeded in the upper chambers of 24-well plates in a serum-starvation medium, and the lower chambers were filled with growth medium containing SOR and/or WAY at IC_50_ values. After 24 h, the cells in the lower chamber were fixed with 60% methanol and stained with hematoxylin and eosin (Dako, Denmark). The number of invading cells was measured using the ImageJ software.

Migration assay was performed using culture-inserts (Ibidi GmbH, Munich, Germany). Cells were seeded at a density of 2 × 10^4^ cells/70 μL per each side. After 24 h, the inserts were detached to create a cell-free area, and the cells were treated with IC_50_ values of SOR and/or WAY for 48 h. The migration area was measured using the ImageJ software.

For long-term colony formation assay, Huh7 and Hep3B cells were seeded in 6-well plates (8 × 10^4^ cells/well). After 24 h, Huh7 and Hep3B cells were treated with SOR, LEN, WAY, and LY294002 at the indicated concentrations (the medium was changed every three days). After 10 d, cells were fixed with 4% paraformaldehyde (Tech & Innovation, Gangwon, Korea) for 20 m and stained with 0.1% crystal violet (Sigma-Aldrich, St Louis, MO, USA) for 20 m. Colony formation rates were measured using the ImageJ software.

### Apoptosis and cell cycle assay

Cells were treated with the IC_50_ values of SOR and WAY. After 24 h, apoptosis assay was performed using the FITC/Annexin V/Dead Cell Apoptosis Kit (Invitrogen, Waltham, MA, USA) according to the manufacturer’s protocol. Apoptotic cells were measured via flow cytometry (Becton Biosciences, Franklin Lakes, NJ, USA).

For cell cycle analysis, cells were treated with SOR and WAY. After 24 h, cells were harvested and fixed with 66% ethanol for 2 h. Fixed cells were stained with propidium iodide and RNase (Abcam, Cambridge, UK) at 37 ℃ for 30 m. Subsequently, cell cycle analysis was conducted via flow cytometry (Becton Biosciences).

### Western blotting

Proteins from HCC cells and mouse liver tissues were obtained using the radioimmunoprecipitation assay buffer (Cell Signaling Technology, Danvers, MA, USA) containing phosphatase inhibitor (GeneDEPOT, Katy, TX, USA). Sodium dodecyl sulfate-polyacrylamide gel electrophoresis (8–15%) was used to separate the proteins (20–40 μg). Subsequently, the proteins were transferred onto polyvinylidene fluoride membranes (GE Healthcare, Piscataway, NJ, USA). Membranes were incubated with primary antibodies (Supplementary Table 1) overnight at 4℃. Subsequently, the membranes were incubated with secondary antibodies, and proteins bands were detected using an enhanced chemiluminescence reagent (PerkinElmer, Waltham, MA, USA).

### RNA-sequencing (RNA-seq) analysis

Cells were treated with IC_50_ values of SOR and WAY. After 24 h, total RNA was extracted from the cells using the RNeasy Mini Kit (Qiagen), according to the manufacturer’s protocol. Subsequently, RNA was quantified using RNA-Seq (Macrogen, Seoul, Korea). Gene sets of the PI3K/Akt (accession no. GSE17661, GSE21755, GSE26599, GSE46693, GSE47108, and GSE55050) and Wnt/β-catenin (GO: 0060070) pathways were obtained from the molecular signatures database, and heat-map analysis was performed using these gene sets.

### Quantitative real-time polymerase chain reaction (qRT-PCR)

RNeasy Mini Kit (Qiagen, Hilden, Germany) was used to obtain the total RNA and cDNA was synthesized using SuperScript^Ⓡ^ III reagent (Invitrogen, Waltham, MA, USA), according to manufacturer’s protocol. Subsequently, cDNA was amplified using SYBR Green PCR Master Mix (Applied Biosystems, Waltham, MA, USA), according to the manufacturer’s protocol. All primer sequences used for qRT-PCR amplification are listed in Supplementary Table 2.

### Statistical analyses

Statistical analyses were conducted using the unpaired parametric Student’s t-test or Fisher’s exact test. Experimental results are presented as the mean ± standard deviation. Statistical significance was set at *P* < 0.05.

Combination index (CI) of SOR + WAY was calculated using the following formula: CI $=\frac{{\mathrm{IC}}_{50}\left(\mathrm{A}+\mathrm{B}\right)}{{\mathrm{IC}}_{50}(\mathrm{A})}+\frac{{\mathrm{IC}}_{50}(\mathrm{A}+\mathrm{B})}{{\mathrm{IC}}_{50}(\mathrm{B})}$ [42]. IC_50_ values for (A) and (B) were obtained for each drug. IC_50_ values of (A + B) were obtained from (A) and (B) treatments. CI > 1, antagonistic effect; CI = 1, additive effect; CI < 1, synergistic effect.

## Results

### DKK1 expression is upregulated in human and mouse HCC

**Fig. 1:**
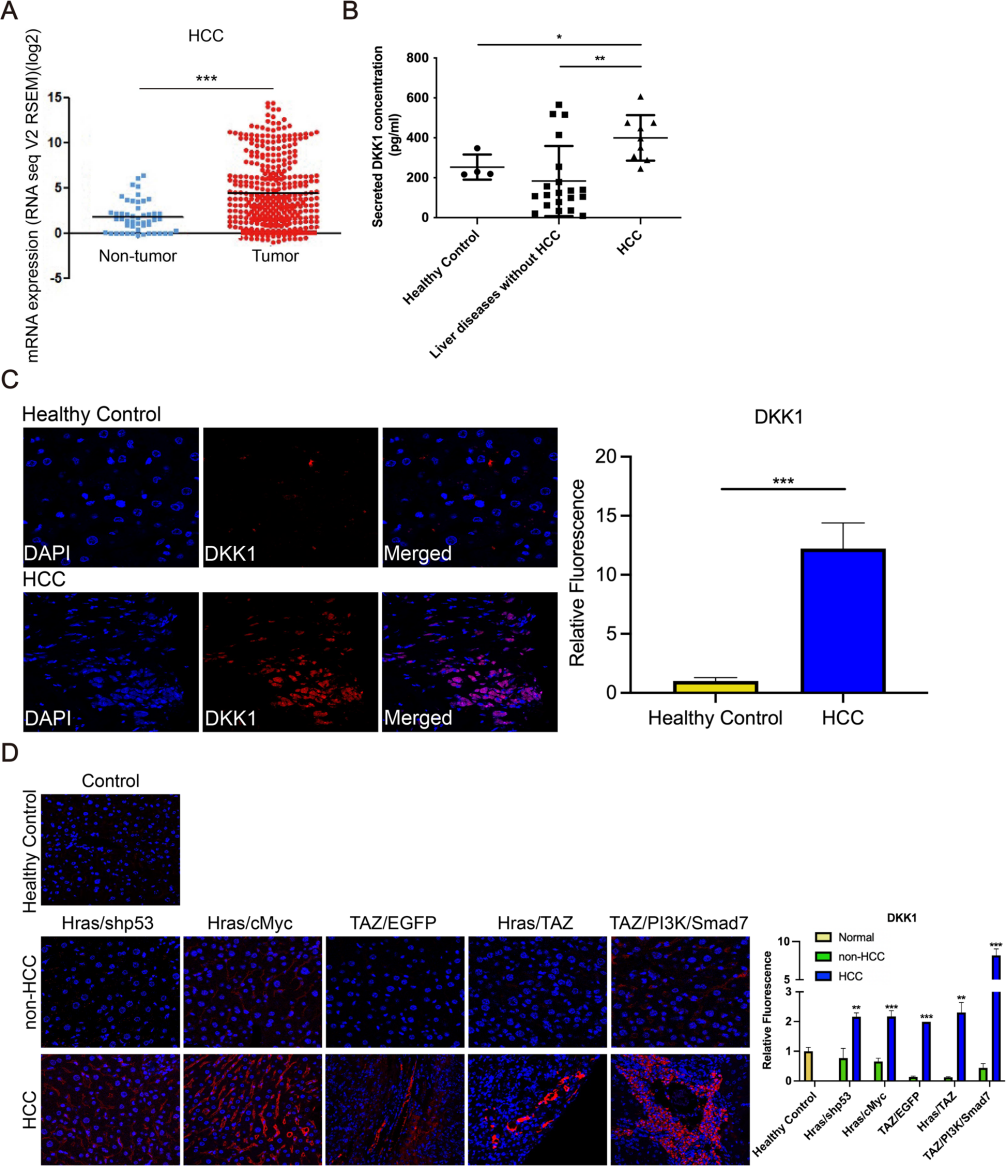
DKK1 is up-regulated in HCC. **A** DKK1 expression levels of non-tumor and tumor tissues were measured using TCGA datasets for HCC. **B** Serum DKK1 levels in healthy controls, patients with liver diseases, but without HCC and patients with HCC. **C** DKK1 expression levels were measured using IF staining in human liver tissues (magnification, 400 ×). **D** Transposon encoding indicated genes were delivered to the mouse liver using hydrodynamic tail vein injection. DKK1 expression levels were measured using IF staining in mouse liver tissues (magnification, 400 ×). Data are presented as the mean ± SD and performed in triplicate independently. Statistical significance is indicated by *P < 0.05, **P < 0.01, ***P < 0.001

TCGA datasets revealed significantly higher mRNA expression levels of DKK1 in patients with HCC (*n* = 373) than in those non-tumor tissues (*n* = 50) (*P* < 0.001) (Fig. 1A). Serum DKK1 levels were significantly higher in patients with HCC (*n* = 9; mean 399.78 ± 113.81 pg/mL) than in the healthy controls (*n* = 4; mean 253.37 ± 63.08 pg/mL) and patients with liver diseases except HCC (mean 183.35 ± 175.92 pg/mL) (hepatitis B virus [*n* = 7], hepatitis C virus [*n* = 3], liver cirrhosis [*n* = 6], and fatty liver [*n* = 4]) (all *P* < 0.05) (Fig. 1B). IF staining revealed that DKK1 levels were significantly higher in patients with HCC than in healthy controls (*P* < 0.001) (Fig. 1C). In the livers of transgenic mice treated with different combinations of oncogenes, DKK1 levels were significantly higher in HCC than in healthy controls and non-tumors (all *P* < 0.01) (Fig. 1D). These findings indicate that DKK1 expression is up-regulated in the blood and tumor tissues of human and mouse HCC.

### SOR + WAY treatment synergistically inhibits the cell viability, invasion, migration, and colony formation in HCC cells in vitro

**Fig. 2:**
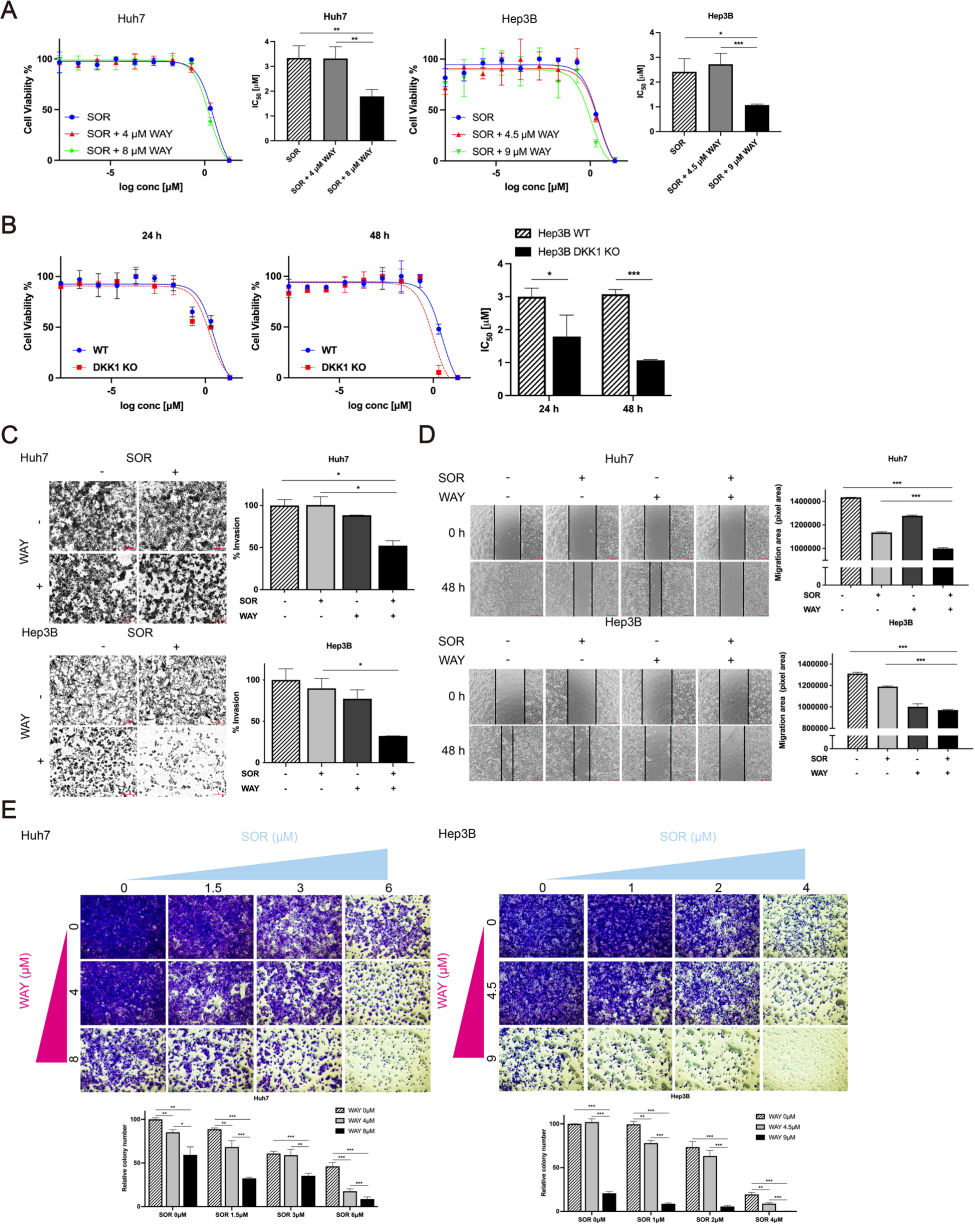
SOR + WAY treatment inhibited the invasion, migration and colony formation abilities of HCC cells. **A** Cell viability assay of diverse concentrations of SOR and SOR combined with WAY to Huh7 and Hep3B cells. **B** Hep3B WT and Hep3B DKK1 KO cells were treated with SOR for 24 h or 48 h. And then cell viability assay of diverse concentrations of SOR to Hep3B WT and Hep3B DKK1 KO cells was evaluated. **C** Invasion, **D** migration, and **E** long-term colony formation assay of Huh7 and Hep3B cells. Data are presented as the mean ± SD and performed in triplicate independently. Statistical significance is indicated by *P < 0.05, **P < 0.01, ***P < 0.001

To investigate the effect of SOR and WAY treatments on the cell viability of HCC cell lines, we performed a cell viability assay. The mean IC_50_ values of WAY were 8.32 ± 0.67 μM and 9.63 ± 0.63 μM in Huh7 and Hep3B cells, respectively (Supplementary Fig. 1A). The mean IC_50_ values of SOR were 3.34 ± 0.49 μM and 2.74 ± 0.34 μM in Huh7 and Hep3B cells, respectively (Fig. 2A). In Huh7 cells, the mean IC_50_ values of SOR + 4 μM WAY and SOR + 8 μM WAY were 3.32 ± 0.47 μM and 1.79 ± 0.28 μM (CI = 0.75, synergistic effect), respectively (Fig. 2A). In Hep3B cells, the IC_50_ values of SOR + 4.5 μM WAY and SOR + 9 μM WAY were 2.72 ± 0.44 μM and 1.07 ± 0.04 μM (CI = 0.56, synergistic effect), respectively (Fig. 2A). In the presence of WAY, the IC_50_ values of SOR were significantly lower than those of SOR treatment alone in Huh7 and Hep3B cells (all *P* < 0.05) (Fig. 2A). LEN was used to further investigate the synergistic effects of the TKI and WAY. In the presence of WAY, the IC_50_ values of LEN were significantly lower than those of LEN treatment alone in Huh7 and Hep3B cells (all *P* < 0.05) (Supplementary Fig. 1B and C) and LEN + WAY treatment inhibited colony formation significantly better than LEN treatment alone in Huh7 and Hep3B cells (all *P* < 0.05) (Supplementary Fig. 1D).

In a previous study, we constructed Hep3B DKK1 KO cells [28]. IC_50_ values of SOR were significantly lower in Hep3B DKK1 KO cells [mean 1.79 ± 0.65 μM (24 h) and 1.07 ± 0.02 μM (48 h)], compared to Hep3B wild type (WT) cells [mean 3.00 ± 0.26 μM (24 h) and 3.08 ± 0.14 μM (48 h)] (all *P* < 0.05) (Fig. 2B). In addition, SOR + WAY treatment inhibited invasion, migration, and colony formation abilities, which were significantly better than those of SOR treatment alone in Huh7 and Hep3B cells (all *P* < 0.05) (Fig. 2C, D and E).

### SOR + WAY treatment synergistically inhibits tumor progression by regulating apoptosis and cell cycle in HCC

**Fig. 3:**
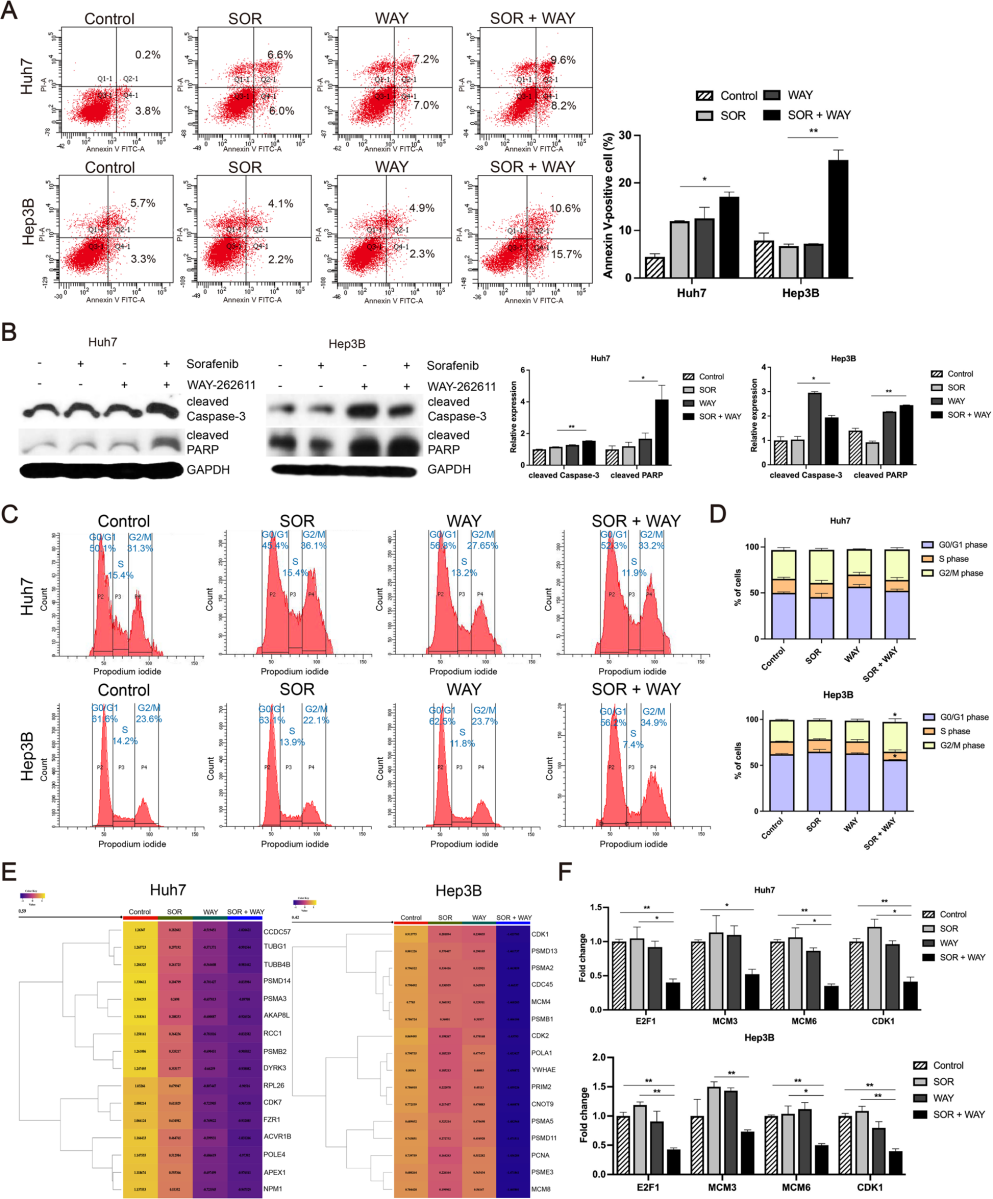
SOR + WAY treatment induced cell apoptosis and cell arrest in HCC cells. Huh7 and Hep3B cells were treated with IC_50_ values of SOR and/or WAY for 24 h. **A** Apoptotic cells were measured using flow cytometric analysis. **B** Apoptosis-related proteins were detected using western blot analysis in Huh7 and Hep3B cells. **C**, **D** Cell cycle analysis of SOR, WAY and their combination in Huh7 and Hep3B cells. **E** Heatmaps of differentially expressed cell cycle associated genes in Huh7 and Hep3 cells. **F** qRT-PCR analysis of cell cycle associated genes in Huh7 and Hep3B cells. Data are presented as the mean ± SD and performed in triplicate independently. Statistical significance is indicated by *P < 0.05, **P < 0.01

SOR + WAY treatment significantly increased the number of apoptotic cells and the expression of apoptotic markers, including cleaved Caspase-3 and cleaved PARP, compared to SOR treatment alone in Huh7 and Hep3B cells (all *P* < 0.05) (Fig. 3A and B). In addition, SOR + WAY treatment increased the percentage of G0/G1 cells, but decreased the percentage of S and G2/M cells better than SOR treatment alone in Huh7 cells, indicating that SOR + WAY treatment induced G0/G1 cell arrest in Huh7 cells (Fig. 3C and D). In Hep3B cells, SOR + WAY treatment increased the percentage of G2/M cells (*P* < 0.05), but decreased the percentage of G0/G1 (*P* < 0.05) and S cells, which was significantly better than SOR treatment alone, indicating that SOR + WAY treatment induced G2/M cell arrest in Hep3B cells (Fig. 3C and D). Heatmaps showed that SOR + WAY treatment decreased the expression of cell cycle associated genes, better effectively than SOR treatment alone in Huh7 and Hep3B cells (Fig. 3E). qRT-PCR showed that SOR + WAY treatment decreased the expression of cell cycle associated genes, including E2F transcription factor 1, microliposome maintenance 6, and cyclin-dependent kinase 1, significantly better than SOR treatment alone in Huh7 and Hep3B cells (all *P* < 0.05) (Fig. 3F). In addition, SOR + WAY treatment significantly decreased tumor weight (*P* < 0.01) and volume (*P* < 0.05), compared to SOR treatment alone in xenograft mice generated using Hep3B cells (Supplementary Fig. 2A, B and C). In SOR + WAY-treated mice, DKK1 levels of tissues and serum (*P* < 0.01) were significantly lower than in SOR-treated mice (Supplementary Fig. 2D and E). In addition, SOR + WAY treatment decreased Ki-67 expression, whereas the increased in cleaved Caspase-3 expression was greater than that of SOR treatment alone (Supplementary Fig. 2F). These findings show that SOR + WAY treatment synergistically inhibited tumor progression by regulating of apoptosis and the cell cycle in HCC.

### SOR + WAY treatment synergistically inhibits the PI3K/Akt and Wnt/β-catenin pathways in vitro

**Fig. 4:**
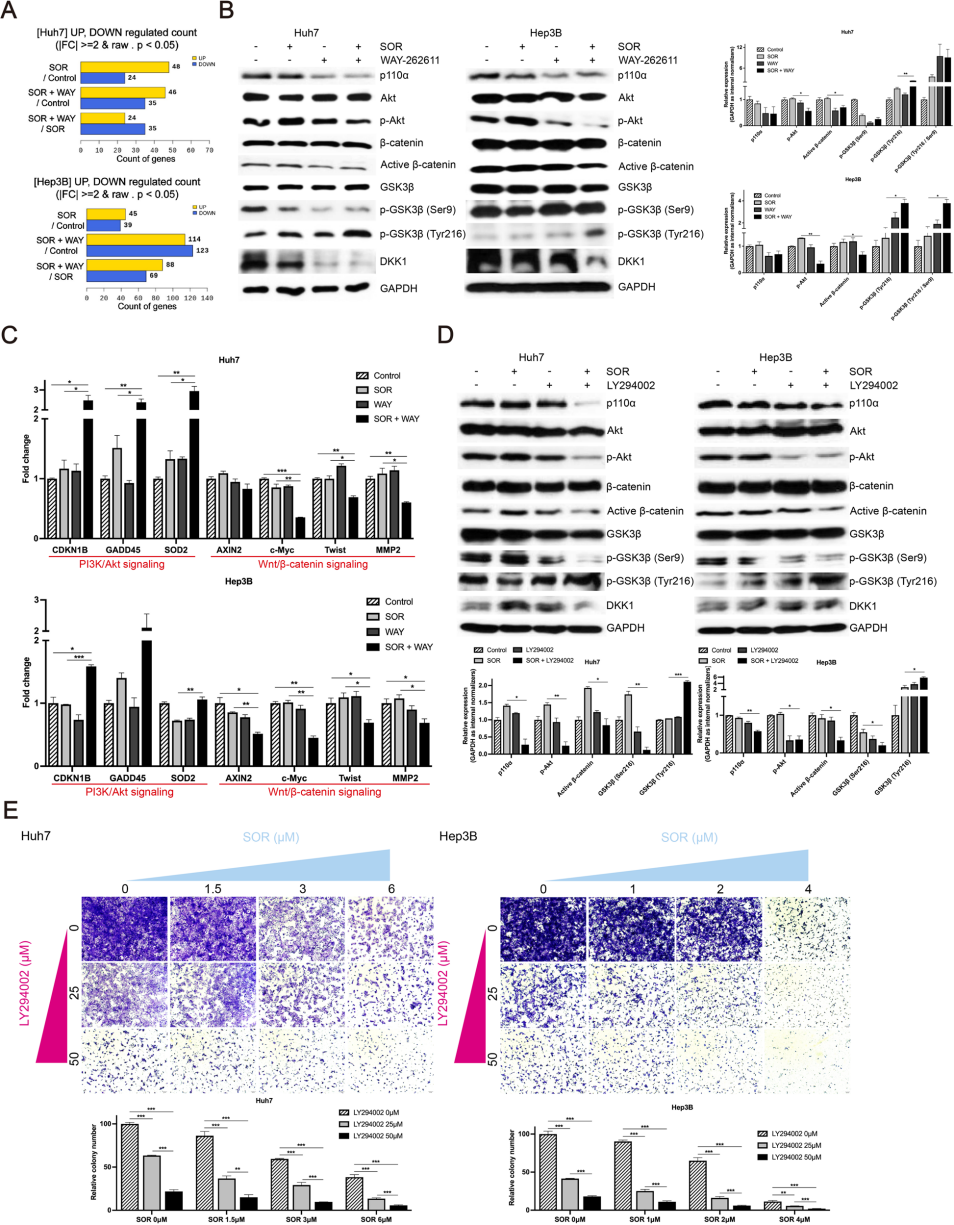
SOR + WAY treatment inhibited the PI3K/Akt and Wnt/β-catenin pathways through GSK3β activation in vitro. Huh7 and Hep3B cells were treated with IC_50_ values of SOR and/or WAY for 24 h. **A** Numbers of differentially expressed genes by comparing SOR to SOR + WAY treatment in Huh7 and Hep3B cells. **B** PI3K/Akt and Wnt/β-catenin pathways associated molecules were detected using western blot analysis in Huh7 and Hep3B cells. **C** qRT-PCR analysis of PI3K/Akt and Wnt/β-catenin pathways associated genes in Huh7 and Hep3B cells. **D** Huh7 and Hep3B cells were treated with IC_50_ values of SOR and/or 25 μM LY294002 for 24 h. PI3K/Akt and Wnt/β-catenin pathways were detected using western blot analysis in Huh7 and Hep3B cells. **E** Huh7 and Hep3B cells were treated with indicated concentrations of SOR and/or LY294002 for 10 d. Subsequently, long-term colony formation assay was confirmed. Data are presented as the mean ± SD and performed in triplicate independently. Statistical significance is indicated by *P < 0.05, **P < 0.01, ***P < 0.001

RNA-seq revealed 59 and 157 differentially expressed genes when comparing the expression of SOR to SOR + WAY treatment in Huh7 and Hep3B cells, respectively (Fig. 4A). SOR + WAY treatment significantly decreased the expression of the PI3K/Akt pathway molecules, such as p110α, p-Akt (all *P* < 0.05), and phosphorylation of GSK3β at Ser 9 (p-GSK3β-Ser9), compared to SOR treatment alone in Huh7 cells (Fig. 4B). In Hep3B cells, SOR + WAY treatment significantly decreased the expression of p110α and p-Akt (*P* < 0.01), compared to SOR treatment alone, however no difference in p-GSK3β-Ser9 levels was observed (Fig. 4B). In addition, SOR + WAY treatment significantly decreased the expression of non-phosphorylated β-catenin (active β-catenin) (all *P* < 0.05), whereas significantly increased the expression of phosphorylation of GSK3β at Tyr 216 (p-GSK3β-Tyr216) (all *P* < 0.05), compared to SOR treatment alone in Huh7 and Hep3B cells (Fig. 4B). These results suggested that SOR + WAY treatment synergistically inhibited the PI3K/Akt and Wnt/β-catenin pathways. Subsequently, SOR + WAY treatment significantly increased the expression levels of the PI3K/Akt inhibitory genes, such as cyclin-dependent kinase inhibitor 1B (all *P* < 0.05), growth arrest and DNA damage-inducible 45, and superoxide dismutase 2 (all *P* < 0.05), whereas it significantly decreased the expression levels of the Wnt/β-catenin pathway target genes, such as c-Myc, Twist, and matrix metalloproteinase 2, compared to SOR treatment alone in Huh7 and Hep3B cells (all *P* < 0.05) (Fig. 4C). Heatmaps showed that SOR + WAY treatment effectively decreased the expression of the PI3K/Akt and Wnt/β-catenin pathway target genes, compared to SOR treatment alone in Huh7 and Hep3B cells (Supplementary Fig. 3A and B).

To further investigate whether SOR + WAY treatment inhibits the PI3K/Akt and Wnt/β-catenin pathways, we used a PI3K inhibitor (LY294002). SOR + LY294002 treatment not only changed the expression of the PI3K/Akt pathway molecules, such as p110α, p-Akt and p-GSK3β-Ser9, but also the expression of the Wnt/β-catenin pathway molecules, such as active β-catenin and p-GSK3β-Tyr216, significantly better than SOR treatment alone in Huh7 and Hep3B cells (all *P* < 0.05) (Fig. 4D). In addition, in the presence of LY294002, SOR significantly inhibited the colony formation abilities of Huh7 and Hep3B cells, compared to SOR treatment alone (all *P* < 0.01) (Fig. 4E).

These findings indicate that SOR + WAY treatment inhibits the PI3K/Akt and Wnt/β-catenin pathways by regulating of GSK3β activity in vitro.

### SOR + WAY treatment synergistically inhibits the PI3K/Akt and Wnt/β-catenin pathways in vivo

**Fig. 5:**
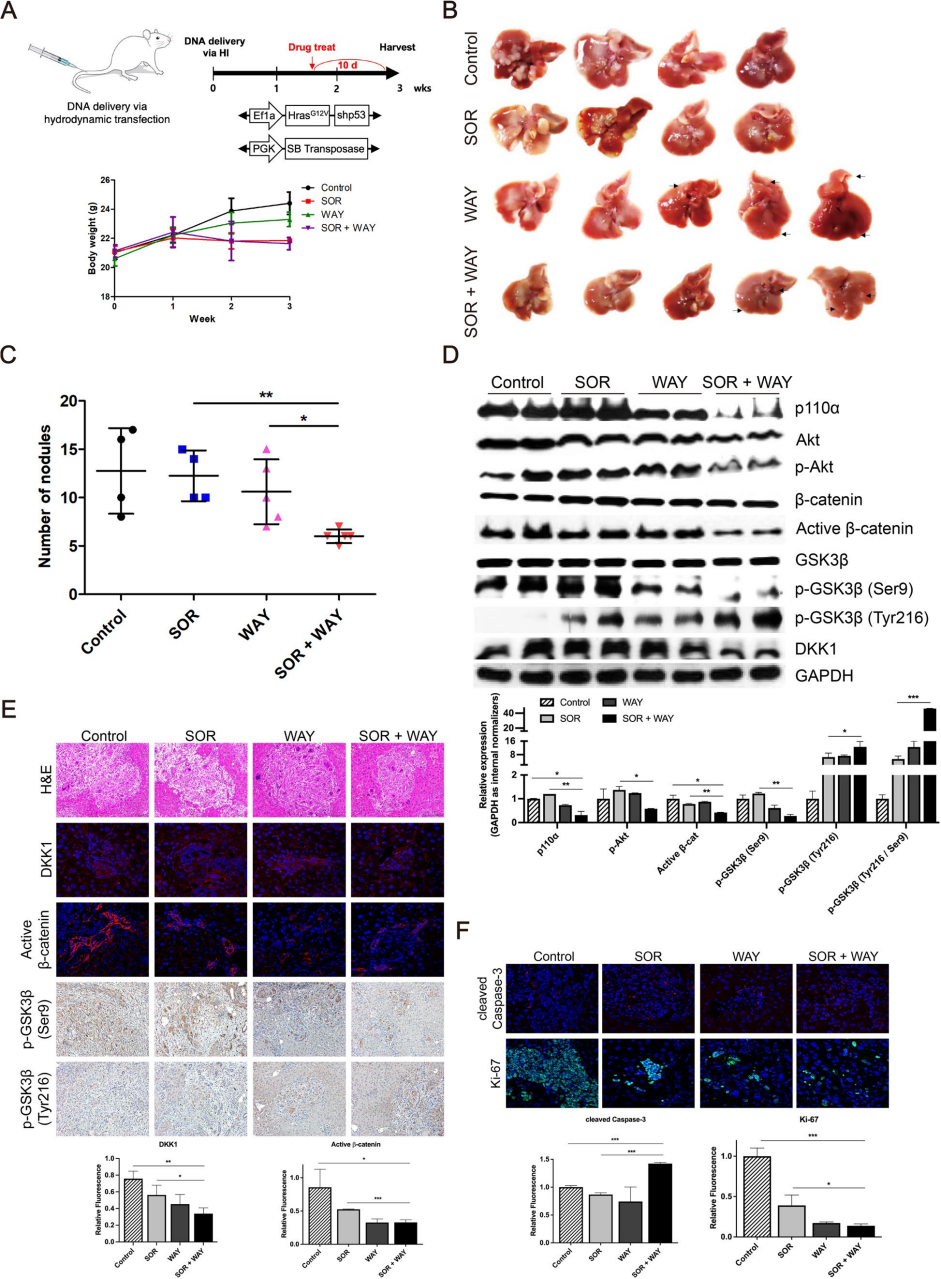
SOR + WAY treatment inhibited the PI3K/Akt and Wnt/β-catenin pathways through GSK3β activation in vivo. **A** Schedule for the experiments on the mice transfected with Hras^G12V^ and shp53. Mice were divided into four groups: Control (*n* = 4), SOR (*n* = 4), WAY (*n* = 5), SOR + WAY (*n* = 5). Mice of each group were orally administered for 10 d before sacrificed. **B** Representative liver pictures of each group. **C** Number of nodules in mouse liver of each group was counted. **D** PI3K/Akt and Wnt/β-catenin pathways associated molecules were detected using western blot analysis in mouse liver tumors. **E** H&E, IF and IHC staining of DKK1, active β-catenin, p-GSK3β-Ser9 and p-GSK3β-Tyr216 in mouse liver. **F** IF staining of cleaved Caspase-3 and Ki-67 in mouse liver. Data are presented as the mean ± SD and performed in triplicate independently. Statistical significance is indicated by *P < 0.05, **P < 0.01, ***P < 0.001

Mice were transfected with Hras^G12V^ and shp53 via hydrodynamic tail vein injection. Subsequently, the mice were orally administered vehicle (*n* = 4), SOR (*n* = 4), WAY (*n* = 5), or SOR + WAY (*n* = 5) for 10 d (Fig. 5A). SOR + WAY treatment inhibited tumor progression, more significantly than SOR treatment alone (*P* < 0.05) (Fig. 5B and C). In mouse liver tumors, SOR + WAY treatment decreased the expression of the PI3K/Akt pathway molecules, such as p110α, p-Akt and p-GSK3β-Ser9, significantly better than SOR treatment alone (all *P* < 0.05) (Fig. 5D and E). In Wnt/β-catenin pathway, SOR + WAY treatment significantly decreased the expression of active β-catenin, whereas significantly increased the expression of p-GSK3β-Tyr216, compared to SOR treatment alone (all *P* < 0.05) (Fig. 5D and E). In addition, SOR + WAY treatment significantly increased cleaved Caspase-3 expression (*P* < 0.001), whereas significantly decreased Ki-67 expression (*P* < 0.05), compared to SOR treatment alone (Fig. 5F).

These findings show that SOR + WAY treatment synergistically inhibits tumor progression by inhibiting the PI3K/Akt and Wnt/β-catenin pathways and altering cell proliferation in vivo.

### Linkage between the PI3K/Akt and Wnt/β-catenin pathways in HCC

**Fig. 6:**
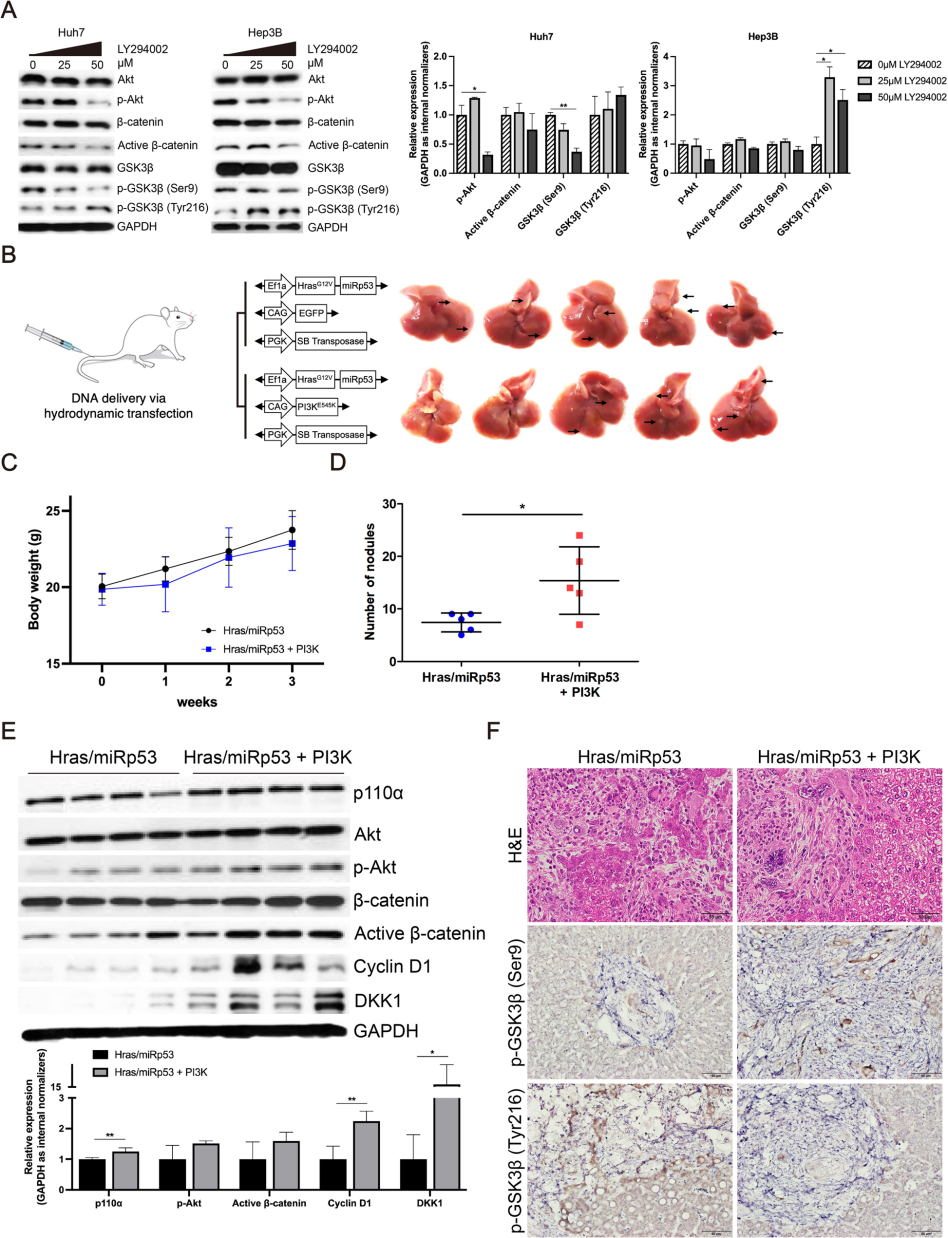
PI3K/Akt and Wnt/β-catenin pathways were connected by GSK3β in HCC. **A** Huh7 and Hep3B cells were treated with the indicated concentrations of LY294002 for 24 h. PI3K/Akt and Wnt/β-catenin pathways associated molecules were detected using western blot analysis. **B** Schematic illustration of the experiments on the mice transfected with Hras^G12V^ and miRp53 (*n* = 5)-injected group and Hras^G12V^, miRp53, and PI3K^E545K^-injected group (*n* = 5). **C** Body weight of each group was confirmed every week for 3 weeks. **D** Number of nodules in mouse liver of each group was counted. **E** PI3K/Akt and Wnt/β-catenin pathways associated molecules were detected using western blot analysis in mouse liver tumors. **F** H&E and IHC staining of p-GSK3β-Ser9 and p-GSK3β-Tyr216 in mouse liver. Data are presented as the mean ± SD and performed in triplicate independently. Statistical significance is indicated by *P < 0.05, **P < 0.01

To identify the correlation between PI3K/Akt and Wnt/β-catenin pathways, Huh7 and Hep3B cells were treated with LY294002, an inhibitor of PI3K. LY294002 significantly decreased the expression of p-Akt (*P* < 0.05), active β-catenin and p-GSK3β-Ser9 (*P* < 0.01), whereas increased p-GSK3β-Tyr216 expression, compared to controls in Huh7 cells (Fig. 6A). In Hep3B cells, LY294002 treatment decreased the expression of p-Akt, active β-catenin and p-GSK3β-Ser9, whereas significantly increased p-GSK3β-Tyr216 expression (*P* < 0.05), compared to controls (*P* < 0.05) (Fig. 6A). These results suggested that inhibition of the PI3K/Akt pathway decreased the expression of active β-catenin via regulation of GSK3β activity in vitro.

To activate the PI3K/Akt pathway, mice were transfected with Hras^G12V^, miRp53 and PI3K^E545K^ via hydrodynamic tail vein injection (Fig. 6B and C). Mice injected with Hras^G12V^, miRp53, and PI3K^E545K^ (Hras/miRp53 + PI3K) (*n* = 5) showed tumor formation significantly more than mice injected with Hras^G12V^ and miRp53 (Hras/miRp53) (*n* = 5) (*P* < 0.05) (Fig. 6D). Simultaneous expression of Hras^G12V^, miRp53, and PI3K^E545K^ significantly increased the expression of p110α (*P* < 0.01), p-Akt, active β-catenin, cyclin D1 (*P* < 0.01), and DKK1 (*P* < 0.05), compared to Hras^G12V^ and miRp53 transfected mouse liver tumors (Fig. 6E). In Hras^G12V^, miRp53, and PI3K^E545K^ transfected mouse liver, p-GSK3β-Ser9 expression increased, whereas p-GSK3β-Tyr216 expression decreased, compared to Hras^G12V^ and miRp53 transfected mouse liver (Fig. 6F). These results show that PI3K/Akt pathway regulates Wnt/β-catenin pathway via regulation of GSK3β activity in vivo.

To further confirm the combined effects of SOR + WAY treatment on PI3K/Akt pathway activation, transgenic mice induced by Hras^G12V^, miRp53, and PI3K^E545K^ were orally administered SOR and/or WAY for 10 d (Supplementary Fig. 4A and B). SOR + WAY treatment inhibited tumor progression more significantly than SOR treatment alone (*P* < 0.05) (Supplementary Fig. 4C and D). In addition, SOR + WAY treatment decreased the expression of p110α, p-Akt and p-GSK3β-Ser9, whereas increased p-GSK3β-Tyr216 expression, compared to SOR treatment alone in Hras^G12V^, miRp53, and PI3K^E545K^ transfected mouse liver tumors (Supplementary Fig. 4E).

**Fig. 7:**
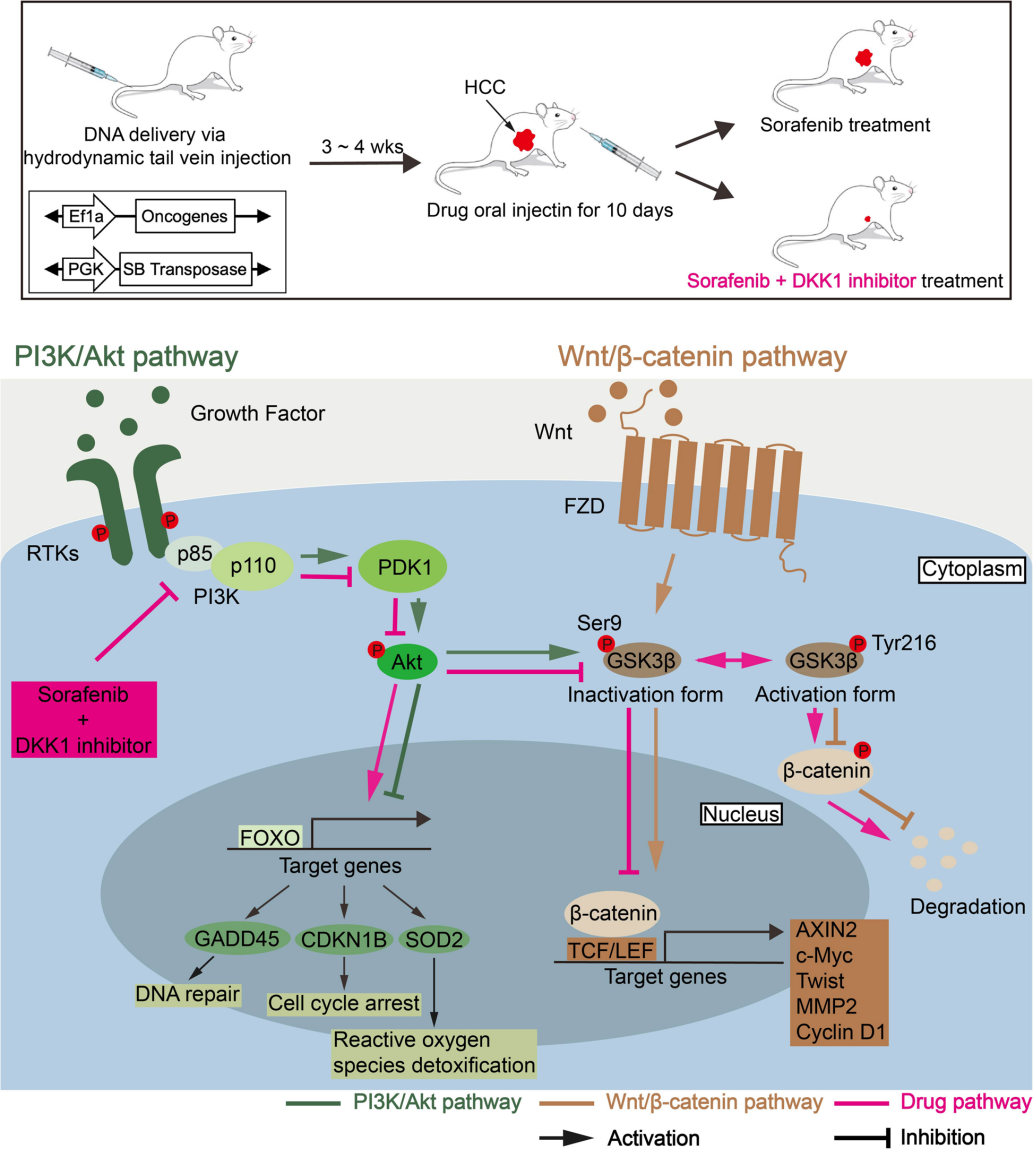
SOR and DKK1 inhibitor treatment inhibited PI3K/Akt and Wnt/β-catenin pathways. Combination treatment of SOR and DKK1 inhibitor decreased the expression of p110α, p-Akt and p-GSK3β-ser9, better than SOR treatment alone in vitro and in vivo. Subsequently, elevated expression of p-GSK3β-Tyr216 induced β-catenin degradation and decreased β-catenin target gene levels

These findings show that the PI3K/Akt pathway is associated with Wnt/β-catenin pathway and that SOR + WAY treatment synergistically inhibited the PI3K/Akt and Wnt/β-catenin pathways via regulation of GSK3β in HCC (Fig. 7).

## Discussion

DKK1 is dysregulated in various cancers, suggesting its potential as a diagnostic biomarker and therapeutic target for multiple cancer types [43–45]. In this study, we found that DKK1 was upregulated in human and mouse HCC. Second, SOR combined with DKK1 inhibitor synergistically inhibited the cell viability, invasion, migration, and colony formation in HCC cells. Third, SOR combined with DKK1 inhibitor also inhibited tumor progression by regulating apoptosis and cell cycle progression. Fourth, SOR combined with DKK1 inhibitor inhibited the PI3K/Akt and Wnt/β-catenin pathways via regulation of GSK3β activity in vitro and in vivo. Fifth, notably, PI3K/Akt and Wnt/β-catenin pathways were correlated through GSK3β in HCC.

Our study has several unique findings. First, DKK1 plays different roles in tumor progression in each tumor type, and the role of its dysregulation in human tumors remains controversial. Although, DKK1 is not detected in normal human tissues, except the placental and embryonic tissues [18, 20], Shen et al. [21] reported that DKK1 levels were significantly higher in patients with HCC than in the healthy controls. Chen et al. [46] showed that elevated DKK1 levels enhanced the migration and invasion of human HCC cells by increasing of β-catenin expression. Fezza et al. [47] showed that DKK1 increased the expression of oncogenes and decreased the expression of tumor suppressor genes by increasing TGF-β1 expression in HCC cells. In addition, we previously reported that DKK1 promoted angiogenesis and tumor progression by regulating the VEGFR2-mediated pathway [28, 29]. In this study, our data showed that DKK1 levels in serum and tissues are significantly higher in human and mouse HCC, which is consistent with the results of previous studies, which is the rationale for our study to investigate the effects of DKK1 inhibitor in HCC.

Second, we found that DKK1 inhibition increased the anti-tumor efficacy of SOR in HCC. Many studies have shown that inhibition of tyrosine kinases decreases the cell proliferation, migration, and invasion abilities of HCC cells [48–50]. However, because TKI-treated patients with HCC have a low response and side effects, the identification of TKI enhancers is important to overcome HCC [51–53]. DKK1 inhibitors, such as small molecules, antibodies, nucleic acids, and proteins or mRNA, have been considered as synergistic agents for combination therapy in diverse tumors [54]. Zhang et al. [55] showed that inhibition of DKK1 by miR203 treated with BPI-9016 M, a novel small-molecule c-MET inhibitor, decreased the migration and invasion abilities of lung adenocarcinoma cells, significantly more than c-MET inhibitor alone. Ryan et al. [56] showed that DKN-01, a humanized IgG4 monoclonal antibody, enhanced the anti-tumor efficacy of paclitaxel in patients with DKK1-expressing esophageal or gastro-esophageal junction tumors. Similarly, our data showed that when DKK1 was inhibited by the small molecule, WAY-262611, the anti-tumor efficacy of SOR on cell viability, invasion, migration, and colony formation was significantly enhanced in HCC cells.

Third, the correlation between DKK1 and the effects of SOR on apoptosis and the cell cycles has not yet been confirmed. Our current study is the first to show that the inhibition of DKK1 regulates the apoptotic activities and cell cycle responses to SOR in HCC cells. It has been well known that dysregulation of apoptosis is closely associated with tumor initiation, progression and metastasis [57]. Liu et al. [58] showed that SOR administration increases the number of apoptotic cells and decreases the expression of phosphorylated eIF4E in HCC xenograft tumors. Sonntag et al. [59] showed that SOR decreased the expression of anti-apoptotic proteins, such as myeloid cell leukemia 1, B-cell lymphoma-2, and B-cell lymphoma-extra-large, whereas it increased the expression of c-Cas-3 in Hepa1-6 hepatoma cells. Zhang et al. [60] showed that SOR induces the intrinsic apoptotic pathway by regulating pro-apoptotic protein levels in acute myeloid leukemia cells. Our current study showed that SOR combined with DKK1 inhibitor increased the number of apoptotic cells and the expression of related proteins significantly better than SOR alone. In addition, because cell division contributes to tumor progression, regulation of the cell cycles is considered a novel therapeutic strategy in cancers. Wei et al. [48] showed that SOR decreased the HCC cell growth by regulating cell cycle regulatory proteins. In the present study, we found that SOR combined with DKK1 inhibitor induced G0/G1 arrest in Huh7 cells and G2/M arrest in Hep3B cells and decreased tumor progression in xenograft mice generated using Hep3B cells.

Fourth, our mechanistic studies are the first to report that SOR combined with DKK1 inhibitor treatment synergistically inhibited the PI3K/Akt and Wnt/β-catenin pathways by regulation of GSK3β in HCC. Although DKK1 is known to inhibit Wnt, Yu et al. [24] showed that DKK1 expression was highly associated with cytosolic or nuclear β-catenin levels in patients with HCC [18, 24]. Zhang et al. [61] showed that the expression levels of WNT1 and β-catenin were lower in DKK1-knockdown HCC cells than in control cells. Our current study showed that SOR combined with DKK1 inhibitor significantly decreased active β-catenin expression and Wnt/β-catenin pathway targeted genes, significantly better than SOR alone. Lachenmayer et al. [62] showed that SOR abolished the nuclear translocation of β-catenin in LiCl-treated Huh7 cells and decreased β-catenin expression in HepG2 cells. These findings showed that SOR combined with DKK1 inhibitor synergistically inhibited Wnt/β-catenin pathway in HCC. In addition, it has been frequently reported that the negative feedback loop of Wnt/β-catenin pathway may be disrupted in HCC because of mutations in the genes involved in Wnt/β-catenin pathway [23, 63]. Therefore, we speculated that other signaling components might contribute to the inhibition of β-catenin.

Because we previously found that DKK1 increased p-Akt expression [29] and activated VEGFR2-mediated PI3K/Akt pathway [28], PI3K/Akt pathway was explored to further investigate mechanism roles of DKK1 inhibitor on Wnt/β-catenin pathway. Kimura et al. [27] found that the inhibition of DKK1 using antibodies decreased p-Akt expression in pancreatic ductal adenocarcinoma cells, and Lyros et al. [64] found that DKK1 inhibition using siRNA decreased p-Akt expression in esophageal adenocarcinoma cells. Zhang et al. [65] found that SOR decreased the expression of PI3K, Akt, and mTOR in human hepatoma SMMC-7721 cells. Uusurprisingly, our study showed that SOR combined with DKK1 inhibitor significantly inhibited the PI3K/Akt pathway in vitro and in vivo. Additionally, we also found changes of GSK3β activity treated by SOR combined with DKK1 inhibitor. Many studies have reported that GSK3β contributes to both the PI3K/Akt and Wnt/β-catenin pathways and that p-GSK3β-Ser9 induces the inhibition of GSK3β activitiy, resulting in β-catenin translocation into the nucleus, in contrast to p-GSK3β-Tyr216 [66–71]. In the current study, the expression of p-GSK3β-Ser9 decreased, whereas that of p-GSK3β-Tyr216 increased, after treatment with SOR combined with DKK1 inhibitor. Furthermore, we showed that SOR combined with PI3K inhibitor treatment decreased active β-catenin expression through changes of GSK3β activity. These findings showed that SOR combined with DKK1 inhibitor synergistically inhibited the PI3K/Akt and Wnt/β-catenin pathways by regulation of GSK3β in HCC.

Fifth, we found that the PI3K/Akt pathway are correlated with Wnt/β-catenin pathway through GKS3β in HCC. Kaidanovich-Beilin et al. [72] found that GSK3β had different intracellular pools to regulate PI3K/Akt and Wnt/β-catenin pathways. McManus et al. [73] found that Akt-induced p-GSK3β-Ser9 was no association with Wnt-induced GSK3β inhibition. Conversely, Ding et al. [74] suggested that hyperactivation of Akt and active Wnt/β-catenin pathway promoted β-catenin activation through GSK3β inhibition and Fleming-de-Moraes et al. [75] found that activation of PI3K/Akt and Wnt/β-catenin pathway induced by insulin like growth factor-1 or L-Wnt3a treatment synergistically accumulated nuclear β-catenin. In our current study, PI3K inhibition induced by LY294002 decreased active β-catenin expression through decreases of p-GSK3β-Ser9 in HCC cells, whereas activation form of PIK3CA (PI3K^E545K^) increased active β-catenin expression through increases of p-GSK3β-Ser9 in mouse liver tumors, suggesting that PI3K/Akt pathway regulated Wnt/β-catenin pathway through GSK3β. Therefore, our results support the hypothesis that PI3K/Akt pathway are connected with Wnt/β-catenin pathway.

Despite several important results, this study has some limitations. First, our results showed that Hep3B is more sensitive to sorafenib compared to Huh7, whereas some studies showed that sorafenib is more sensitive to Huh7 cells than Hep3B cells [76]. This phenomenon can be explained in part by the well-known fact that various seeding densities per well result in different IC_50_ values in the same tumor cell lines and the increased tumor cell density could be associated with increased chemo-resistance [77]. Thus, molecular mechanism of cell density-related chemo-resistance should be further considered. Second, our data revealed that SOR combined with DKK1 inhibitor inhibited PI3K/Akt pathway, but specific mechanism studies of SOR and DKK1 inhibitor on PI3K/Akt pathway should be further confirmed. Third, we showed that the DKK1 inhibitor increased the anti-tumor efficacy of LEN, but the combination treatment had no effect on the PI3K/Akt and Wnt/β-catenin pathways. In Supplementary Fig. 3C and D, LEN + WAY treatment decreased the expression levels of PI3K/Akt pathway molecules, such as p110α, p-Akt, and p-GSK3β-Ser9 (all *P* < 0.05), significantly better than LEN treatment alone, but no difference in the Wnt/β-catenin pathway was observed in Huh7 cells, and LEN + WAY treatment had no effects on PI3K/Akt and Wnt/β-catenin pathways in Hep3B cells. Although WAY significantly enhanced the anti-colony formation ability of LEN (Supplementary Fig. 1D), the action mechanism of LEN in combination with DKK1 inhibitor requires further investigation. Fourth, immunotherapy is widely used to treat diverse tumors. Although we found that DKK1 increased the expression of PD-L1 and decreased the expression of CD4 and CD8 in HCC mouse models (Supplementary Fig. 5A and B), the effects of DKK1 inhibitors on immuno-oncological therapeutics, such as anti-PD-L1 antibodies, should be further investigated in the future.

## Conclusions

In conclusion, we found that DKK1 inhibition significantly enhanced the anti-tumor efficacy of SOR by inhibiting the PI3K/Akt and Wnt/β-catenin pathways and both pathways were connected via GSK3β in HCC. Therefore, inhibition of DKK1 may be a novel therapeutic strategy for HCC.

### Supplementary Information

**Additional file 1: Supplementary Figure 1.** The effects of LEN, WAY or their combination treatment on cell viability or colony formation in HCC cells. **Supplementary Figure 2.** Combination effects of SOR + WAY treatment on tumor progression in xenograft mouse model. **Supplementary Figure 3.** SOR + WAY treatment regulates PI3K/Akt and Wnt/β-catenin pathways in HCC. **Supplementary Figure 4.** The combination effects of SOR + WAY treatment under condition of PI3K activation. **Supplementary Figure 5.** Correlation between DKK1 expression and immune cell infiltration. **Supplementary Table 1.** Primary and secondary anti-bodies used in this study. **Supplementary Table 2.** Primer sequences used in the qRT-PCR.

**Additional file 2.**

**Additional file 3.**

## Data Availability

All data generated or analysed during this study are included in its supplementary information files.

## Abbreviations

HCC: Hepatocellular carcinoma
SOR: Sorafenib
LEN: Lenvatinib
TKI: Tyrosine kinase inhibitor
VEGFR: Vascular endothelial growth factor receptor
DKK1: Dickkopf-1
PI3K: Phosphoinositide 3-kinase
Akt: Protein kinase B
IRB: Institutional Review Board
ELISA: Enzyme-linked immunosorbent assay
IHC: Immunohistochemistry
IF: Immunofluorescence
shp53: Short-hairpin RNA down-regulation p53
miRp53: Micro RNA down-regulating p53
Hep3B DKK1 KO cells: DKK1 knockout system in Hep3B cells
qRT-PCR: Quantitative real-time polymerase chain reaction
CI: Combination index
p-GSK3β-Ser9: Phosphorylation of GSK3β at Ser 9
Active β-catenin: Non-phosphorylated β-catenin
p-GSK3β-Tyr216: Phosphorylation of GSK3β at Tyr 216
