# Supplementary material for: Inhibition of Dickkopf-1 enhances the anti-tumor efficacy of sorafenib via inhibition of the PI3K/Akt and Wnt/β-catenin pathways in hepatocellular carcinoma
Source: Cell Commun Signal. 2023 Nov 27;21:339. doi: 10.1186/s12964-023-01355-2 (PMC10680194; doi:10.1186/s12964-023-01355-2)
Supplement: Supplementary file 2 — Additional file 1: Supplementary Figure 1. The effects of LEN, WAY or their combination treatment on cell viability or colony formation in HCC cells. Supplementary Figure 2. Combination effects of SOR + WAY treatment on tumor progression in xenograft mouse model. Supplementary Figure 3. SOR + WAY treatment regulates PI3K/Akt and Wnt/β-catenin pathways in HCC. Supplementary Figure 4. The combination effects of SOR + WAY treatment under condition of PI3K activation. Supplementary Figure 5. Correlation between DKK1 expression and immune cell infiltration. Supplementary Table 1. Primary and secondary anti-bodies used in this study. Supplementary Table 2. Primer sequences used in the qRT-PCR. [file 12964_2023_1355_MOESM1_ESM.pdf]

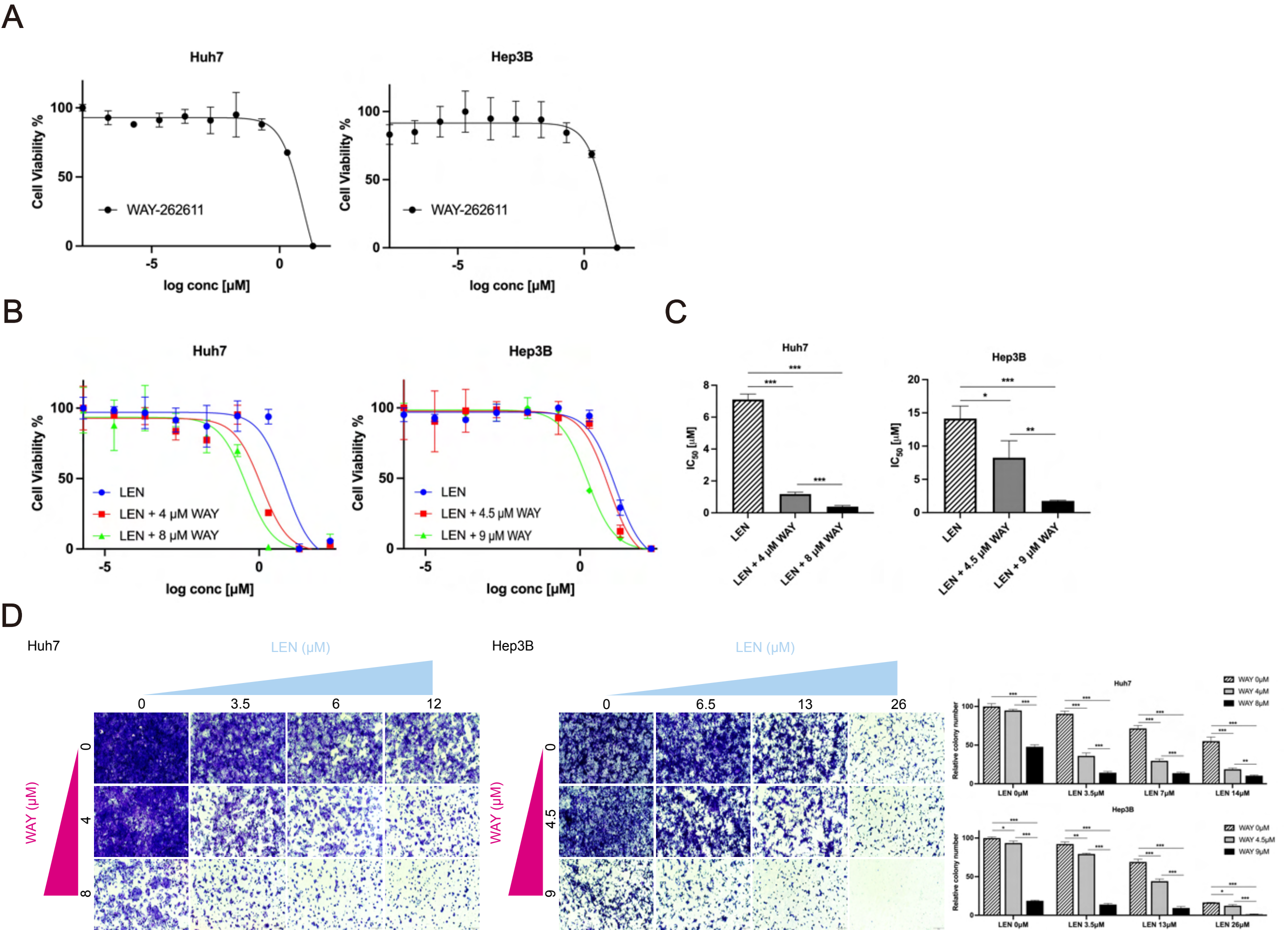

A
Huh7
Hep3B
Cell Viability %
100
50
0
-5
0
log conc [μM]
WAY-262611
B
Huh7
Hep3B
Cell Viability %
LEN
LEN + 4 μM WAY
LEN + 8 μM WAY
LEN + 4.5 μM WAY
LEN + 9 μM WAY
log conc [μM]
C
Huh7
Hep3B
IC50 [μM]
LEN
LEN + 4 μM WAY
LEN + 8 μM WAY
LEN + 4.5 μM WAY
LEN + 9 μM WAY
D
Huh7
LEN (μM)
0
3.5
6
12
WAY (μM)
0
4
8
Hep3B
LEN (μM)
0
6.5
13
26
WAY (μM)
0
4.5
9
Huh7
WAY 0μM
WAY 4μM
WAY 8μM
Relative colony number
LEN 0μM
LEN 3.5μM
LEN 7μM
LEN 14μM
Hep3B
WAY 0μM
WAY 4.5μM
WAY 9μM
Relative colony number
LEN 0μM
LEN 3.5μM
LEN 13μM
LEN 26μM

Supplementary Figure 1. The effects of LEN, WAY or their combination treatment on cell viability or colony formation in HCC cells. (A) Cell viability assay of diverse concentrations of WAY to Huh7 and Hep3B cells. (B, C) Cell viability assay of diverse concentrations of LEN and LEN combined with WAY to Huh7 and Hep3B cells. In Huh7 cells, IC_{50} values of LEN, LEN + 4 μM WAY and LEN + 8 μM WAY were 7.11 ± 0.34 μM, 1.18 ± 0.12 μM (CI=0.31, synergistic effect) and 0.38 ± 0.07 μM (CI=0.1, synergistic effect), respectively. In Hep3B cells, IC_{50} values of LEN, LEN + 4.5 μM WAY and LEN + 9 μM WAY were 13.98 ± 1.88 μM, 8.26 ± 2.54 μM and 1.74 ± 0.11 μM (CI=0.31, synergistic effect), respectively. (D) Huh7 and Hep3B cells were treated with indicated concentrations of LEN and/or WAY for 10 d. Subsequently, long-term colony formation assay was confirmed. Data are presented as the mean ± SD and performed in triplicate independently. Statistical significance is indicated by $*P < 0.05$, $**P < 0.01$, $***P < 0.001$.

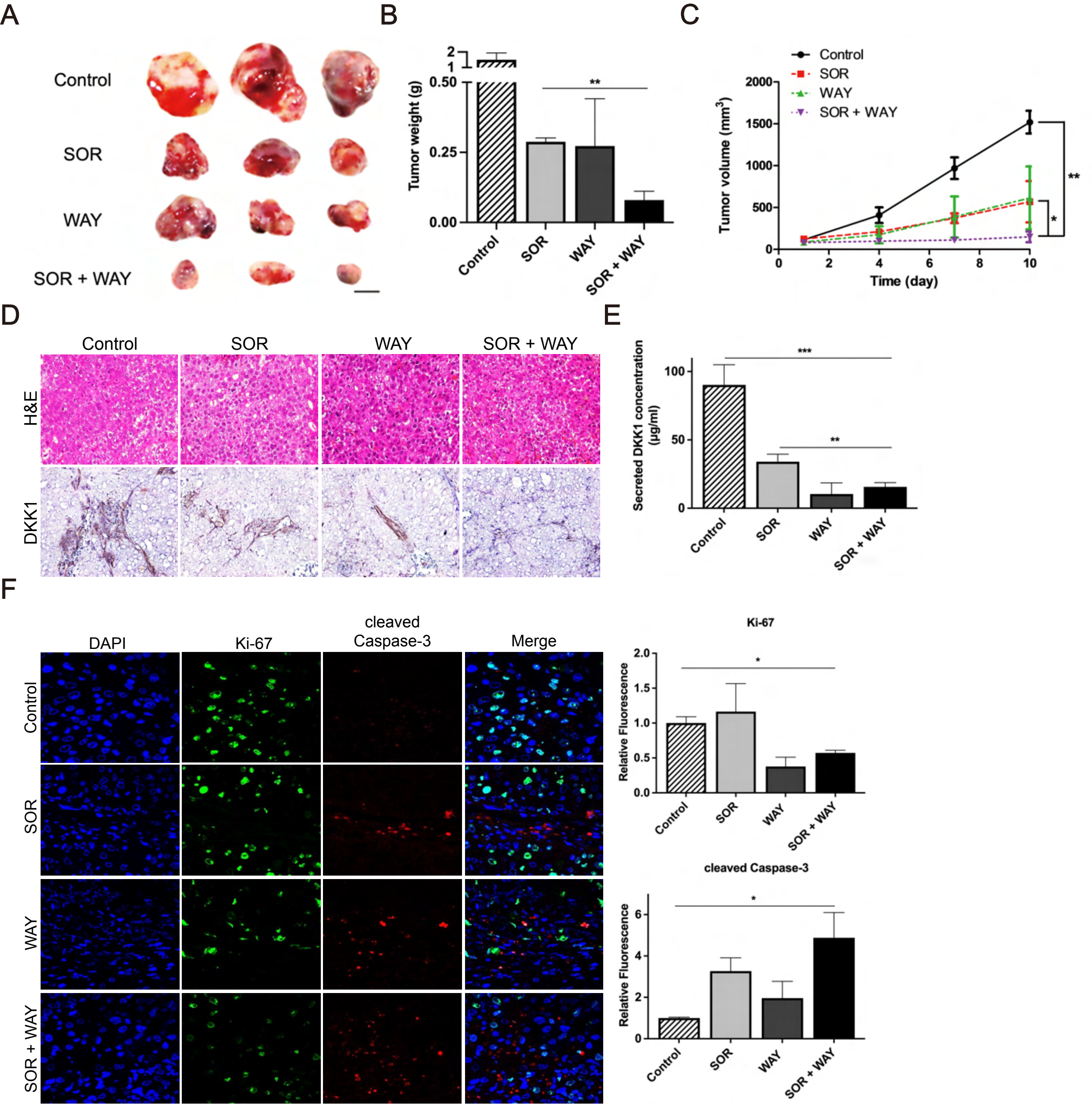

A
Control
SOR
WAY
SOR + WAY
B
Tumor weight (g)
2
1
0.50
0.25
0.00
**
Control
SOR
WAY
SOR + WAY
C
Control
SOR
WAY
SOR + WAY
Tumor volume (mm3)
2000
1500
1000
500
0
0
2
4
6
8
10
Time (day)
**
*
D
Control
SOR
WAY
SOR + WAY
H&E
DKK1
E
Secreted DKK1 concentration (μg/ml)
100
50
0
***
**
Control
SOR
WAY
SOR + WAY
F
DAPI
Ki-67
cleaved Caspase-3
Merge
Control
SOR
WAY
SOR + WAY
Ki-67
Relative Fluorescence
2.0
1.5
1.0
0.5
0.0
*
Control
SOR
WAY
SOR + WAY
cleaved Caspase-3
Relative Fluorescence
6
4
2
0
*
Control
SOR
WAY
SOR + WAY

Supplementary Figure 2. Combination effects of SOR + WAY treatment on tumor progression in xenograft mouse model. (A) Images of SOR and/or WAY treated xenograft tumors (scale bar = 6 mm). (B) Tumor weights and (C) tumor volume curves on xenograft tumors. (D) H&E and IHC staining of DKK1 in xenograft tumors. (E) Serum DKK1 levels in each group of xenograft mouse. (F) IF staining of Ki-67 and cleaved Caspase-3 in xenograft mouse. Data are presented as the mean ± SD and performed in triplicate independently. Statistical significance is indicated by *$P < 0.05$, **$P < 0.01$.

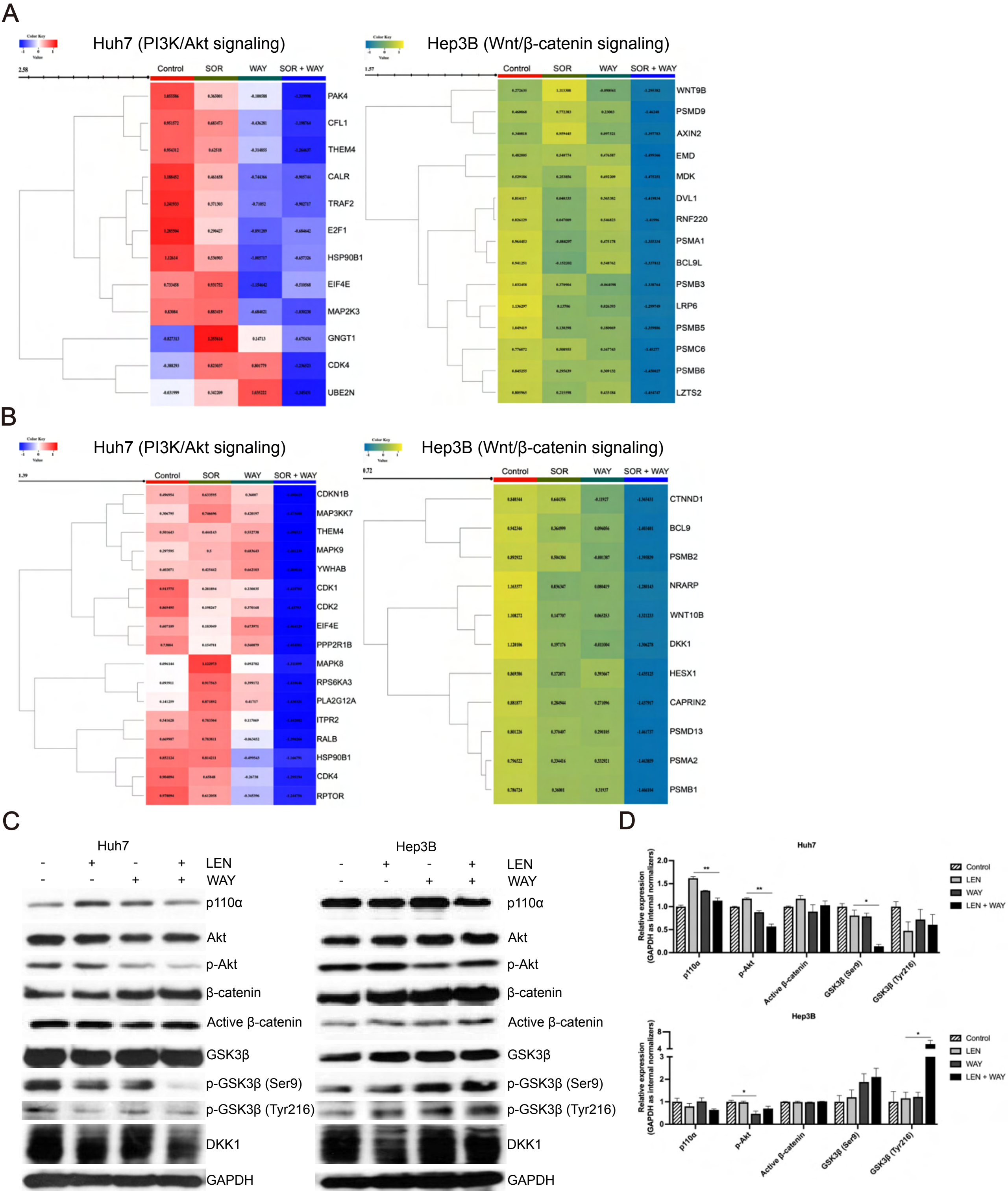
A
Huh7 (PI3K/Akt signaling)
Control
SOR
WAY
SOR + WAY
PAK4
CFL1
THEM4
CALR
TRAF2
E2F1
HSP90B1
EIF4E
MAP2K3
GNGT1
CDK4
UBE2N
Hep3B (Wnt/β-catenin signaling)
WNT9B
PSMD9
AXIN2
EMD
MDK
DVL1
RNF220
PSMA1
BCL9L
PSMB3
LRP6
PSMB5
PSMC6
PSMB6
LZTS2
B
Huh7 (PI3K/Akt signaling)
CDKN1B
MAP3KK7
THEM4
MAPK9
YWHAB
CDK1
CDK2
EIF4E
PPP2R1B
MAPK8
RPS6KA3
PLA2G12A
ITPR2
RALB
HSP90B1
CDK4
RPTOR
Hep3B (Wnt/β-catenin signaling)
CTNND1
BCL9
PSMB2
NRARP
WNT10B
DKK1
HESX1
CAPRIN2
PSMD13
PSMA2
PSMB1
C
Huh7
Hep3B
LEN
WAY
p110α
Akt
p-Akt
β-catenin
Active β-catenin
GSK3β
p-GSK3β (Ser9)
p-GSK3β (Tyr216)
DKK1
GAPDH
D
Huh7
Relative expression (GAPDH as internal normalizers)
Control
LEN
WAY
LEN + WAY
p110α
p-Akt
Active β-catenin
GSK3β (Ser9)
GSK3β (Tyr216)
Hep3B

Supplementary Figure 3. SOR + WAY treatment regulates PI3K/Akt and Wnt/β-catenin pathways in HCC. (A, B) Huh7 and Hep3B cells were treated with IC_{50} values of SOR and/or WAY for 24 h. Subsequently, heatmaps of differentially expressed PI3K/Akt and Wnt/β-catenin pathways associated genes were confirmed in Huh7 and Hep3B cells. (C, D) Huh7 and Hep3B cells were treated with IC_{50} values of LEN and/or WAY for 24 h. Subsequently, PI3K/Akt and Wnt/β-catenin pathways associated molecules were detected using western blot analysis. Data are presented as the mean ± SD and performed in triplicate independently. Statistical significance is indicated by $^{*}P < 0.05$, $^{**}P < 0.01$.

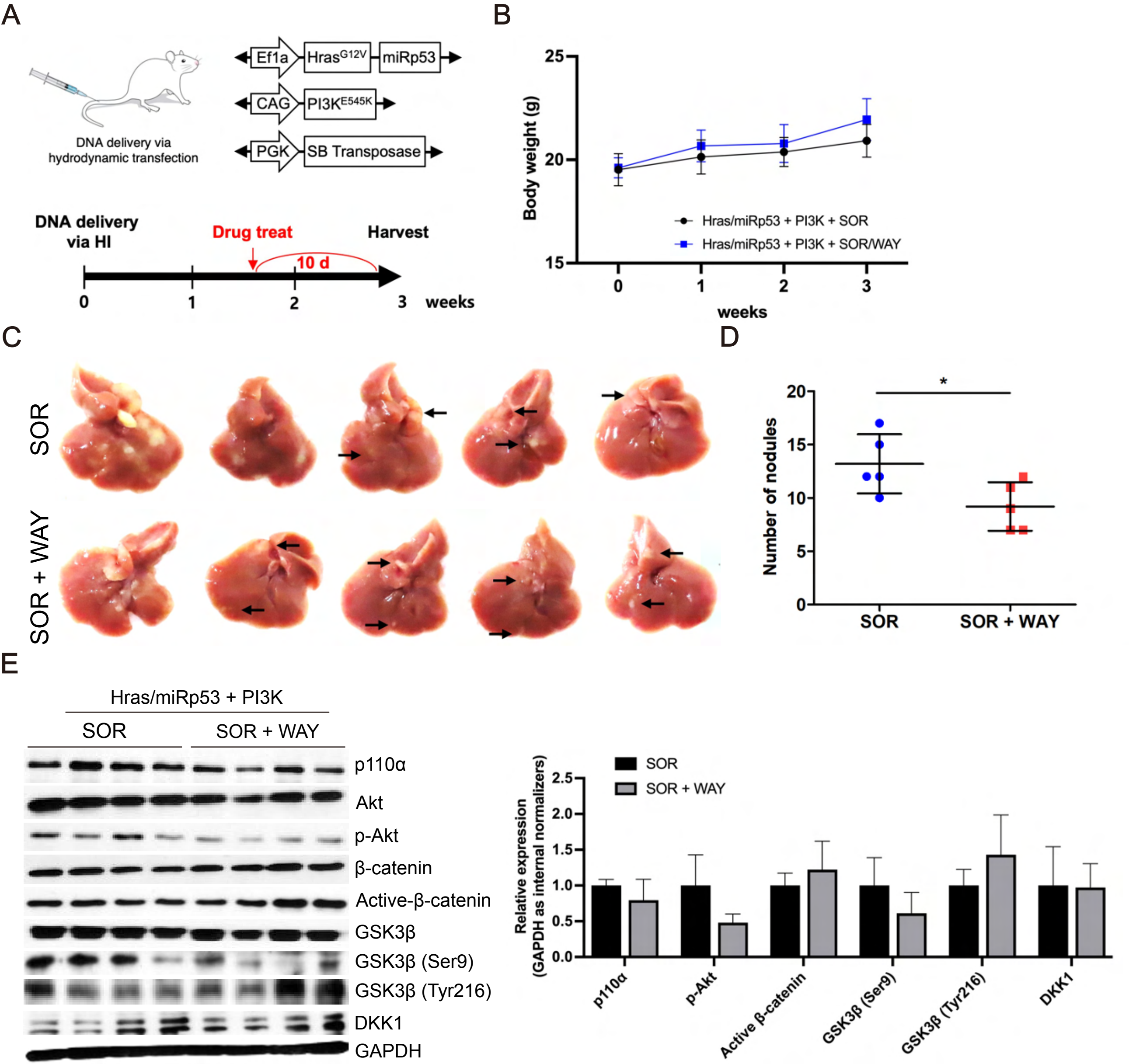

A
Ef1a
HrasG12V
miRp53
CAG
PI3KE545K
PGK
SB Transposase
DNA delivery via hydrodynamic transfection
DNA delivery via HI
Drug treat
Harvest
10 d
0
1
2
3
weeks
B
Body weight (g)
25
20
15
0
1
2
3
weeks
Hras/miRp53 + PI3K + SOR
Hras/miRp53 + PI3K + SOR/WAY
C
SOR
SOR + WAY
D
Number of nodules
20
15
10
5
0
*
SOR
SOR + WAY
E
Hras/miRp53 + PI3K
SOR
SOR + WAY
p110α
Akt
p-Akt
β-catenin
Active-β-catenin
GSK3β
GSK3β (Ser9)
GSK3β (Tyr216)
DKK1
GAPDH
Relative expression (GAPDH as internal normalizers)
2.5
2.0
1.5
1.0
0.5
0.0
SOR
SOR + WAY
p110α
p-Akt
Active β-catenin
GSK3β (Ser9)
GSK3β (Tyr216)
DKK1

Supplementary Figure 4. The combination effects of SOR + WAY treatment under condition of PI3K activation. (A) Mice were transfected with $Hras^{G12V}$, miRp53 and $PI3K^{E545K}$ through hydrodynamic tail vein injection. Mice were treated with SOR or SOR + WAY for 10 d (each group, n=5). (B) Body weight was measured every week for 3 weeks. (C) Representative liver pictures of each group. (D) Number of nodules in mouse liver of each group was counted. (E) PI3K/Akt and Wnt/β-catenin pathways associated molecules were detected using western blot analysis in mouse liver tissues. Data are presented as the mean ± SD and performed in triplicate independently. Statistical significance is indicated by $^*P < 0.05$.

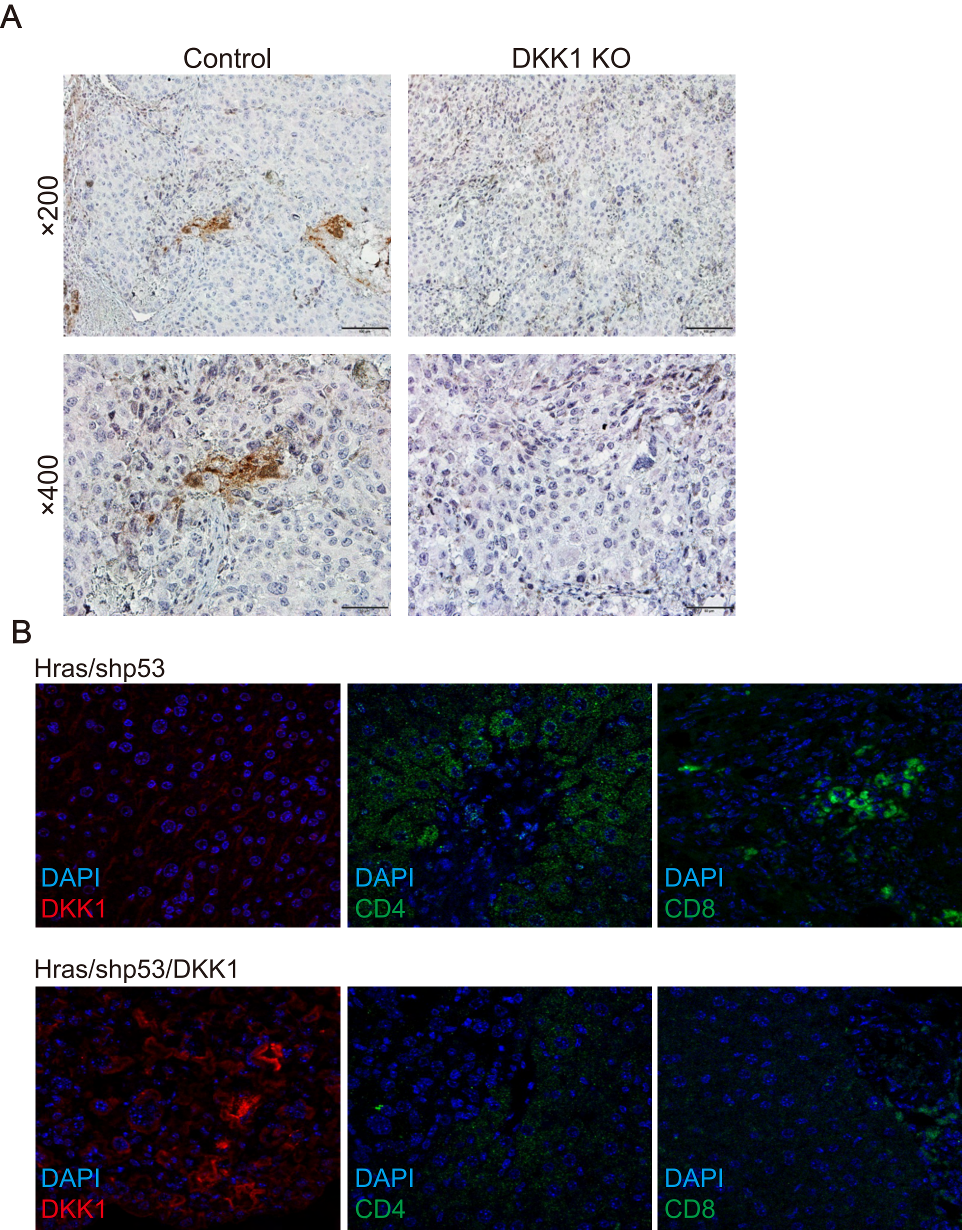
A
Control
DKK1 KO
×200
×400
B
Hras/shp53
DAPI
DKK1
DAPI
CD4
DAPI
CD8
Hras/shp53/DKK1
DAPI
DKK1
DAPI
CD4
DAPI
CD8

Supplementary Figure 5. Correlation between DKK1 expression and immune cell infiltration. (A) IHC staining of PD-L1 in xenograft mice generated using Hep3B cells or Hep3B DKK1 KO cells. (B) IF staining of DKK1, CD4 and CD8 in mice transfected with $Hras^{G12V}$, shp53 or DKK1.

Supplementary Table 1. Primary and secondary anti-bodies used in this study

Antibodies	Company	Product Number	Dilution
DKK1	R&D Systems<br>Santa Cruz Biotechnology	AF1096<br>SC374574	1:200 (WB)<br>1:50 (IHC, IF)
cleaved Caspase-3	Cell Signaling Technology	9661	1:1000 (WB)<br>1:400 (IF)
cleaved PARP	Cell Signaling Technology	9544	1:1000 (WB)
P110α	Cell Signaling Technology	4249	1:1000 (WB)
Akt	Cell Signaling Technology	9272	1:1000 (WB)
p-Akt	Cell Signaling Technology	4060	1:2000 (WB)<br>1:400 (IF)
β-catenin	Cell Signaling Technology	8480	1:1000 (WB)
Active β-catenin	Cell Signaling Technology	8814	1:1000 (WB)<br>1:800 (IF)
GSK3β	Cell Signaling Technology	9315	1:1000 (WB)
p-GSK3β (Ser9)	Abcam	ab107166	1:1000 (WB, IHC)
p-GSK3β (Tyr216)	Abcam	Ab75745	1:1000 (WB)<br>1:200 (IHC)
GAPDH	Cell Signaling Technology	2118	1:2000 (WB)
Ki-67	Cell Signaling Technology	9449	1:800 (IF)
Vimentin	Cell Signaling Technology	5741	1:100 (IF)
N-cadherin	Cell Signaling Technology	13116	1:200 (IF)
E-cadherin	Cell Signaling Technology	14472	1:200 (IF)
Cyclin D1	Cell Signaling Technology	2978	1:1000 (WB)
Goat Anti-Rabbit IgG	Dako	P0448	1:2000<br>(WB, IHC, IF)
Rabbit Anti-Goat IgG	Dako	P0449	1:2000 (WB)
Horse Anti-Mouse IgG	Cell Signaling Technology	7076	1:2000 (IF)

Supplementary Figure 2. Primer sequences used in the qRT-PCR

Gene name	Type	5′-3′
E2F1	Forward	ACGTGACGTGTCAGGACCT
	Reverse	GATCGGGCCTTGTTTGCTCTT
MCM3	Forward	TCTAAGCCGCCATTTCGATTT
	Reverse	AAGACGCTGGAAAGCTGGATA
MCM6	Forward	GAGGAACTGATTCGTCCTGAGA
	Reverse	CAAGGCCCGACACAGGTAAG
CDK1	Forward	AAACTACAGGTCAAGTGGTAGCC
	Reverse	TCCTGCATAAGCACATCCTGA
CDKN1B	Forward	TAATTGGGGCTCCGGCTAACT
	Reverse	TGCAGGTCGCTTCCTTATTCC
GADD45	Forward	TACGAGTCGGCCAAGTTGATG
	Reverse	GGATGAGCGTGAAGTGGATTT
SOD2	Forward	GGAAGCCATCAAACGTGACTT
	Reverse	CCCGTTCCTTATTGAAACCAAGC
AXIN2	Forward	CAACACCAGGCGGAACGAA
	Reverse	GCCCAATAAGGAGTGTAAGGACT
c-Myc	Forward	AATAGAGCTGCTTCGCCTAGA
	Reverse	GAGGTGGTTCATACTGAGCAAG
Twist	Forward	GCCTAGAGTTGCCGACTTATG
	Reverse	TGCGTTTCCTGTTAAGGTAGC
MMP2	Forward	TGACTTTCTTGGATCGGGTCG
	Reverse	AAGCACCACATCAGATGACTG
