# Supplementary material for: Inhibition of Dickkopf-1 enhances the anti-tumor efficacy of sorafenib via inhibition of the PI3K/Akt and Wnt/β-catenin pathways in hepatocellular carcinoma
Source: Cell Commun Signal. 2023 Nov 27;21:339. doi: 10.1186/s12964-023-01355-2 (PMC10680194; doi:10.1186/s12964-023-01355-2)
Supplement: Supplementary file 3 — Additional file 2. [file 12964_2023_1355_MOESM2_ESM.zip › raw data/Figure 3/Figure 3A_Hep3B.pdf]

BD FACSDiva 8.0.2

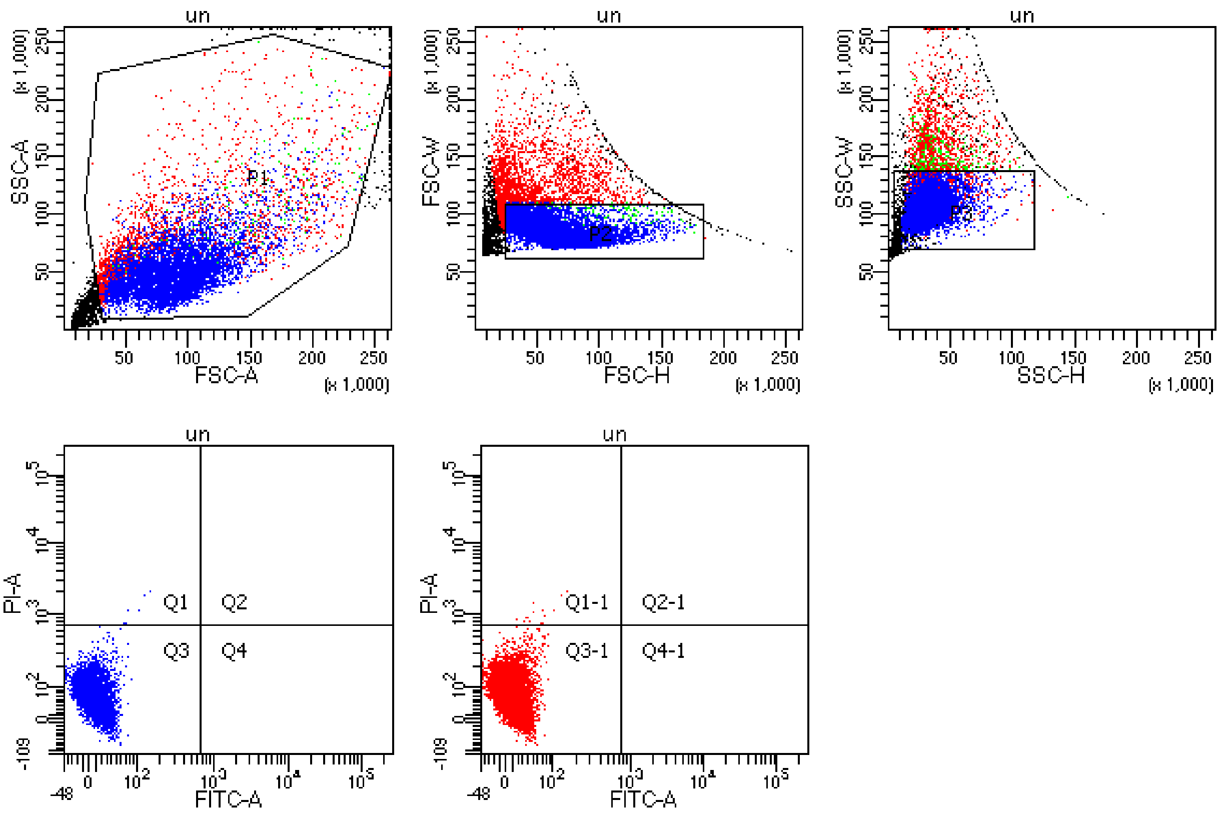

Tube: un

Population	#Events	%Parent	%Total
All Events	10,000	####	100.0
P1	8,545	85.4	85.4
P2	6,272	73.4	62.7
P3	6,065	96.7	60.7
Q1	6	0.1	0.1
Q2	0	0.0	0.0
Q3	6,059	99.9	60.6
Q4	0	0.0	0.0
Q1-1	16	0.2	0.2
Q2-1	0	0.0	0.0
Q3-1	8,529	99.8	85.3
Q4-1	0	0.0	0.0

BD FACSDiva 8.0.2

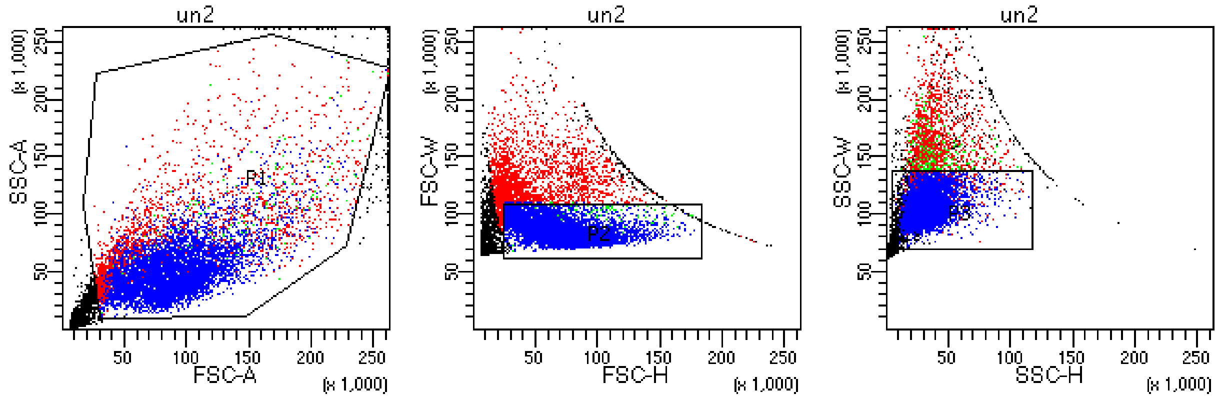

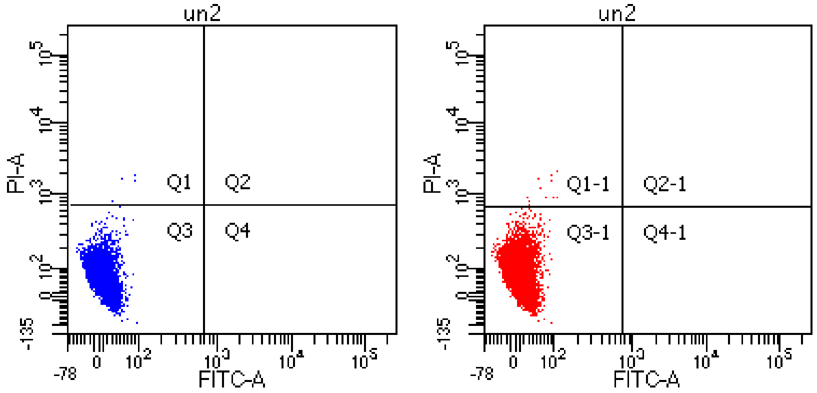

Tube: un2

Population	#Events	%Parent	%Total
All Events	10,000	####	100.0
P1	8,481	84.8	84.8
P2	6,239	73.6	62.4
P3	6,049	97.0	60.5
Q1	4	0.1	0.0
Q2	0	0.0	0.0
Q3	6,045	99.9	60.4
Q4	0	0.0	0.0
Q1-1	11	0.1	0.1
Q2-1	0	0.0	0.0
Q3-1	8,470	99.9	84.7
Q4-1	0	0.0	0.0

BD FACSDiva 8.0.2

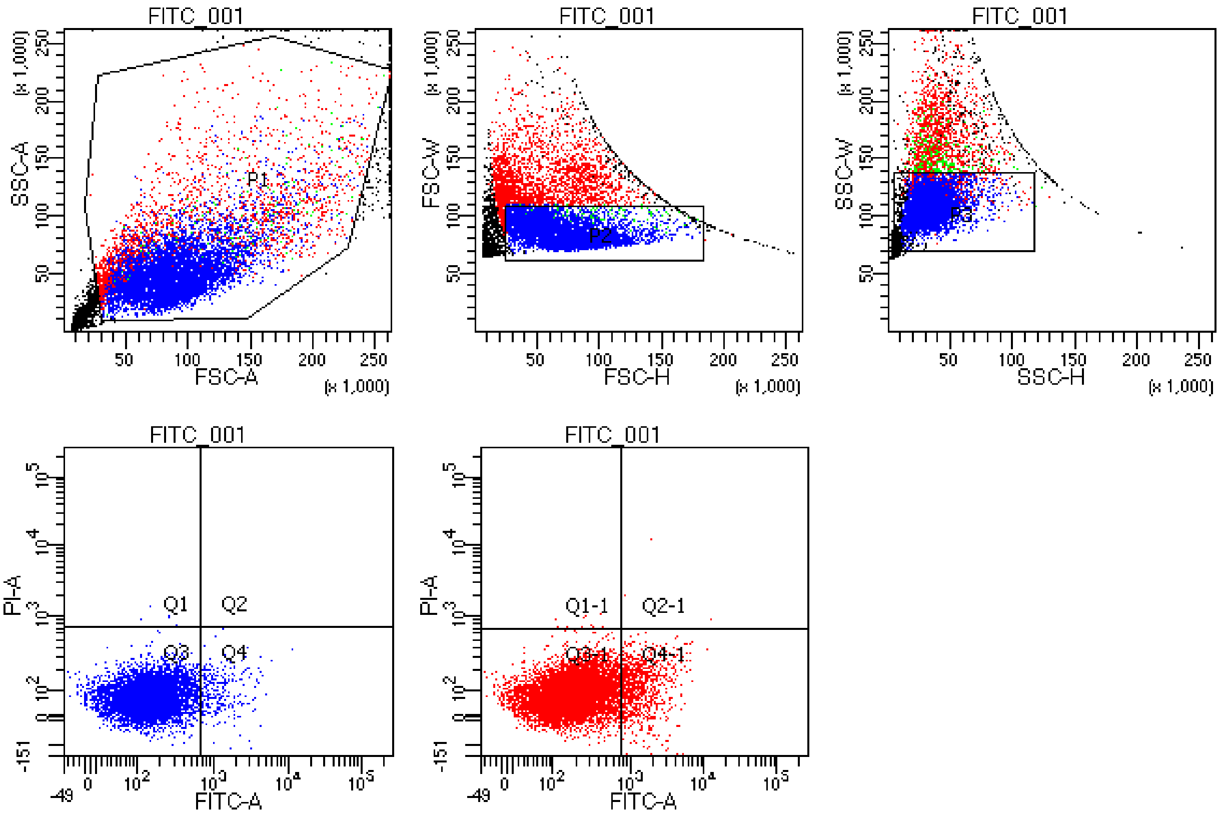

Tube: FITC_001

Population	#Events	%Parent	%Total
All Events	10,000	####	100.0
P1	8,515	85.2	85.2
P2	6,105	71.7	61.1
P3	5,898	96.6	59.0
Q1	6	0.1	0.1
Q2	0	0.0	0.0
Q3	5,639	95.6	56.4
Q4	253	4.3	2.5
Q1-1	11	0.1	0.1
Q2-1	3	0.0	0.0
Q3-1	7,540	88.5	75.4
Q4-1	961	11.3	9.6

BD FACSDiva 8.0.2

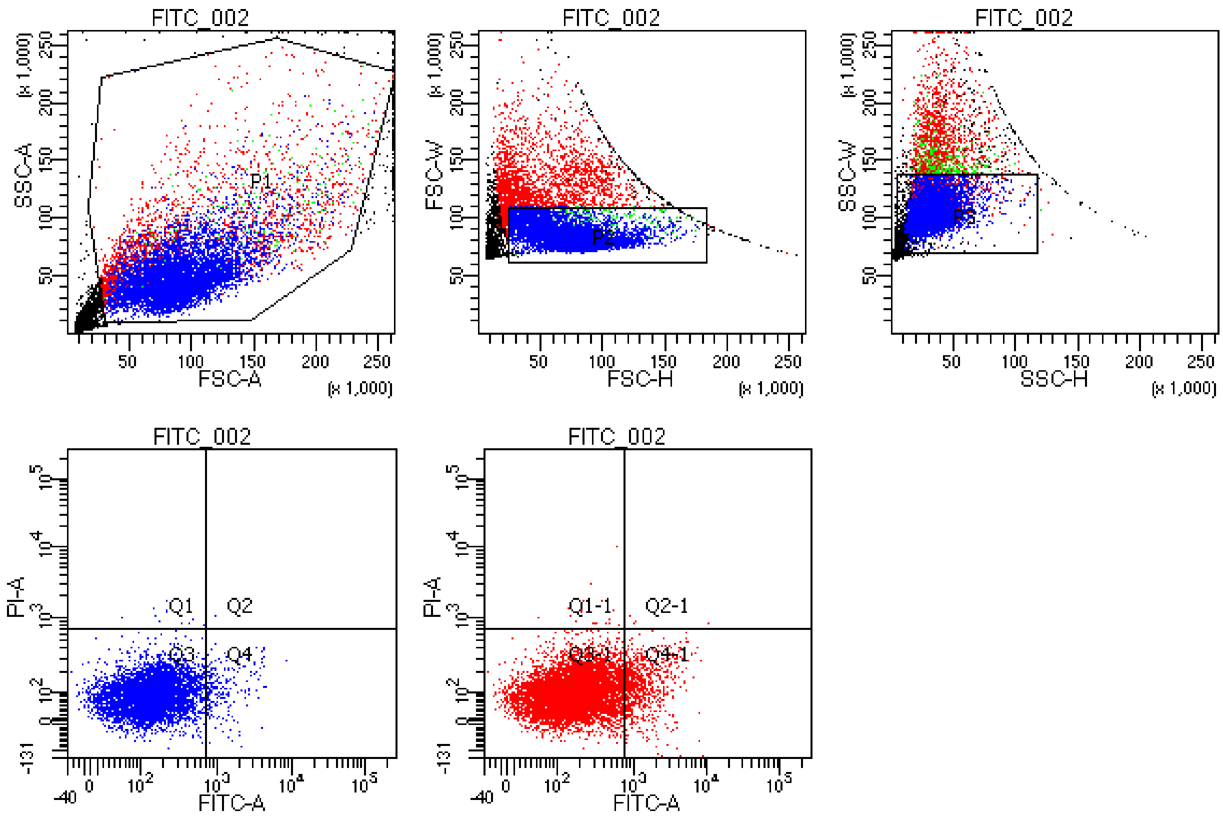

Tube: FITC_002

Population	#Events	%Parent	%Total
All Events	10,000	####	100.0
P1	8,466	84.7	84.7
P2	6,229	73.6	62.3
P3	5,991	96.2	59.9
Q1	12	0.2	0.1
Q2	1	0.0	0.0
Q3	5,771	96.3	57.7
Q4	207	3.5	2.1
Q1-1	19	0.2	0.2
Q2-1	4	0.0	0.0
Q3-1	7,688	90.8	76.9
Q4-1	755	8.9	7.6

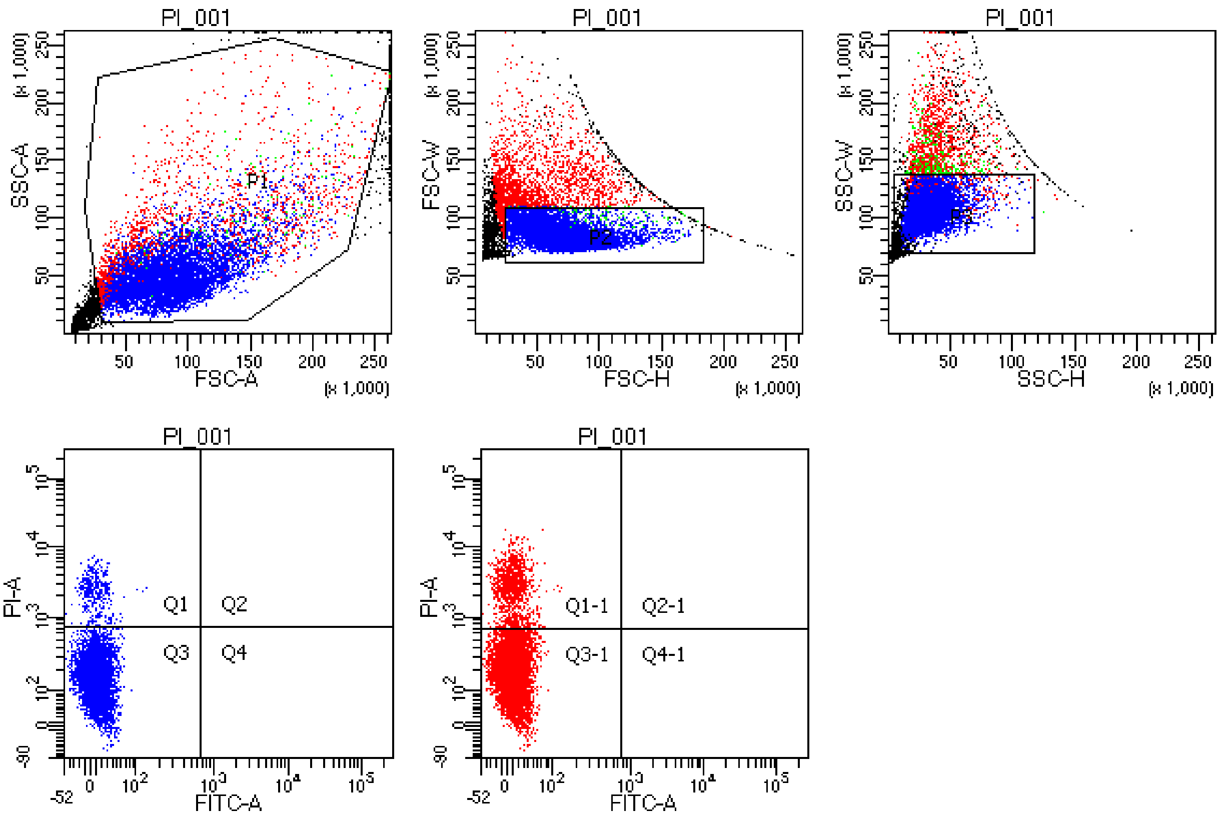

Tube: PI_001

Population	#Events	%Parent	%Total
All Events	10,000	####	100.0
P1	8,383	83.8	83.8
P2	6,190	73.8	61.9
P3	5,987	96.7	59.9
Q1	356	5.9	3.6
Q2	0	0.0	0.0
Q3	5,631	94.1	56.3
Q4	0	0.0	0.0
Q1-1	1,267	15.1	12.7
Q2-1	0	0.0	0.0
Q3-1	7,116	84.9	71.2
Q4-1	0	0.0	0.0

BD FACSDiva 8.0.2

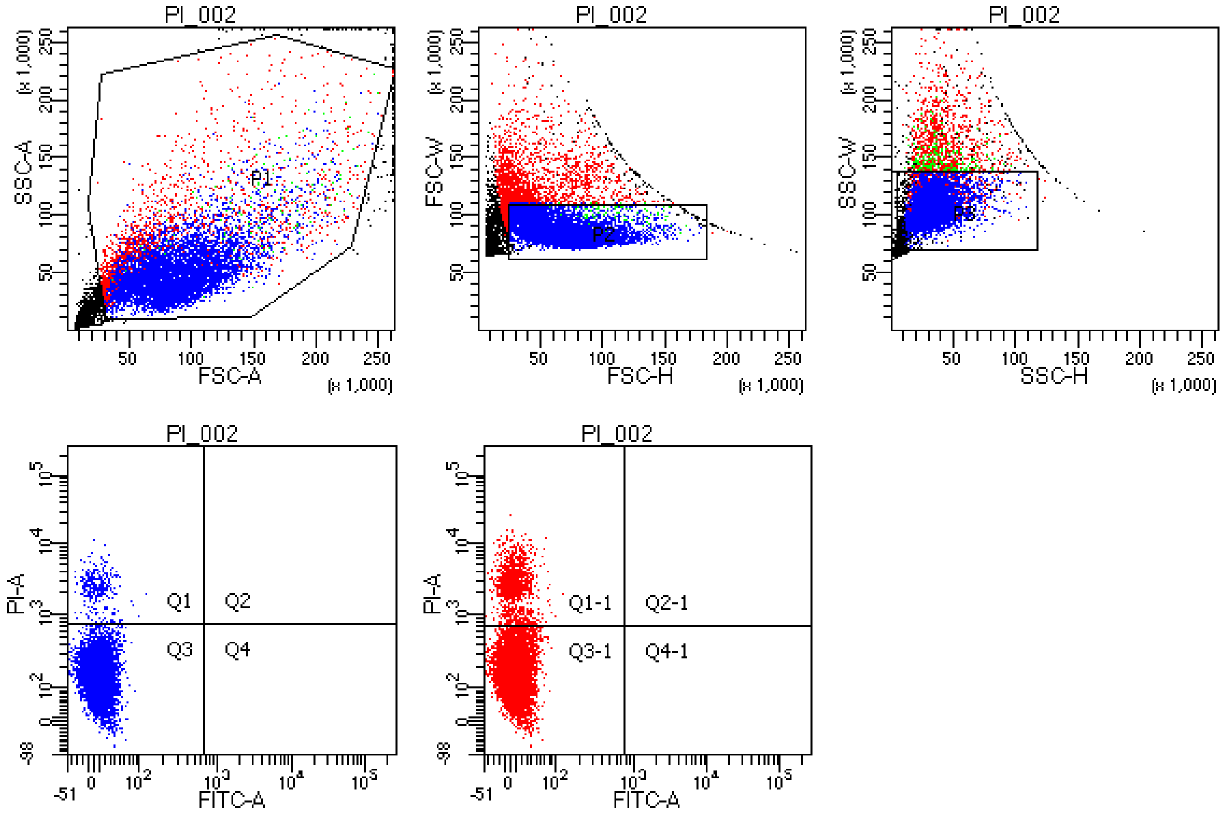

Tube: PI_002

Population	#Events	%Parent	%Total
All Events	10,000	####	100.0
P1	8,406	84.1	84.1
P2	6,355	75.6	63.6
P3	6,157	96.9	61.6
Q1	319	5.2	3.2
Q2	0	0.0	0.0
Q3	5,838	94.8	58.4
Q4	0	0.0	0.0
Q1-1	1,184	14.1	11.8
Q2-1	0	0.0	0.0
Q3-1	7,222	85.9	72.2
Q4-1	0	0.0	0.0

BD FACSDiva 8.0.2

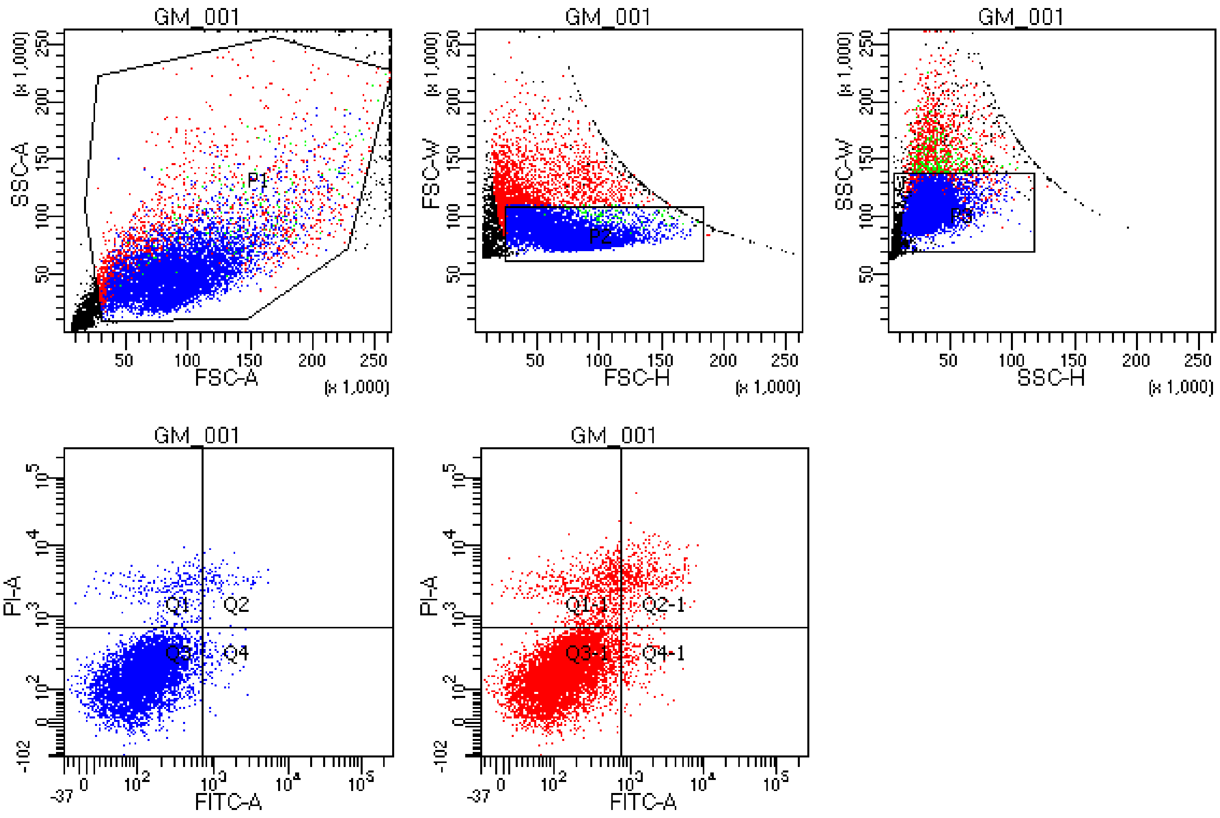

Tube: GM_001

Population	#Events	%Parent	%Total
All Events	10,000	####	100.0
P1	8,577	85.8	85.8
P2	6,603	77.0	66.0
P3	6,408	97.0	64.1
Q1	268	4.2	2.7
Q2	89	1.4	0.9
Q3	5,975	93.2	59.8
Q4	76	1.2	0.8
Q1-1	759	8.8	7.6
Q2-1	415	4.8	4.2
Q3-1	7,229	84.3	72.3
Q4-1	174	2.0	1.7

BD FACSDiva 8.0.2

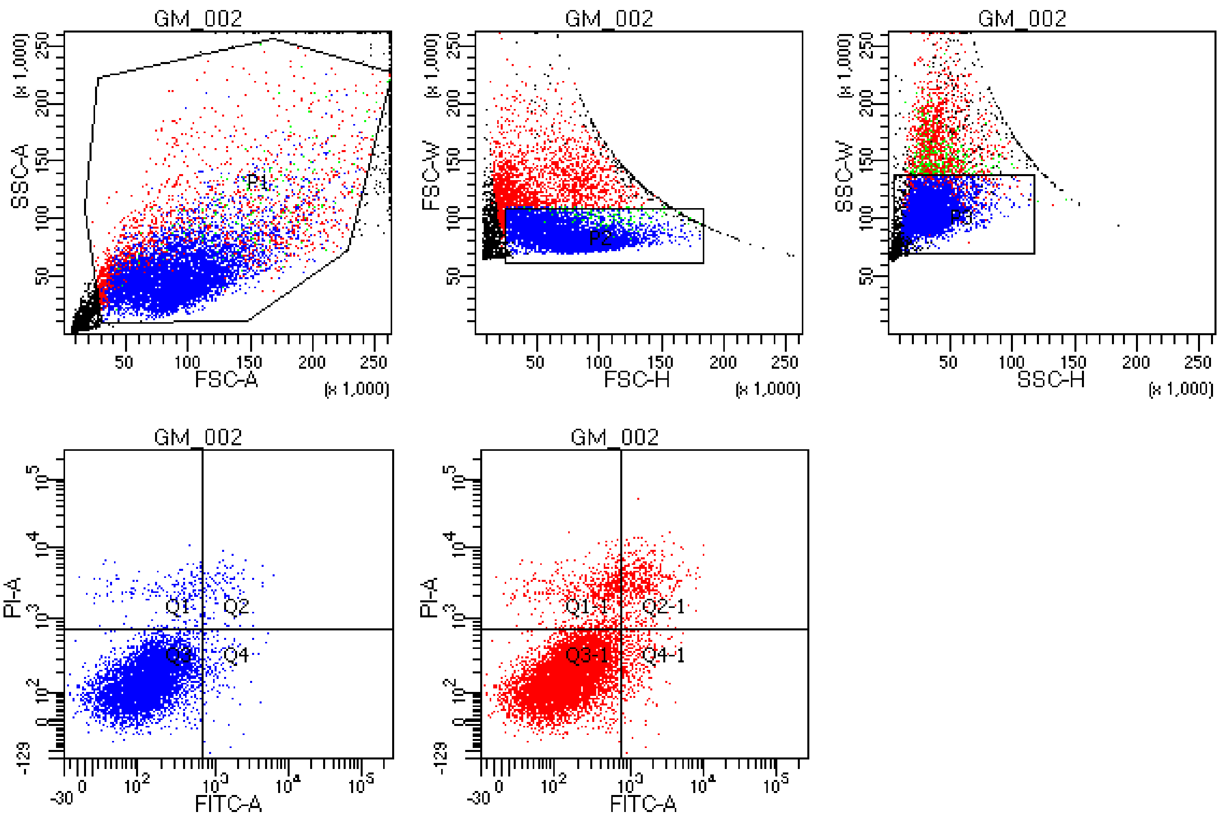

Tube: GM_002

Population	#Events	%Parent	%Total
All Events	10,000	####	100.0
P1	8,461	84.6	84.6
P2	6,328	74.8	63.3
P3	6,093	96.3	60.9
Q1	199	3.3	2.0
Q2	102	1.7	1.0
Q3	5,666	93.0	56.7
Q4	126	2.1	1.3
Q1-1	660	7.8	6.6
Q2-1	482	5.7	4.8
Q3-1	7,044	83.3	70.4
Q4-1	275	3.3	2.8

BD FACSDiva 8.0.2

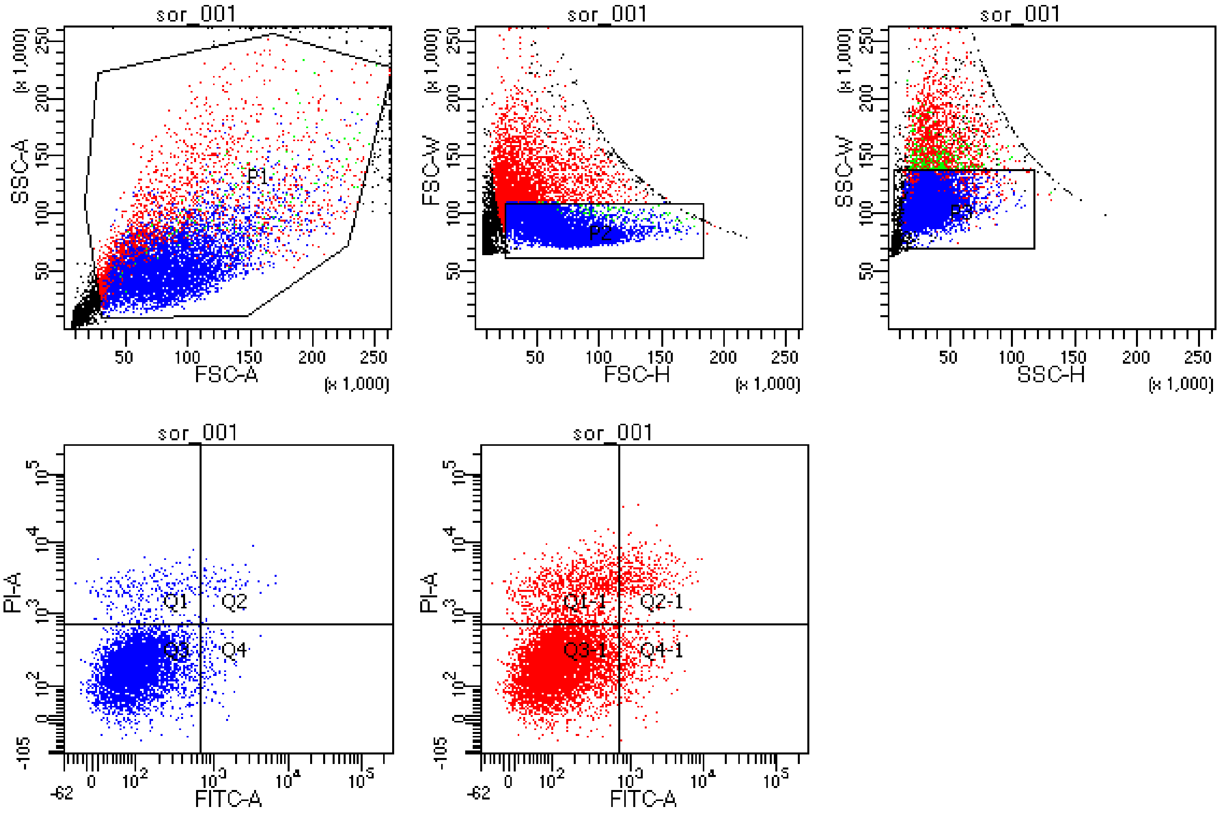

Tube: sor_001

Population	#Events	%Parent	%Total
All Events	10,000	####	100.0
P1	8,441	84.4	84.4
P2	5,333	63.2	53.3
P3	5,100	95.6	51.0
Q1	263	5.2	2.6
Q2	66	1.3	0.7
Q3	4,682	91.8	46.8
Q4	89	1.7	0.9
Q1-1	1,109	13.1	11.1
Q2-1	346	4.1	3.5
Q3-1	6,739	79.8	67.4
Q4-1	247	2.9	2.5

BD FACSDiva 8.0.2

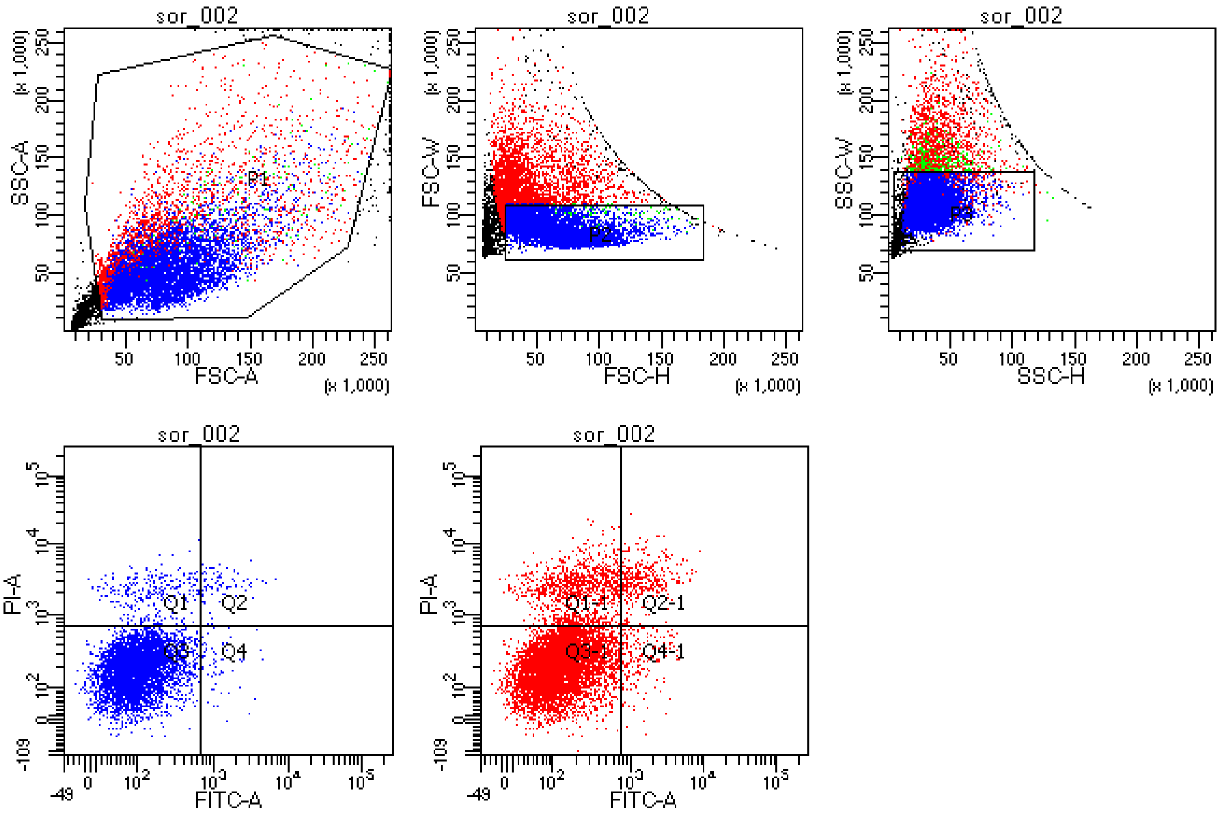

Tube: sor_002

Population	#Events	%Parent	%Total
All Events	10,000	####	100.0
P1	8,534	85.3	85.3
P2	5,416	63.5	54.2
P3	5,200	96.0	52.0
Q1	283	5.4	2.8
Q2	74	1.4	0.7
Q3	4,788	92.1	47.9
Q4	55	1.1	0.5
Q1-1	1,084	12.7	10.8
Q2-1	352	4.1	3.5
Q3-1	6,914	81.0	69.1
Q4-1	184	2.2	1.8

BD FACSDiva 8.0.2

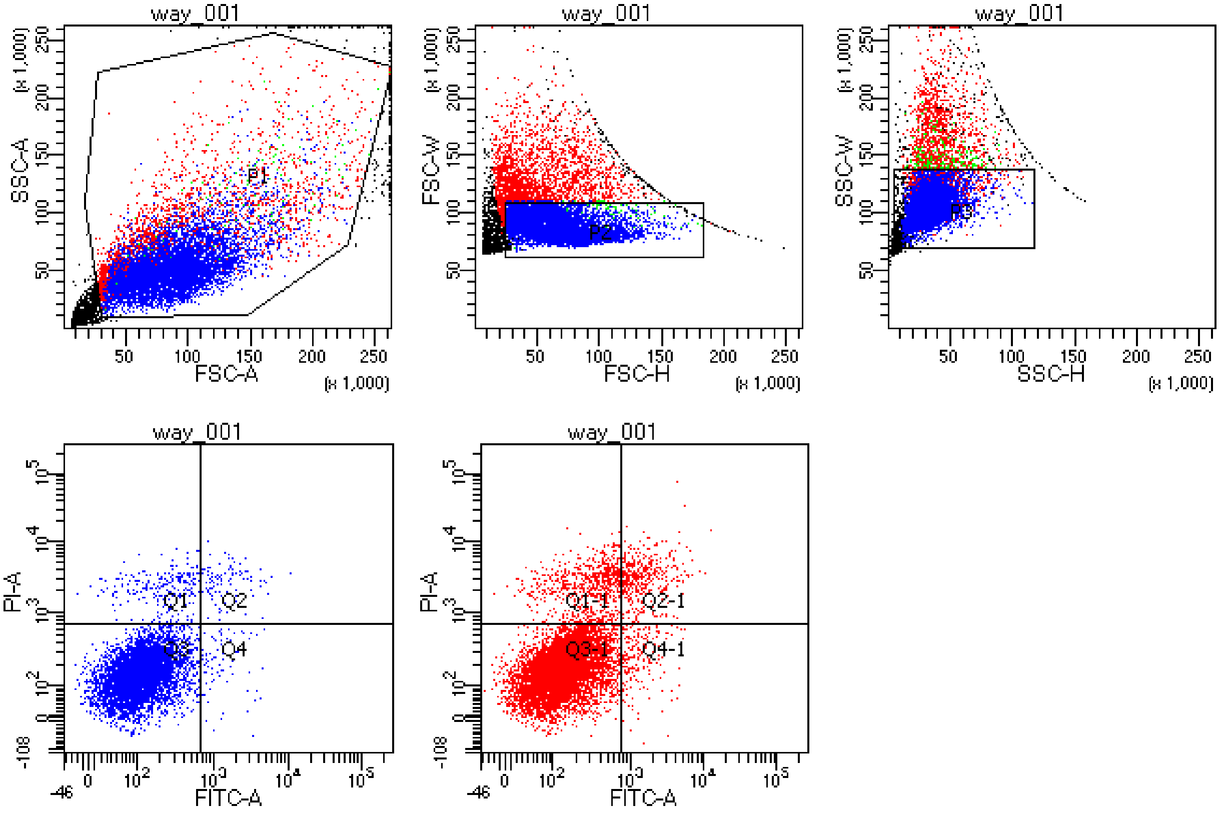

Tube: way_001

Population	#Events	%Parent	%Total
All Events	10,000	####	100.0
P1	8,615	86.2	86.2
P2	6,165	71.6	61.7
P3	6,001	97.3	60.0
Q1	237	3.9	2.4
Q2	97	1.6	1.0
Q3	5,593	93.2	55.9
Q4	74	1.2	0.7
Q1-1	726	8.4	7.3
Q2-1	418	4.9	4.2
Q3-1	7,269	84.4	72.7
Q4-1	202	2.3	2.0

BD FACSDiva 8.0.2

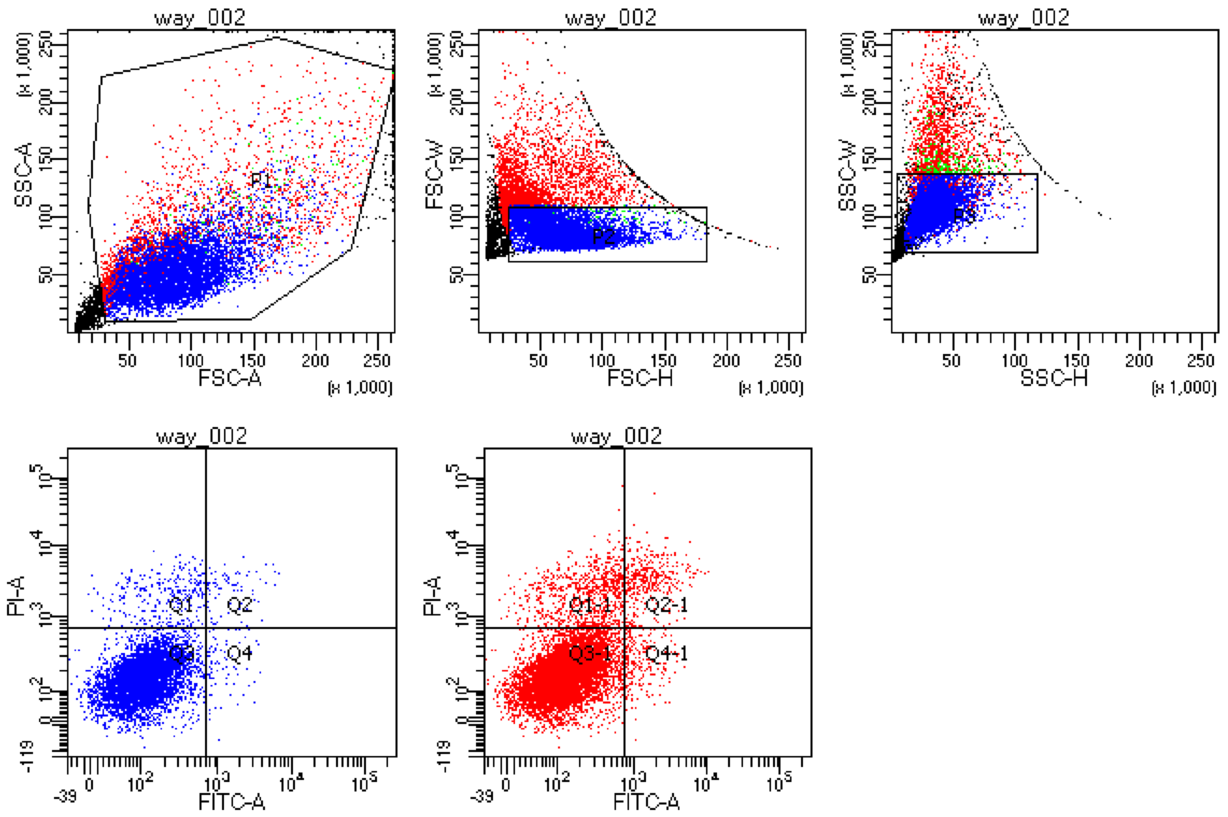

Tube: way_002

Population	#Events	%Parent	%Total
All Events	10,000	####	100.0
P1	8,607	86.1	86.1
P2	6,067	70.5	60.7
P3	5,909	97.4	59.1
Q1	267	4.5	2.7
Q2	72	1.2	0.7
Q3	5,484	92.8	54.8
Q4	86	1.5	0.9
Q1-1	854	9.9	8.5
Q2-1	397	4.6	4.0
Q3-1	7,136	82.9	71.4
Q4-1	220	2.6	2.2

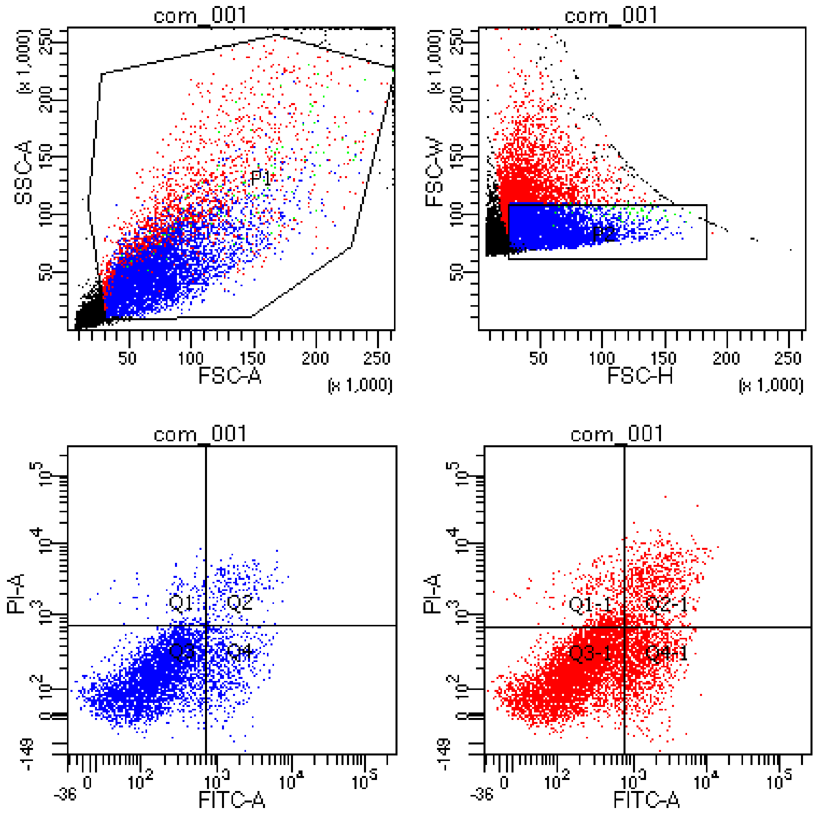

Tube: com_001

Population	#Events	%Parent	%Total
All Events	10,000	####	100.0
P1	7,498	75.0	75.0
P2	4,685	62.5	46.8
P3	4,540	96.9	45.4
Q1	121	2.7	1.2
Q2	202	4.4	2.0
Q3	3,741	82.4	37.4
Q4	476	10.5	4.8
Q1-1	395	5.3	4.0
Q2-1	794	10.6	7.9
Q3-1	5,133	68.5	51.3
Q4-1	1,176	15.7	11.8

BD FACSDiva 8.0.2

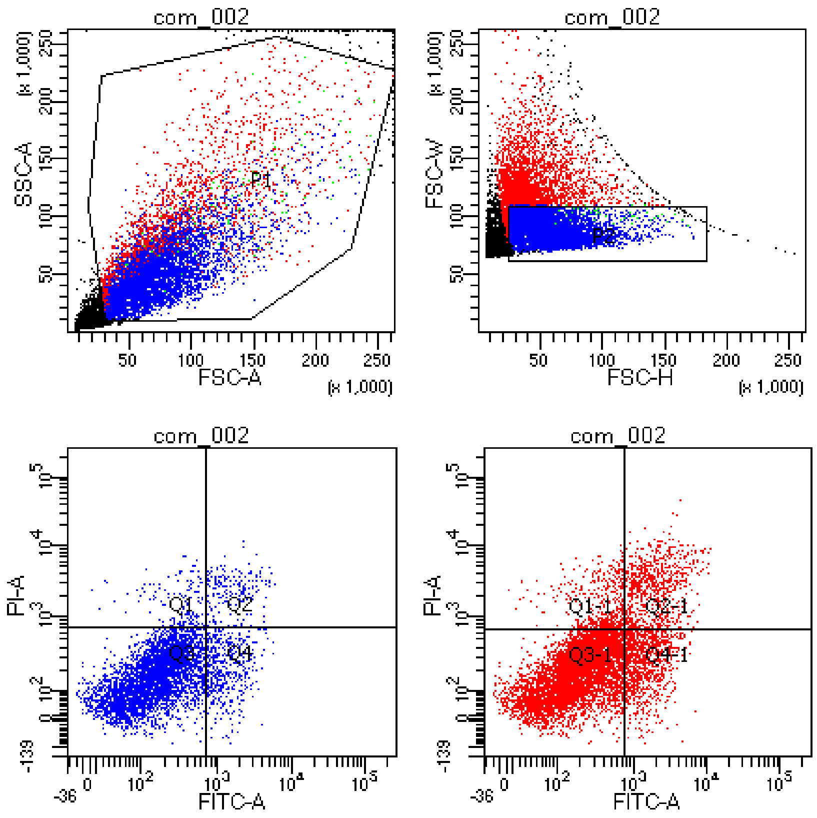

Tube: com_002

Population	#Events	%Parent	%Total
All Events	10,000	####	100.0
P1	7,662	76.6	76.6
P2	4,822	62.9	48.2
P3	4,698	97.4	47.0
Q1	155	3.3	1.6
Q2	171	3.6	1.7
Q3	3,917	83.4	39.2
Q4	455	9.7	4.6
Q1-1	483	6.3	4.8
Q2-1	760	9.9	7.6
Q3-1	5,391	70.4	53.9
Q4-1	1,028	13.4	10.3
