# Supplementary material for: Inhibition of Dickkopf-1 enhances the anti-tumor efficacy of sorafenib via inhibition of the PI3K/Akt and Wnt/β-catenin pathways in hepatocellular carcinoma
Source: Cell Commun Signal. 2023 Nov 27;21:339. doi: 10.1186/s12964-023-01355-2 (PMC10680194; doi:10.1186/s12964-023-01355-2)
Supplement: Supplementary file 3 — Additional file 2. [file 12964_2023_1355_MOESM2_ESM.zip › raw data/Figure 3/Figure 3A_Huh7.pdf]

BD FACSDiva 8.0.2

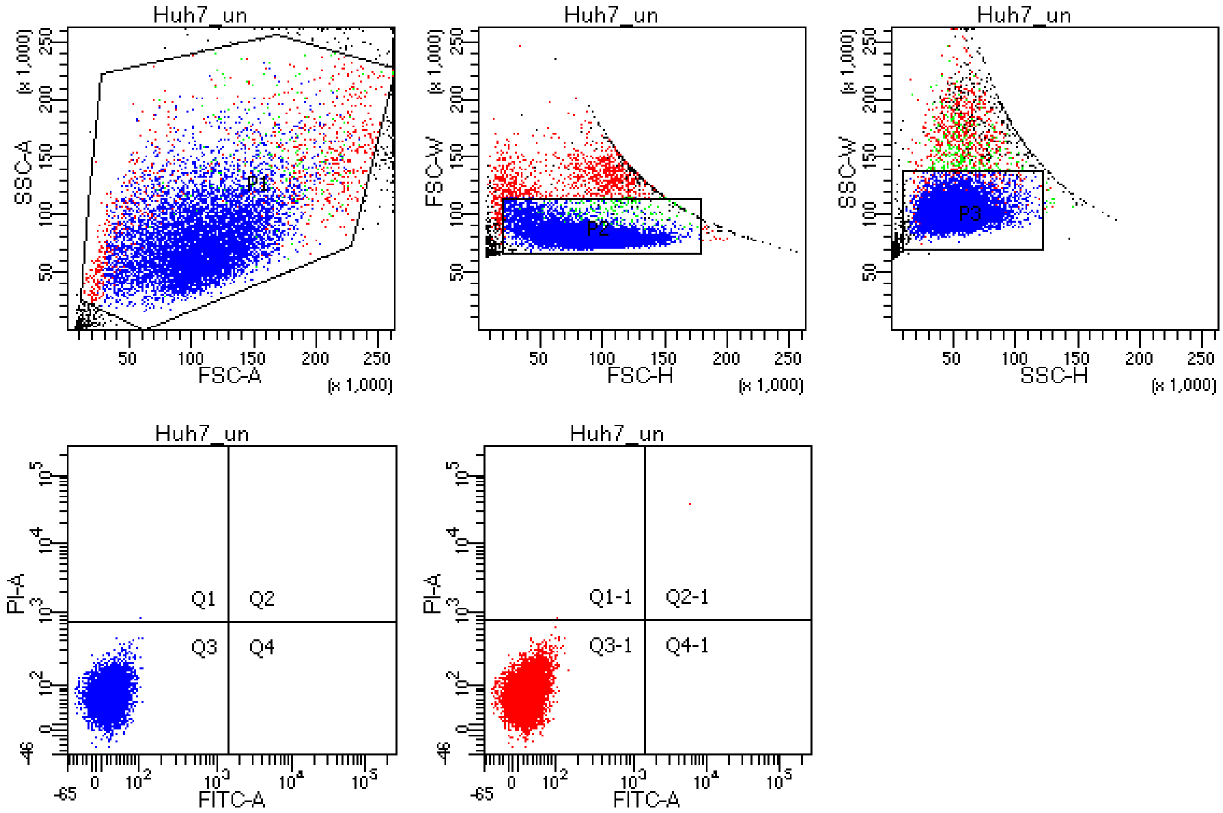

Tube: Huh7_un

Population	#Events	%Parent	%Total
All Events	10,000	####	100.0
P1	9,299	93.0	93.0
P2	8,270	88.9	82.7
P3	8,040	97.2	80.4
Q1	1	0.0	0.0
Q2	0	0.0	0.0
Q3	8,039	100.0	80.4
Q4	0	0.0	0.0
Q1-1	1	0.0	0.0
Q2-1	1	0.0	0.0
Q3-1	9,297	100.0	93.0
Q4-1	0	0.0	0.0

BD FACSDiva 8.0.2

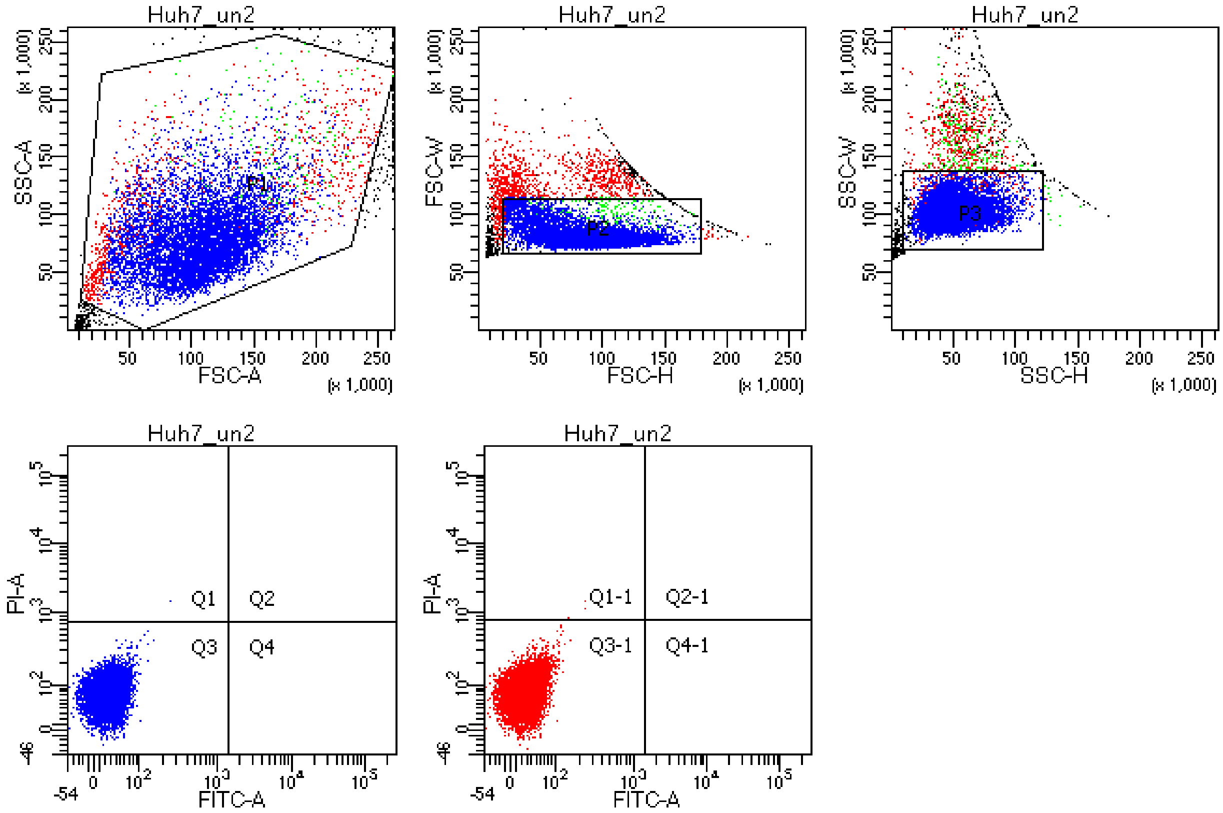

Tube: Huh7_un2

Population	#Events	%Parent	%Total
All Events	10,000	####	100.0
P1	9,434	94.3	94.3
P2	8,401	89.1	84.0
P3	8,209	97.7	82.1
Q1	1	0.0	0.0
Q2	0	0.0	0.0
Q3	8,208	100.0	82.1
Q4	0	0.0	0.0
Q1-1	3	0.0	0.0
Q2-1	0	0.0	0.0
Q3-1	9,431	100.0	94.3
Q4-1	0	0.0	0.0

BD FACSDiva 8.0.2

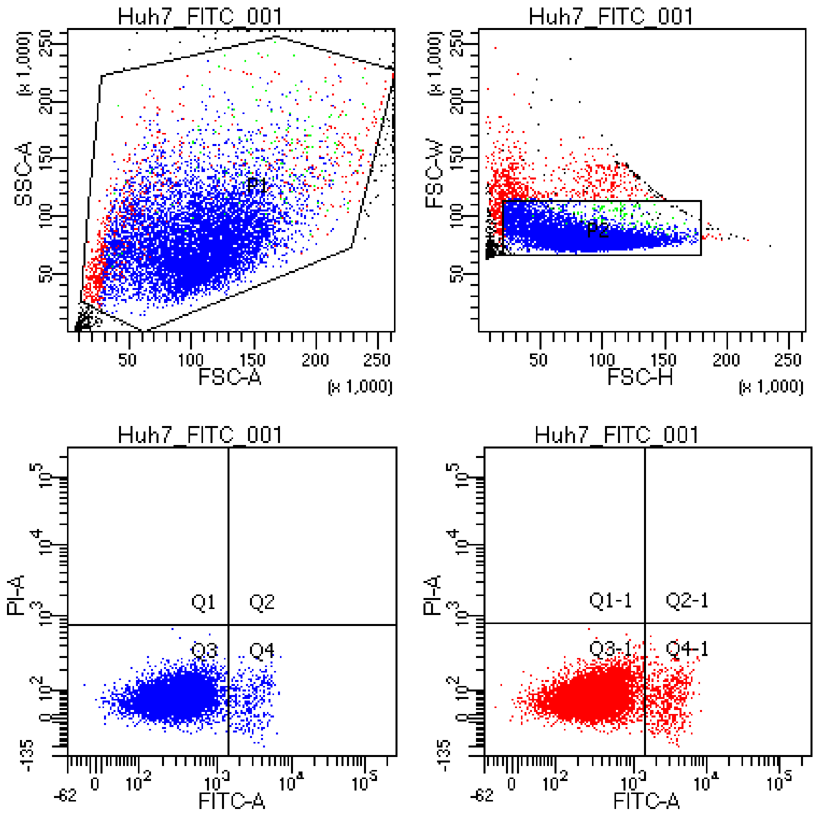

Tube: Huh7_FITC_001

Population	#Events	%Parent	%Total
All Events	10,000	####	100.0
P1	9,538	95.4	95.4
P2	8,640	90.6	86.4
P3	8,484	98.2	84.8
Q1	0	0.0	0.0
Q2	0	0.0	0.0
Q3	8,183	96.5	81.8
Q4	301	3.5	3.0
Q1-1	0	0.0	0.0
Q2-1	0	0.0	0.0
Q3-1	9,031	94.7	90.3
Q4-1	507	5.3	5.1

BD FACSDiva 8.0.2

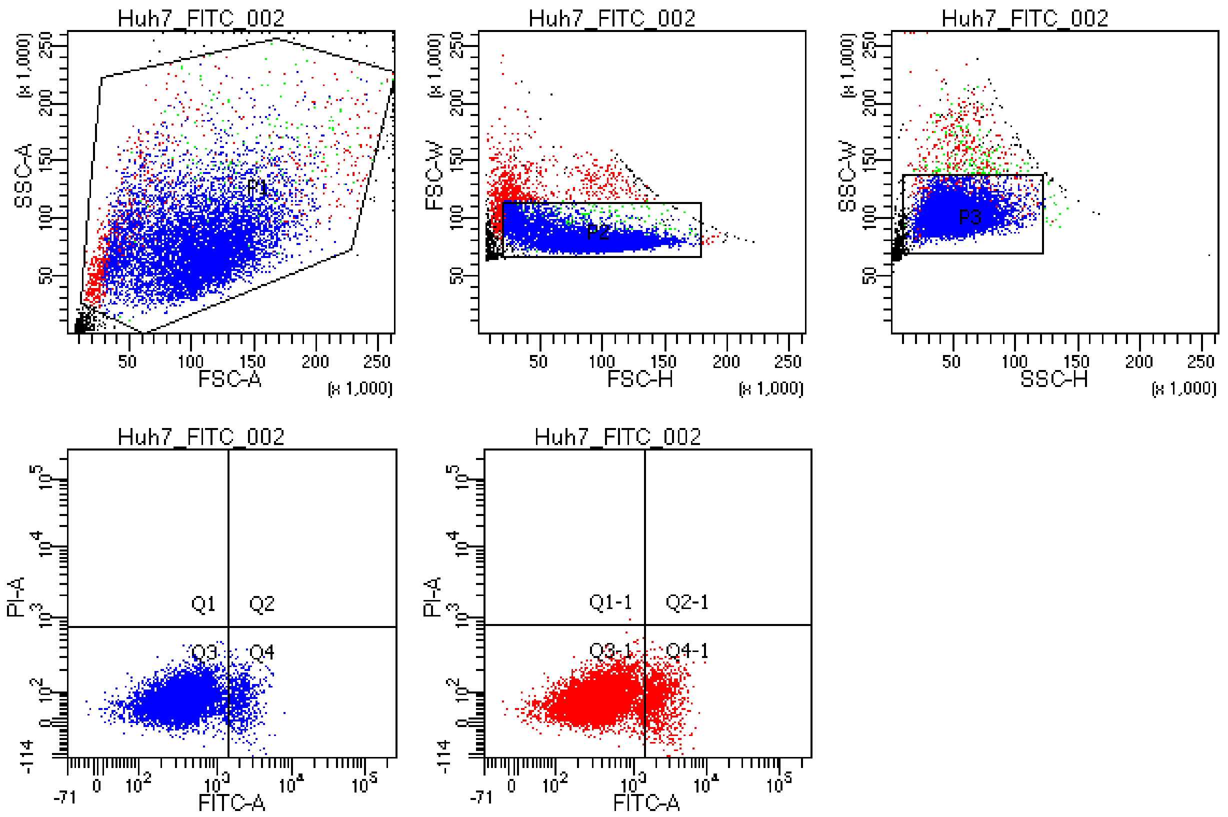

Tube: Huh7_FITC_002

Population	#Events	%Parent	%Total
All Events	10,000	####	100.0
P1	9,464	94.6	94.6
P2	8,495	89.8	85.0
P3	8,339	98.2	83.4
Q1	0	0.0	0.0
Q2	0	0.0	0.0
Q3	7,735	92.8	77.4
Q4	604	7.2	6.0
Q1-1	1	0.0	0.0
Q2-1	0	0.0	0.0
Q3-1	8,430	89.1	84.3
Q4-1	1,033	10.9	10.3

BD FACSDiva 8.0.2

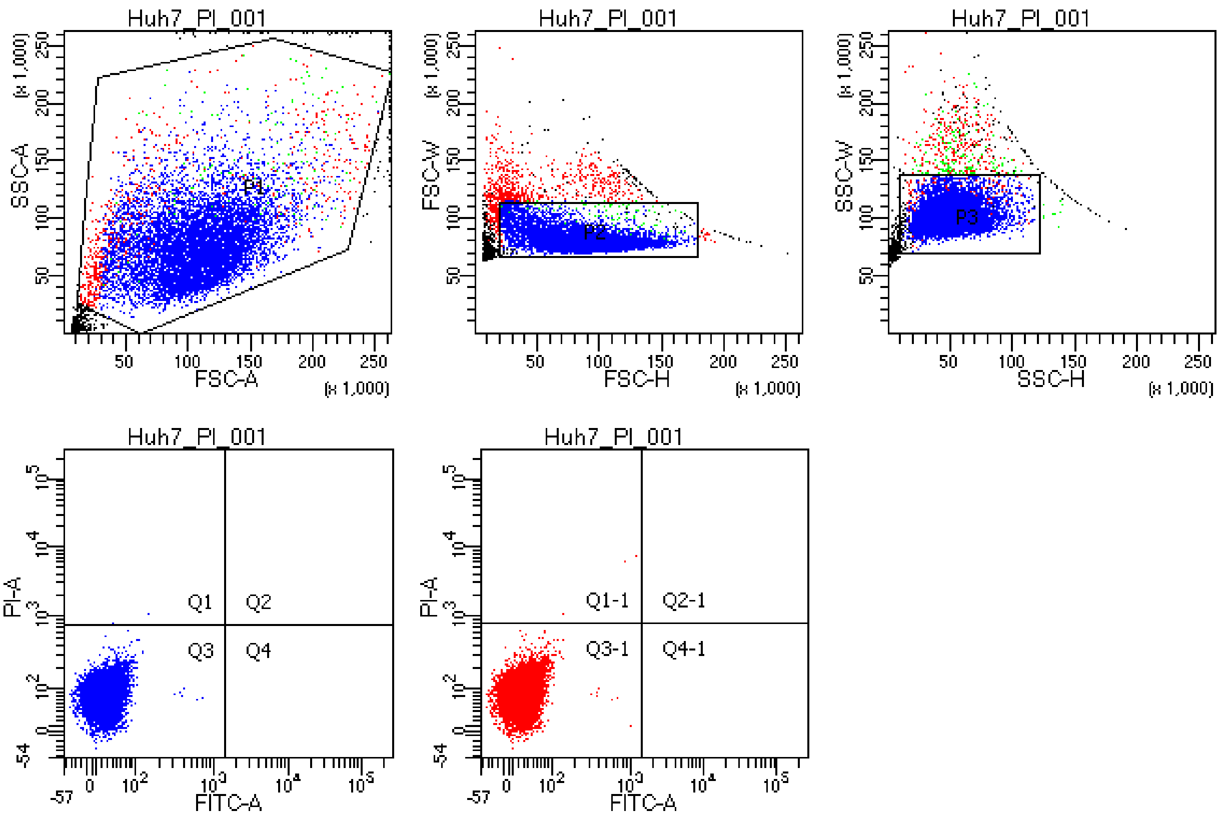

Tube: Huh7_PI_001

Population	#Events	%Parent	%Total
All Events	10,000	####	100.0
P1	9,462	94.6	94.6
P2	8,723	92.2	87.2
P3	8,570	98.2	85.7
Q1	2	0.0	0.0
Q2	0	0.0	0.0
Q3	8,568	100.0	85.7
Q4	0	0.0	0.0
Q1-1	4	0.0	0.0
Q2-1	0	0.0	0.0
Q3-1	9,458	100.0	94.6
Q4-1	0	0.0	0.0

BD FACSDiva 8.0.2

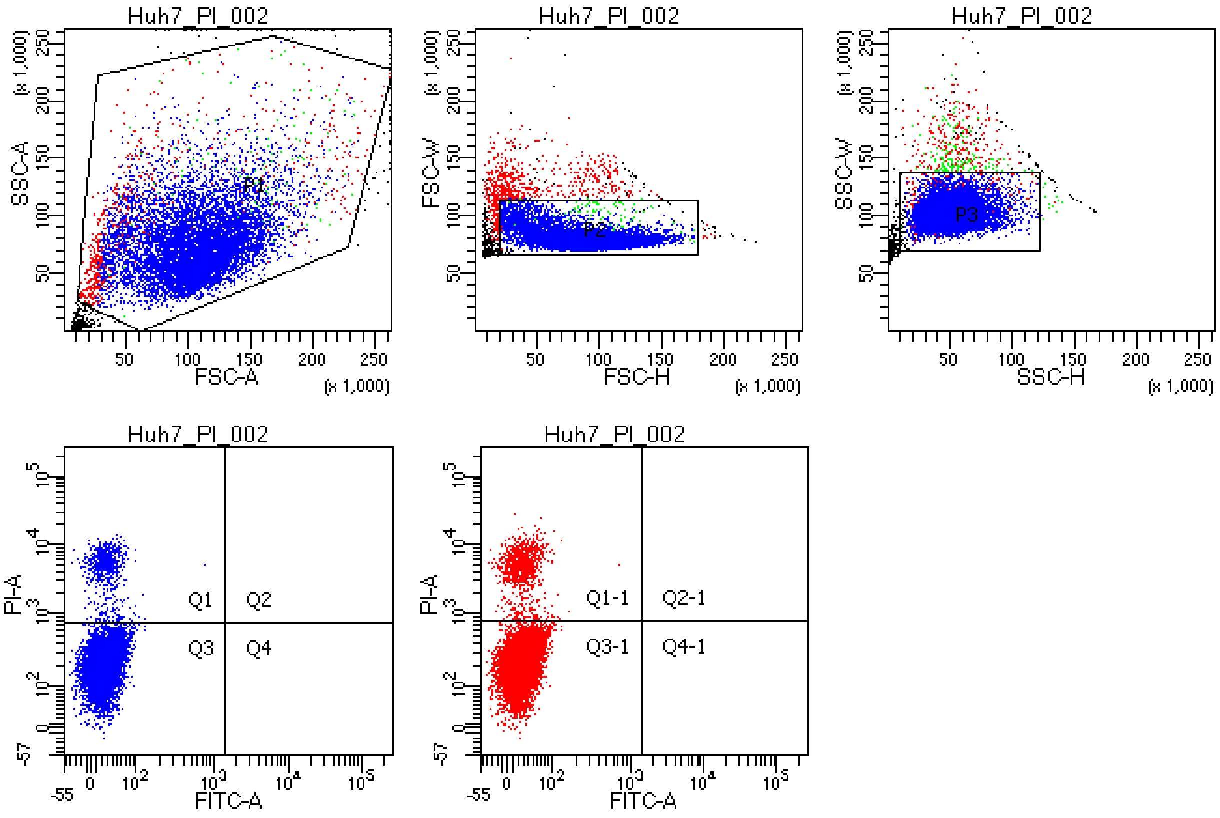

Tube: Huh7_PI_002

Population	#Events	%Parent	%Total
All Events	10,000	####	100.0
P1	9,576	95.8	95.8
P2	8,795	91.8	87.9
P3	8,646	98.3	86.5
Q1	689	8.0	6.9
Q2	0	0.0	0.0
Q3	7,957	92.0	79.6
Q4	0	0.0	0.0
Q1-1	1,202	12.6	12.0
Q2-1	0	0.0	0.0
Q3-1	8,374	87.4	83.7
Q4-1	0	0.0	0.0

BD FACSDiva 8.0.2

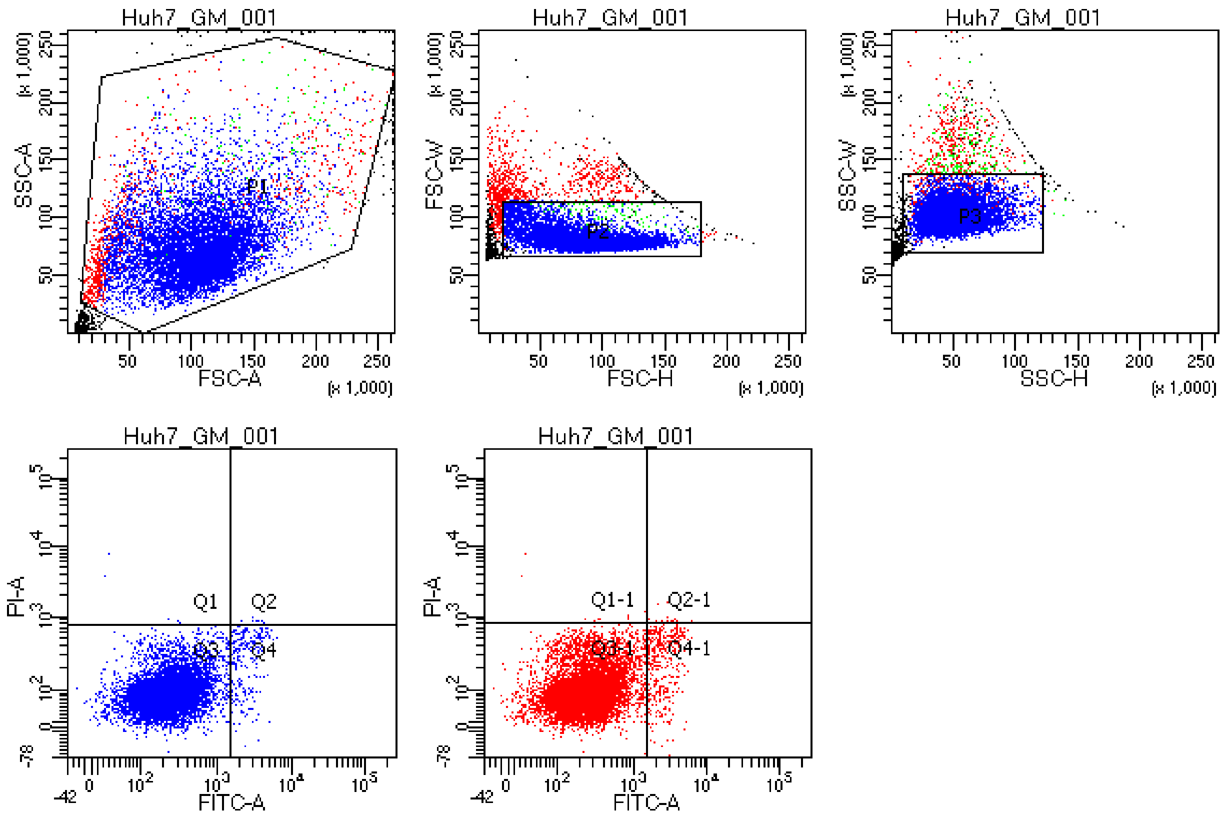

Tube: Huh7_GM_001

Population	#Events	%Parent	%Total
All Events	10,000	####	100.0
P1	9,463	94.6	94.6
P2	8,508	89.9	85.1
P3	8,359	98.2	83.6
Q1	5	0.1	0.0
Q2	7	0.1	0.1
Q3	8,133	97.3	81.3
Q4	214	2.6	2.1
Q1-1	15	0.2	0.2
Q2-1	15	0.2	0.2
Q3-1	9,077	95.9	90.8
Q4-1	356	3.8	3.6

BD FACSDiva 8.0.2

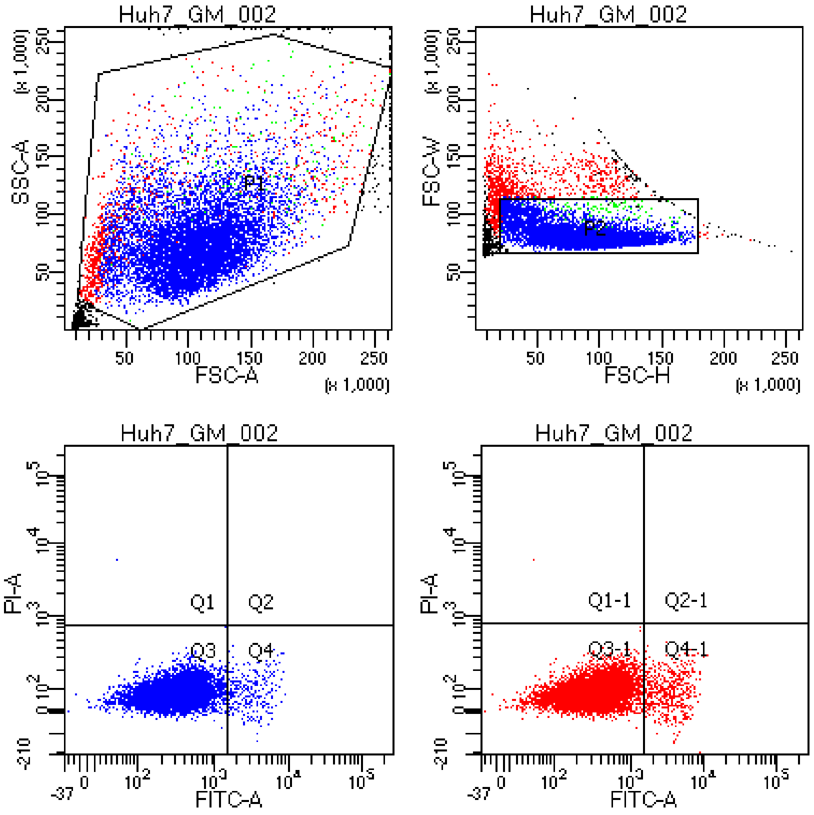

Tube: Huh7_GM_002

Population	#Events	%Parent	%Total
All Events	10,000	####	100.0
P1	9,485	94.8	94.8
P2	8,473	89.3	84.7
P3	8,296	97.9	83.0
Q1	1	0.0	0.0
Q2	0	0.0	0.0
Q3	8,044	97.0	80.4
Q4	251	3.0	2.5
Q1-1	1	0.0	0.0
Q2-1	0	0.0	0.0
Q3-1	9,023	95.1	90.2
Q4-1	461	4.9	4.6

BD FACSDiva 8.0.2

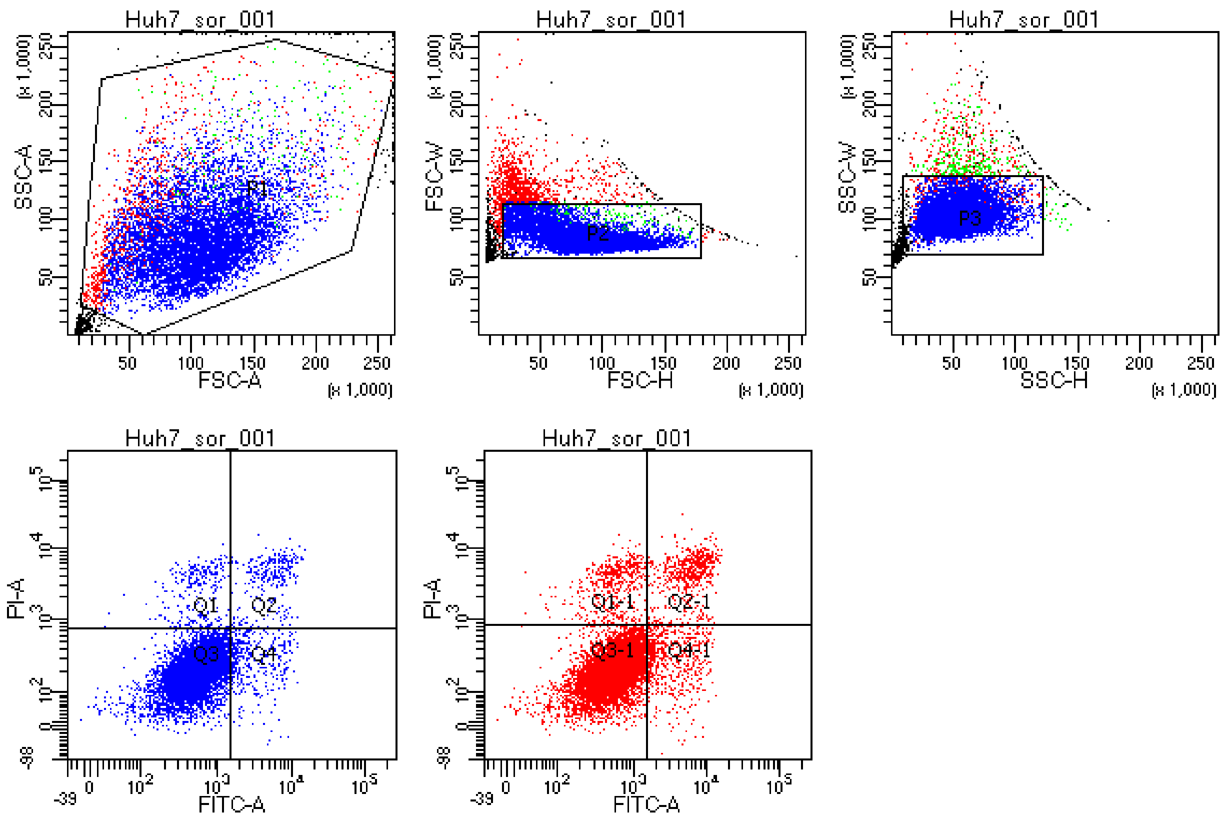

Tube: Huh7_sor_001

Population	#Events	%Parent	%Total
All Events	10,000	####	100.0
P1	9,427	94.3	94.3
P2	8,320	88.3	83.2
P3	8,079	97.1	80.8
Q1	262	3.2	2.6
Q2	280	3.5	2.8
Q3	7,137	88.3	71.4
Q4	400	5.0	4.0
Q1-1	557	5.9	5.6
Q2-1	624	6.6	6.2
Q3-1	7,685	81.5	76.8
Q4-1	561	6.0	5.6

BD FACSDiva 8.0.2

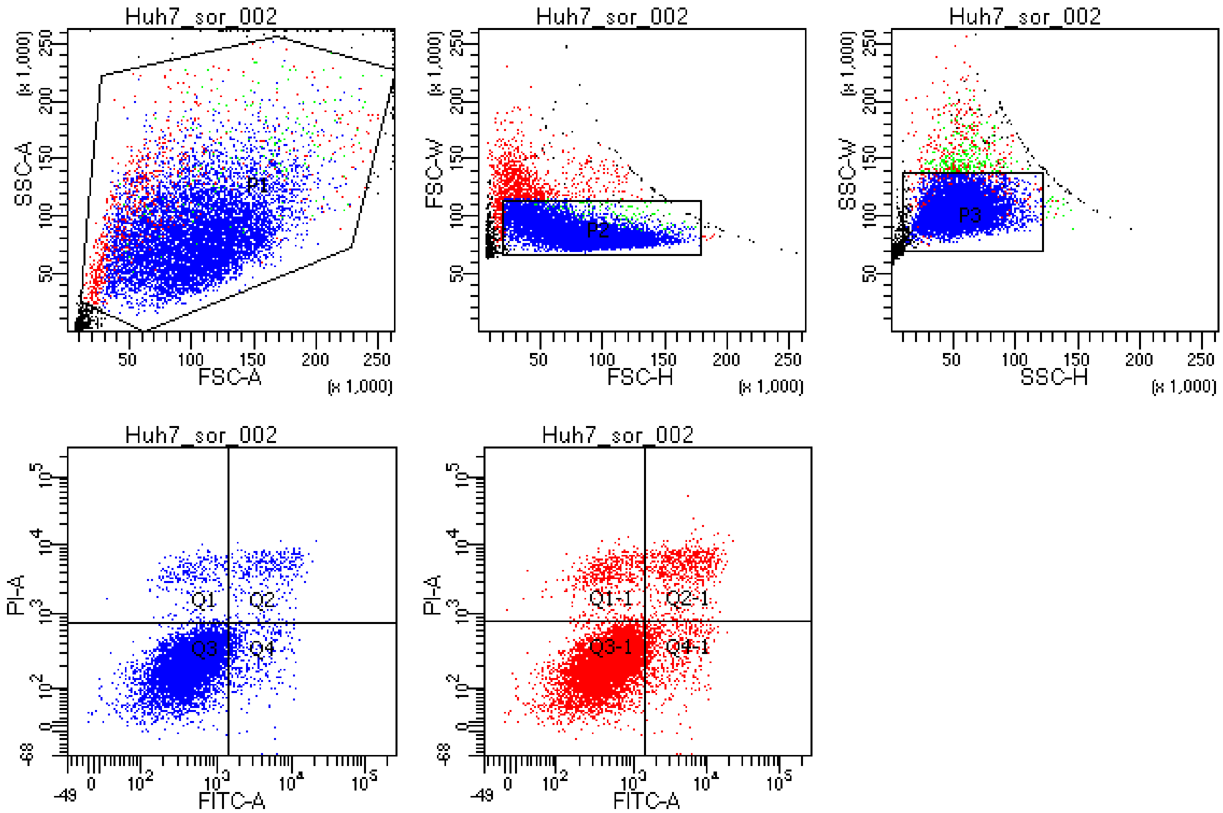

Tube: Huh7_sor_002

Population	#Events	%Parent	%Total
All Events	10,000	####	100.0
P1	9,479	94.8	94.8
P2	8,387	88.5	83.9
P3	8,165	97.4	81.6
Q1	274	3.4	2.7
Q2	327	4.0	3.3
Q3	7,224	88.5	72.2
Q4	340	4.2	3.4
Q1-1	571	6.0	5.7
Q2-1	654	6.9	6.5
Q3-1	7,774	82.0	77.7
Q4-1	480	5.1	4.8

BD FACSDiva 8.0.2

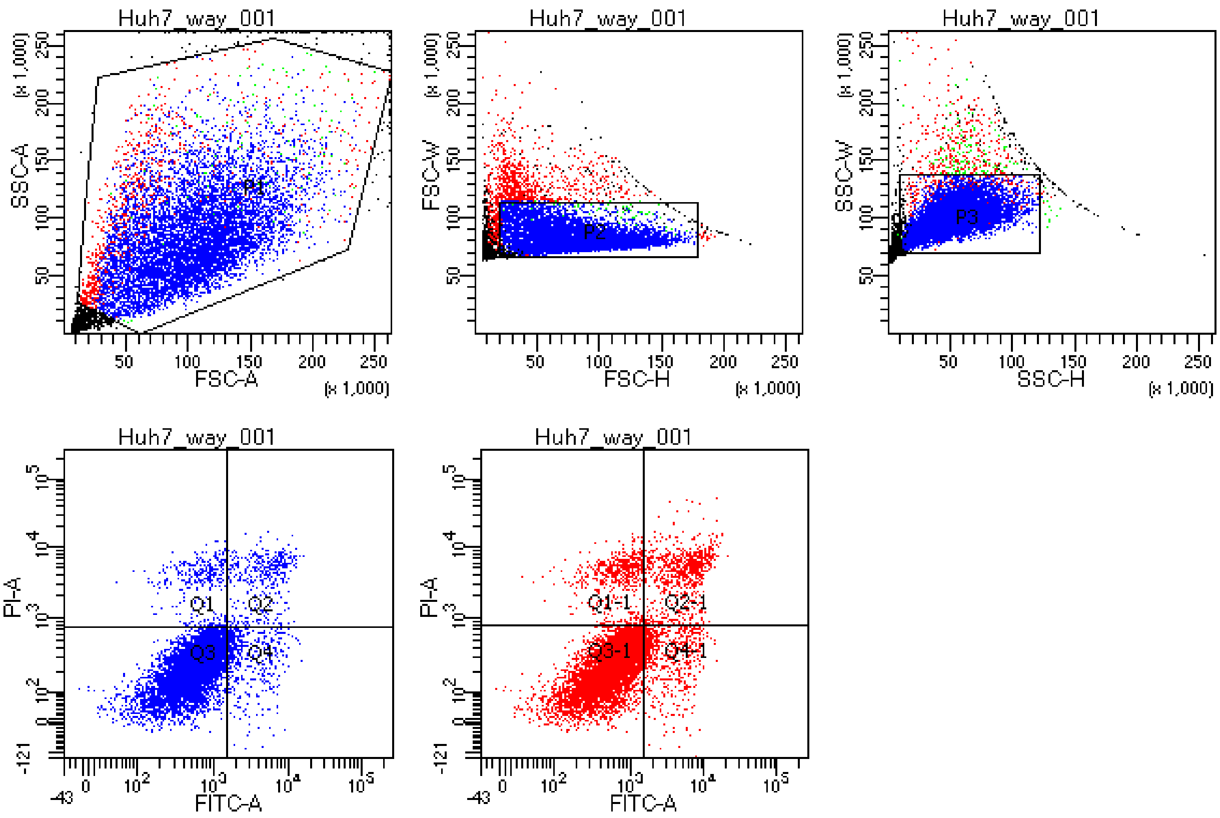

Tube: Huh7_way_001

Population	#Events	%Parent	%Total
All Events	10,000	####	100.0
P1	8,856	88.6	88.6
P2	7,836	88.5	78.4
P3	7,707	98.4	77.1
Q1	354	4.6	3.5
Q2	393	5.1	3.9
Q3	6,440	83.6	64.4
Q4	520	6.7	5.2
Q1-1	634	7.2	6.3
Q2-1	805	9.1	8.0
Q3-1	6,794	76.7	67.9
Q4-1	623	7.0	6.2

BD FACSDiva 8.0.2

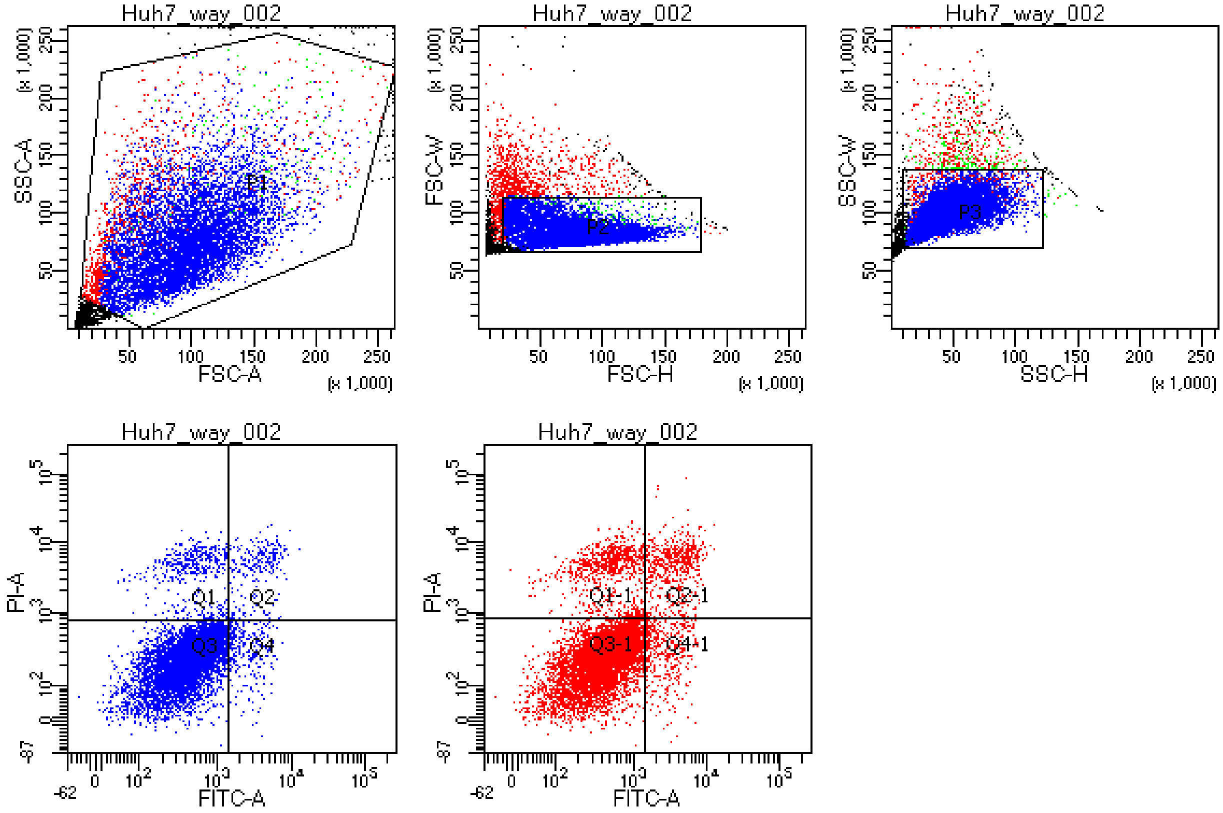

Tube: Huh7_way_002

Population	#Events	%Parent	%Total
All Events	10,000	####	100.0
P1	9,029	90.3	90.3
P2	7,839	86.8	78.4
P3	7,693	98.1	76.9
Q1	482	6.3	4.8
Q2	267	3.5	2.7
Q3	6,702	87.1	67.0
Q4	242	3.1	2.4
Q1-1	953	10.6	9.5
Q2-1	636	7.0	6.4
Q3-1	7,086	78.5	70.9
Q4-1	354	3.9	3.5

BD FACSDiva 8.0.2

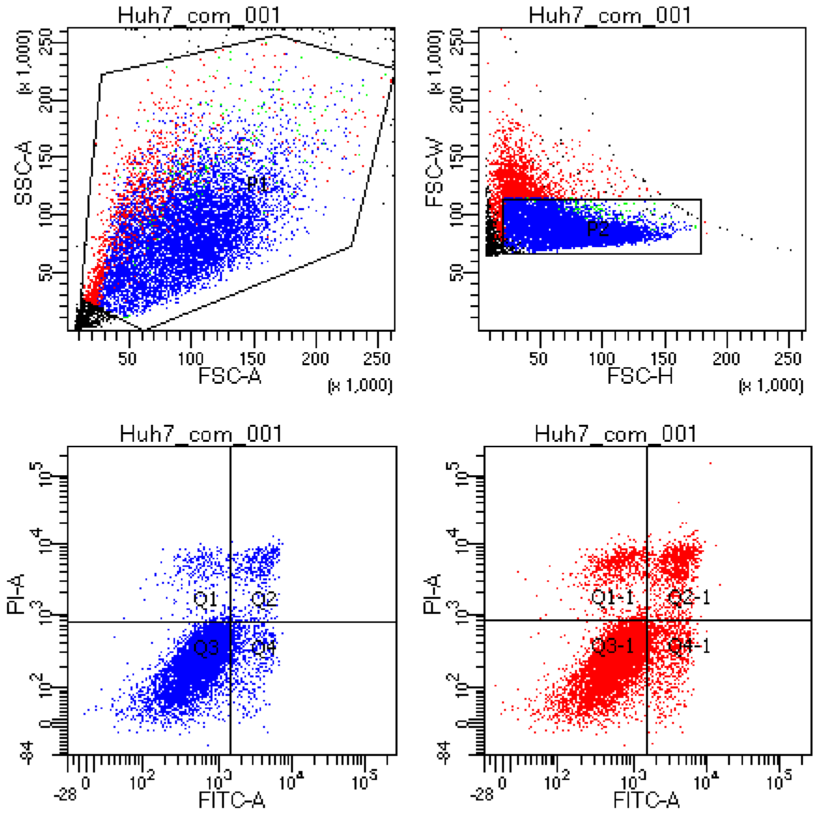

Tube: Huh7_com_001

Population	#Events	%Parent	%Total
All Events	10,000	####	100.0
P1	9,095	91.0	91.0
P2	7,482	82.3	74.8
P3	7,257	97.0	72.6
Q1	313	4.3	3.1
Q2	375	5.2	3.8
Q3	6,090	83.9	60.9
Q4	479	6.6	4.8
Q1-1	730	8.0	7.3
Q2-1	877	9.6	8.8
Q3-1	6,744	74.2	67.4
Q4-1	744	8.2	7.4

BD FACSDiva 8.0.2

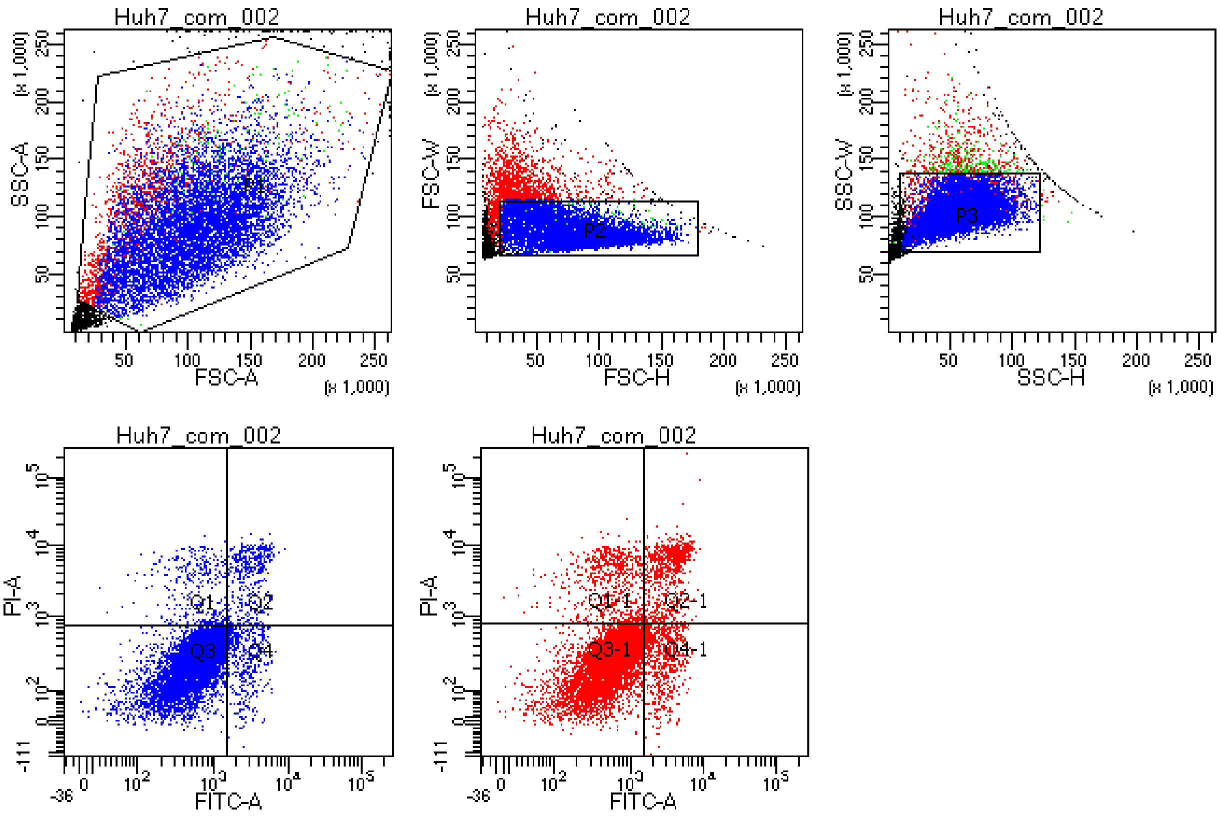

Tube: Huh7_com_002

Population	#Events	%Parent	%Total
All Events	10,426	####	100.0
P1	9,430	90.4	90.4
P2	7,935	84.1	76.1
P3	7,732	97.4	74.2
Q1	375	4.8	3.6
Q2	436	5.6	4.2
Q3	6,446	83.4	61.8
Q4	475	6.1	4.6
Q1-1	789	8.4	7.6
Q2-1	849	9.0	8.1
Q3-1	7,097	75.3	68.1
Q4-1	695	7.4	6.7

BD FACSDiva 8.0.2

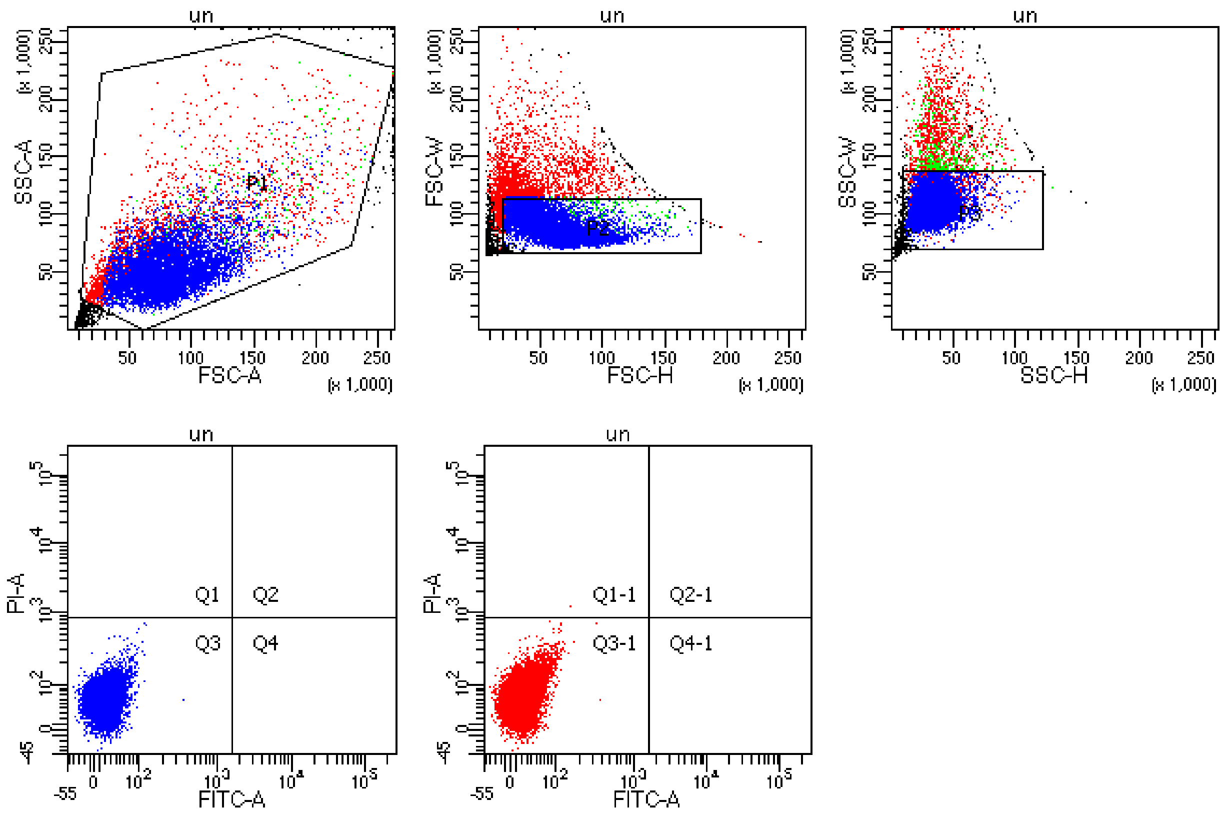

Tube: un

Population	#Events	%Parent	%Total
All Events	10,000	####	100.0
P1	9,319	93.2	93.2
P2	7,074	75.9	70.7
P3	6,779	95.8	67.8
Q1	0	0.0	0.0
Q2	0	0.0	0.0
Q3	6,779	100.0	67.8
Q4	0	0.0	0.0
Q1-1	1	0.0	0.0
Q2-1	0	0.0	0.0
Q3-1	9,318	100.0	93.2
Q4-1	0	0.0	0.0

BD FACSDiva 8.0.2

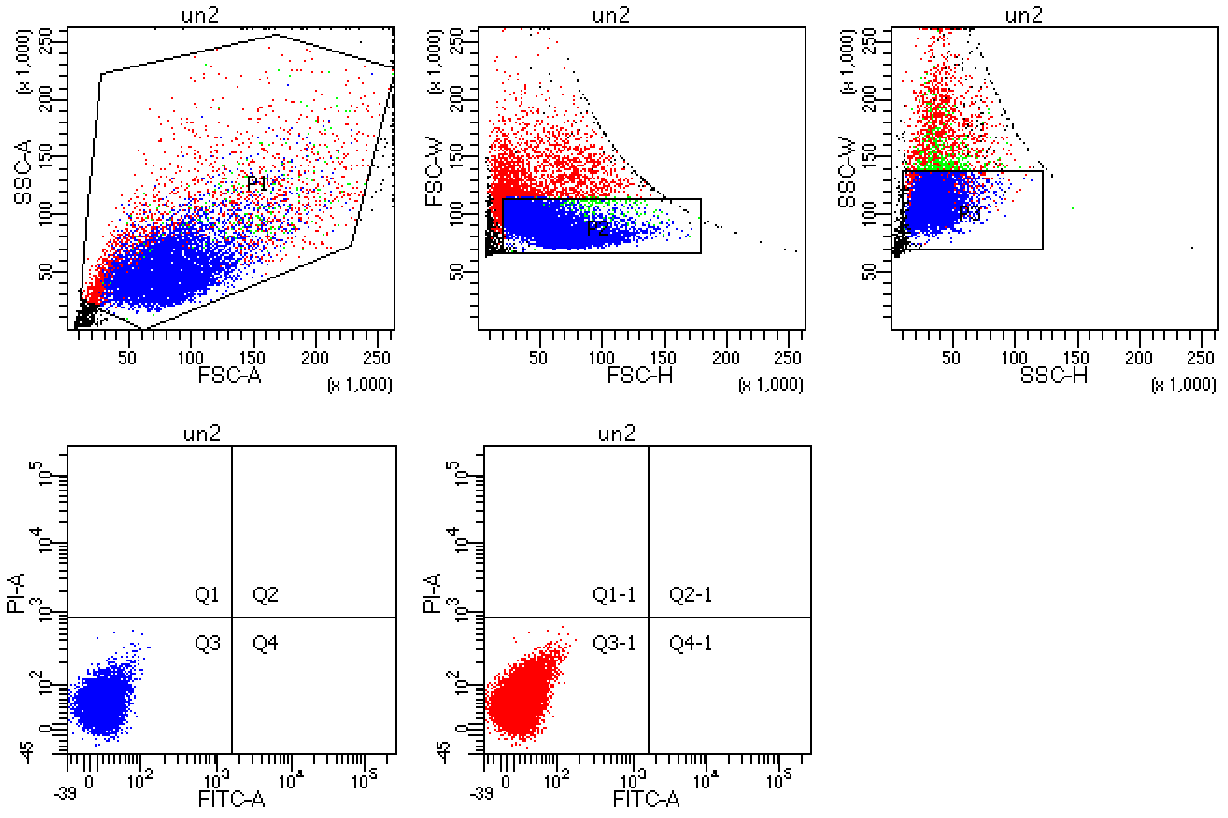

Tube: un2

Population	#Events	%Parent	%Total
All Events	10,000	####	100.0
P1	9,355	93.6	93.6
P2	6,902	73.8	69.0
P3	6,585	95.4	65.8
Q1	0	0.0	0.0
Q2	0	0.0	0.0
Q3	6,585	100.0	65.8
Q4	0	0.0	0.0
Q1-1	0	0.0	0.0
Q2-1	0	0.0	0.0
Q3-1	9,355	100.0	93.6
Q4-1	0	0.0	0.0

BD FACSDiva 8.0.2

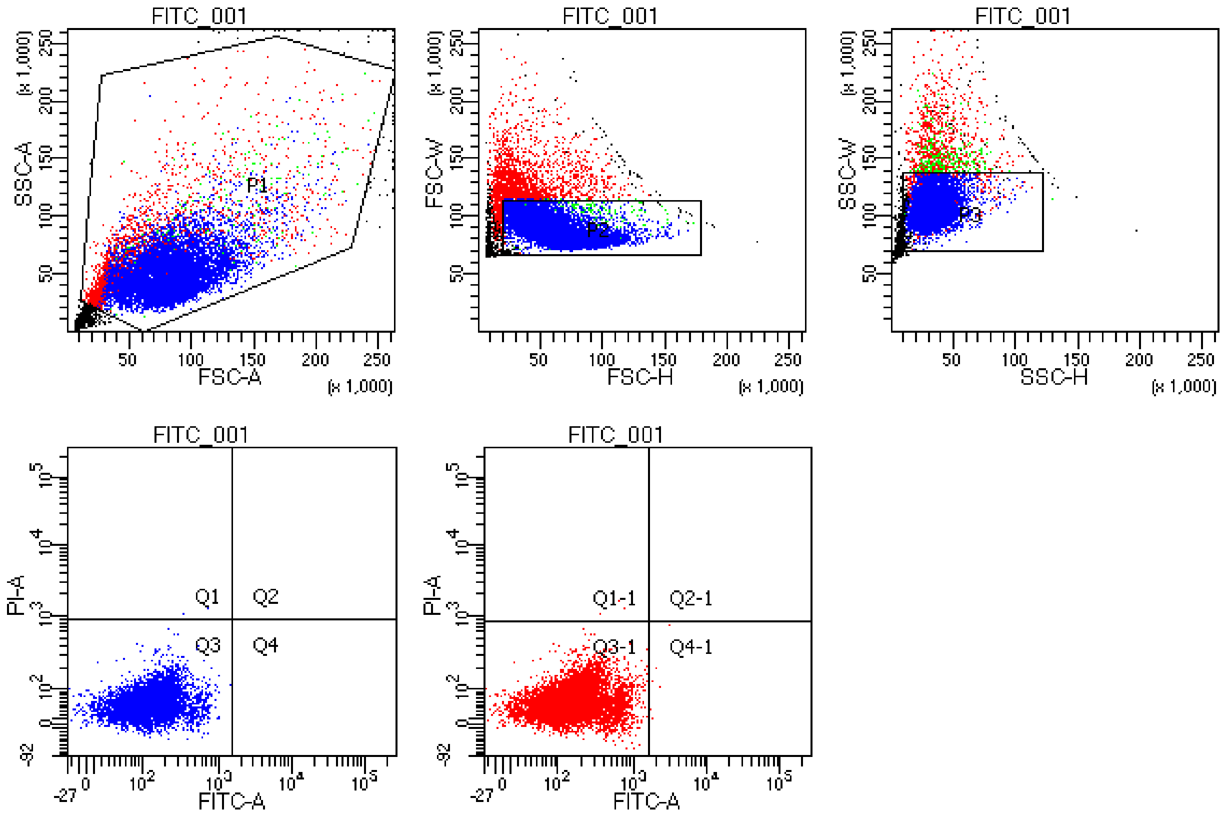

Tube: FITC_001

Population	#Events	%Parent	%Total
All Events	10,000	####	100.0
P1	9,320	93.2	93.2
P2	7,292	78.2	72.9
P3	7,053	96.7	70.5
Q1	2	0.0	0.0
Q2	0	0.0	0.0
Q3	7,051	100.0	70.5
Q4	0	0.0	0.0
Q1-1	3	0.0	0.0
Q2-1	0	0.0	0.0
Q3-1	9,311	99.9	93.1
Q4-1	6	0.1	0.1

BD FACSDiva 8.0.2

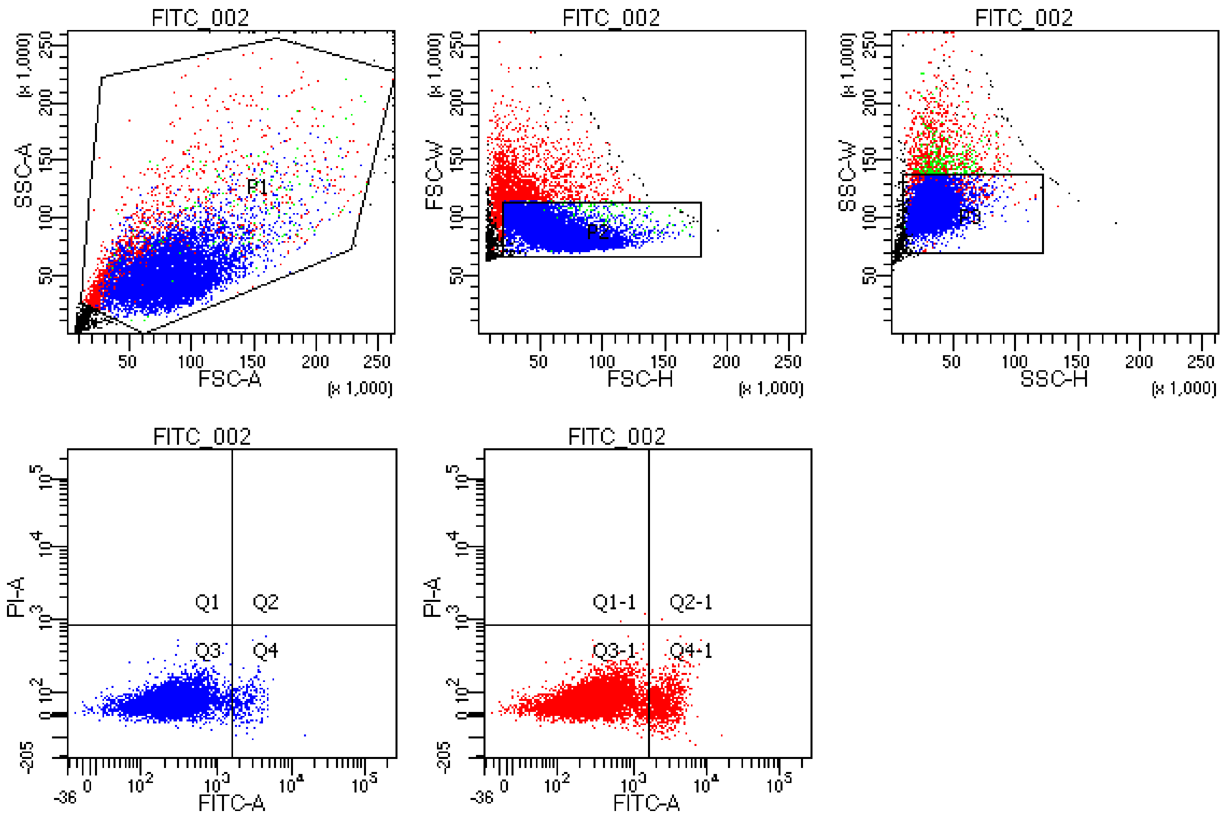

Tube: FITC_002

Population	#Events	%Parent	%Total
All Events	10,000	####	100.0
P1	9,330	93.3	93.3
P2	7,307	78.3	73.1
P3	7,073	96.8	70.7
Q1	0	0.0	0.0
Q2	0	0.0	0.0
Q3	6,817	96.4	68.2
Q4	256	3.6	2.6
Q1-1	2	0.0	0.0
Q2-1	1	0.0	0.0
Q3-1	8,430	90.4	84.3
Q4-1	897	9.6	9.0

BD FACSDiva 8.0.2

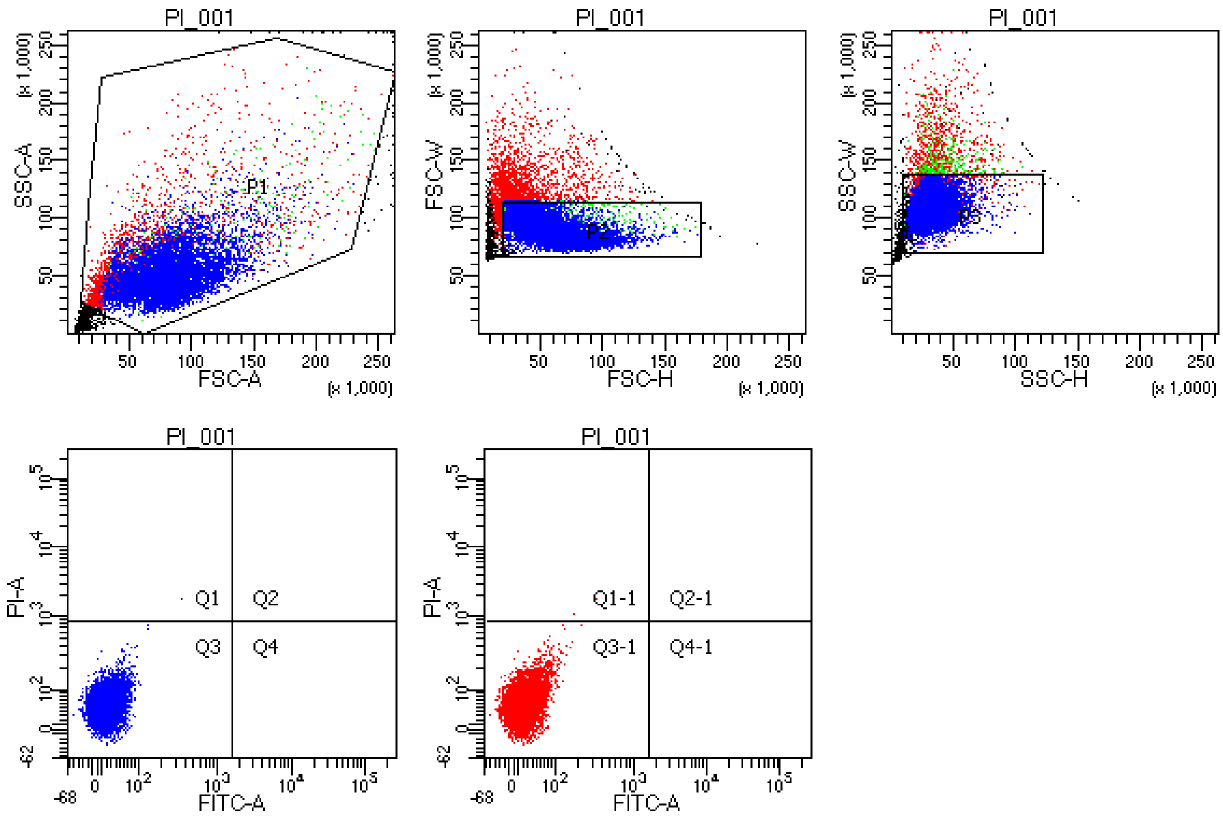

Tube: PI_001

Population	#Events	%Parent	%Total
All Events	10,000	####	100.0
P1	9,272	92.7	92.7
P2	7,298	78.7	73.0
P3	7,056	96.7	70.6
Q1	1	0.0	0.0
Q2	0	0.0	0.0
Q3	7,055	100.0	70.6
Q4	0	0.0	0.0
Q1-1	2	0.0	0.0
Q2-1	0	0.0	0.0
Q3-1	9,270	100.0	92.7
Q4-1	0	0.0	0.0

BD FACSDiva 8.0.2

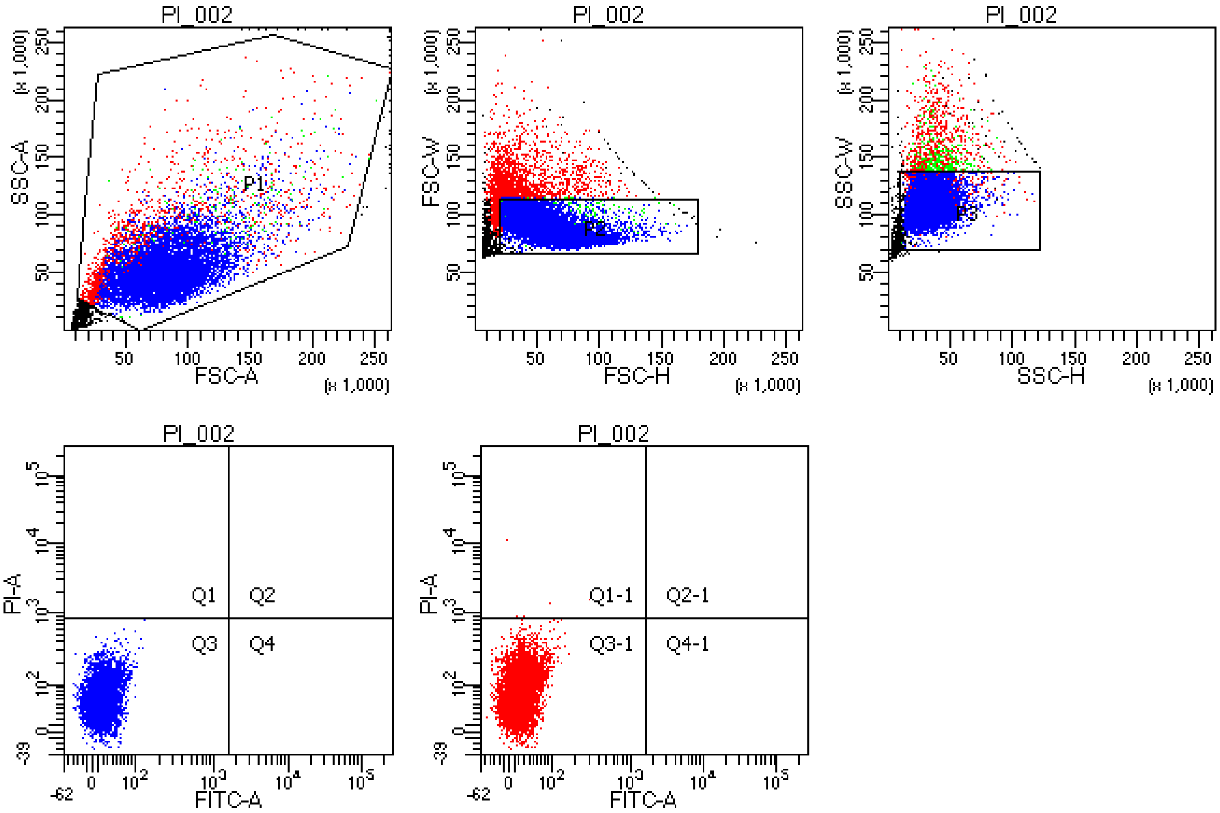

Tube: PI_002

Population	#Events	%Parent	%Total
All Events	10,000	####	100.0
P1	9,268	92.7	92.7
P2	7,354	79.3	73.5
P3	7,106	96.6	71.1
Q1	0	0.0	0.0
Q2	0	0.0	0.0
Q3	7,106	100.0	71.1
Q4	0	0.0	0.0
Q1-1	5	0.1	0.0
Q2-1	0	0.0	0.0
Q3-1	9,263	99.9	92.6
Q4-1	0	0.0	0.0

BD FACSDiva 8.0.2

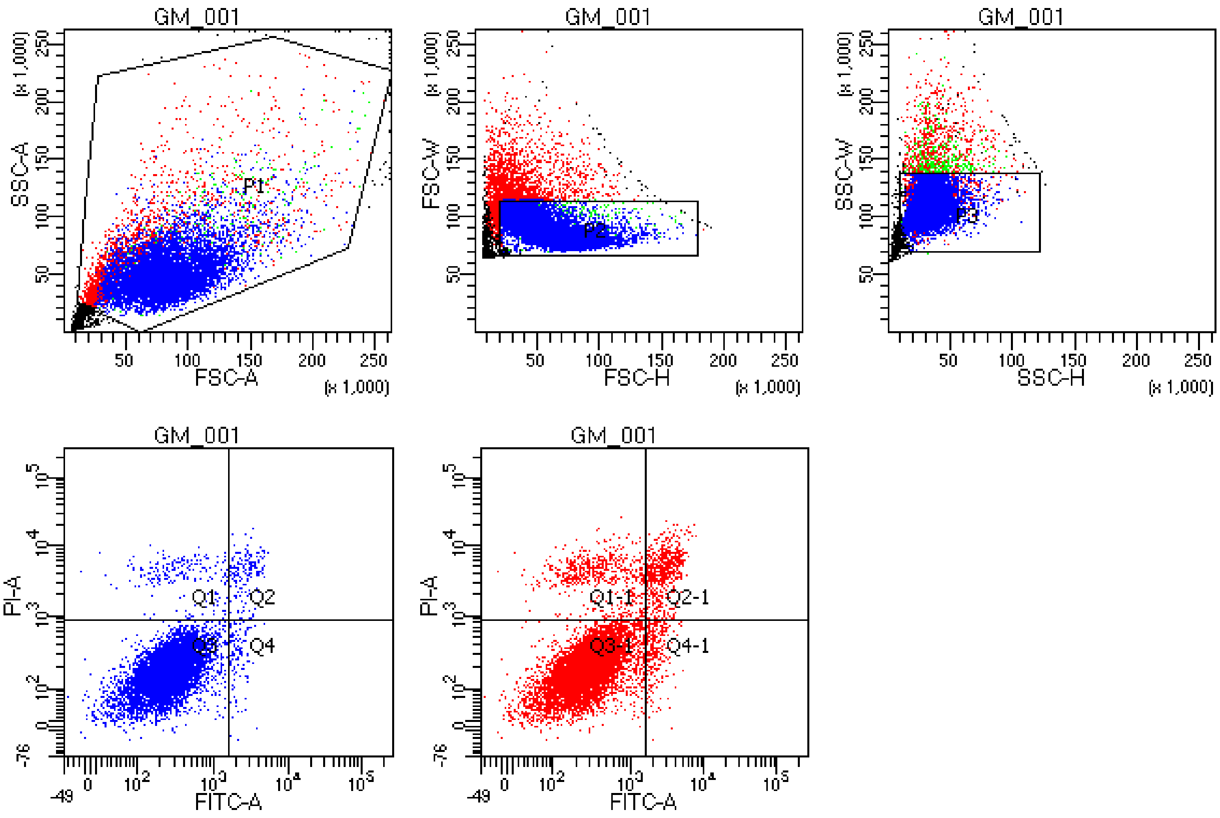

Tube: GM_001

Population	#Events	%Parent	%Total
All Events	10,000	####	100.0
P1	9,314	93.1	93.1
P2	7,329	78.7	73.3
P3	7,076	96.5	70.8
Q1	216	3.1	2.2
Q2	175	2.5	1.8
Q3	6,572	92.9	65.7
Q4	113	1.6	1.1
Q1-1	643	6.9	6.4
Q2-1	680	7.3	6.8
Q3-1	7,738	83.1	77.4
Q4-1	253	2.7	2.5

BD FACSDiva 8.0.2

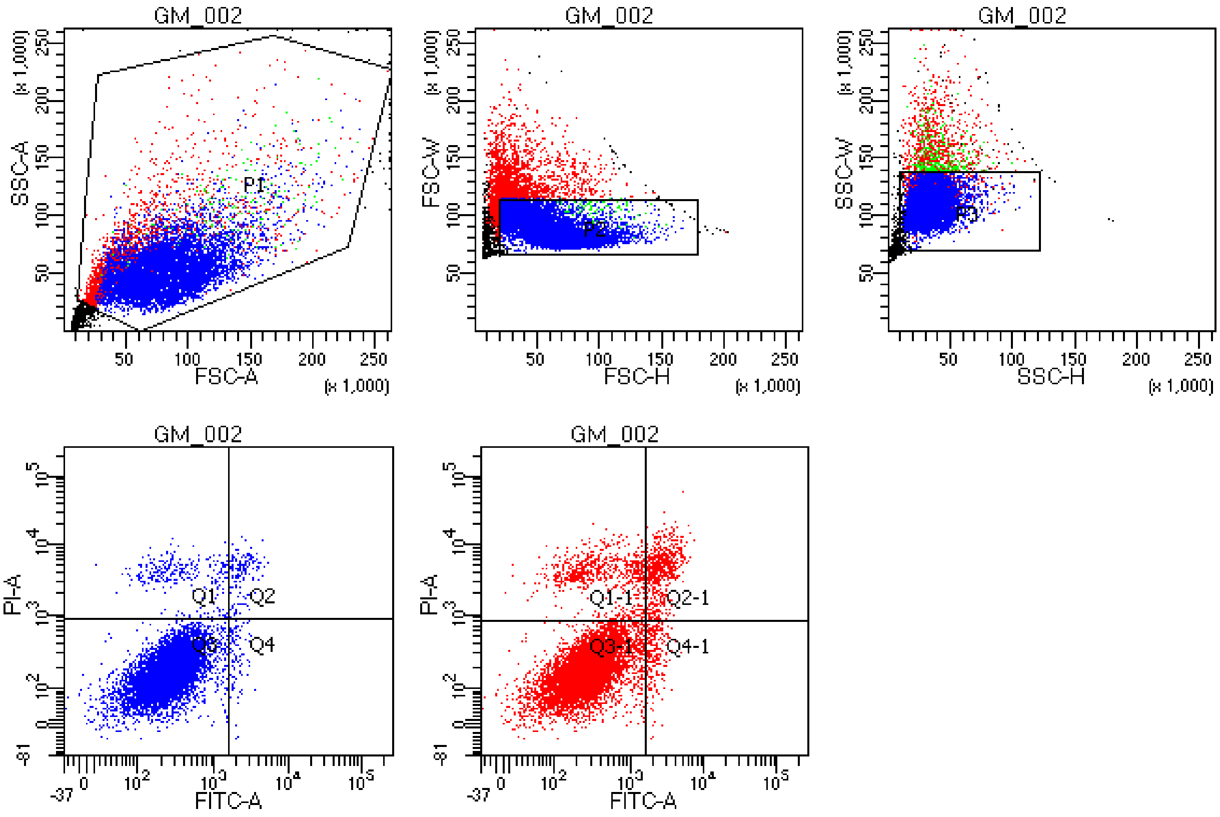

Tube: GM_002

Population	#Events	%Parent	%Total
All Events	10,000	####	100.0
P1	9,328	93.3	93.3
P2	7,293	78.2	72.9
P3	7,043	96.6	70.4
Q1	281	4.0	2.8
Q2	145	2.1	1.5
Q3	6,521	92.6	65.2
Q4	96	1.4	1.0
Q1-1	742	8.0	7.4
Q2-1	619	6.6	6.2
Q3-1	7,737	82.9	77.4
Q4-1	230	2.5	2.3

BD FACSDiva 8.0.2

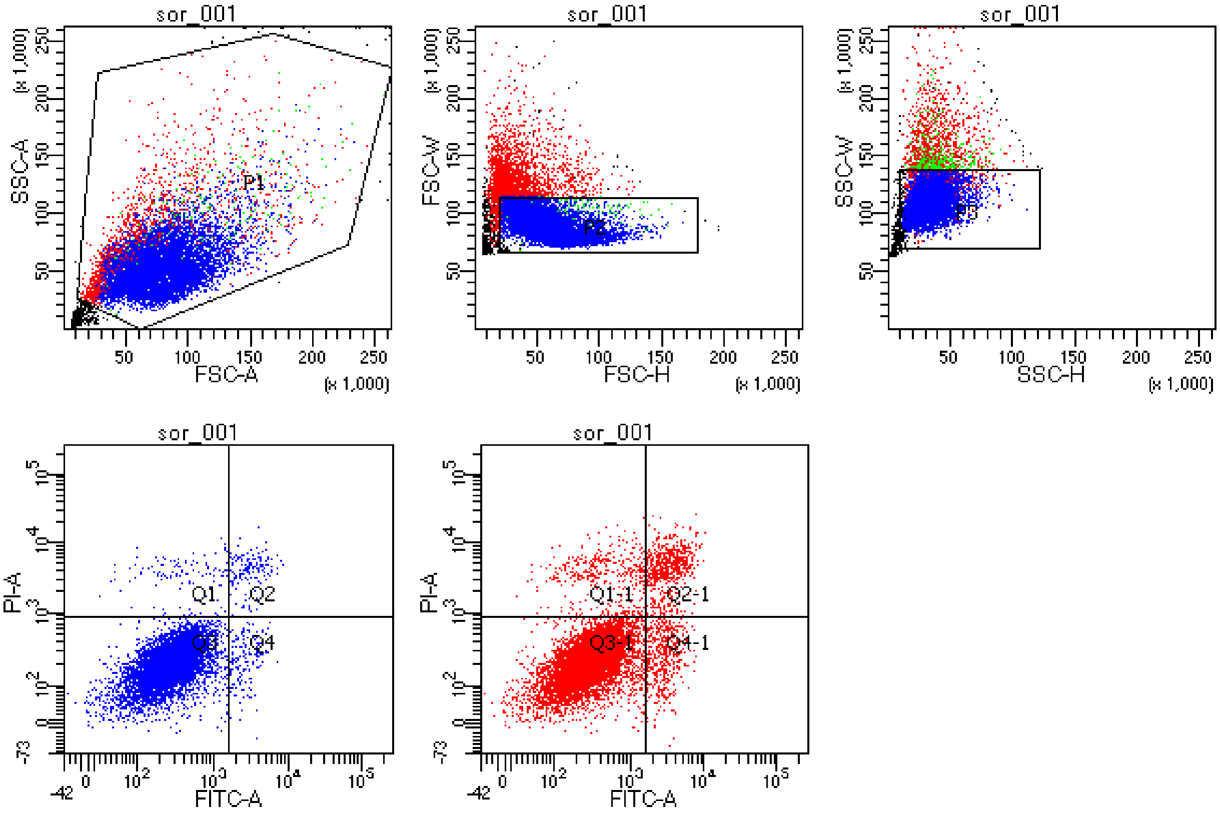

Tube: sor_001

Population	#Events	%Parent	%Total
All Events	10,000	####	100.0
P1	9,461	94.6	94.6
P2	7,147	75.5	71.5
P3	6,891	96.4	68.9
Q1	123	1.8	1.2
Q2	155	2.2	1.6
Q3	6,454	93.7	64.5
Q4	159	2.3	1.6
Q1-1	442	4.7	4.4
Q2-1	657	6.9	6.6
Q3-1	7,861	83.1	78.6
Q4-1	501	5.3	5.0

BD FACSDiva 8.0.2

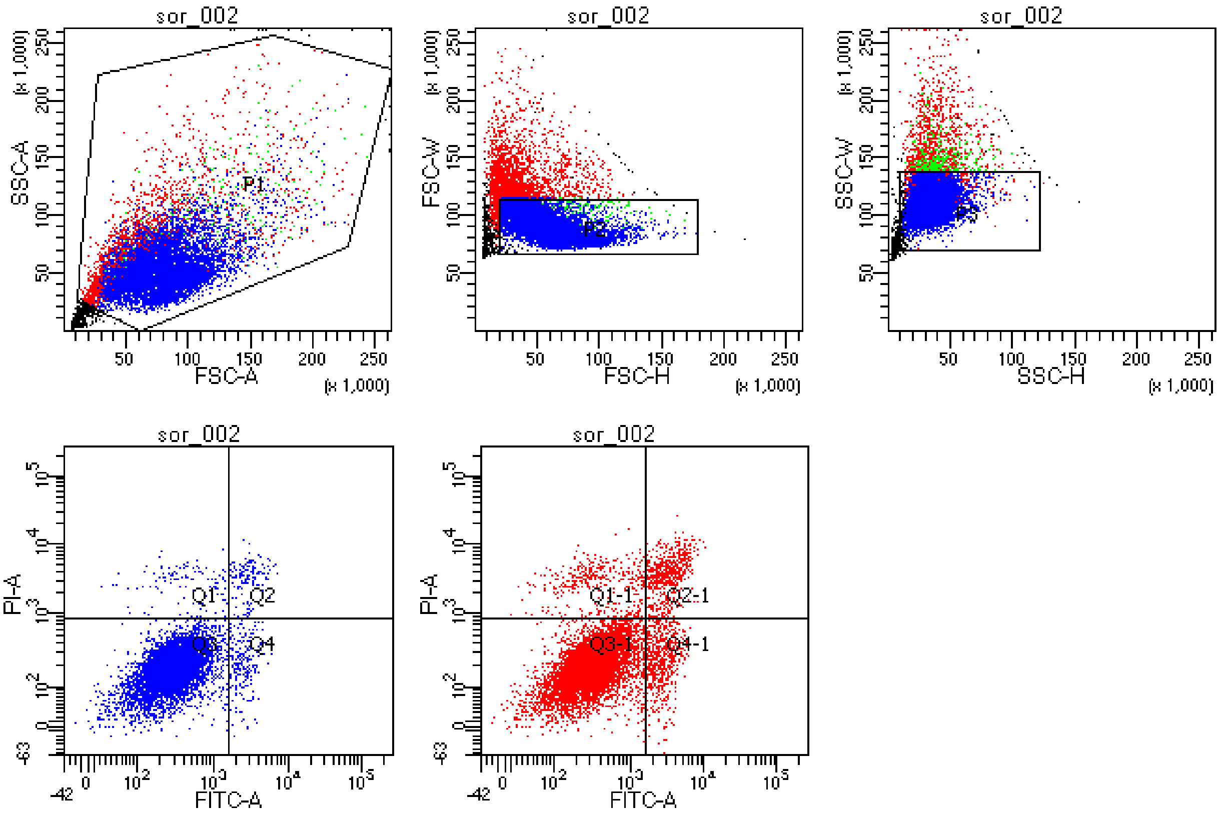

Tube: sor_002

Population	#Events	%Parent	%Total
All Events	10,000	####	100.0
P1	9,450	94.5	94.5
P2	7,006	74.1	70.1
P3	6,726	96.0	67.3
Q1	115	1.7	1.2
Q2	143	2.1	1.4
Q3	6,249	92.9	62.5
Q4	219	3.3	2.2
Q1-1	409	4.3	4.1
Q2-1	634	6.7	6.3
Q3-1	7,792	82.5	77.9
Q4-1	615	6.5	6.2

BD FACSDiva 8.0.2

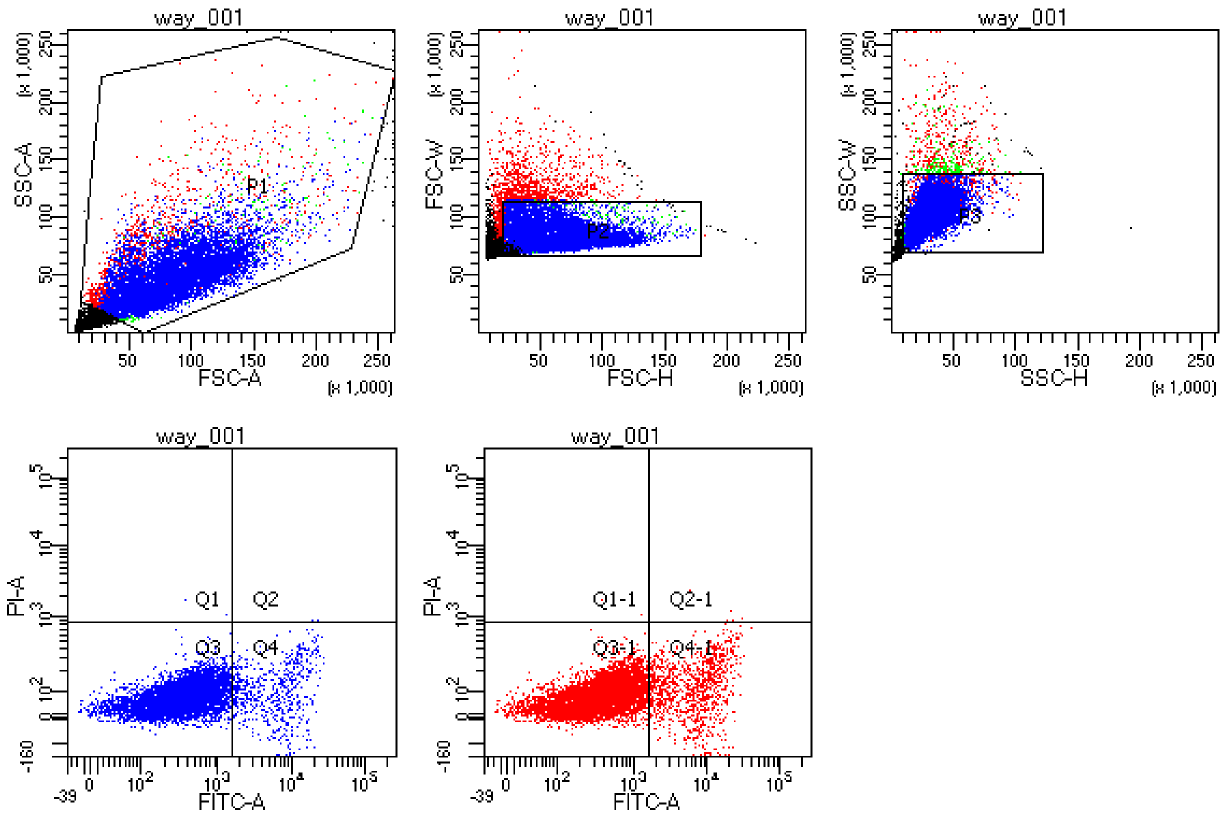

Tube: way_001

Population	#Events	%Parent	%Total
All Events	10,000	####	100.0
P1	8,630	86.3	86.3
P2	7,542	87.4	75.4
P3	7,354	97.5	73.5
Q1	2	0.0	0.0
Q2	2	0.0	0.0
Q3	6,704	91.2	67.0
Q4	646	8.8	6.5
Q1-1	2	0.0	0.0
Q2-1	5	0.1	0.0
Q3-1	7,467	86.5	74.7
Q4-1	1,156	13.4	11.6

BD FACSDiva 8.0.2

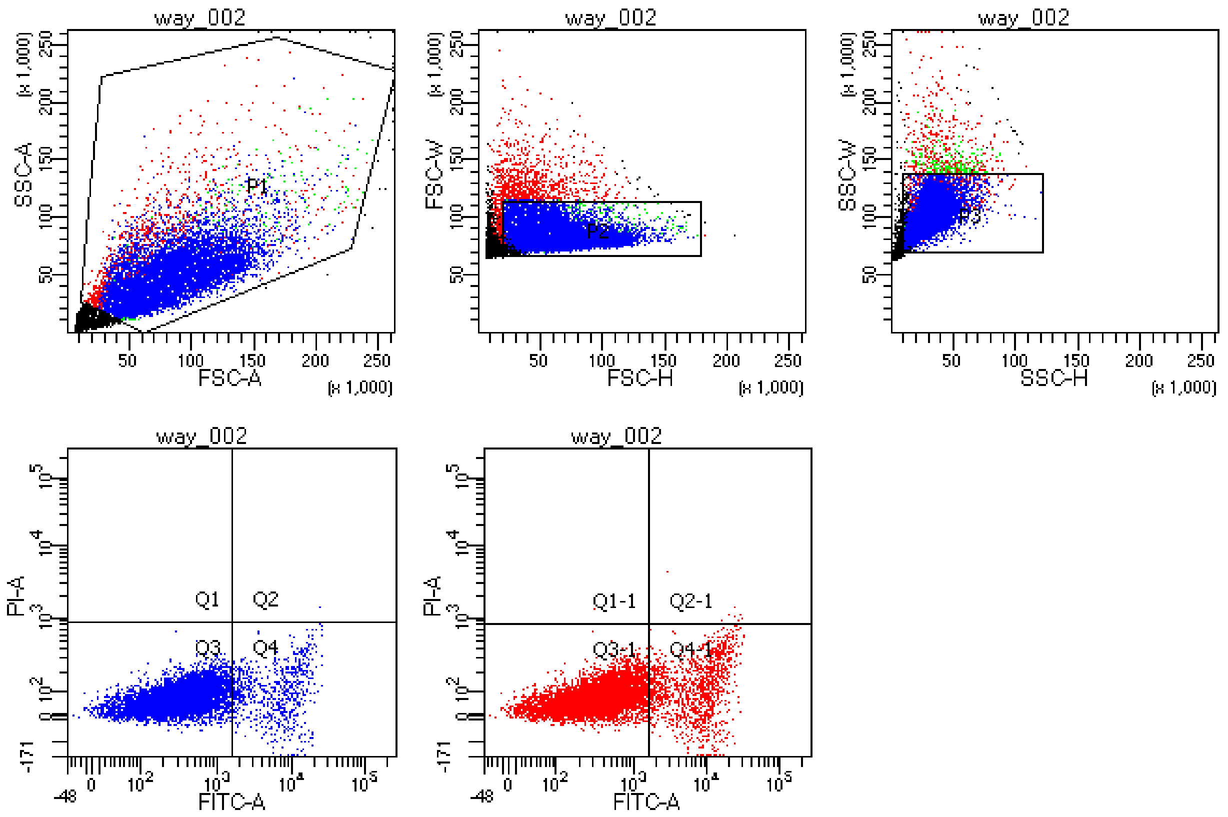

Tube: way_002

Population	#Events	%Parent	%Total
All Events	10,000	####	100.0
P1	8,564	85.6	85.6
P2	7,435	86.8	74.4
P3	7,237	97.3	72.4
Q1	0	0.0	0.0
Q2	1	0.0	0.0
Q3	6,511	90.0	65.1
Q4	725	10.0	7.2
Q1-1	1	0.0	0.0
Q2-1	10	0.1	0.1
Q3-1	7,275	84.9	72.8
Q4-1	1,278	14.9	12.8

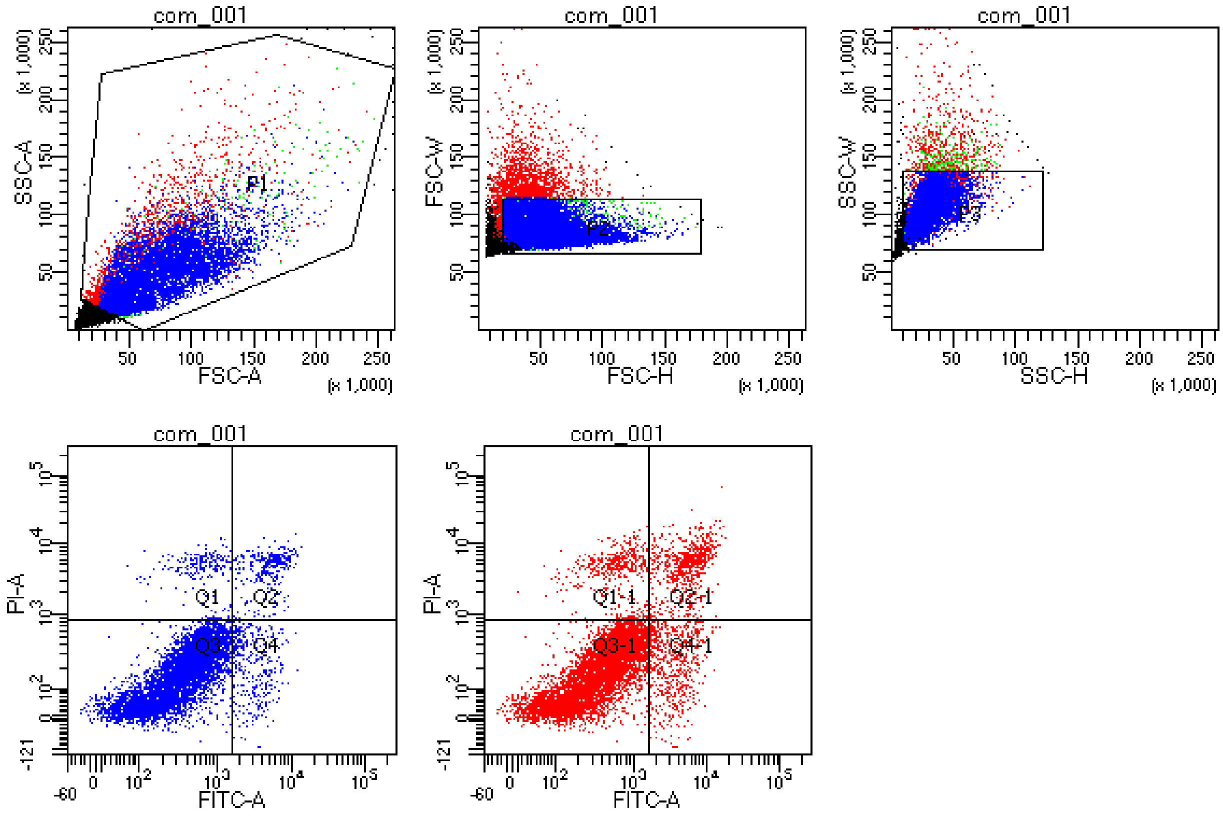

Tube: com_001

Population	#Events	%Parent	%Total
All Events	10,000	####	100.0
P1	8,419	84.2	84.2
P2	6,655	79.0	66.6
P3	6,449	96.9	64.5
Q1	234	3.6	2.3
Q2	332	5.1	3.3
Q3	5,522	85.6	55.2
Q4	361	5.6	3.6
Q1-1	520	6.2	5.2
Q2-1	772	9.2	7.7
Q3-1	6,506	77.3	65.1
Q4-1	621	7.4	6.2

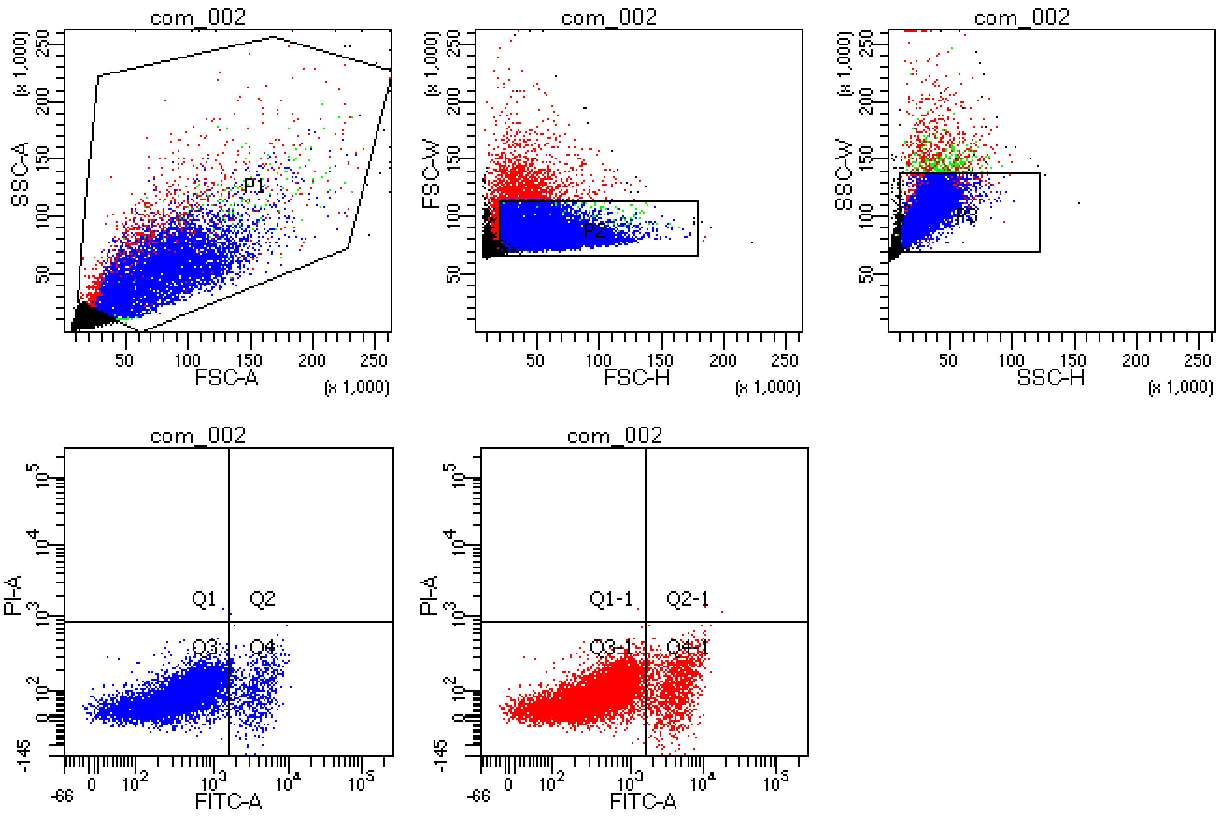

Tube: com_002

Population	#Events	%Parent	%Total
All Events	10,000	####	100.0
P1	8,326	83.3	83.3
P2	6,645	79.8	66.4
P3	6,401	96.3	64.0
Q1	1	0.0	0.0
Q2	1	0.0	0.0
Q3	5,764	90.0	57.6
Q4	635	9.9	6.4
Q1-1	1	0.0	0.0
Q2-1	3	0.0	0.0
Q3-1	7,086	85.1	70.9
Q4-1	1,236	14.8	12.4
