# Supplementary material for: Inhibition of Dickkopf-1 enhances the anti-tumor efficacy of sorafenib via inhibition of the PI3K/Akt and Wnt/β-catenin pathways in hepatocellular carcinoma
Source: Cell Commun Signal. 2023 Nov 27;21:339. doi: 10.1186/s12964-023-01355-2 (PMC10680194; doi:10.1186/s12964-023-01355-2)
Supplement: Supplementary file 3 — Additional file 2. [file 12964_2023_1355_MOESM2_ESM.zip › raw data/Figure 3/Figure 3C_Hep3B.pdf]

BD FACSDiva 8.0.2

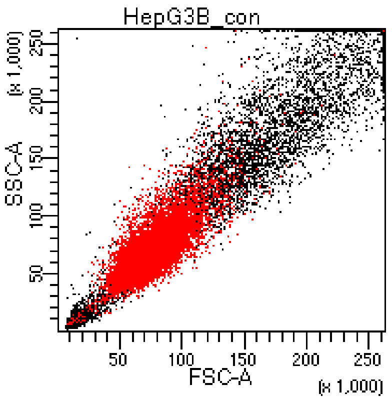

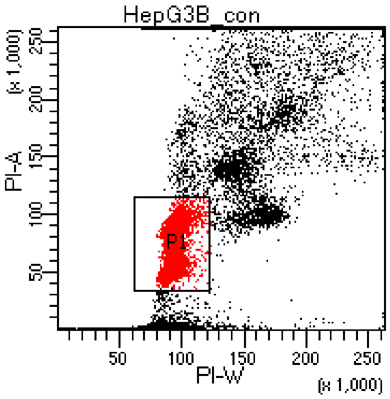

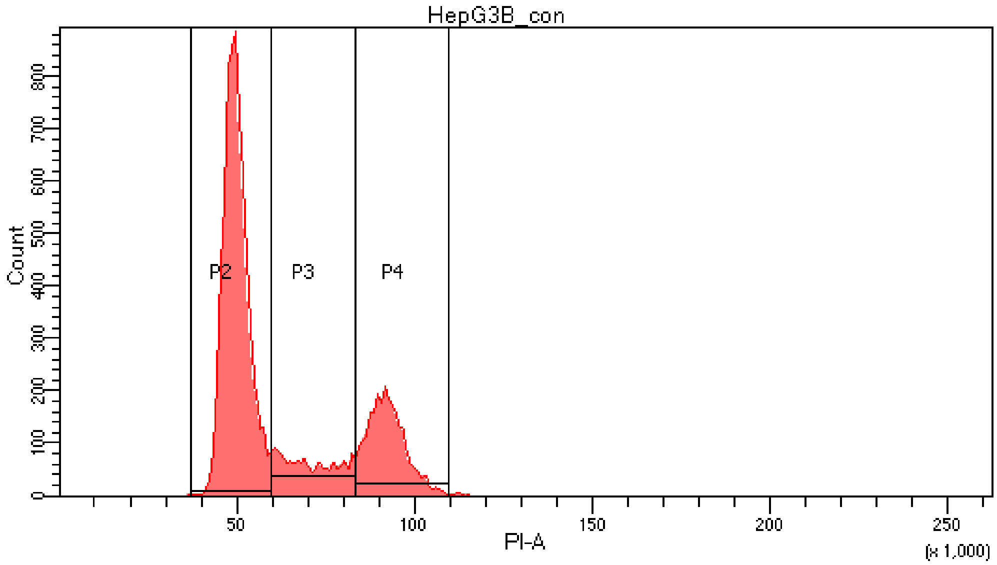

Tube: HepG3B_con

Population	#Events	%Parent	%Total
All Events	20,000	####	100.0
P1	11,089	55.4	55.4
P2	6,944	62.6	34.7
P3	1,559	14.1	7.8
P4	2,526	22.8	12.6

BD FACSDiva 8.0.2

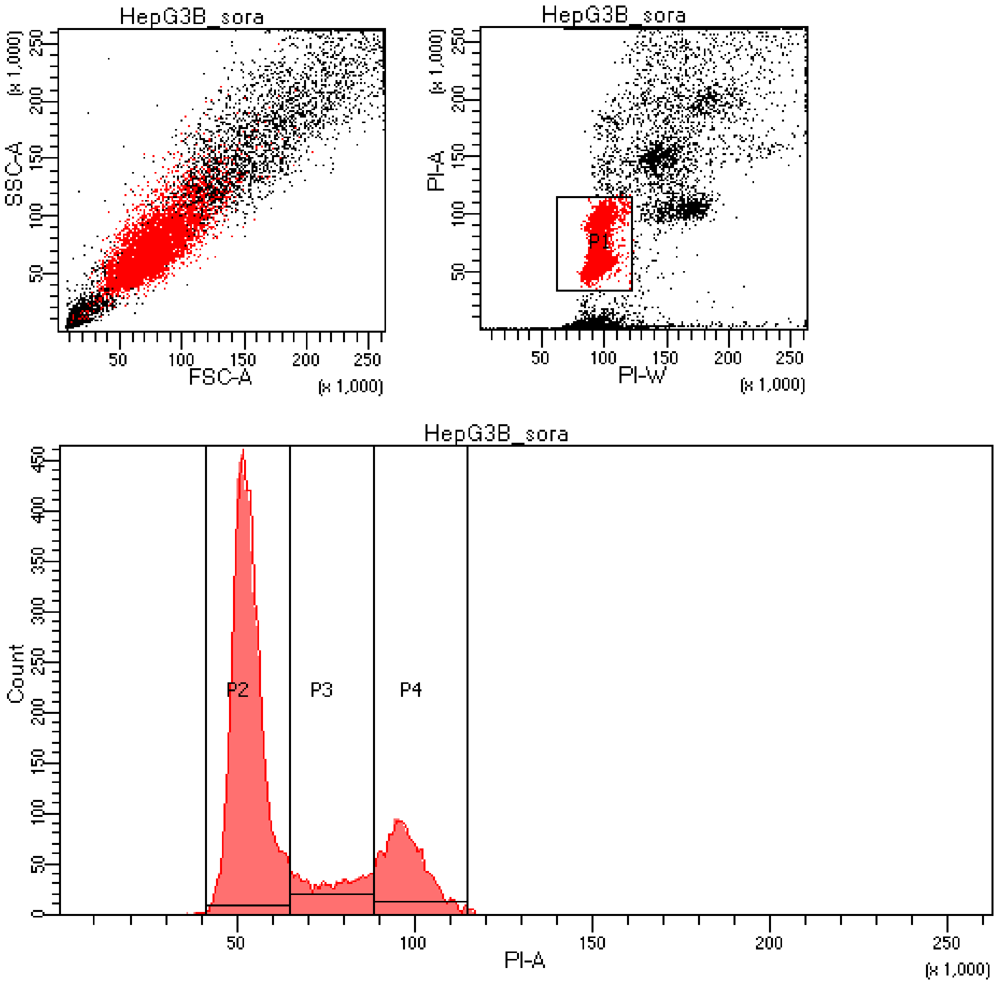

Tube: HepG3B_sora

Population	#Events	%Parent	%Total
All Events	12,621	####	100.0
P1	6,394	50.7	50.7
P2	4,261	66.6	33.8
P3	809	12.7	6.4
P4	1,292	20.2	10.2

BD FACSDiva 8.0.2

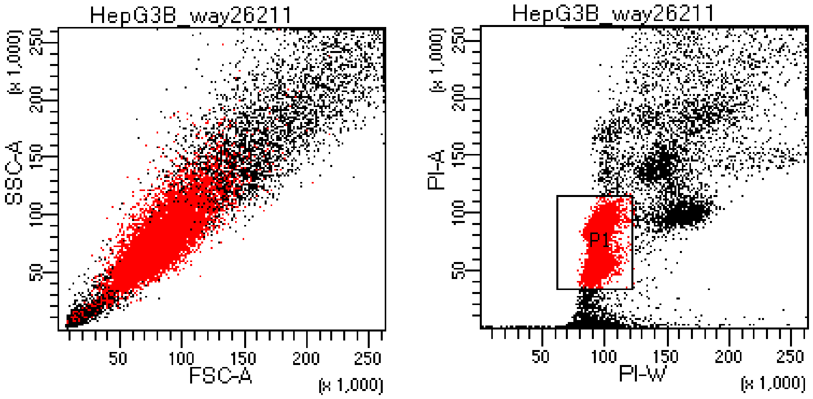

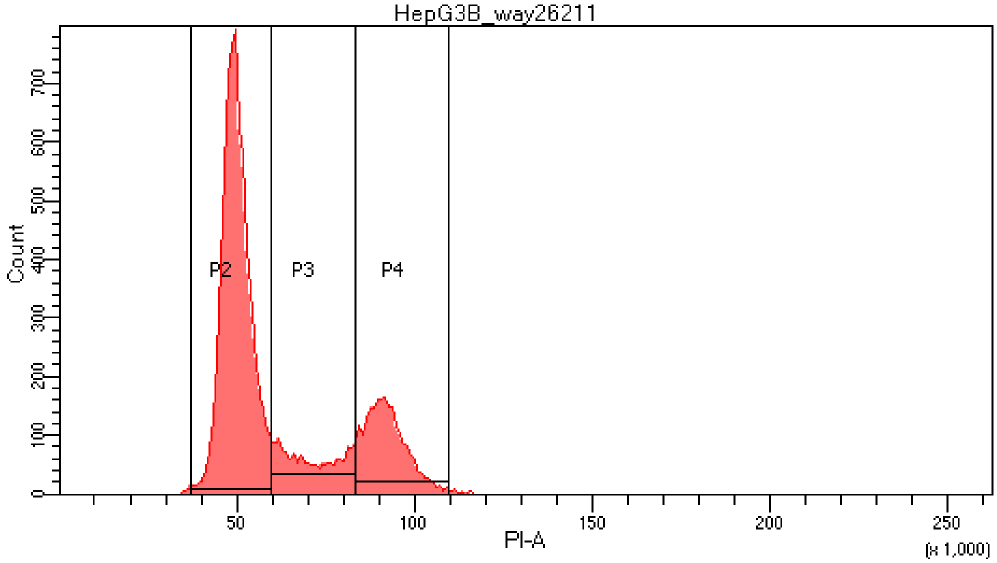

Tube: HepG3B_way26211

Population	#Events	%Parent	%Total
All Events	18,700	####	100.0
P1	10,699	57.2	57.2
P2	6,779	63.4	36.3
P3	1,551	14.5	8.3
P4	2,275	21.3	12.2

BD FACSDiva 8.0.2

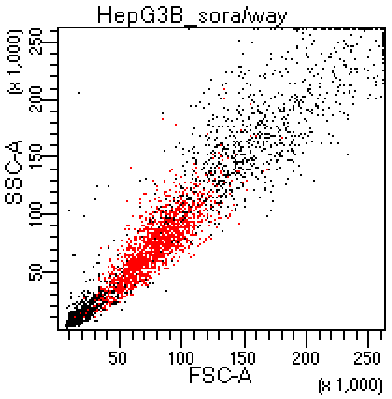

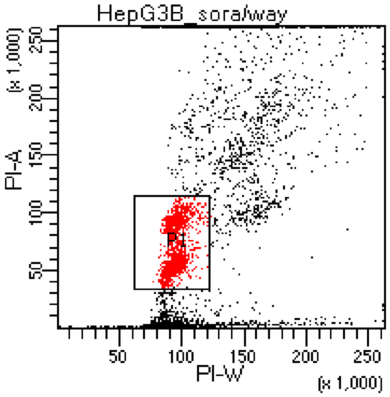

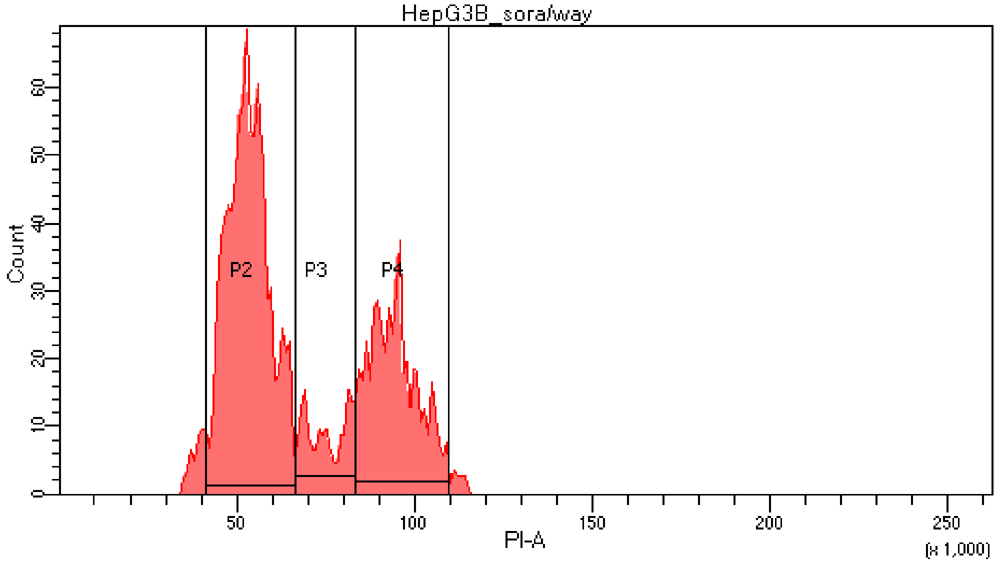

Tube: HepG3B_sora/way

Population	#Events	%Parent	%Total
All Events	3,923	####	100.0
P1	1,592	40.6	40.6
P2	891	56.0	22.7
P3	160	10.1	4.1
P4	478	30.0	12.2

BD FACSDiva 8.0.2

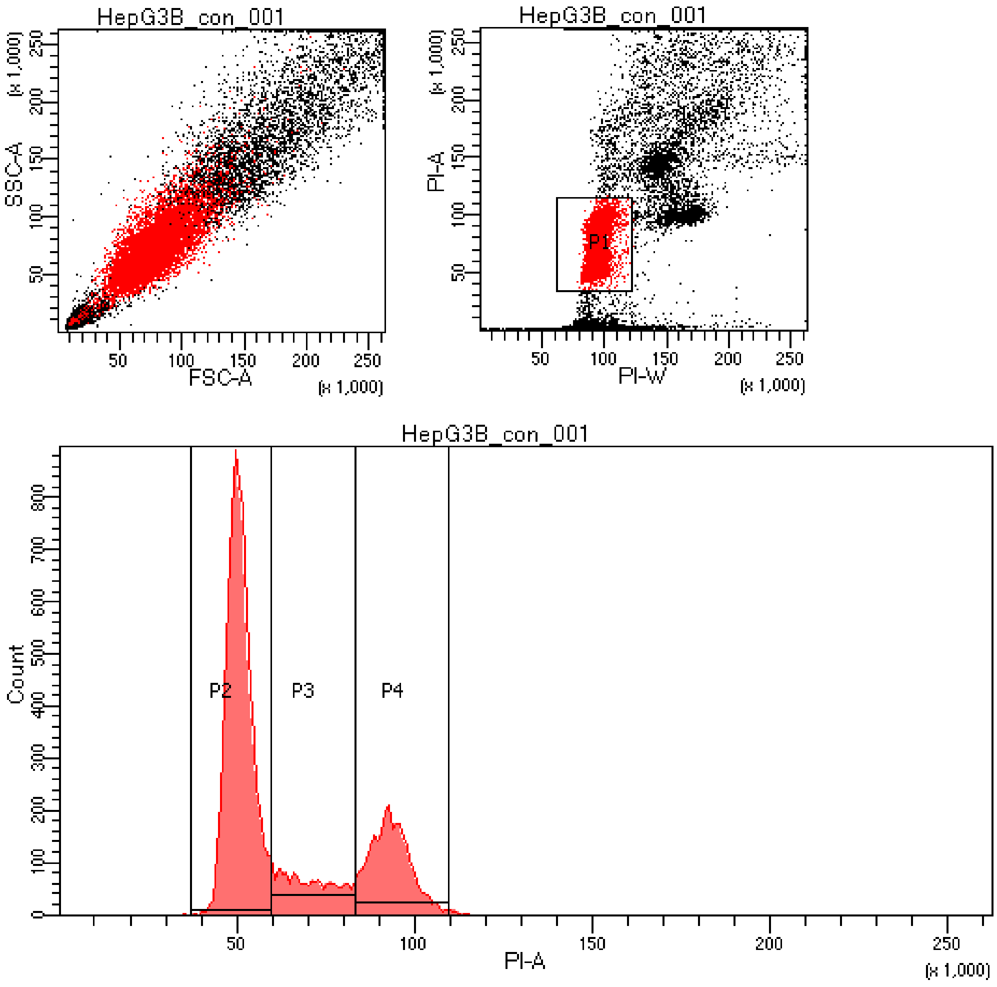

Tube: HepG3B_con_001

Population	#Events	%Parent	%Total
All Events	20,000	####	100.0
P1	11,214	56.1	56.1
P2	6,910	61.6	34.6
P3	1,590	14.2	8.0
P4	2,646	23.6	13.2

BD FACSDiva 8.0.2

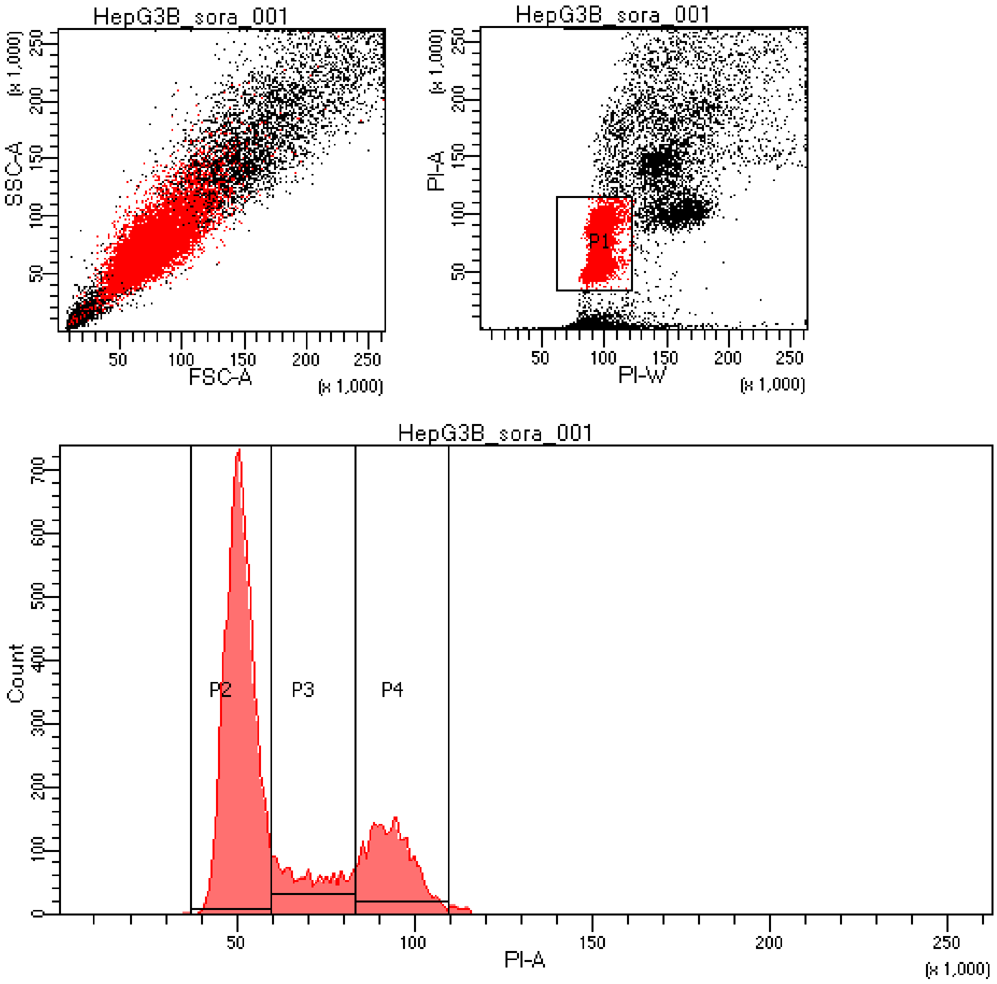

Tube: HepG3B_sora_001

Population	#Events	%Parent	%Total
All Events	18,952	####	100.0
P1	10,683	56.4	56.4
P2	6,742	63.1	35.6
P3	1,480	13.9	7.8
P4	2,364	22.1	12.5

BD FACSDiva 8.0.2

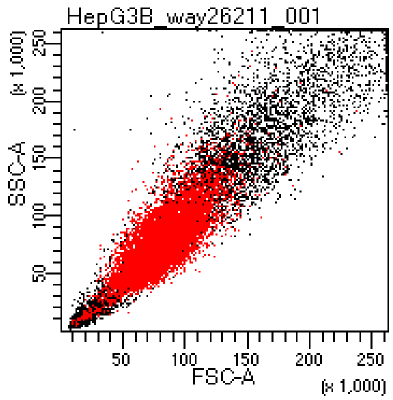

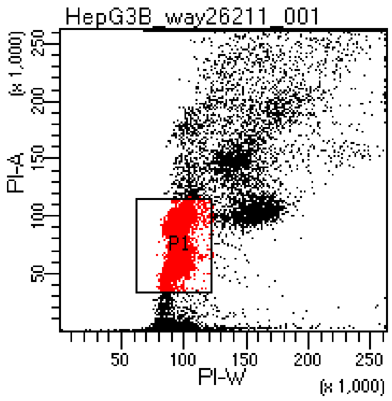

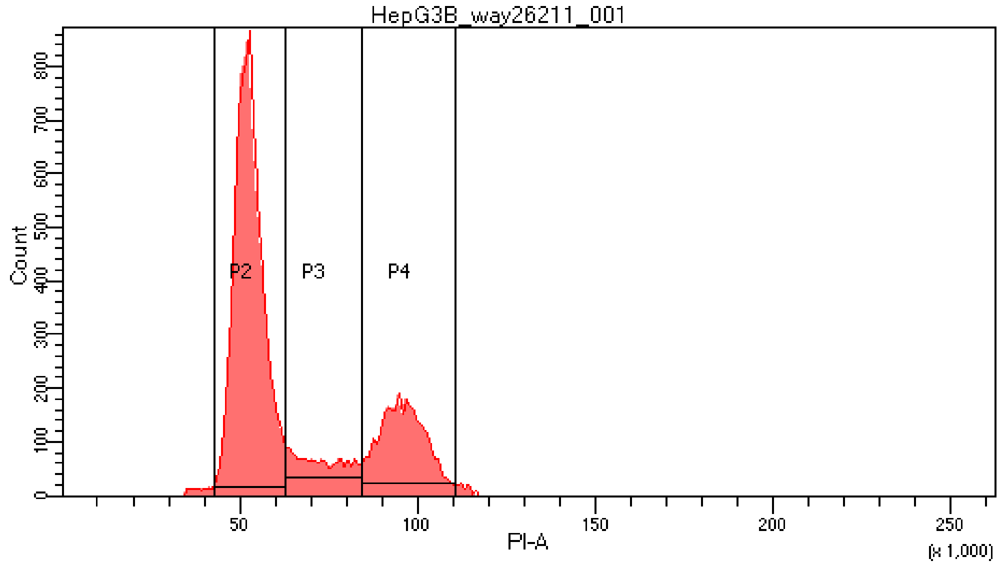

Tube: HepG3B_way26211_001

Population	#Events	%Parent	%Total
All Events	20,000	####	100.0
P1	12,463	62.3	62.3
P2	7,792	62.5	39.0
P3	1,465	11.8	7.3
P4	2,948	23.7	14.7

BD FACSDiva 8.0.2

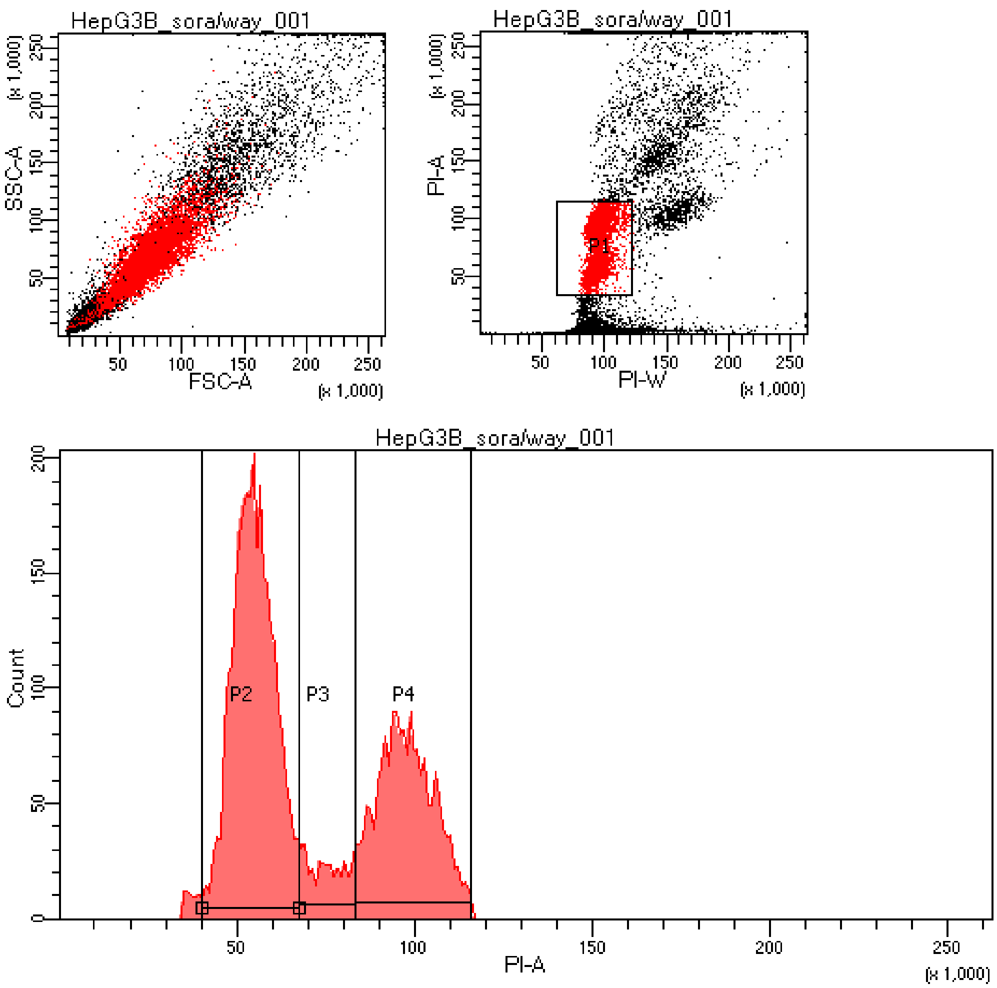

Tube: HepG3B_sora/way_001

Population	#Events	%Parent	%Total
All Events	10,529	####	100.0
P1	4,904	46.6	46.6
P2	2,757	56.2	26.2
P3	365	7.4	3.5
P4	1,712	34.9	16.3
