# Supplementary material for: Inhibition of Dickkopf-1 enhances the anti-tumor efficacy of sorafenib via inhibition of the PI3K/Akt and Wnt/β-catenin pathways in hepatocellular carcinoma
Source: Cell Commun Signal. 2023 Nov 27;21:339. doi: 10.1186/s12964-023-01355-2 (PMC10680194; doi:10.1186/s12964-023-01355-2)
Supplement: Supplementary file 3 — Additional file 2. [file 12964_2023_1355_MOESM2_ESM.zip › raw data/Figure 3/Figure 3C_Huh7.pdf]

BD FACSDiva 8.0.2

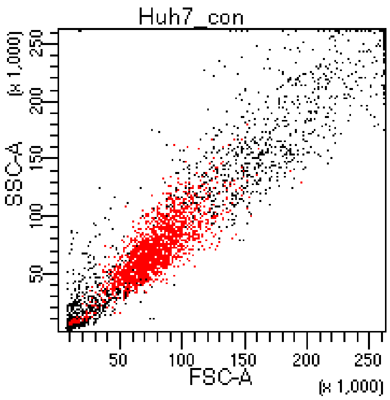

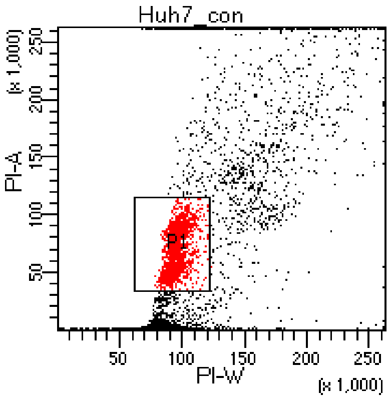

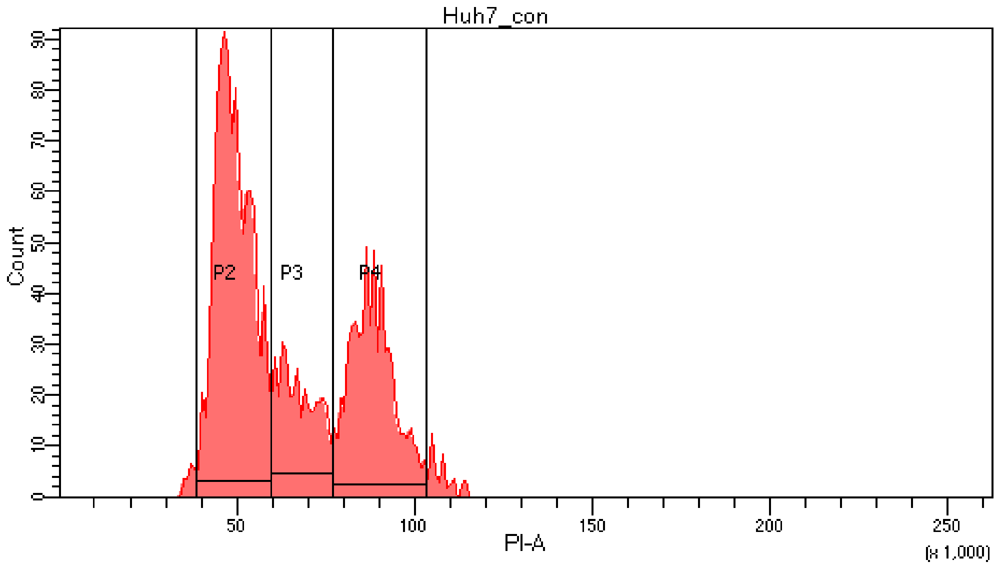

Tube: Huh7_con

Population	#Events	%Parent	%Total
All Events	4,417	####	100.0
P1	2,109	47.7	47.7
P2	1,070	50.7	24.2
P3	347	16.5	7.9
P4	616	29.2	13.9

BD FACSDiva 8.0.2

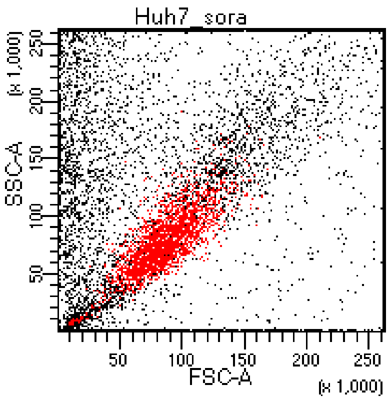

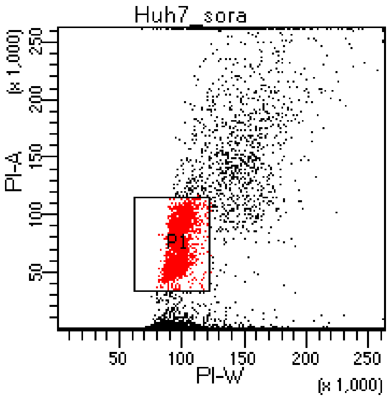

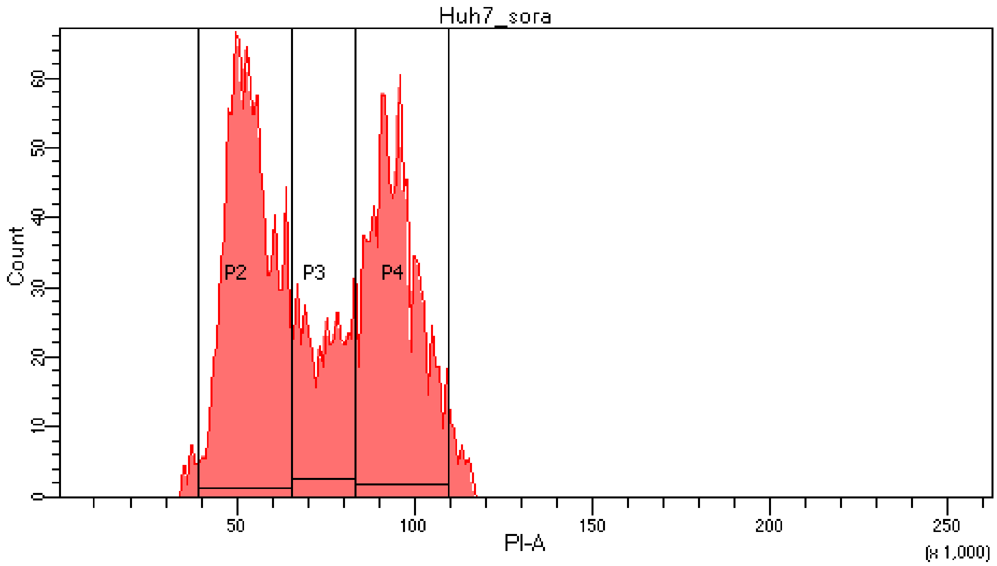

Tube: Huh7_sora

Population	#Events	%Parent	%Total
All Events	17,152	####	100.0
P1	2,369	13.8	13.8
P2	1,007	42.5	5.9
P3	412	17.4	2.4
P4	881	37.2	5.1

BD FACSDiva 8.0.2

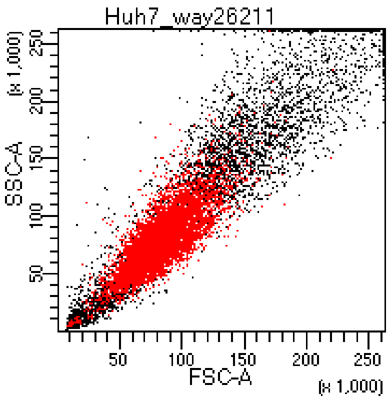

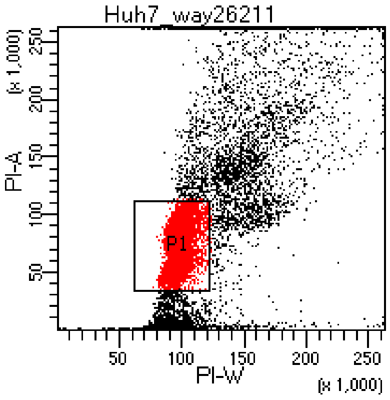

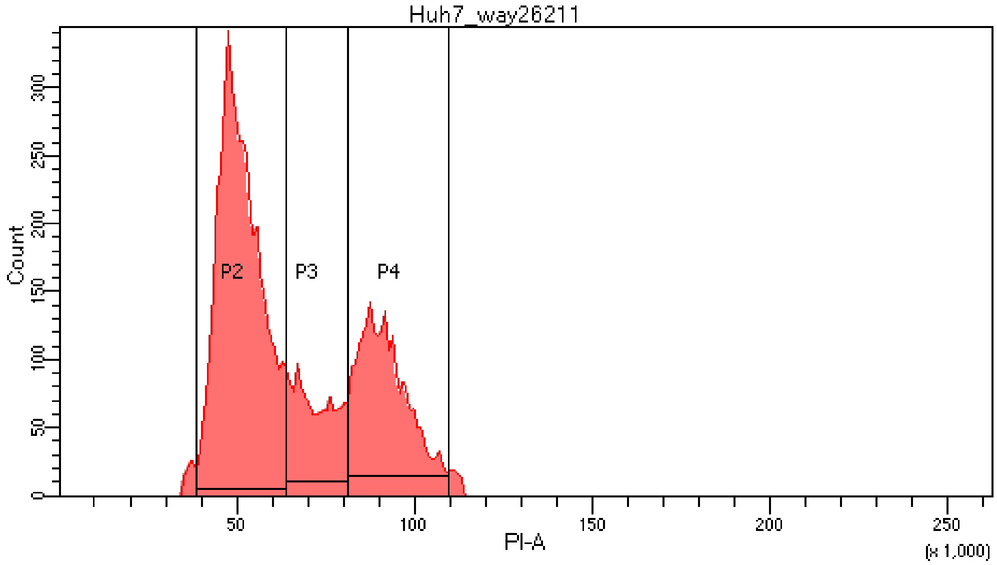

Tube: Huh7_way26211

Population	#Events	%Parent	%Total
All Events	13,112	####	100.0
P1	8,025	61.2	61.2
P2	4,425	55.1	33.7
P3	1,196	14.9	9.1
P4	2,238	27.9	17.1

BD FACSDiva 8.0.2

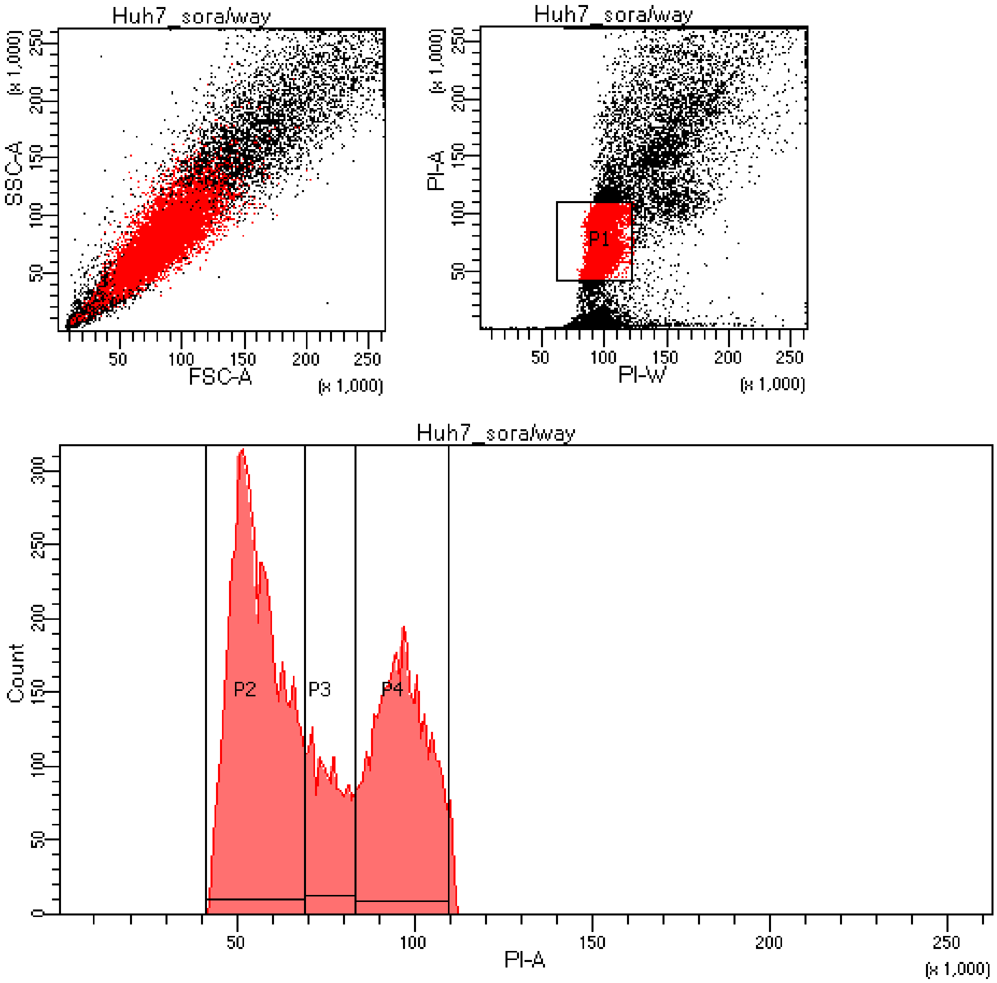

Tube: Huh7_sora/way

Population	#Events	%Parent	%Total
All Events	20,000	####	100.0
P1	9,739	48.7	48.7
P2	4,954	50.9	24.8
P3	1,331	13.7	6.7
P4	3,368	34.6	16.8

BD FACSDiva 8.0.2

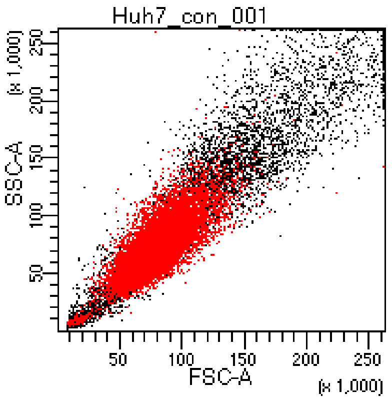

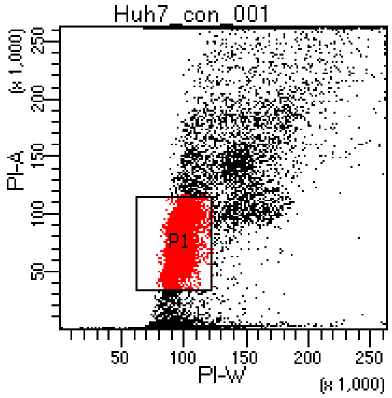

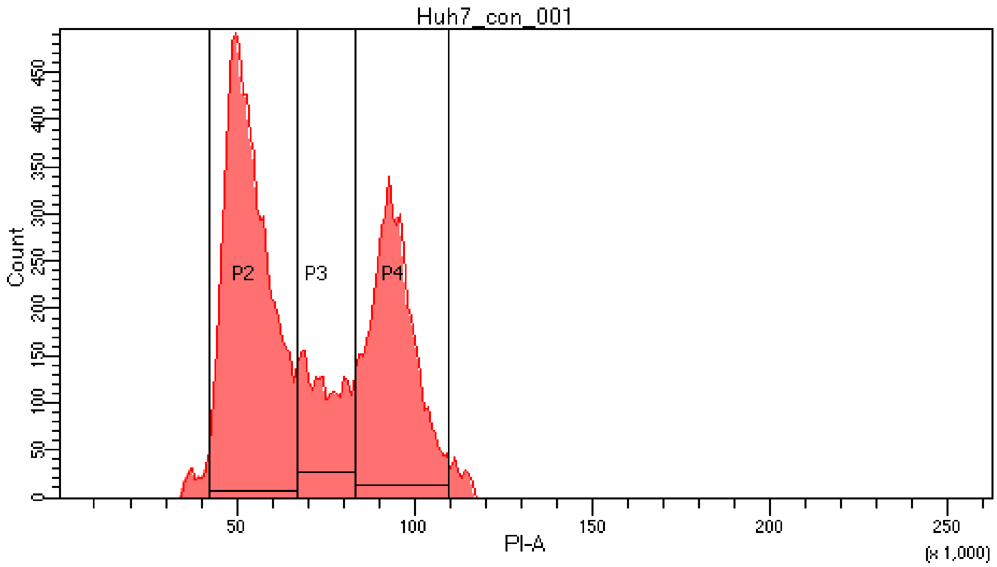

Tube: Huh7_con_001

Population	#Events	%Parent	%Total
All Events	20,000	####	100.0
P1	13,874	69.4	69.4
P2	6,852	49.4	34.3
P3	1,968	14.2	9.8
P4	4,622	33.3	23.1

BD FACSDiva 8.0.2

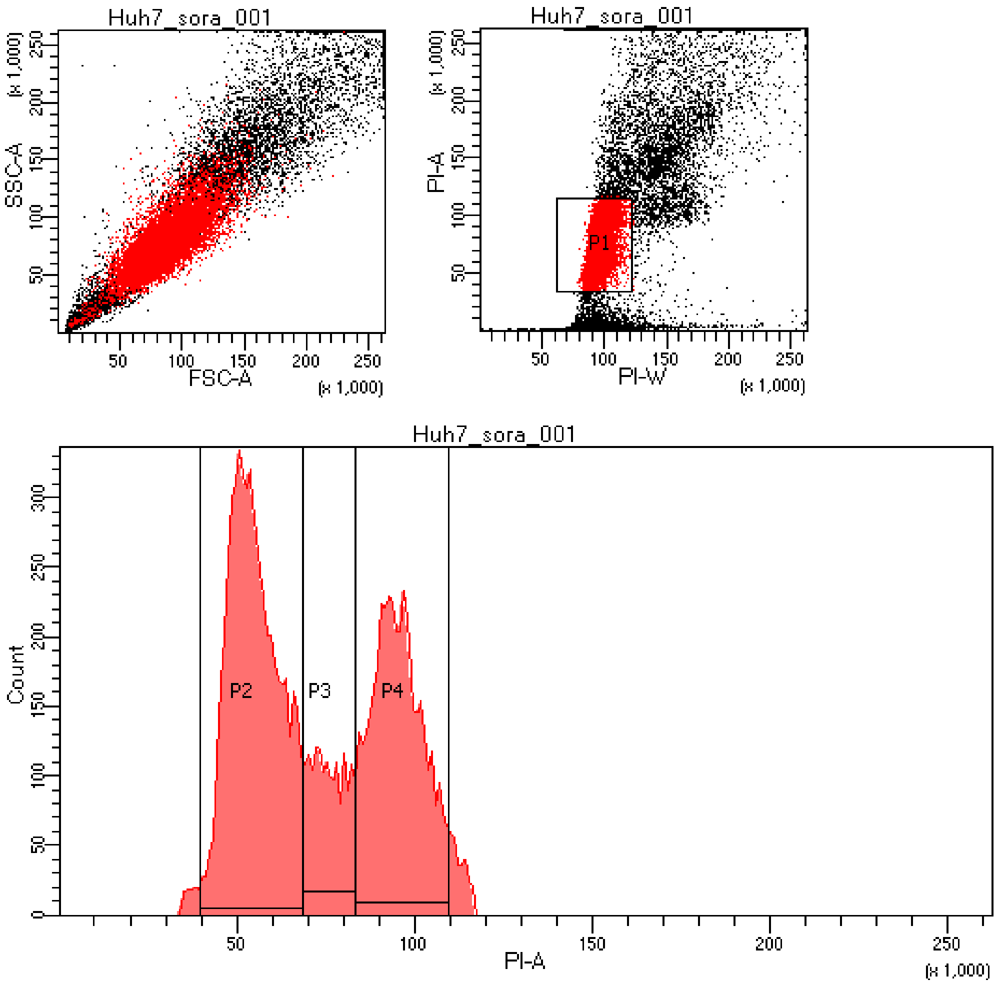

Tube: Huh7_sora_001

Population	#Events	%Parent	%Total
All Events	20,000	####	100.0
P1	11,454	57.3	57.3
P2	5,535	48.3	27.7
P3	1,535	13.4	7.7
P4	4,004	35.0	20.0

BD FACSDiva 8.0.2

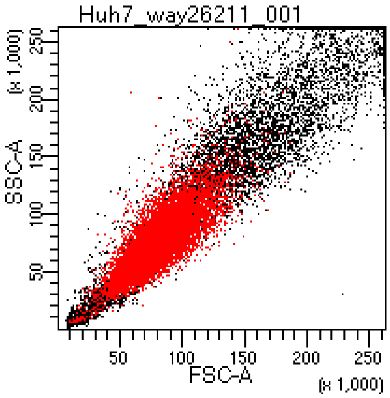

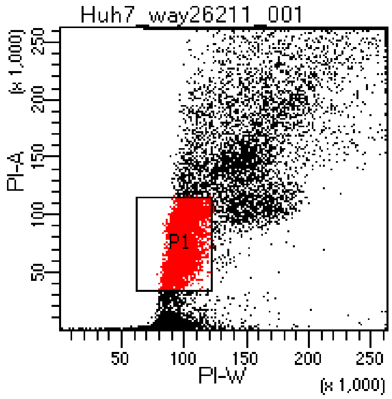

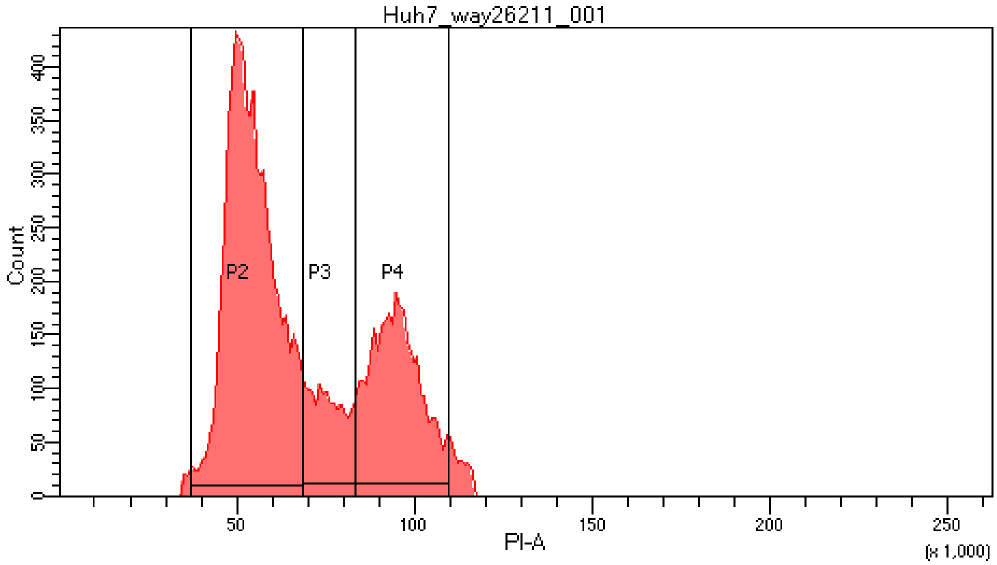

Tube: Huh7_way26211_001

Population	#Events	%Parent	%Total
All Events	20,000	####	100.0
P1	11,431	57.2	57.2
P2	6,688	58.5	33.4
P3	1,311	11.5	6.6
P4	3,129	27.4	15.6

BD FACSDiva 8.0.2

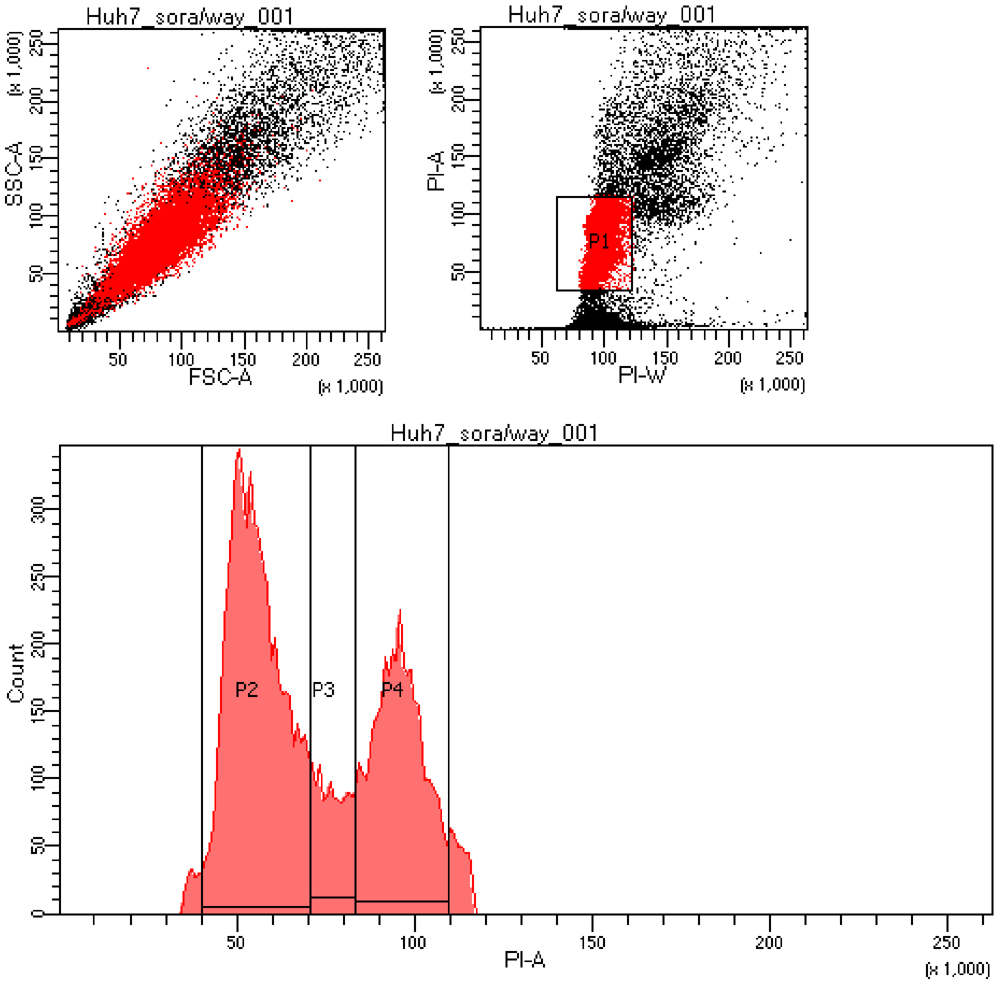

Tube: Huh7_sora/way_001

Population	#Events	%Parent	%Total
All Events	20,000	####	100.0
P1	11,122	55.6	55.6
P2	5,962	53.6	29.8
P3	1,128	10.1	5.6
P4	3,540	31.8	17.7
