# Supplementary figures and images for: Inhibition of Dickkopf-1 enhances the anti-tumor efficacy of sorafenib via inhibition of the PI3K/Akt and Wnt/β-catenin pathways in hepatocellular carcinoma (part 2 of 2)
Source: Cell Commun Signal. 2023 Nov 27;21:339. doi: 10.1186/s12964-023-01355-2 (PMC10680194; doi:10.1186/s12964-023-01355-2)

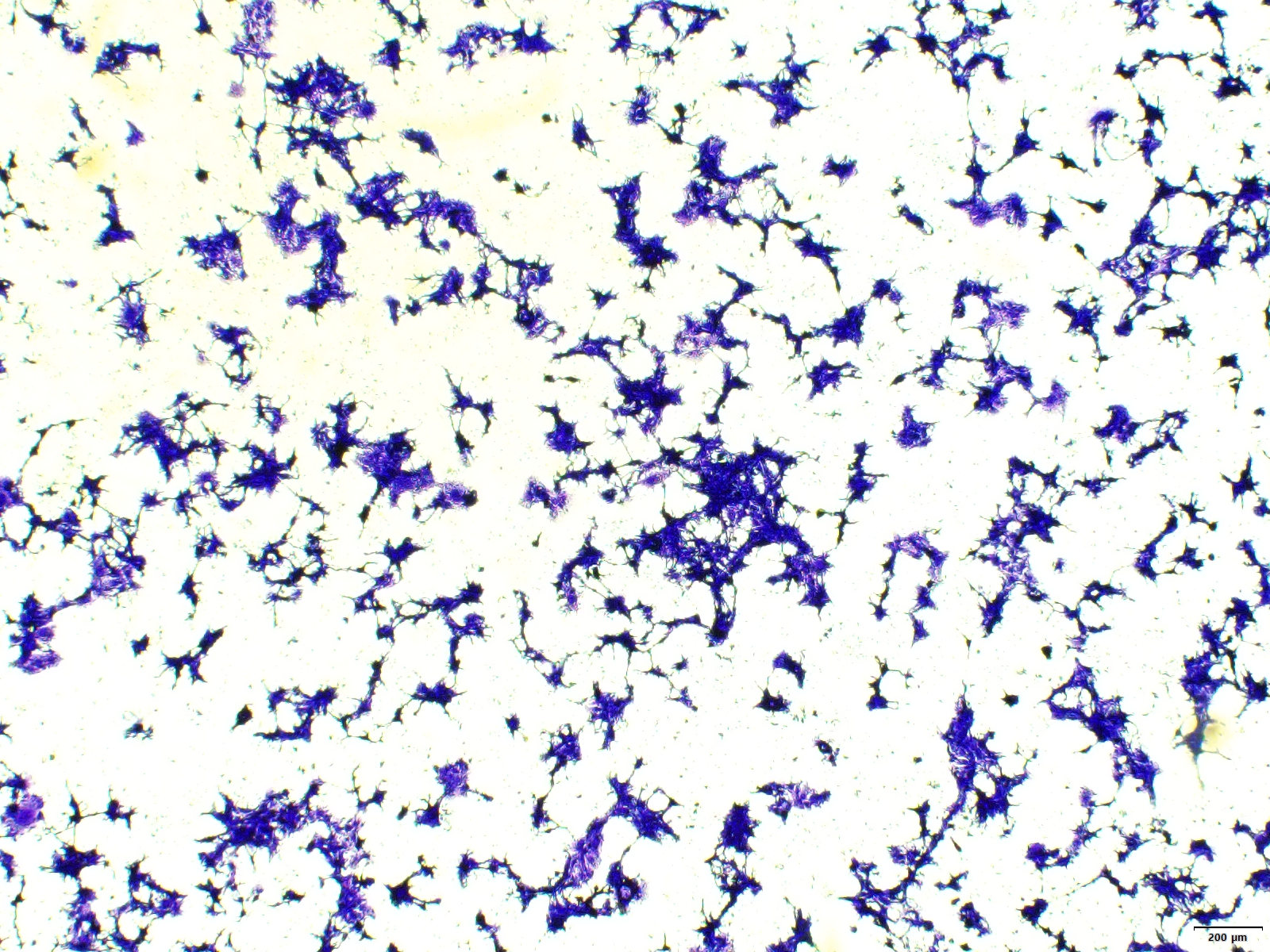

Supplement: Supplementary file 3 — Additional file 2. [file 12964_2023_1355_MOESM2_ESM.zip › raw data/Figure 4/Figure 4E/Hep3B/SOR 1 ╬╝M+LY294002 25 ╬╝M.jpg]

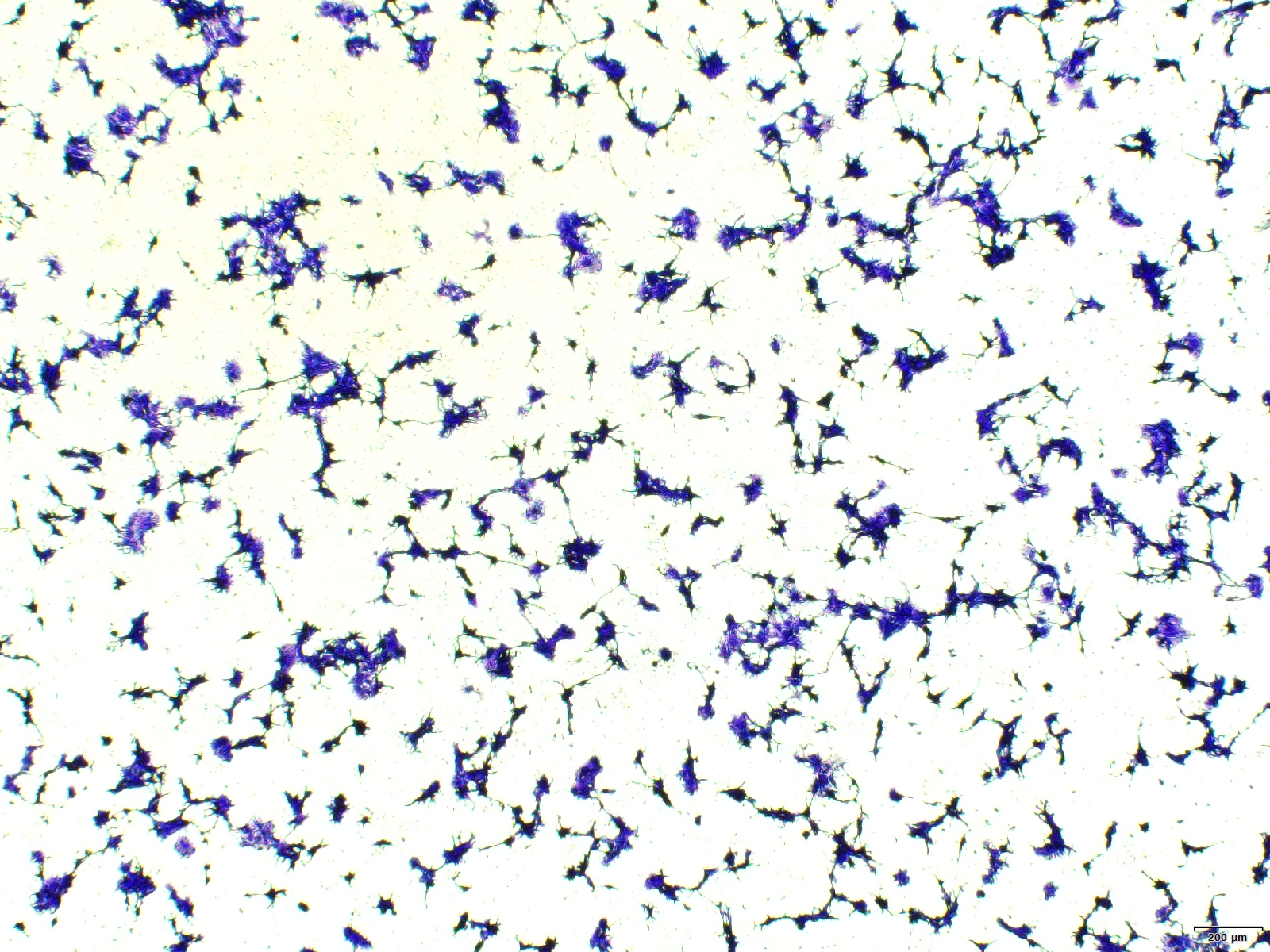

Supplement: Supplementary file 3 — Additional file 2. [file 12964_2023_1355_MOESM2_ESM.zip › raw data/Figure 4/Figure 4E/Hep3B/SOR 2 ╬╝M+LY294002 25 ╬╝M.jpg]

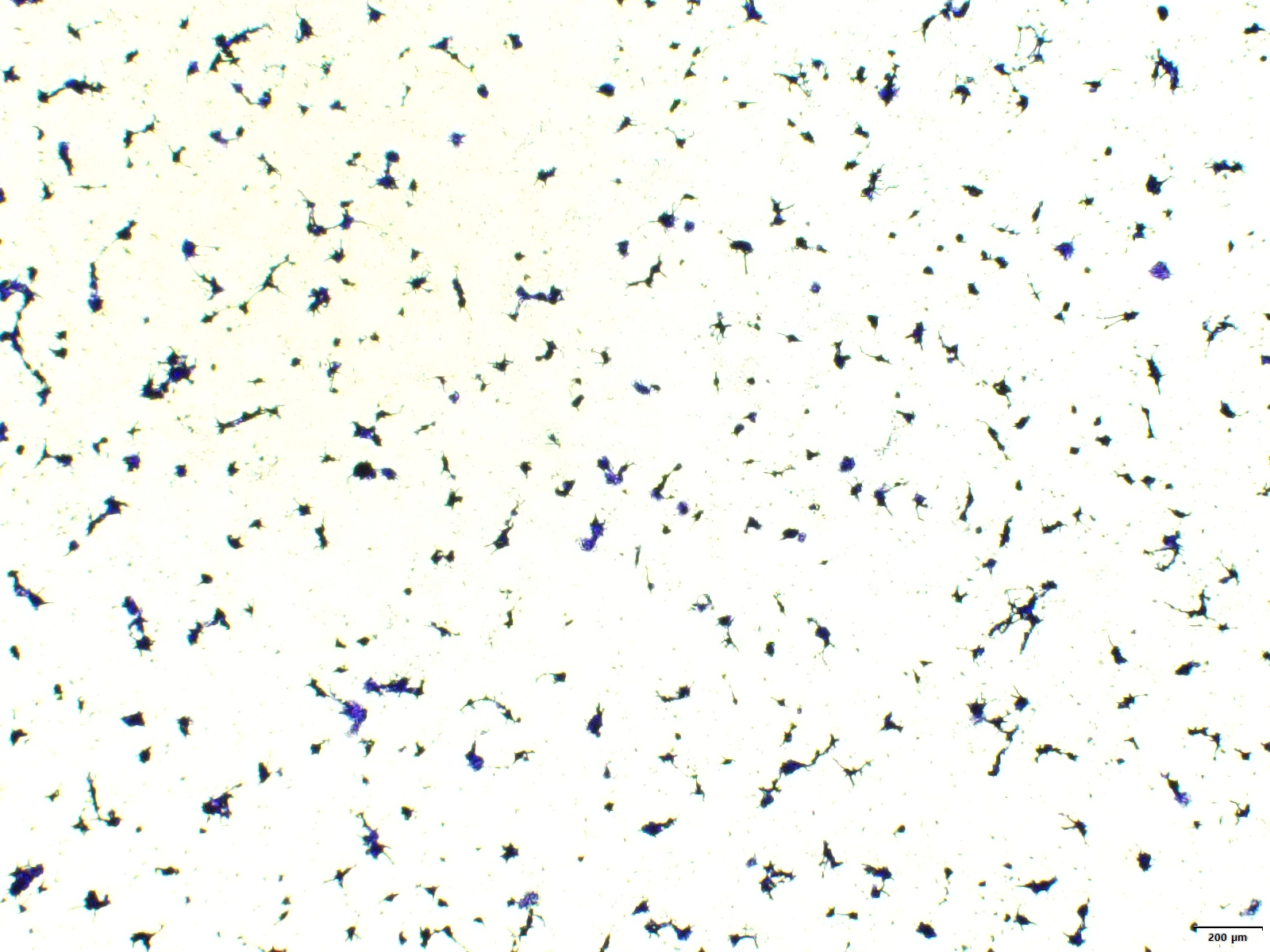

Supplement: Supplementary file 3 — Additional file 2. [file 12964_2023_1355_MOESM2_ESM.zip › raw data/Figure 4/Figure 4E/Hep3B/SOR 4 ╬╝M+LY294002 25 ╬╝M.jpg]

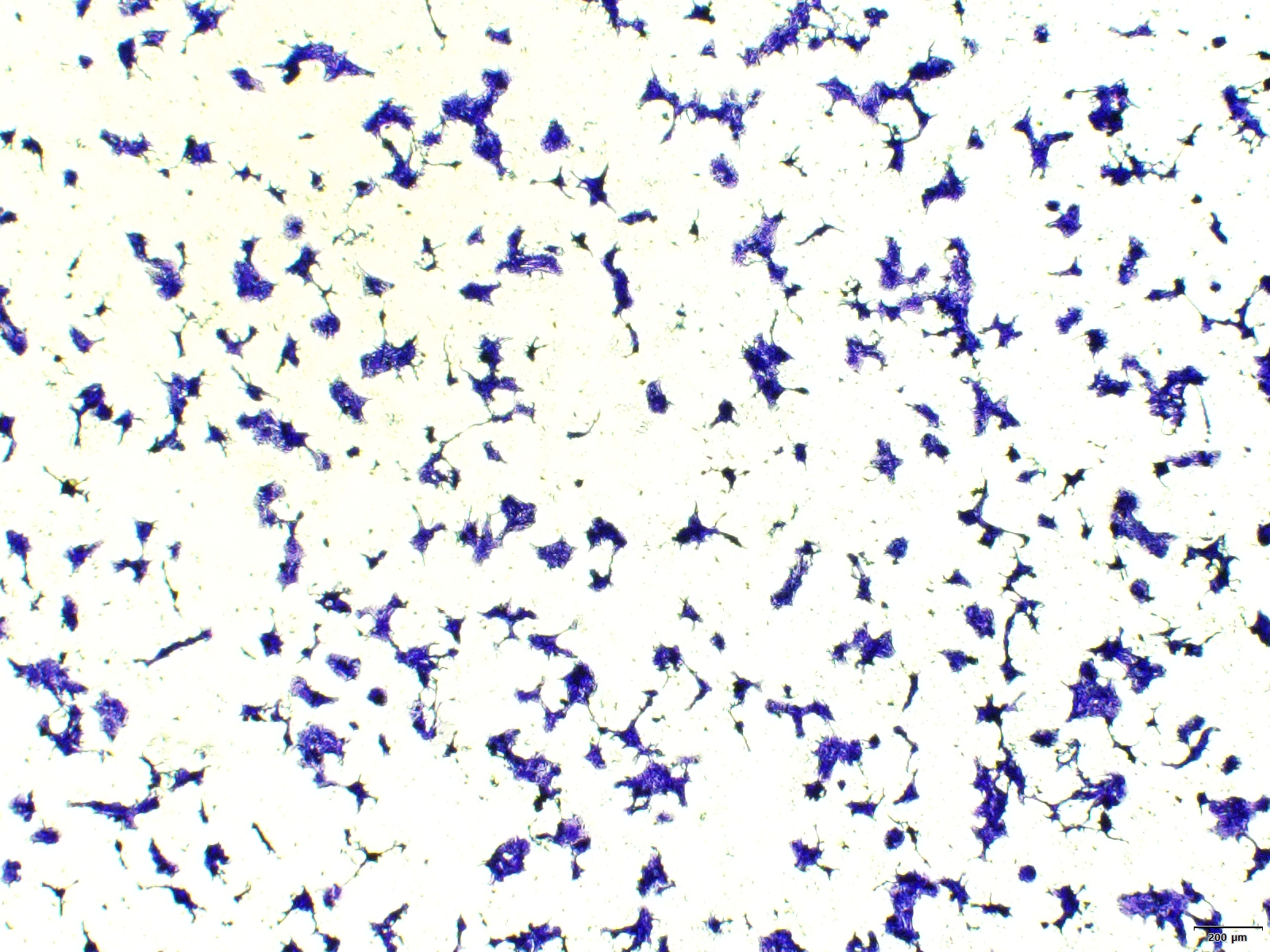

Supplement: Supplementary file 3 — Additional file 2. [file 12964_2023_1355_MOESM2_ESM.zip › raw data/Figure 4/Figure 4E/Hep3B/SOR 0 ╬╝M+LY294002 50 ╬╝M.jpg]

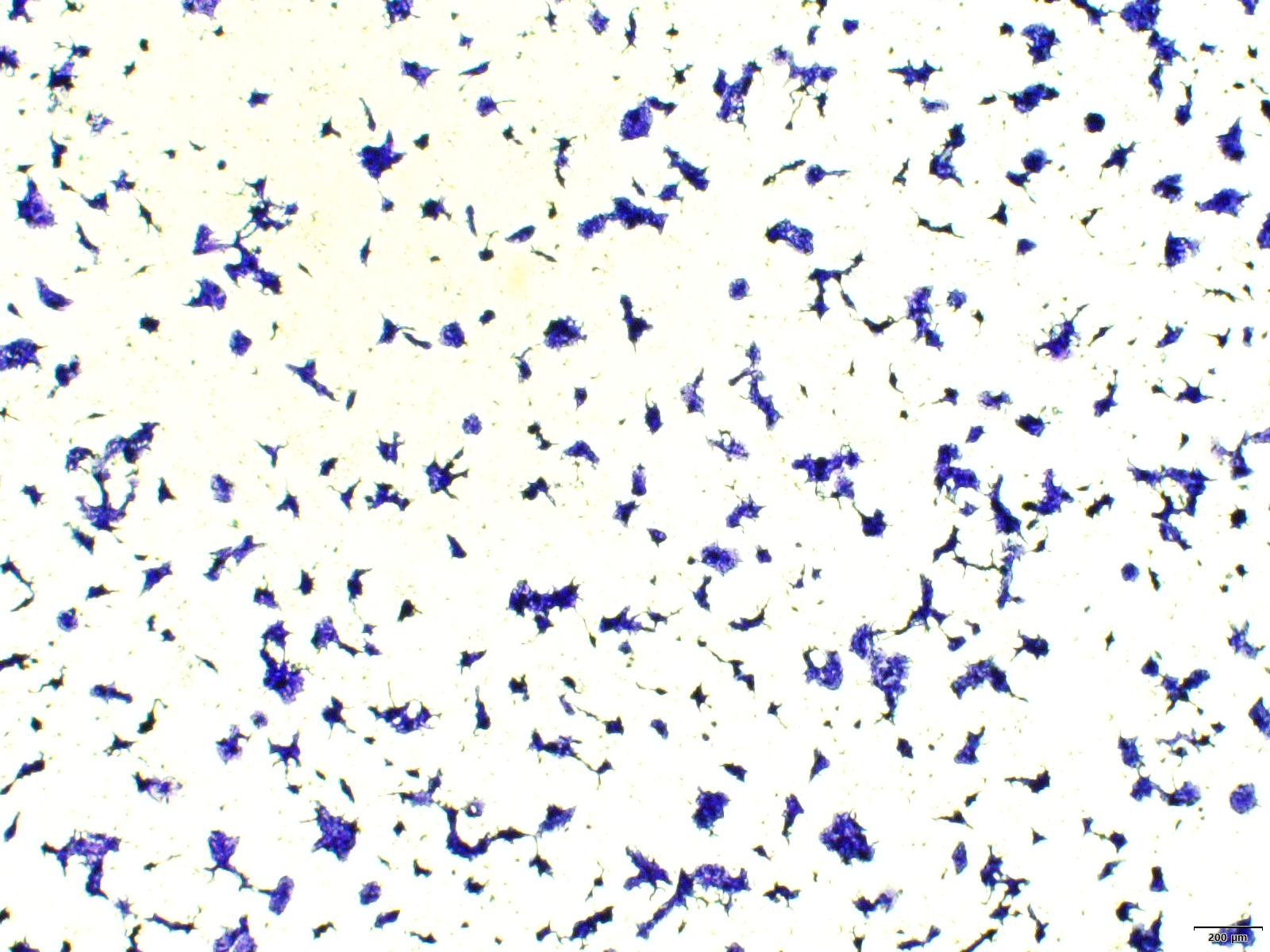

Supplement: Supplementary file 3 — Additional file 2. [file 12964_2023_1355_MOESM2_ESM.zip › raw data/Figure 4/Figure 4E/Hep3B/SOR 1 ╬╝M+LY294002 50 ╬╝M.jpg]

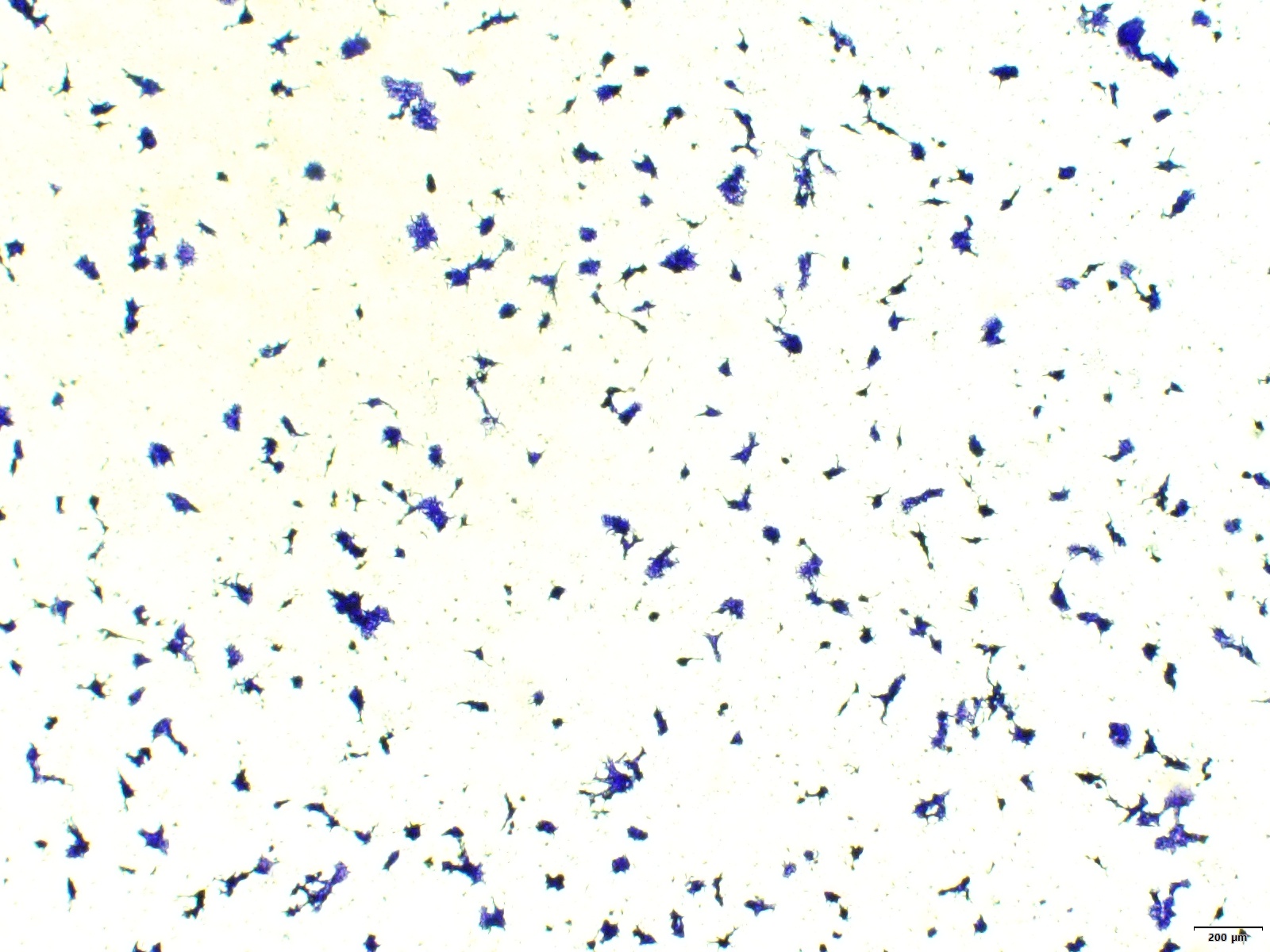

Supplement: Supplementary file 3 — Additional file 2. [file 12964_2023_1355_MOESM2_ESM.zip › raw data/Figure 4/Figure 4E/Hep3B/SOR 2 ╬╝M+LY294002 50 ╬╝M.jpg]

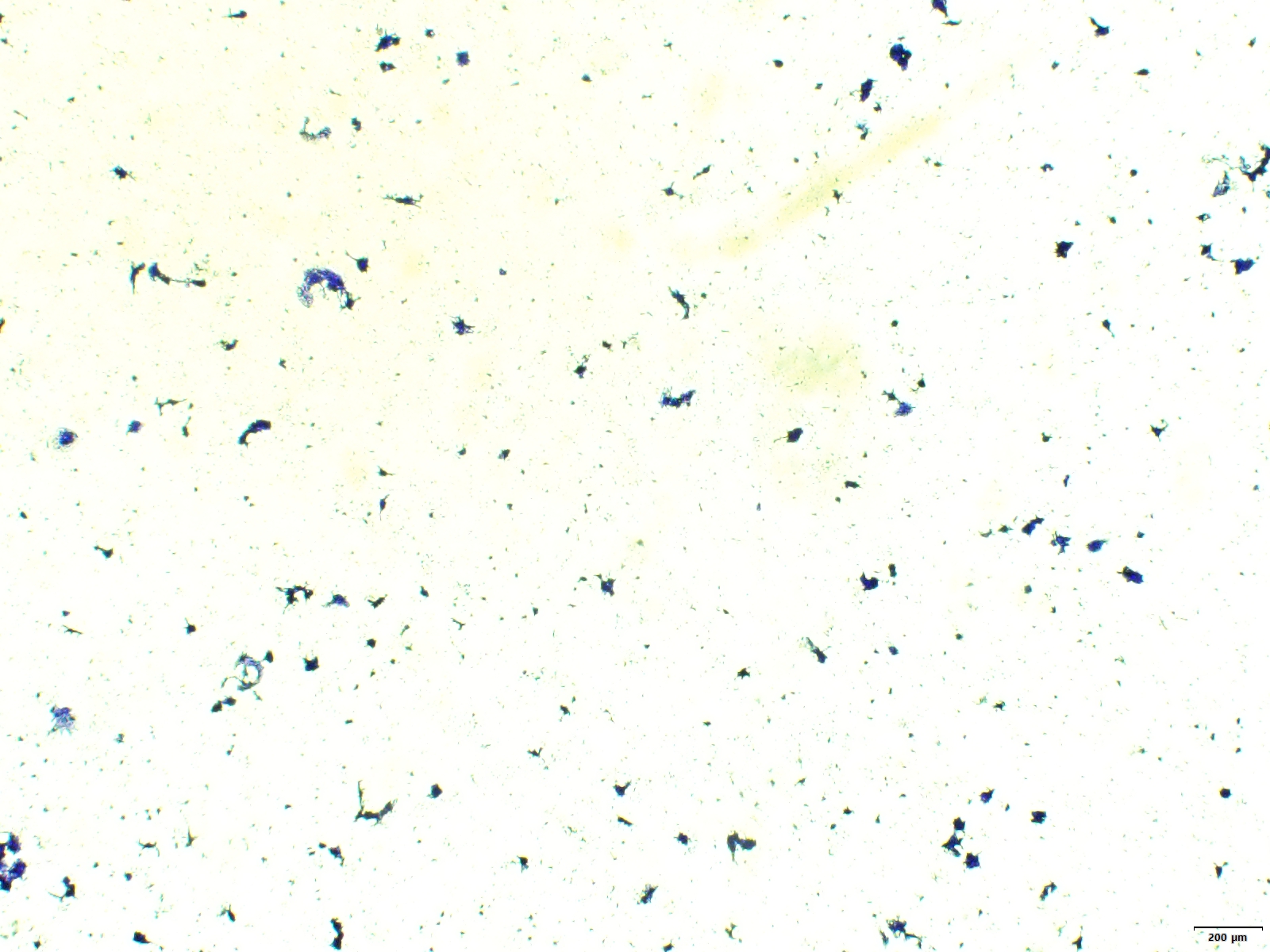

Supplement: Supplementary file 3 — Additional file 2. [file 12964_2023_1355_MOESM2_ESM.zip › raw data/Figure 4/Figure 4E/Hep3B/SOR 4 ╬╝M+LY294002 50 ╬╝M.jpg]

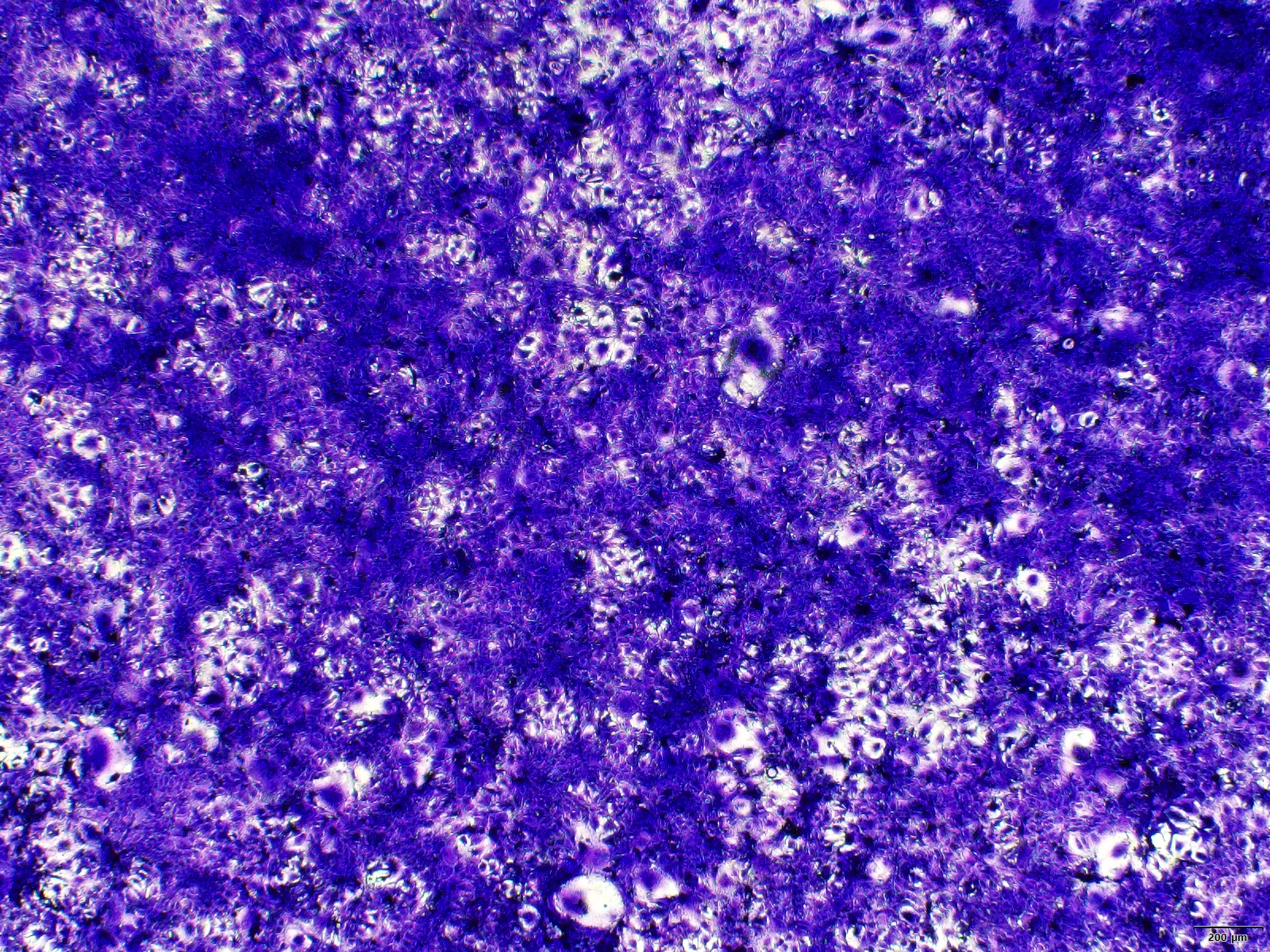

Supplement: Supplementary file 3 — Additional file 2. [file 12964_2023_1355_MOESM2_ESM.zip › raw data/Figure 4/Figure 4E/Huh7/SOR 0 ╬╝M+LY294002 0 ╬╝M.jpg]

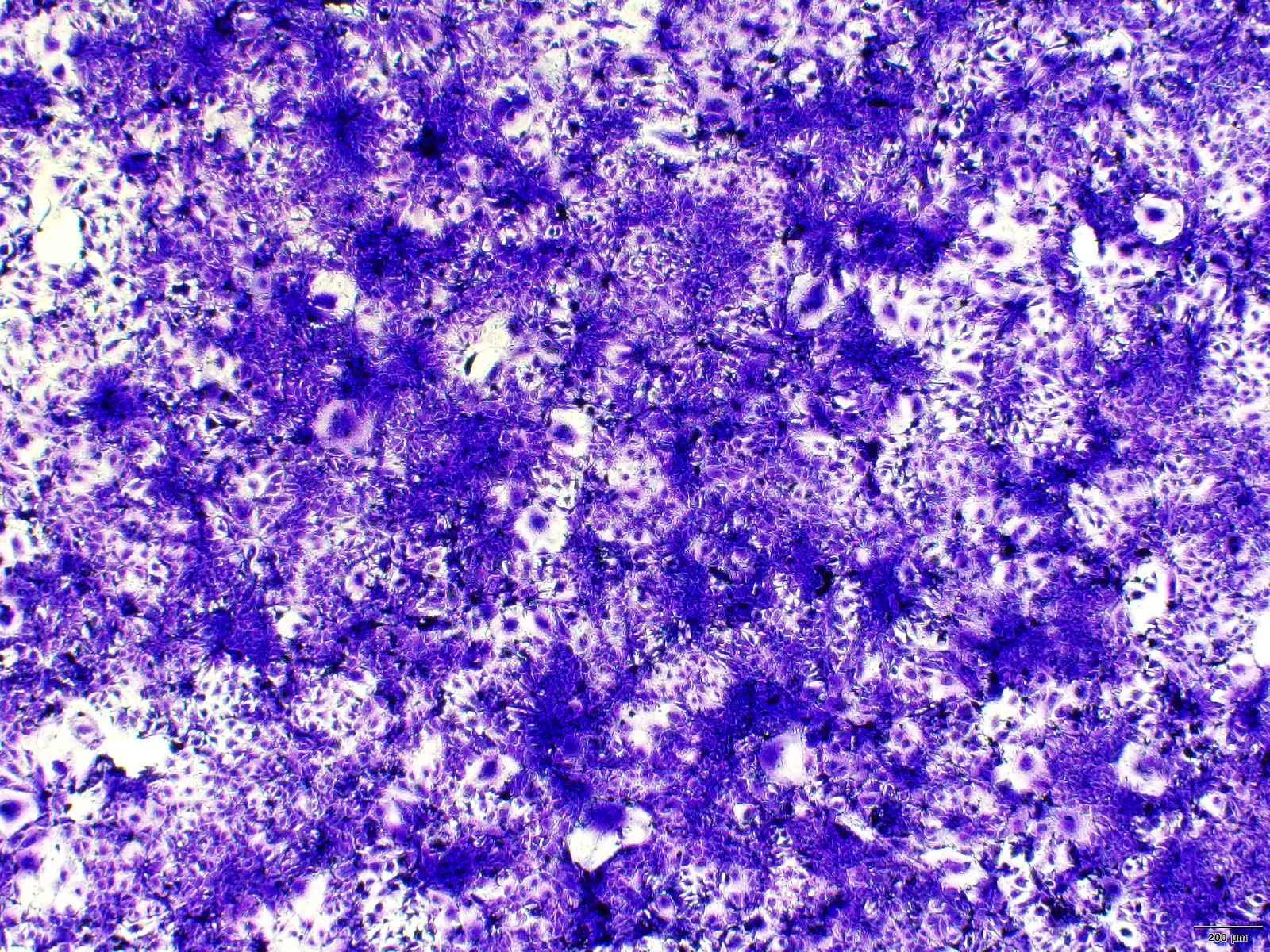

Supplement: Supplementary file 3 — Additional file 2. [file 12964_2023_1355_MOESM2_ESM.zip › raw data/Figure 4/Figure 4E/Huh7/SOR 1.5 ╬╝M+LY294002 0 ╬╝M.jpg]

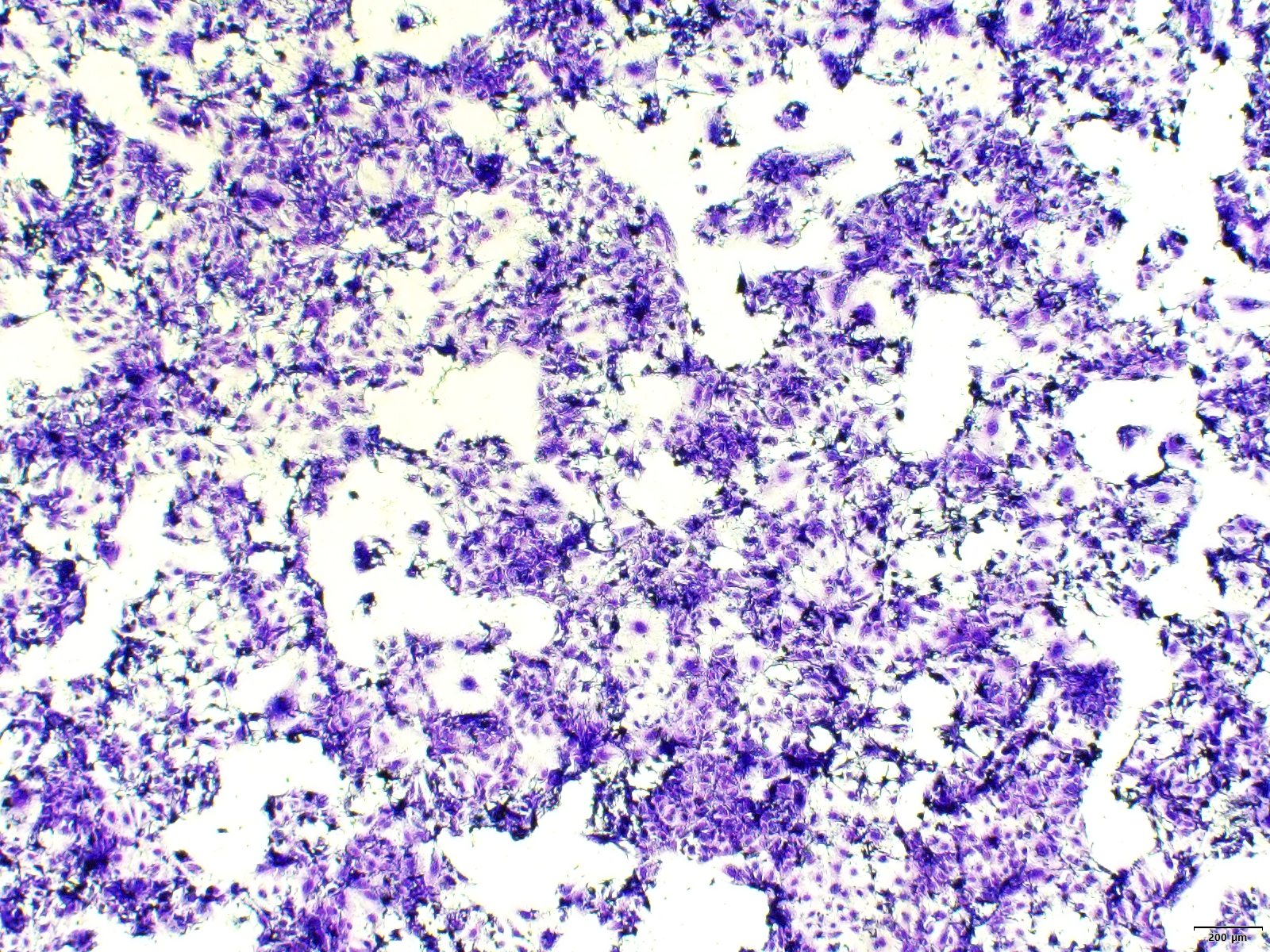

Supplement: Supplementary file 3 — Additional file 2. [file 12964_2023_1355_MOESM2_ESM.zip › raw data/Figure 4/Figure 4E/Huh7/SOR 3 ╬╝M+LY294002 0 ╬╝M.jpg]

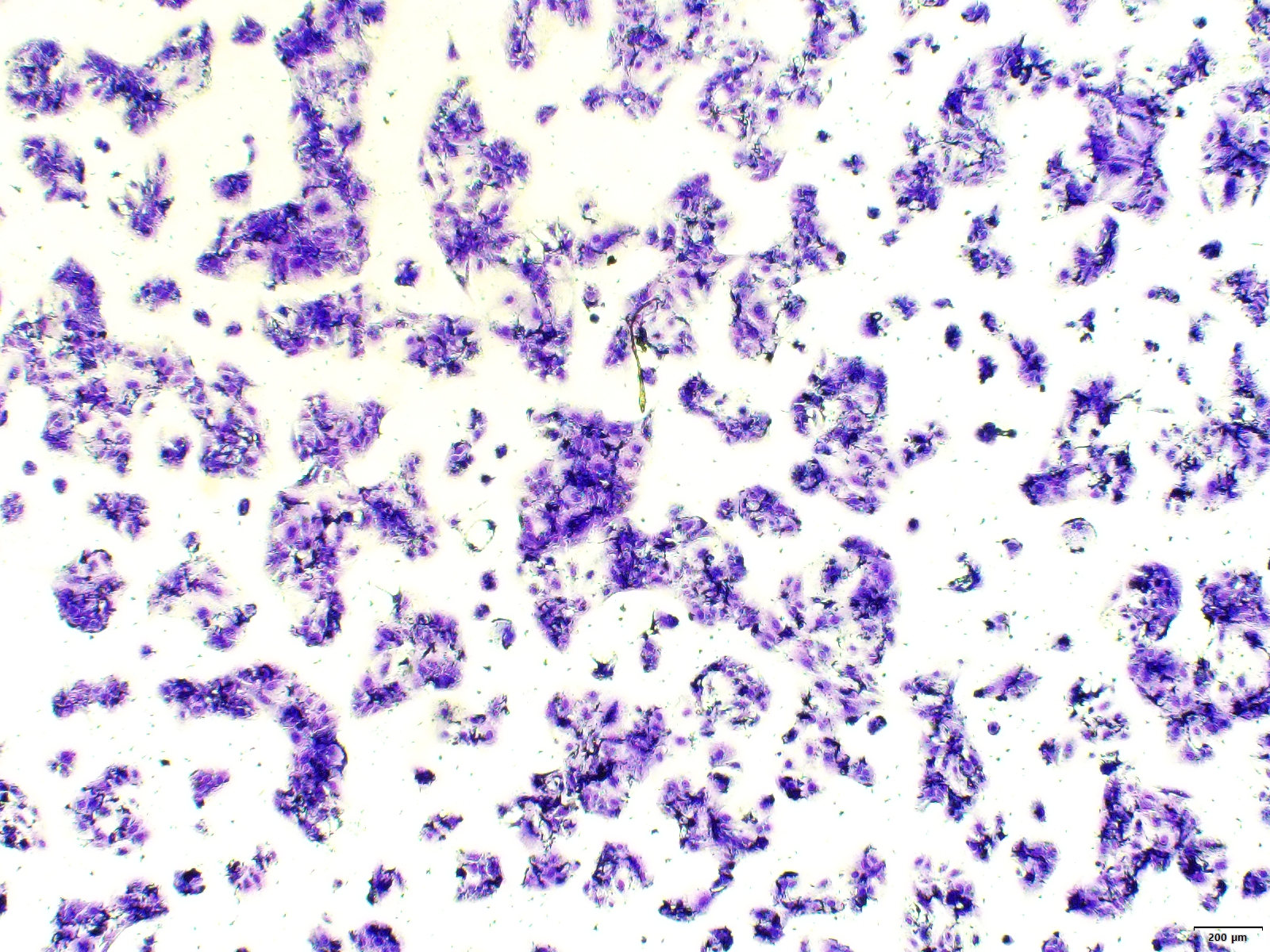

Supplement: Supplementary file 3 — Additional file 2. [file 12964_2023_1355_MOESM2_ESM.zip › raw data/Figure 4/Figure 4E/Huh7/SOR 6 ╬╝M+LY294002 0 ╬╝M.jpg]

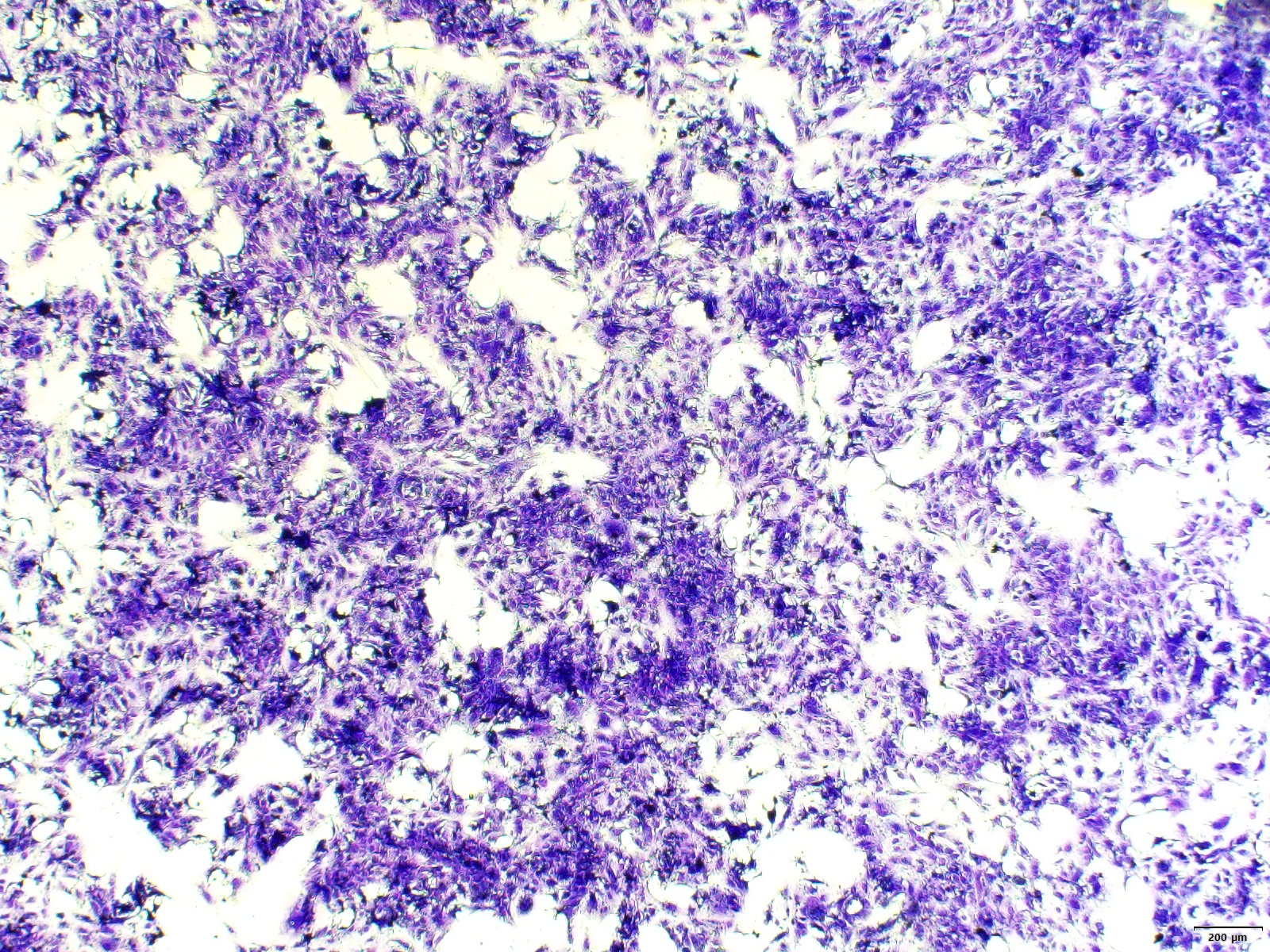

Supplement: Supplementary file 3 — Additional file 2. [file 12964_2023_1355_MOESM2_ESM.zip › raw data/Figure 4/Figure 4E/Huh7/SOR 0 ╬╝M+LY294002 25 ╬╝M.jpg]

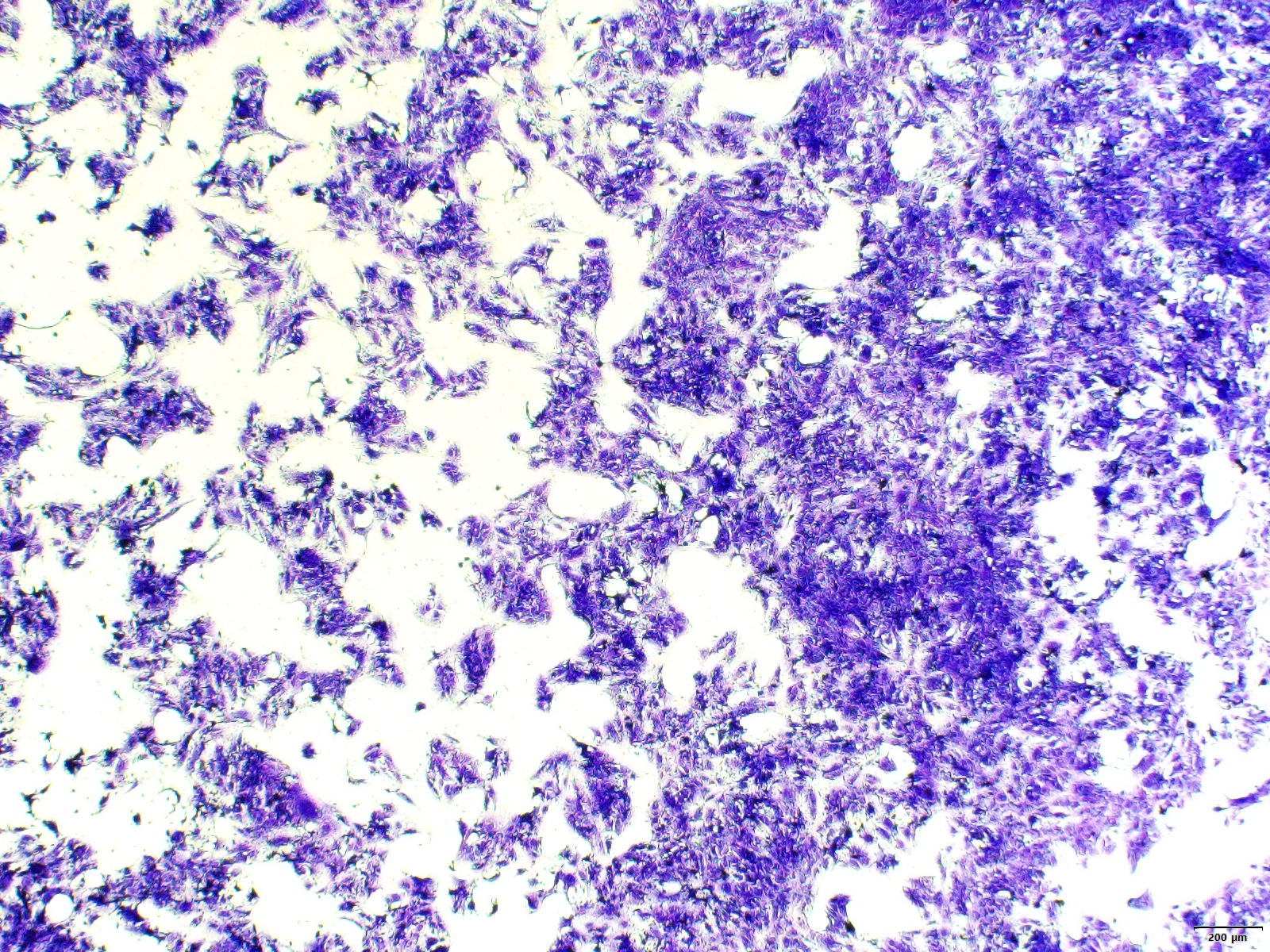

Supplement: Supplementary file 3 — Additional file 2. [file 12964_2023_1355_MOESM2_ESM.zip › raw data/Figure 4/Figure 4E/Huh7/SOR 1.5 ╬╝M+LY294002 25 ╬╝M.jpg]

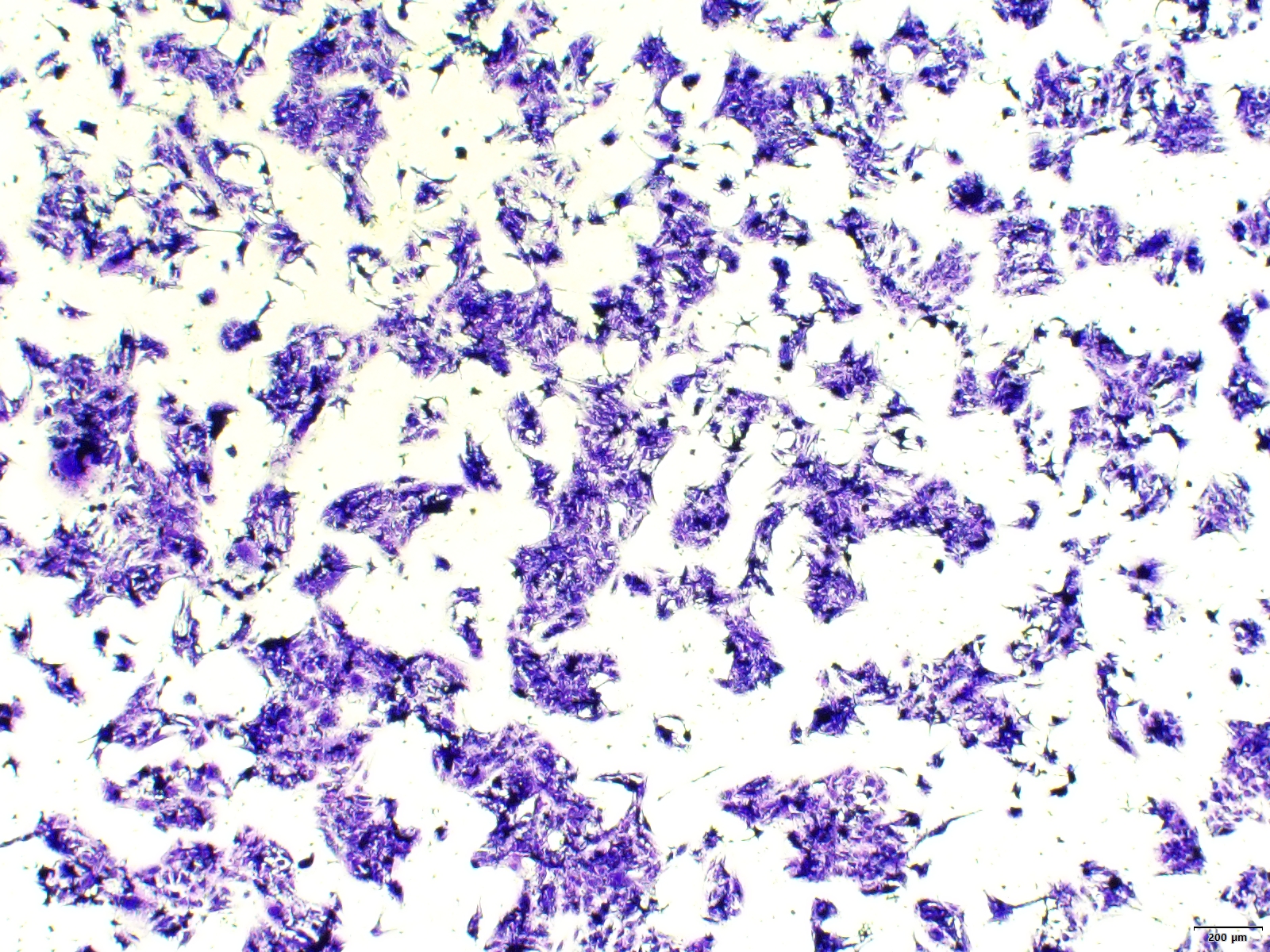

Supplement: Supplementary file 3 — Additional file 2. [file 12964_2023_1355_MOESM2_ESM.zip › raw data/Figure 4/Figure 4E/Huh7/SOR 3 ╬╝M+LY294002 25 ╬╝M.jpg]

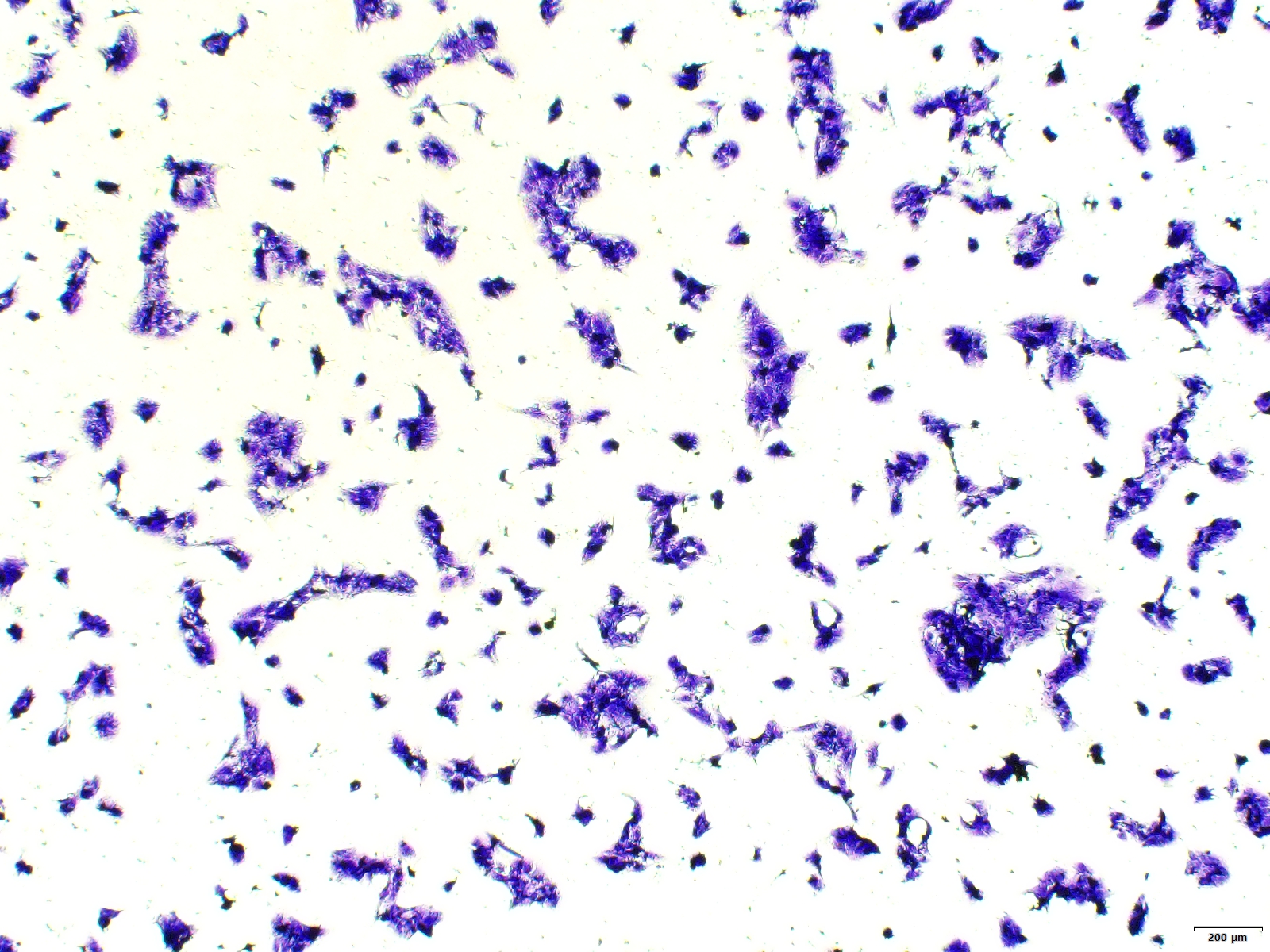

Supplement: Supplementary file 3 — Additional file 2. [file 12964_2023_1355_MOESM2_ESM.zip › raw data/Figure 4/Figure 4E/Huh7/SOR 6 ╬╝M+LY294002 25 ╬╝M.jpg]

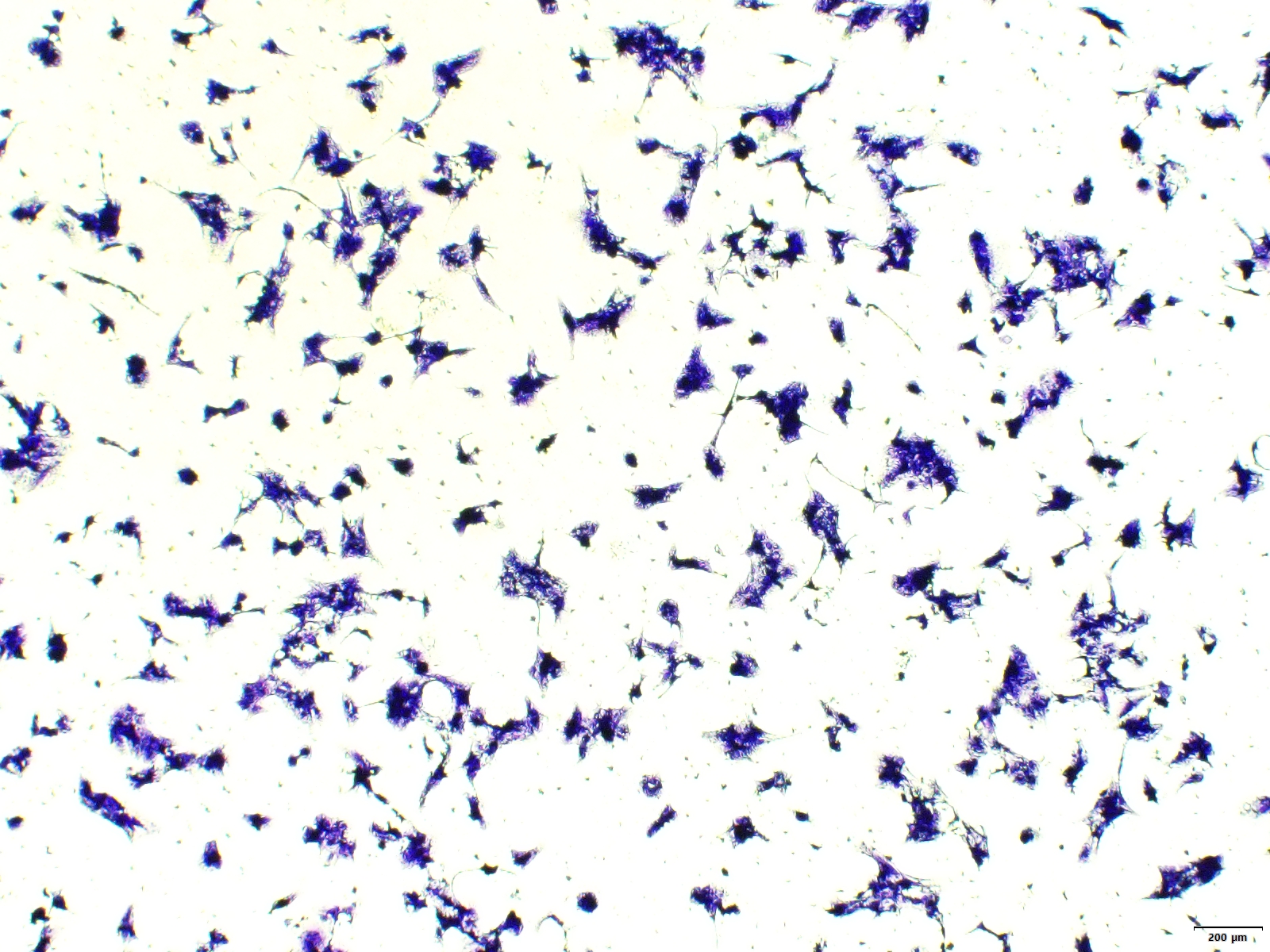

Supplement: Supplementary file 3 — Additional file 2. [file 12964_2023_1355_MOESM2_ESM.zip › raw data/Figure 4/Figure 4E/Huh7/SOR 0 ╬╝M+LY294002 50 ╬╝M.jpg]

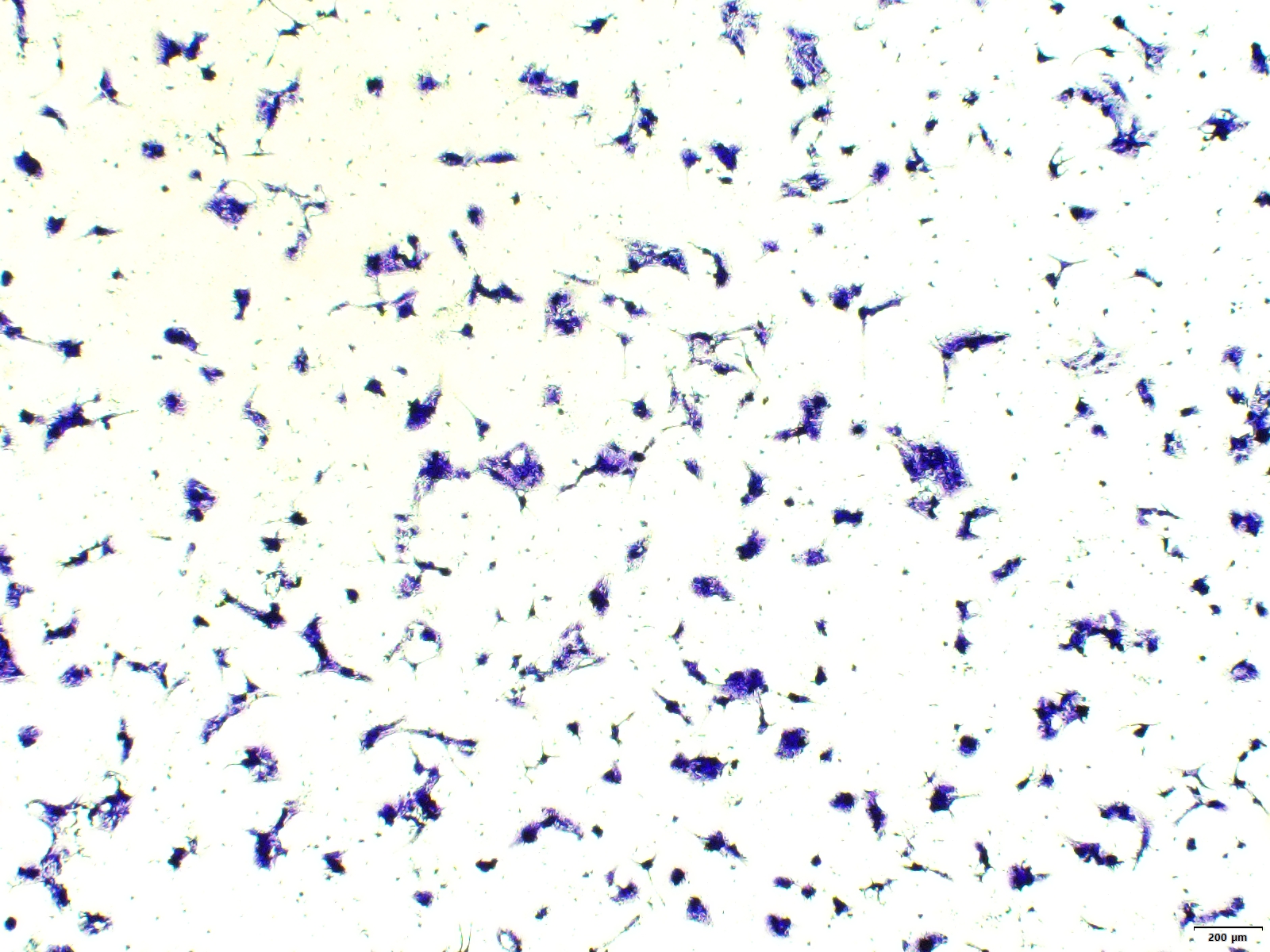

Supplement: Supplementary file 3 — Additional file 2. [file 12964_2023_1355_MOESM2_ESM.zip › raw data/Figure 4/Figure 4E/Huh7/SOR 3 ╬╝M+LY294002 50 ╬╝M.jpg]

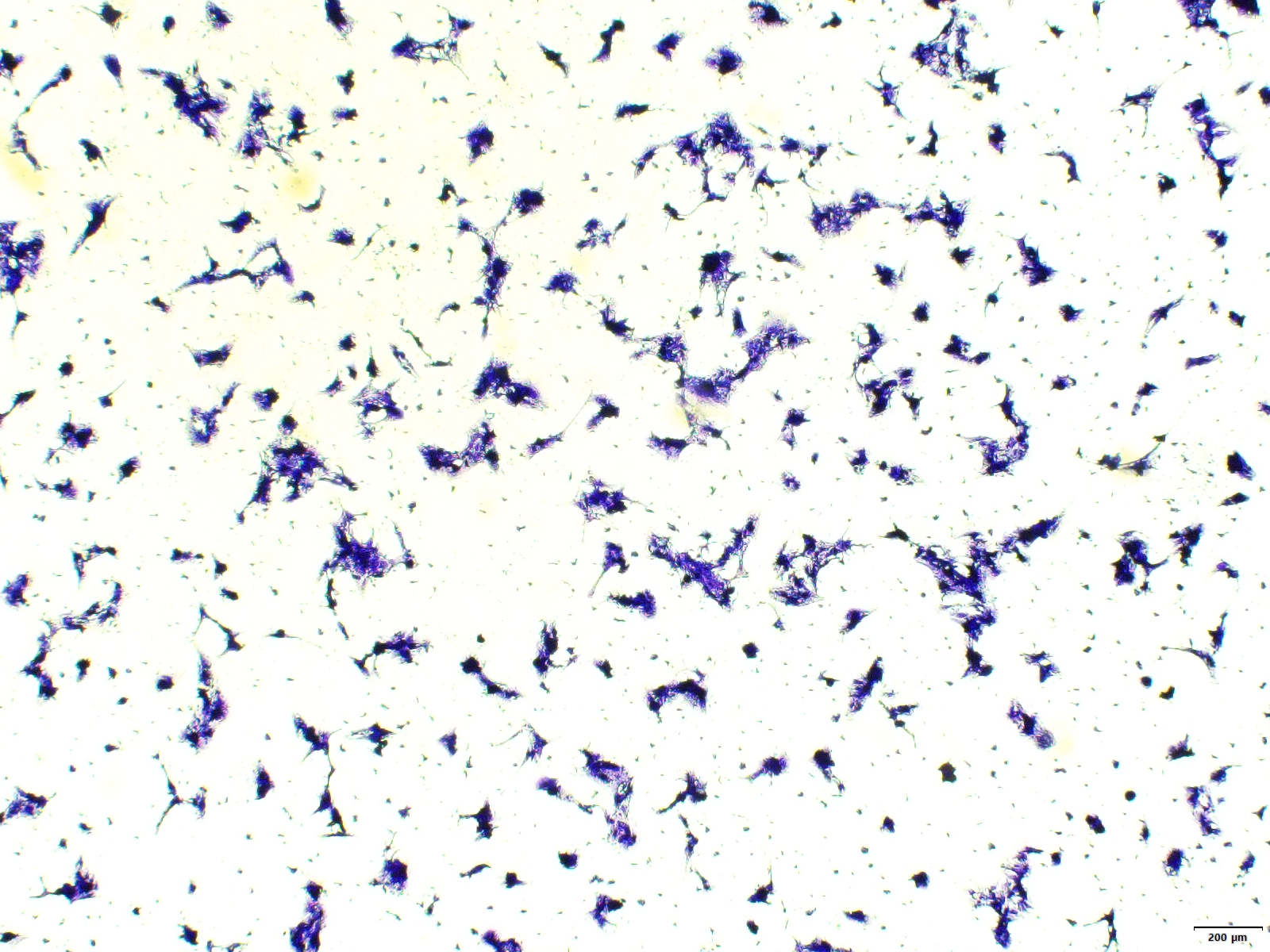

Supplement: Supplementary file 3 — Additional file 2. [file 12964_2023_1355_MOESM2_ESM.zip › raw data/Figure 4/Figure 4E/Huh7/SOR 1.5 ╬╝M+LY294002 50 ╬╝M.jpg]

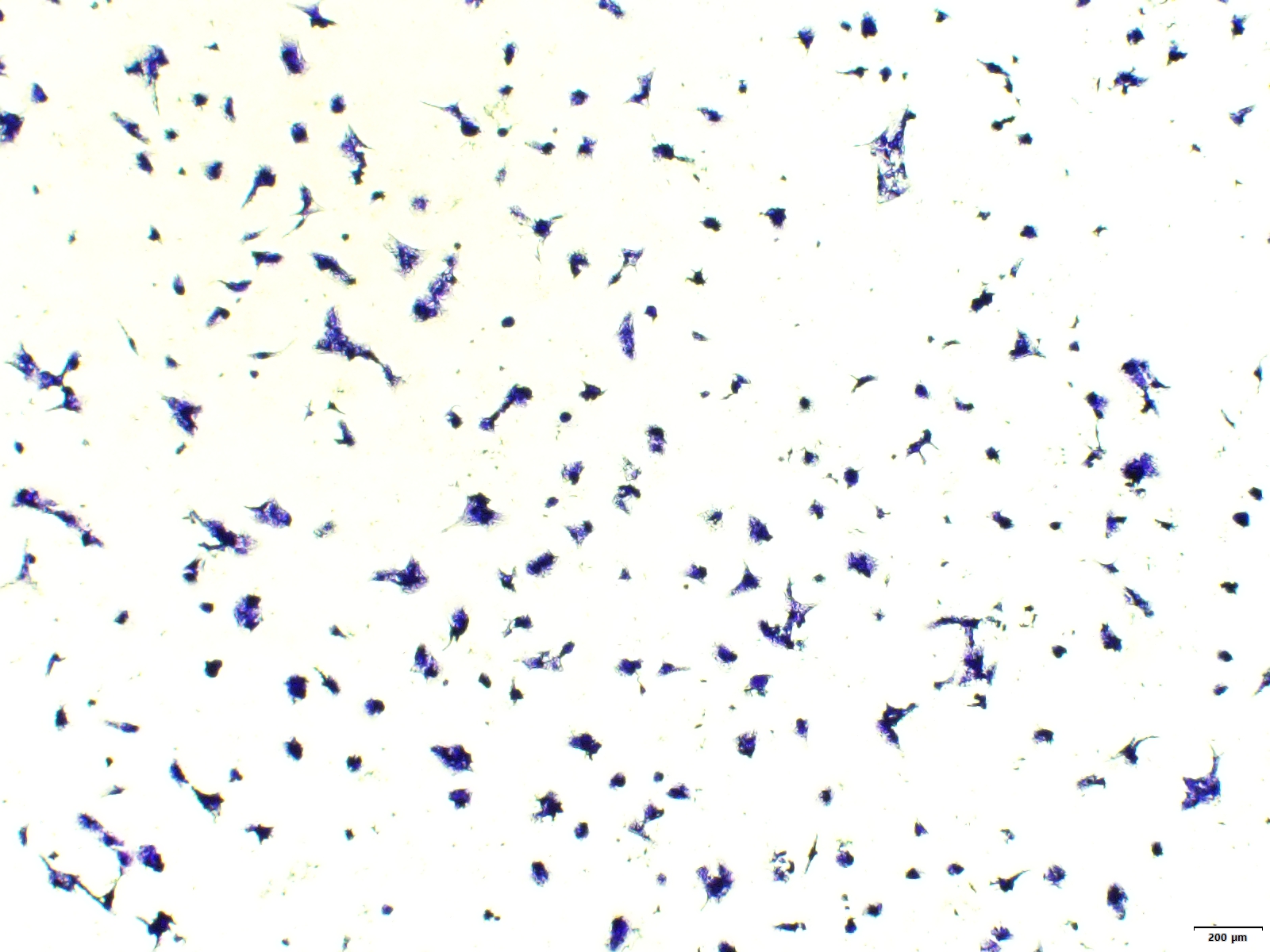

Supplement: Supplementary file 3 — Additional file 2. [file 12964_2023_1355_MOESM2_ESM.zip › raw data/Figure 4/Figure 4E/Huh7/SOR 6 ╬╝M+LY294002 50 ╬╝M.jpg]
