# Supplementary figures and images for: Inhibition of Dickkopf-1 enhances the anti-tumor efficacy of sorafenib via inhibition of the PI3K/Akt and Wnt/β-catenin pathways in hepatocellular carcinoma (part 1 of 2)
Source: Cell Commun Signal. 2023 Nov 27;21:339. doi: 10.1186/s12964-023-01355-2 (PMC10680194; doi:10.1186/s12964-023-01355-2)

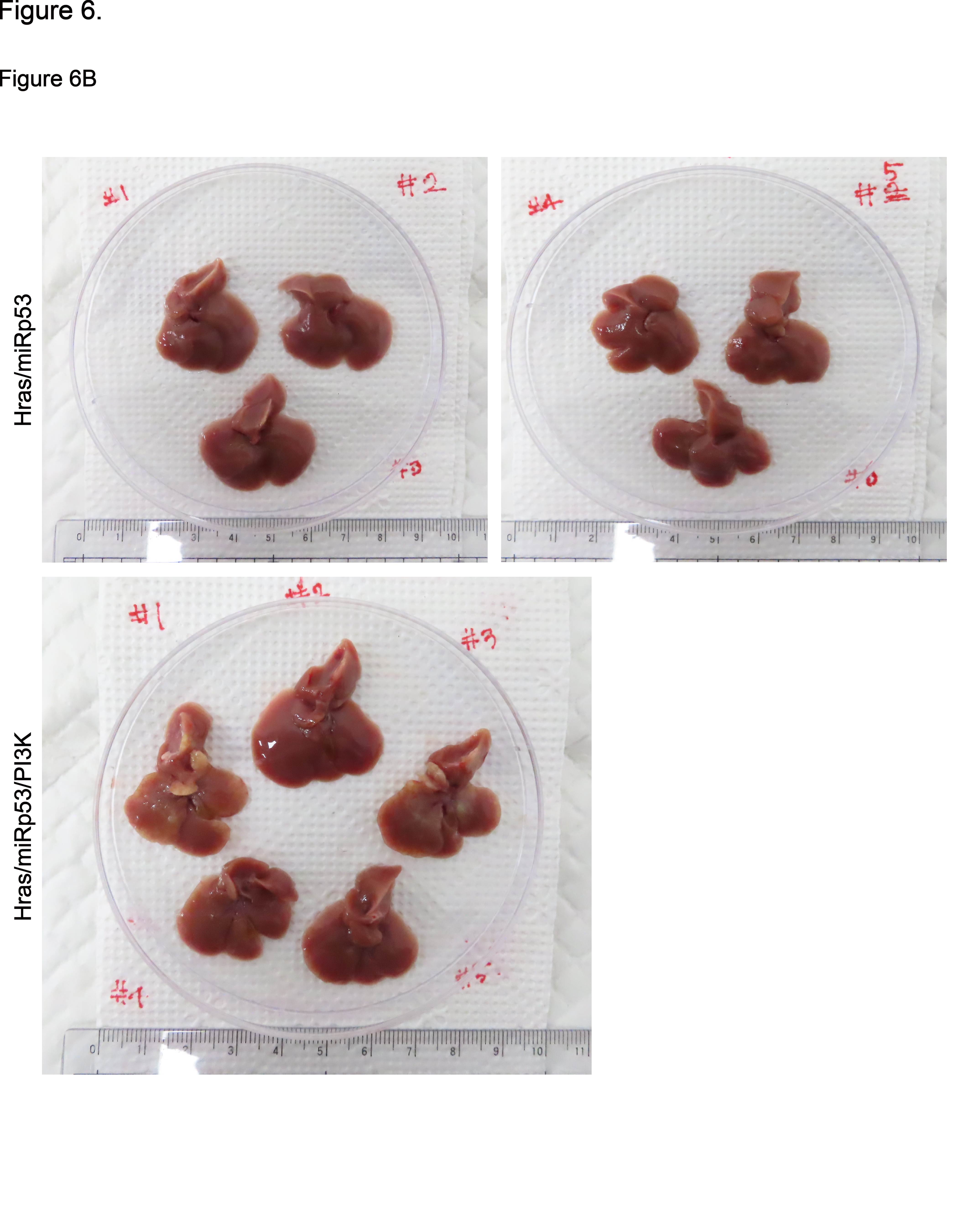

Supplement: Supplementary file 3 — Additional file 2. [file 12964_2023_1355_MOESM2_ESM.zip › raw data/Figure 6/Figure 6B.jpg]

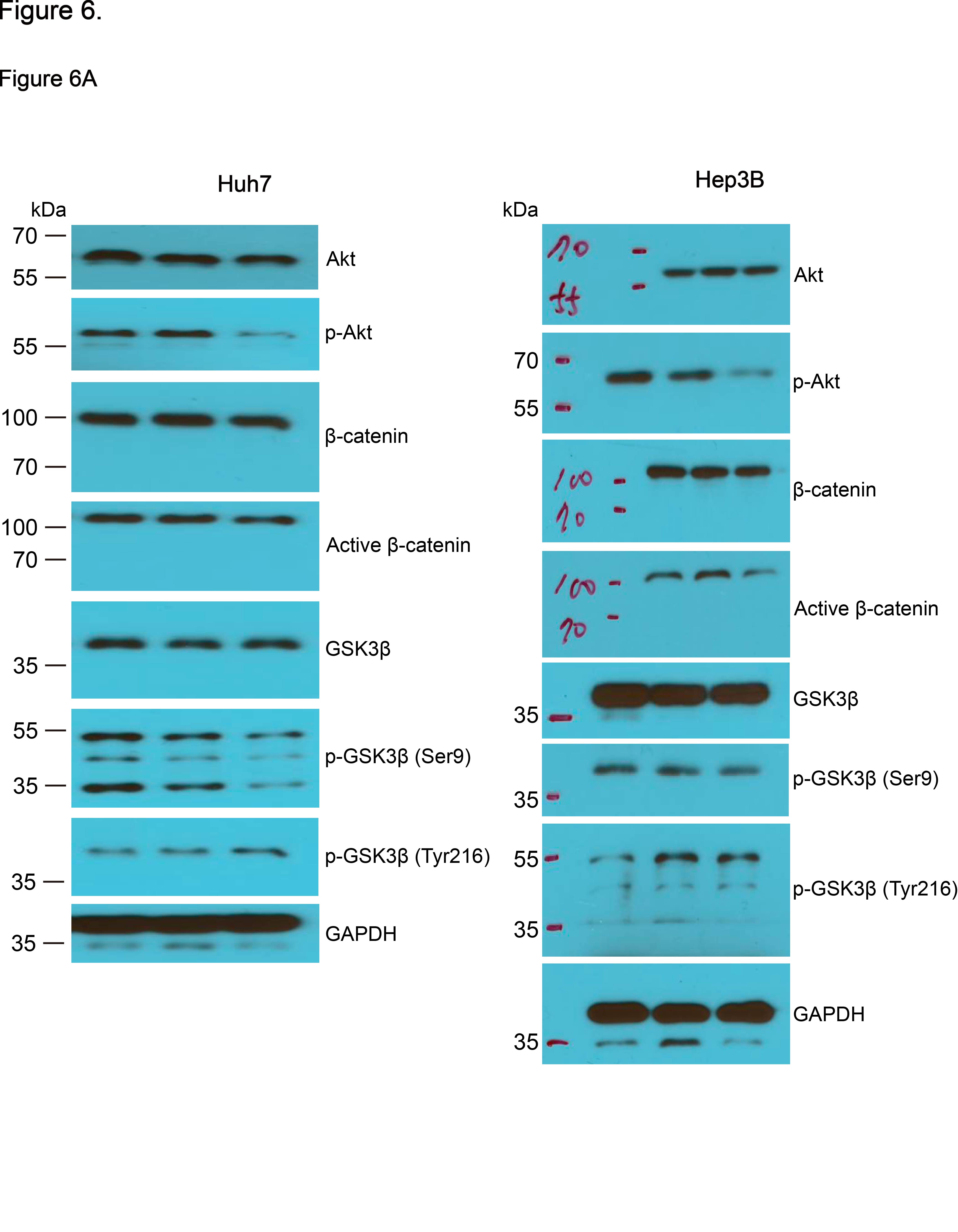

Supplement: Supplementary file 3 — Additional file 2. [file 12964_2023_1355_MOESM2_ESM.zip › raw data/Figure 6/Figure 6A.jpg]

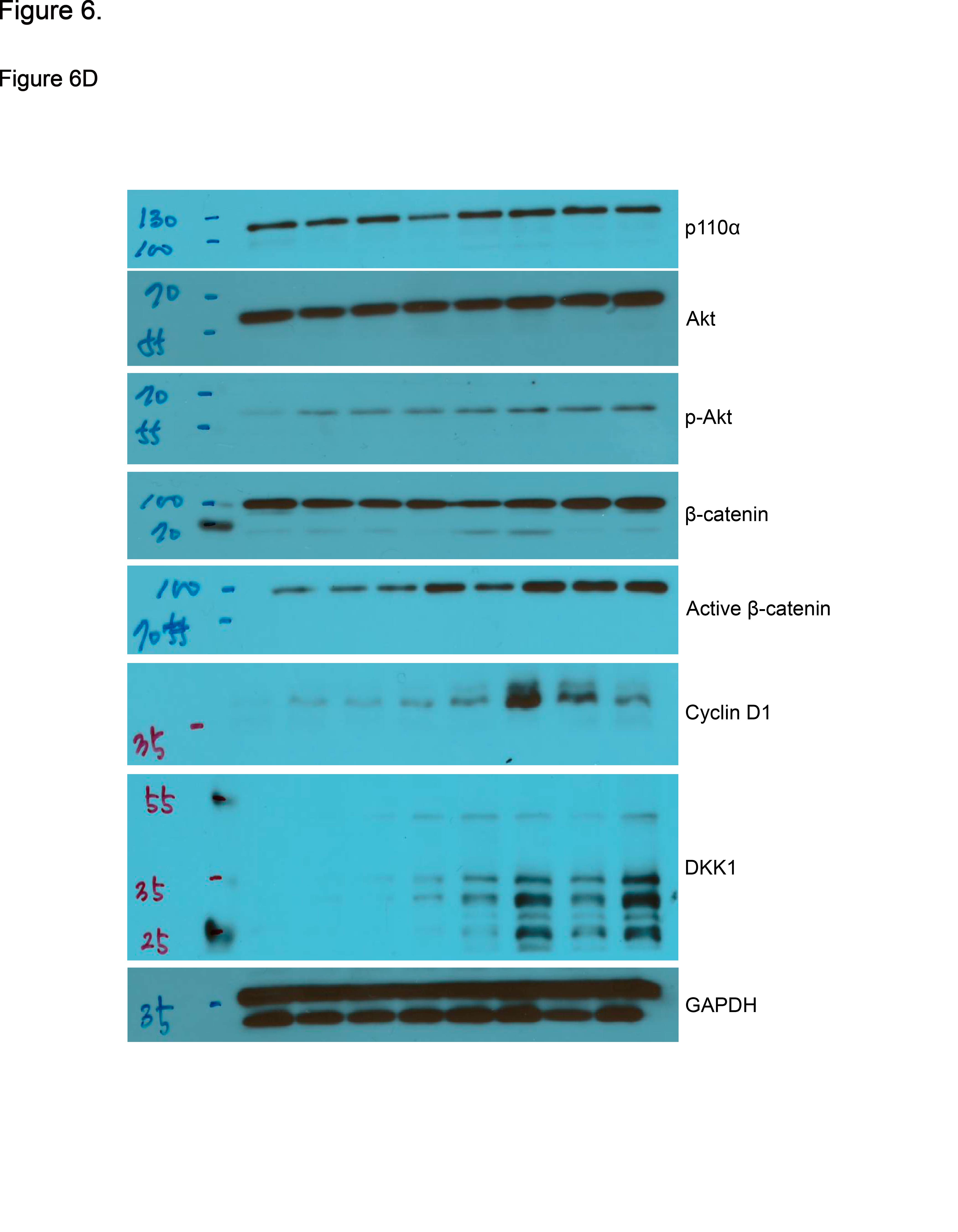

Supplement: Supplementary file 3 — Additional file 2. [file 12964_2023_1355_MOESM2_ESM.zip › raw data/Figure 6/Figure 6D.jpg]

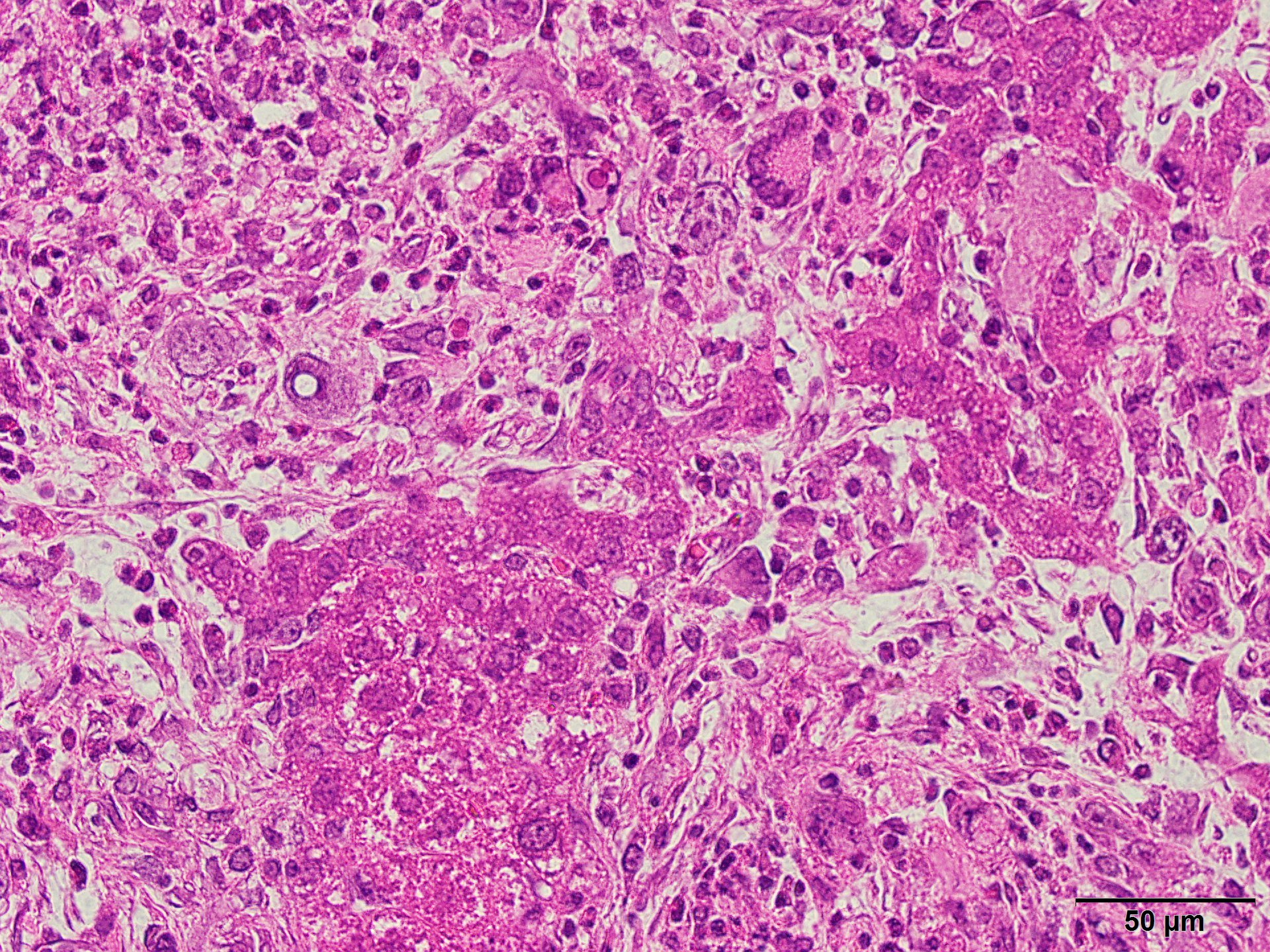

Supplement: Supplementary file 3 — Additional file 2. [file 12964_2023_1355_MOESM2_ESM.zip › raw data/Figure 6/Figure 6E_Hras:miRp53_H&E.jpg]

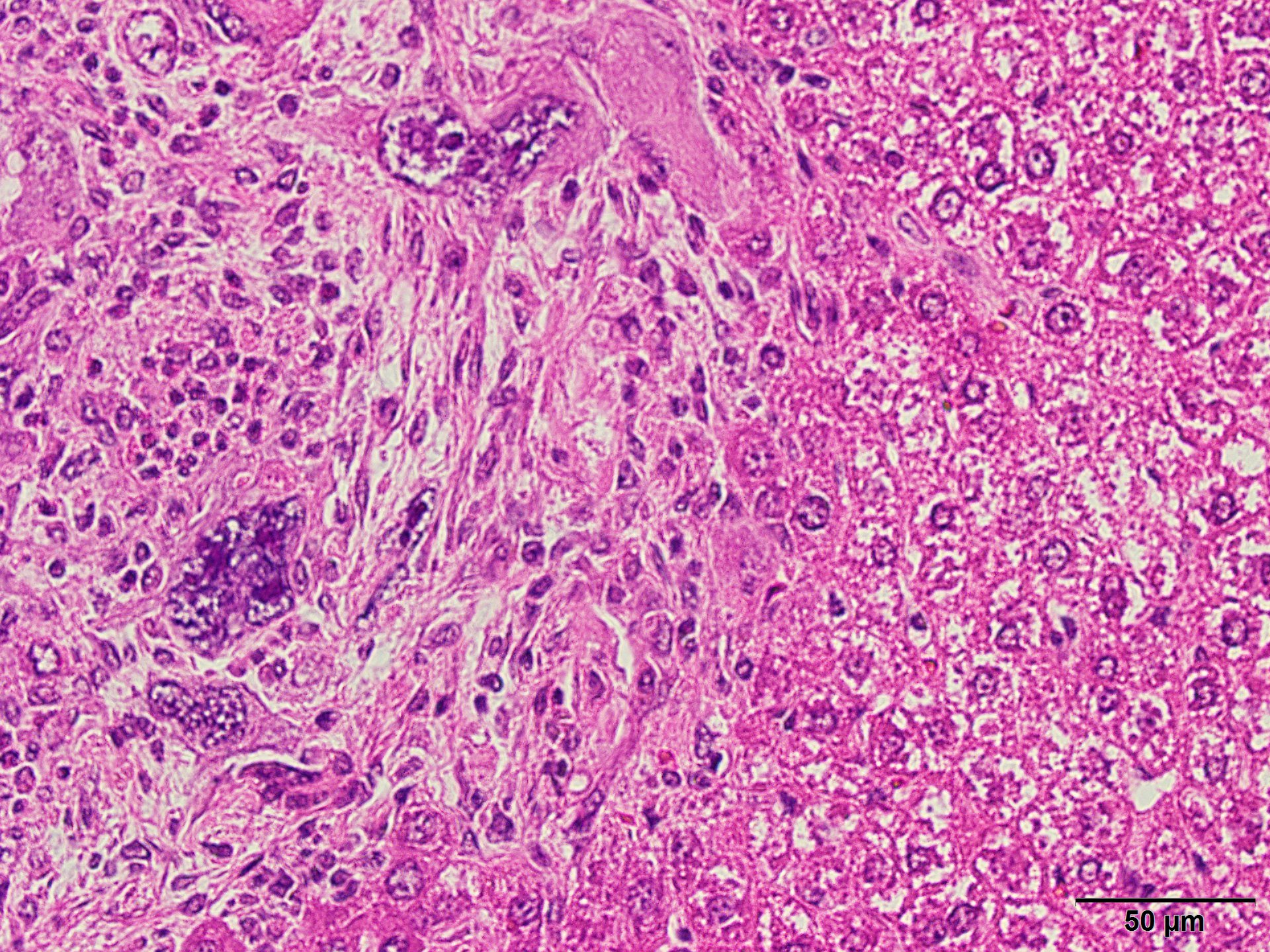

Supplement: Supplementary file 3 — Additional file 2. [file 12964_2023_1355_MOESM2_ESM.zip › raw data/Figure 6/Figure 6E_Hras:miRp53 + PI3K_H&E.jpg]

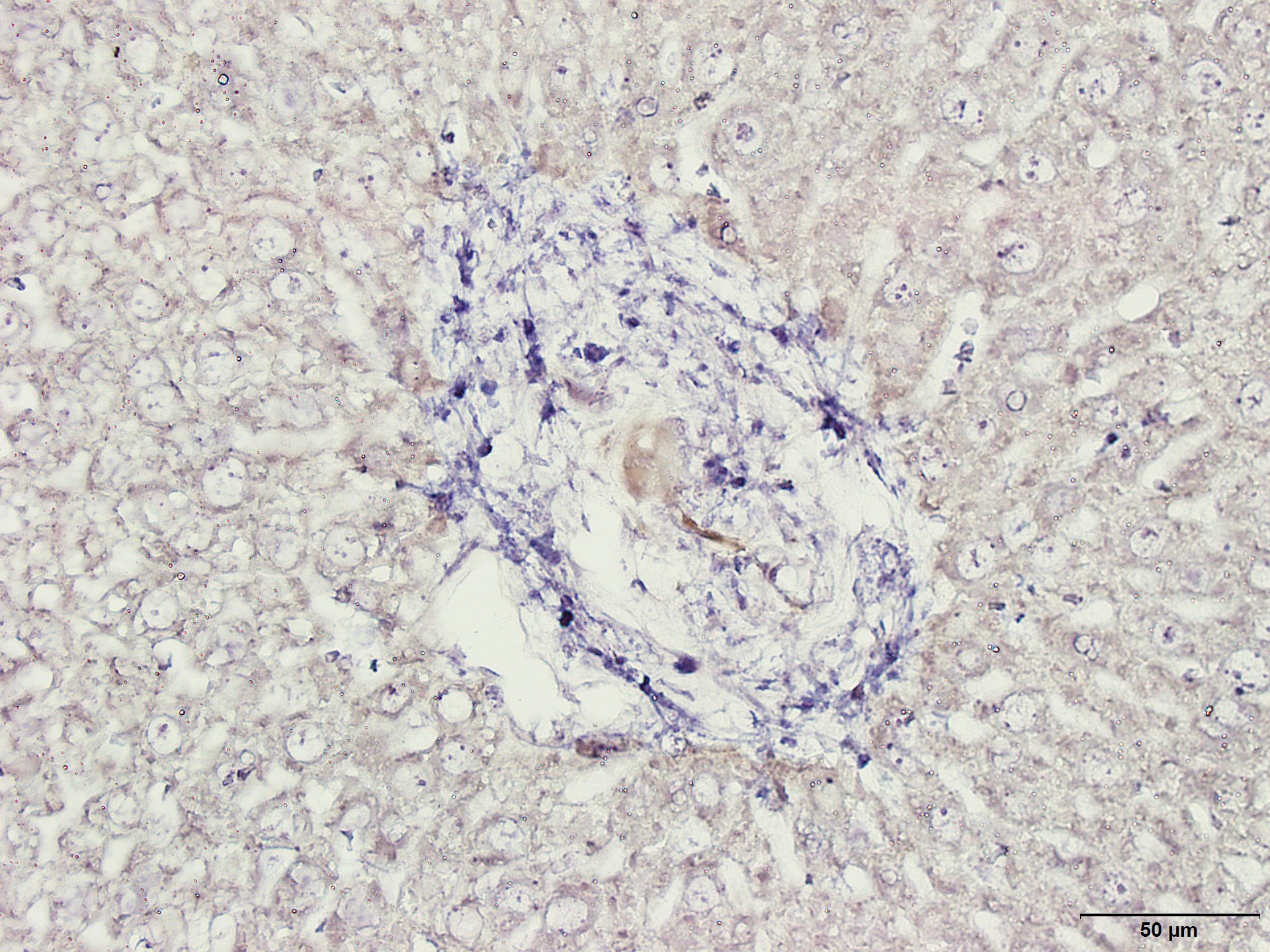

Supplement: Supplementary file 3 — Additional file 2. [file 12964_2023_1355_MOESM2_ESM.zip › raw data/Figure 6/Figure 6E_Hras:miRp53_p-GSK3╬▓ (Ser9).jpg]

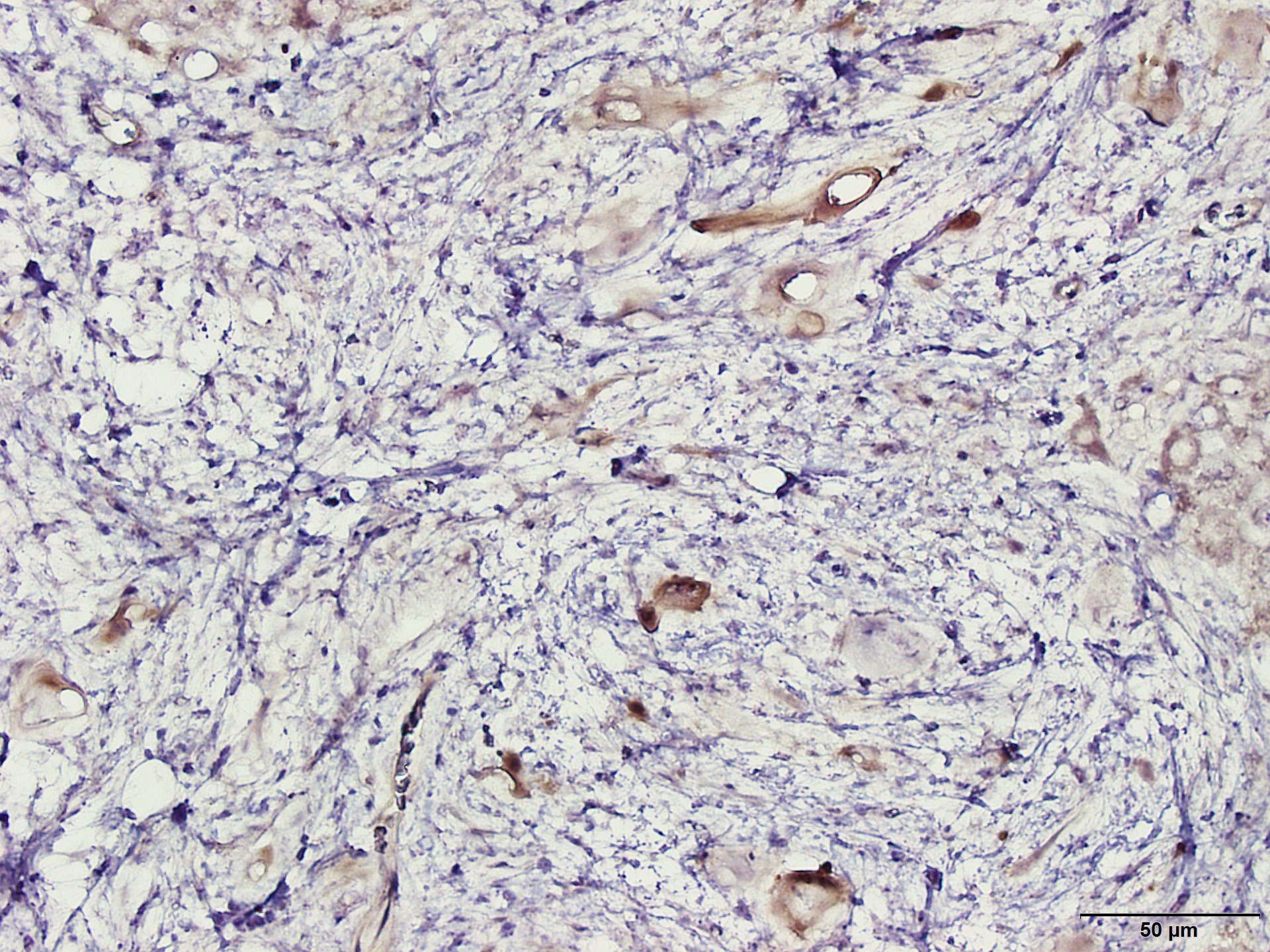

Supplement: Supplementary file 3 — Additional file 2. [file 12964_2023_1355_MOESM2_ESM.zip › raw data/Figure 6/Figure 6E_Hras:miRp53 + PI3K_p-GSK3╬▓ (Ser9).jpg]

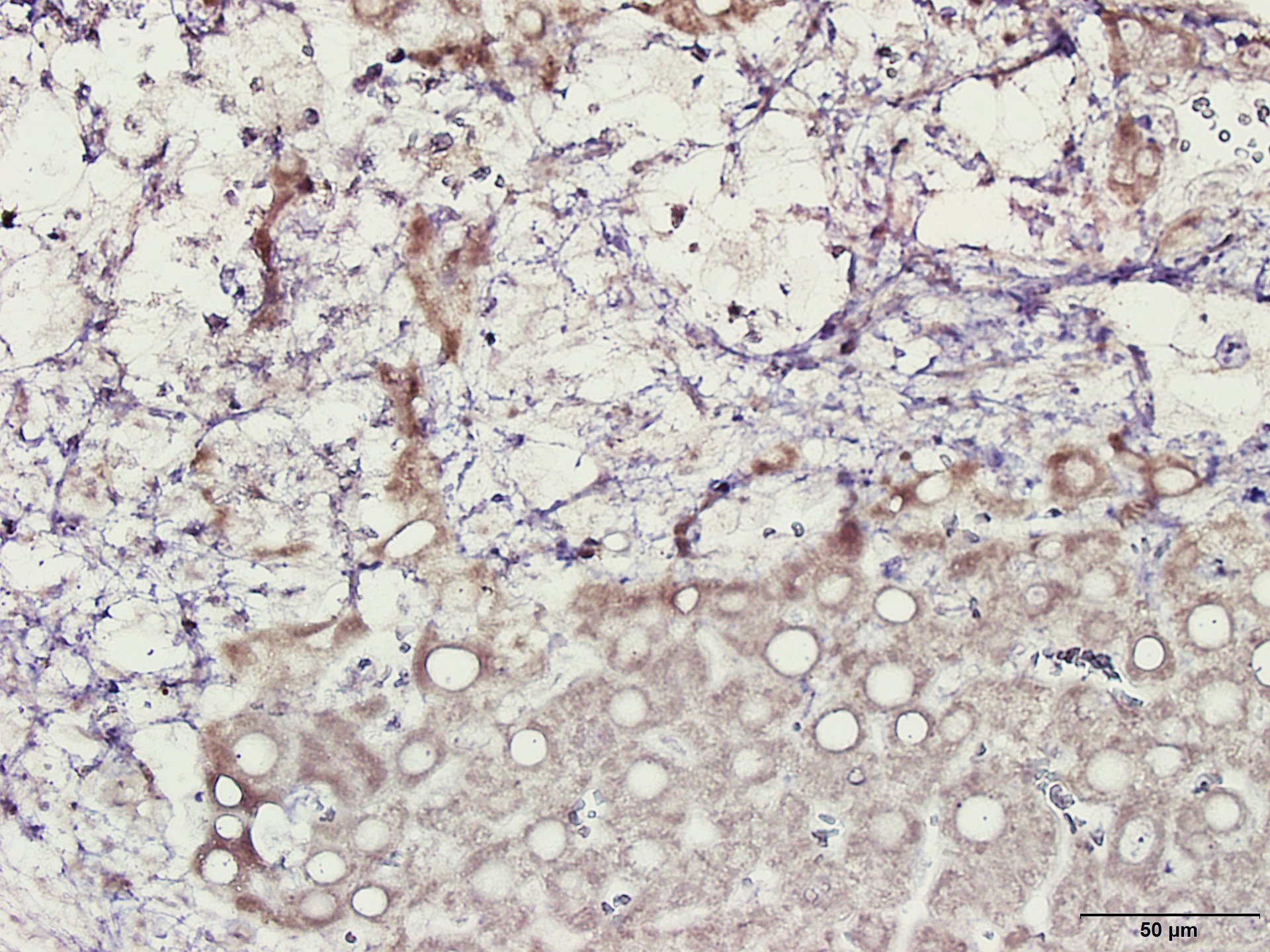

Supplement: Supplementary file 3 — Additional file 2. [file 12964_2023_1355_MOESM2_ESM.zip › raw data/Figure 6/Figure 6E_Hras:miRp53_p-GSK3╬▓ (Tyr216).jpg]

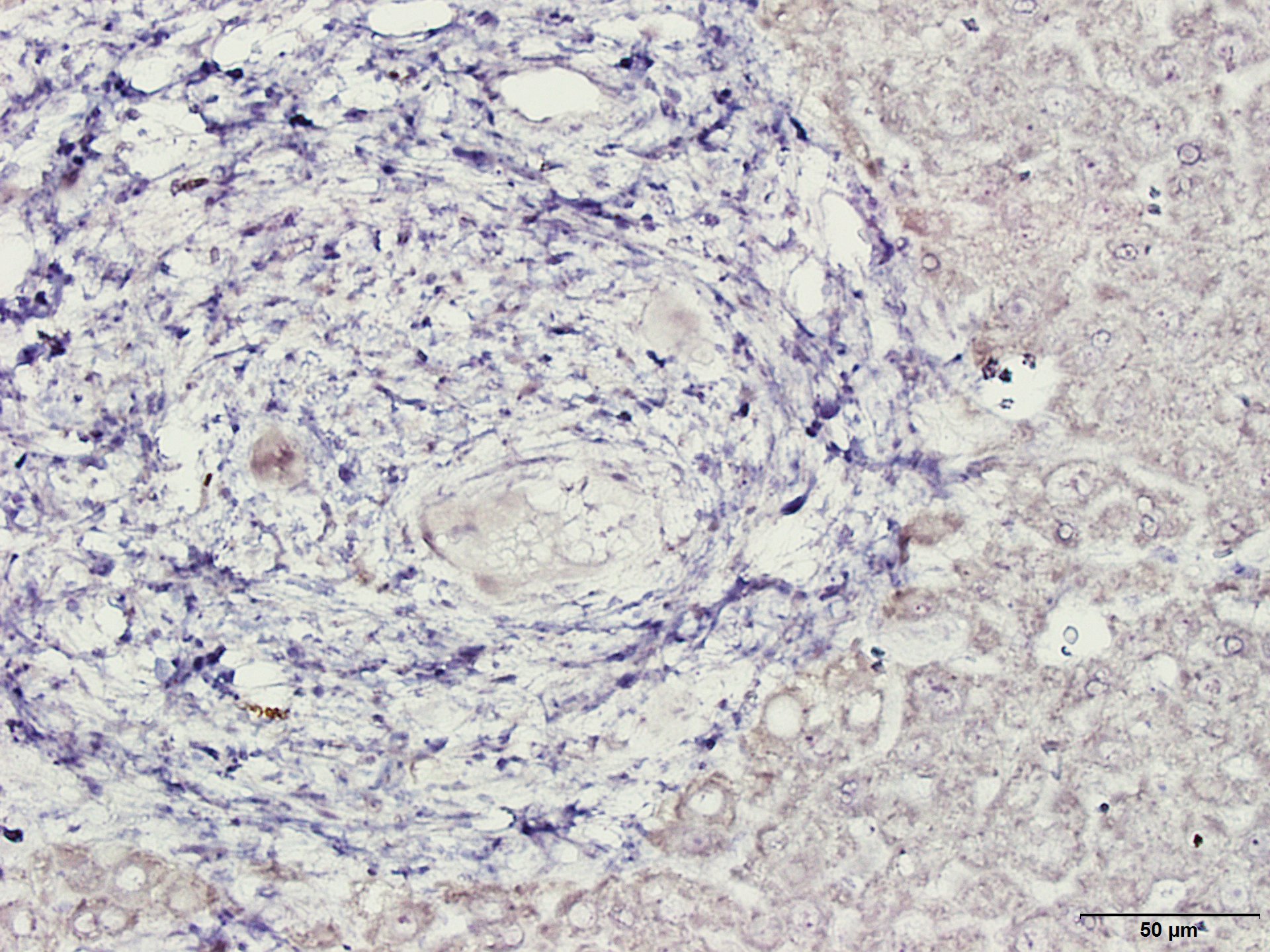

Supplement: Supplementary file 3 — Additional file 2. [file 12964_2023_1355_MOESM2_ESM.zip › raw data/Figure 6/Figure 6E_Hras:miRp53 + PI3K_p-GSK3╬▓ (Tyr216).jpg]

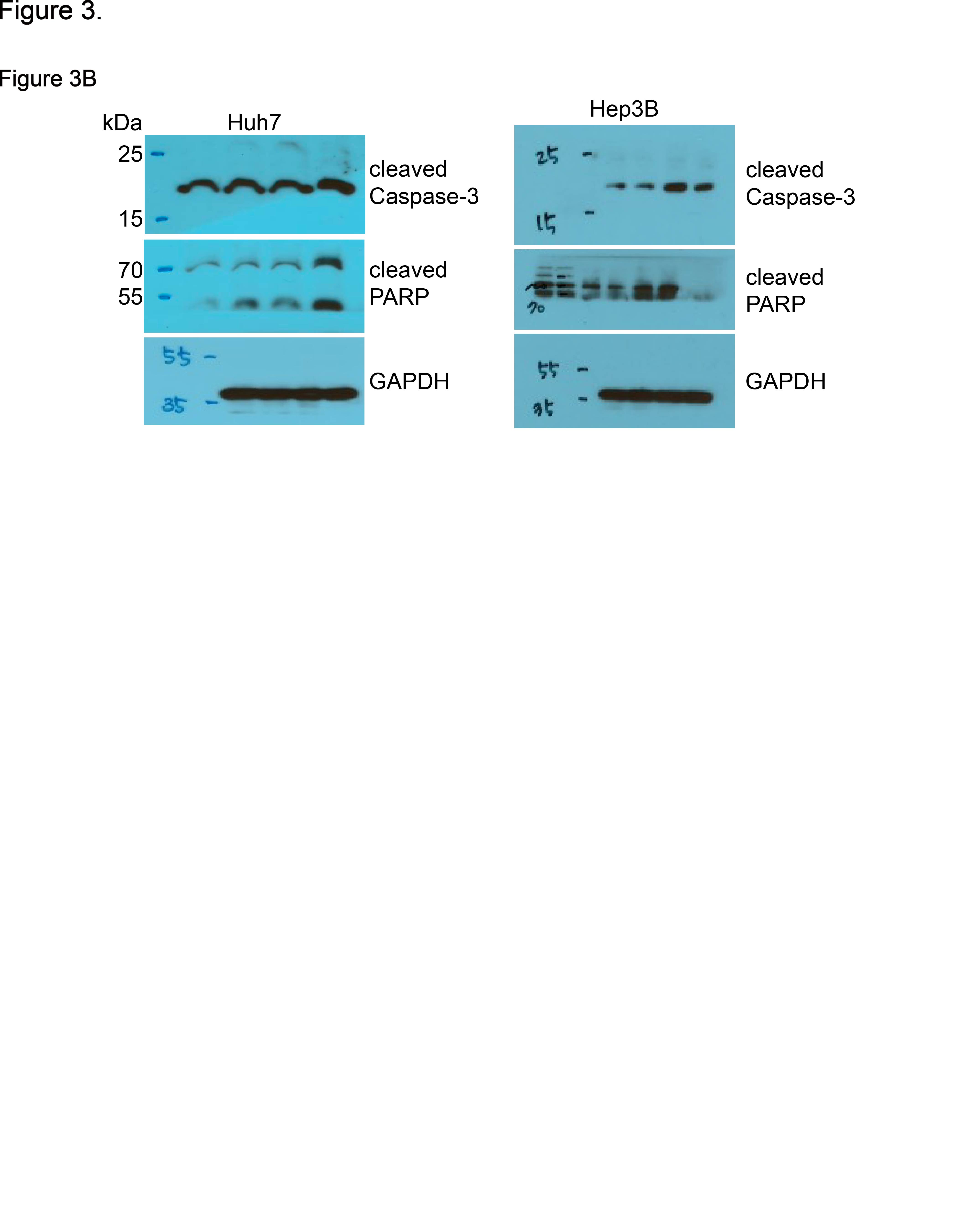

Supplement: Supplementary file 3 — Additional file 2. [file 12964_2023_1355_MOESM2_ESM.zip › raw data/Figure 3/Figure 3B.jpg]

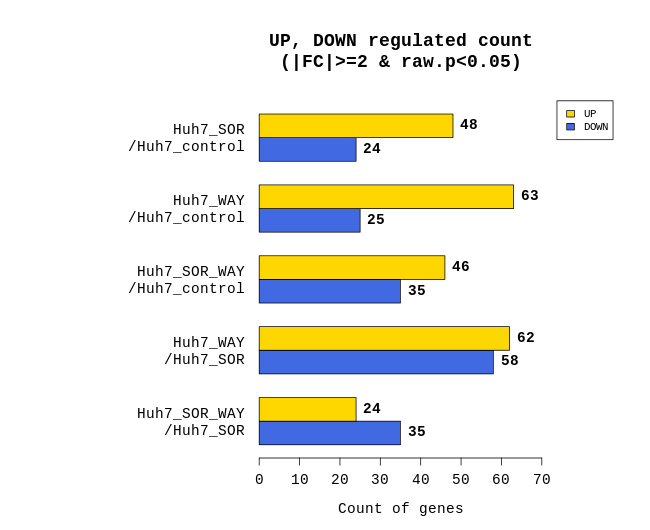

Supplement: Supplementary file 3 — Additional file 2. [file 12964_2023_1355_MOESM2_ESM.zip › raw data/Figure 4/Figure 4A_Huh7_Sig_count_fc_raw.p.png]

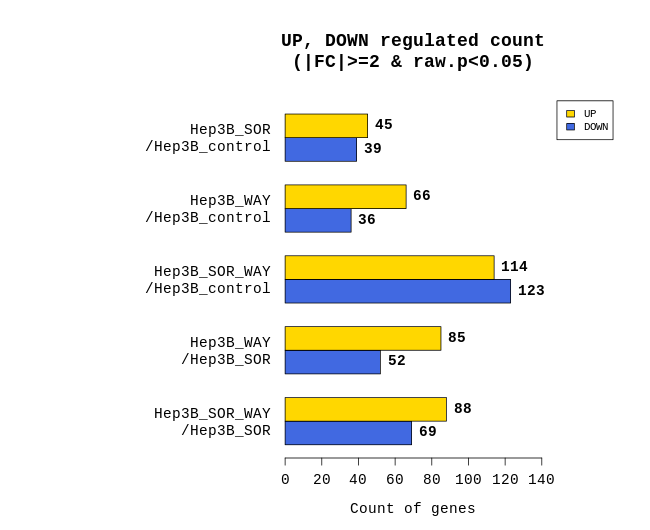

Supplement: Supplementary file 3 — Additional file 2. [file 12964_2023_1355_MOESM2_ESM.zip › raw data/Figure 4/Figure 4A_Hep3B_Sig_count_fc_raw.p.png]

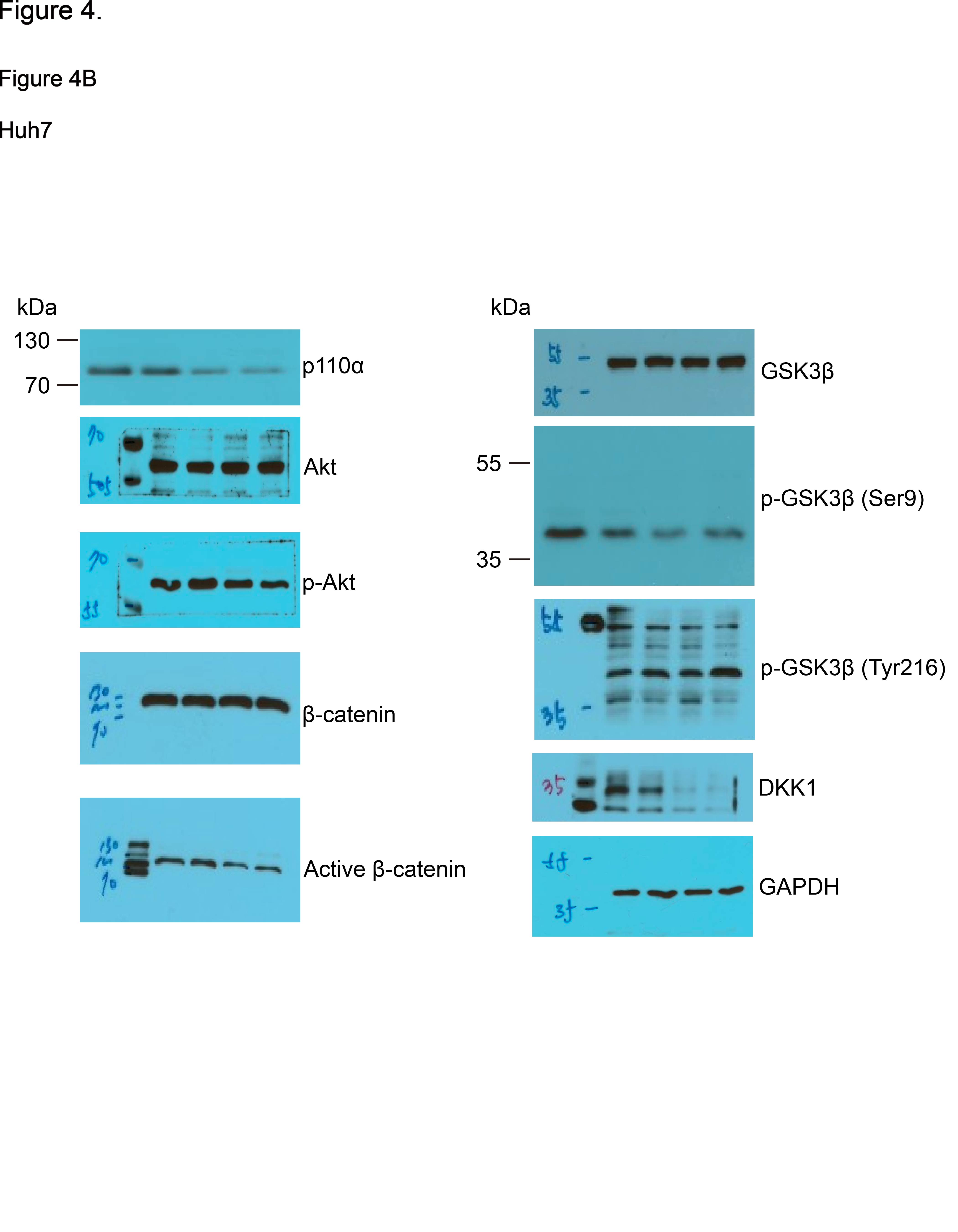

Supplement: Supplementary file 3 — Additional file 2. [file 12964_2023_1355_MOESM2_ESM.zip › raw data/Figure 4/Figure 4B_Huh7.jpg]

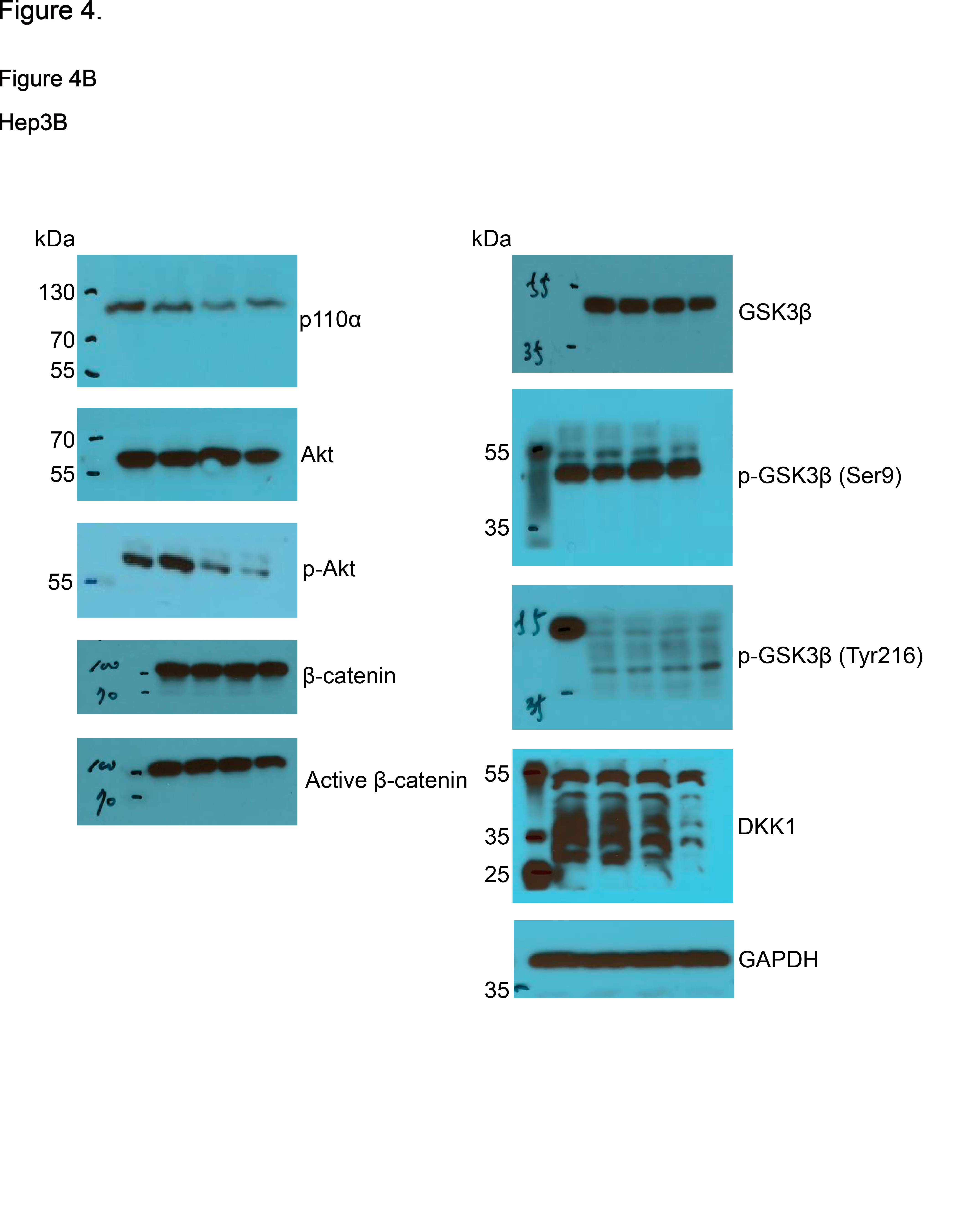

Supplement: Supplementary file 3 — Additional file 2. [file 12964_2023_1355_MOESM2_ESM.zip › raw data/Figure 4/Figure 4B_Hep3B.jpg]

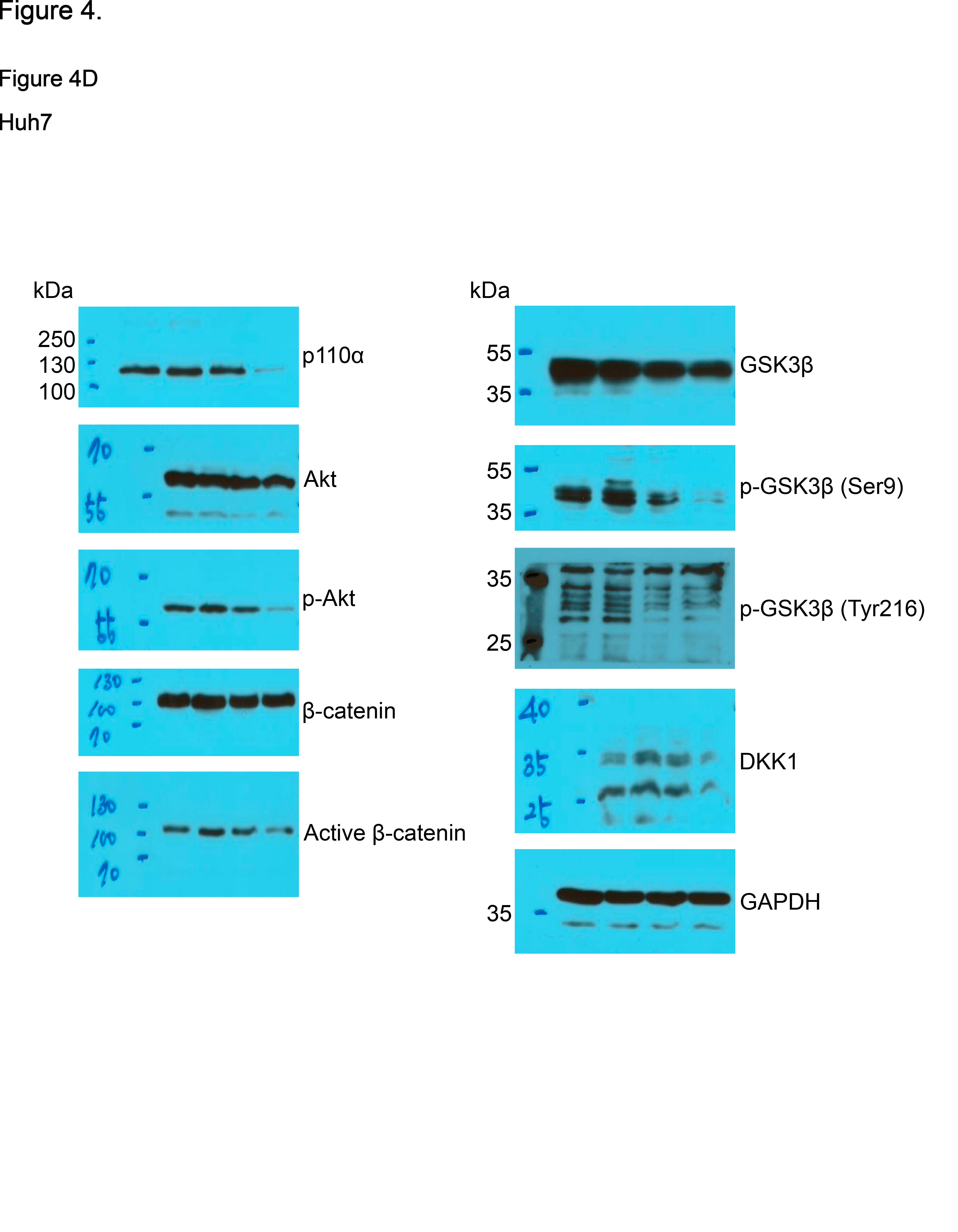

Supplement: Supplementary file 3 — Additional file 2. [file 12964_2023_1355_MOESM2_ESM.zip › raw data/Figure 4/Figure 4D_Huh7.jpg]

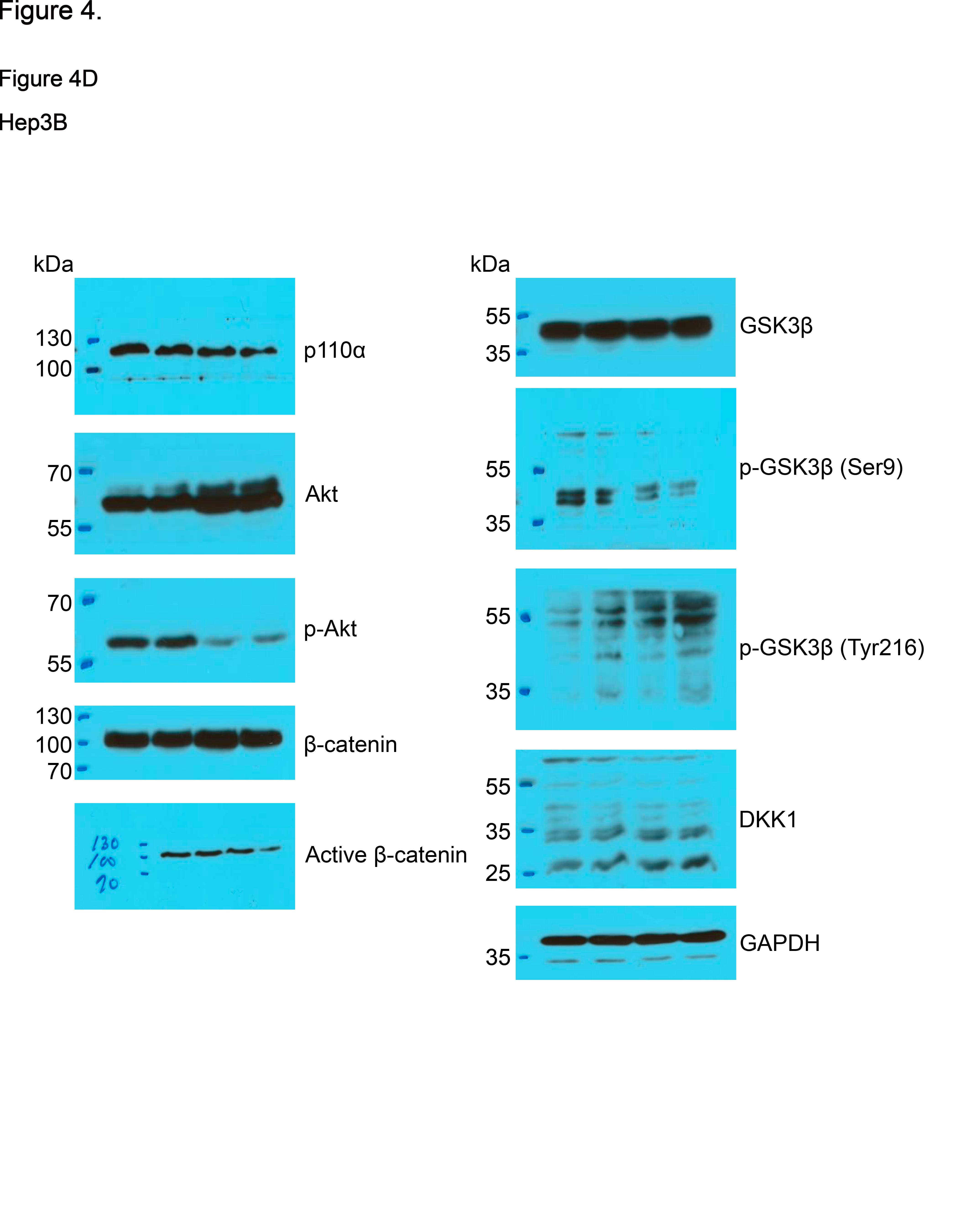

Supplement: Supplementary file 3 — Additional file 2. [file 12964_2023_1355_MOESM2_ESM.zip › raw data/Figure 4/Figure 4D_Hep3B.jpg]

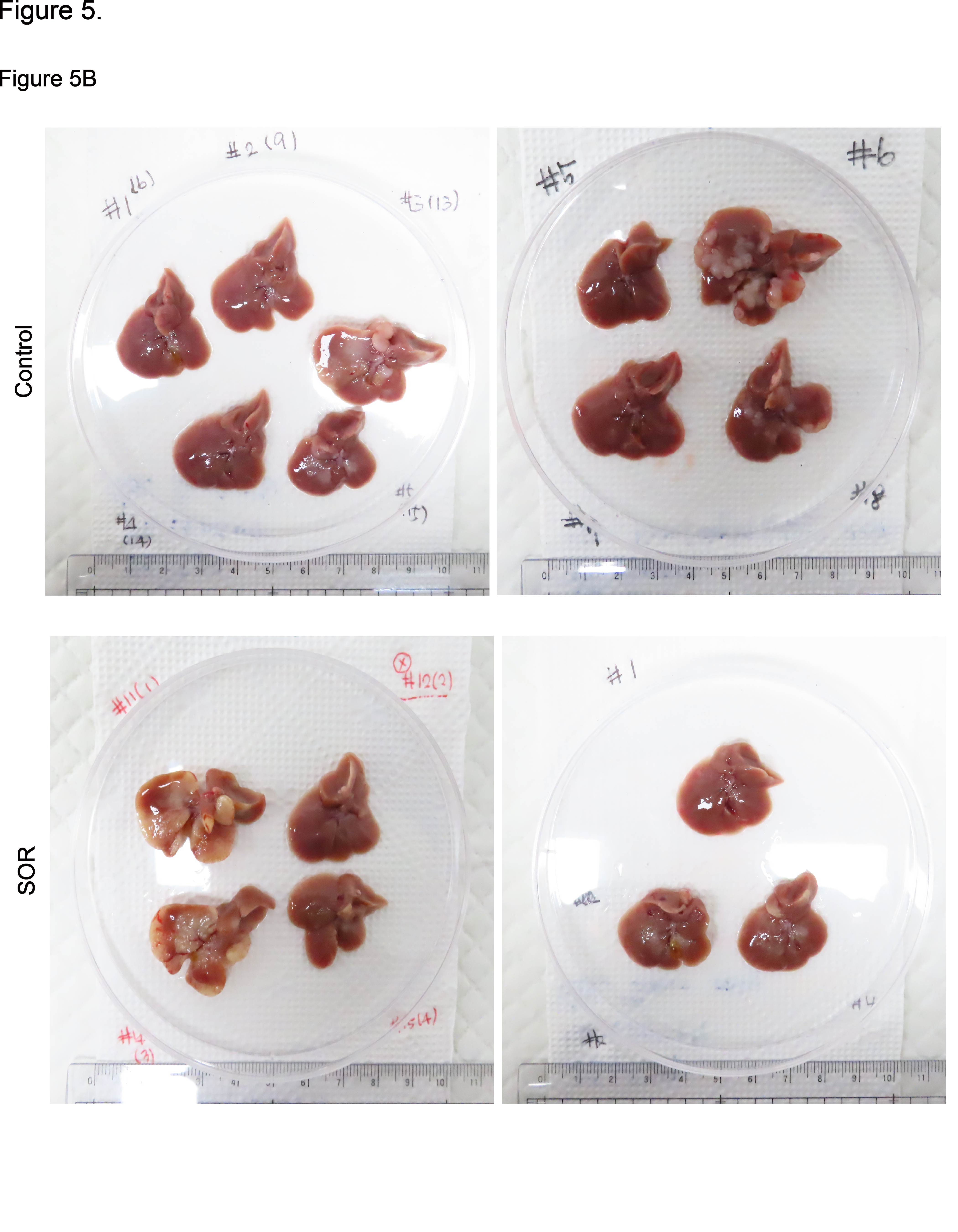

Supplement: Supplementary file 3 — Additional file 2. [file 12964_2023_1355_MOESM2_ESM.zip › raw data/Figure 5/Figure 5B_Control, SOR group.jpg]

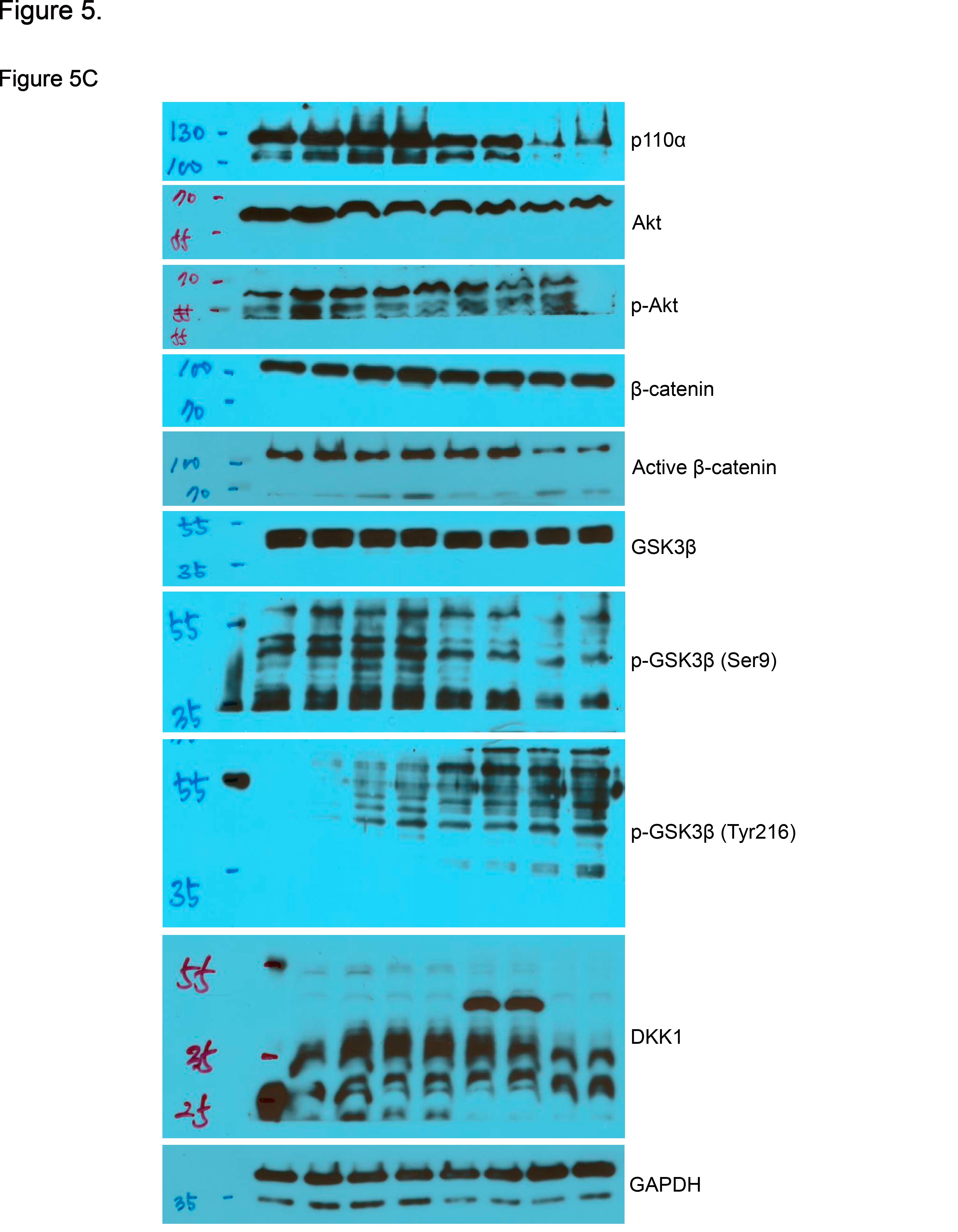

Supplement: Supplementary file 3 — Additional file 2. [file 12964_2023_1355_MOESM2_ESM.zip › raw data/Figure 5/Figure 5C.jpg]

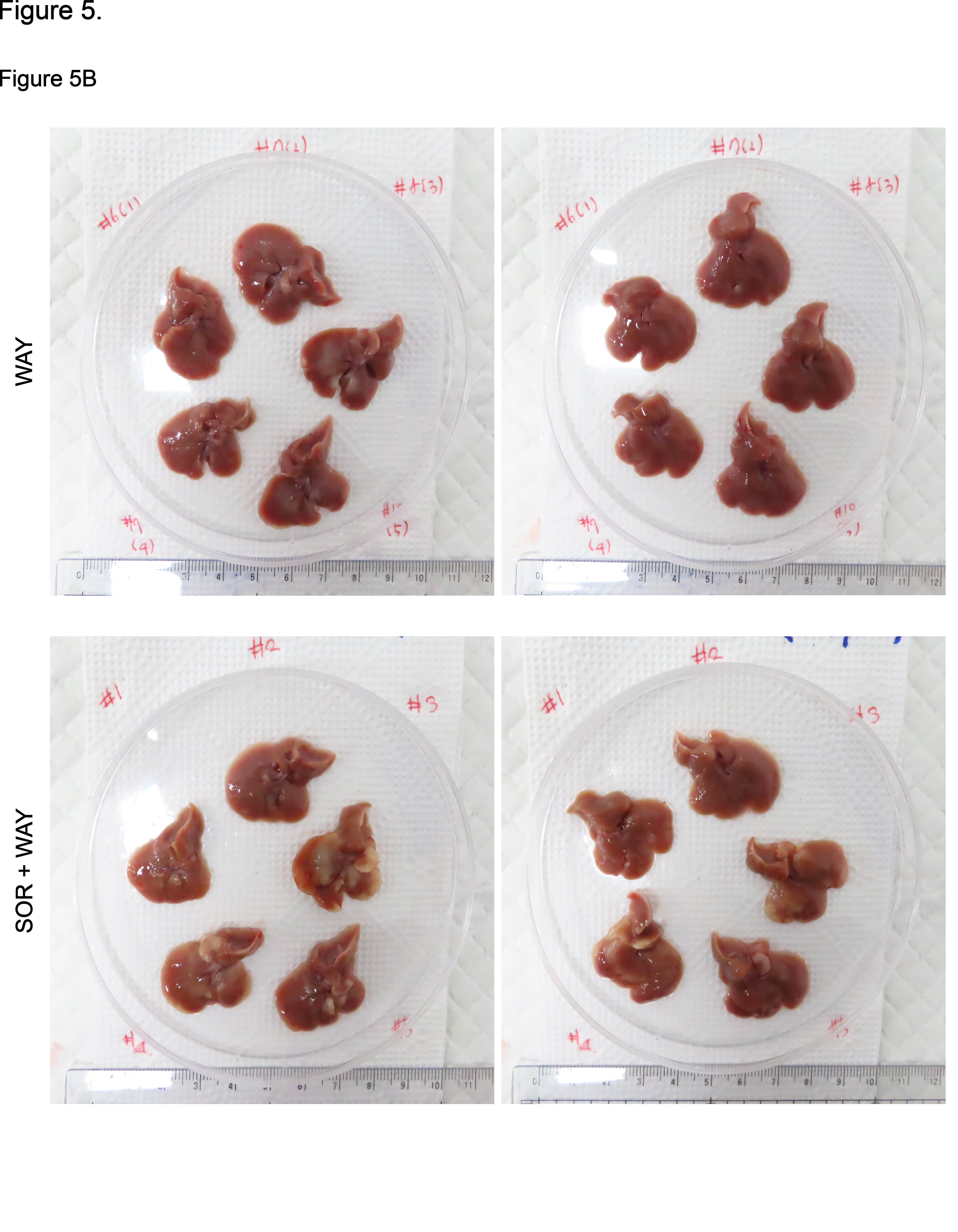

Supplement: Supplementary file 3 — Additional file 2. [file 12964_2023_1355_MOESM2_ESM.zip › raw data/Figure 5/Figure 5B_WAY, SOR + WAY group.jpg]

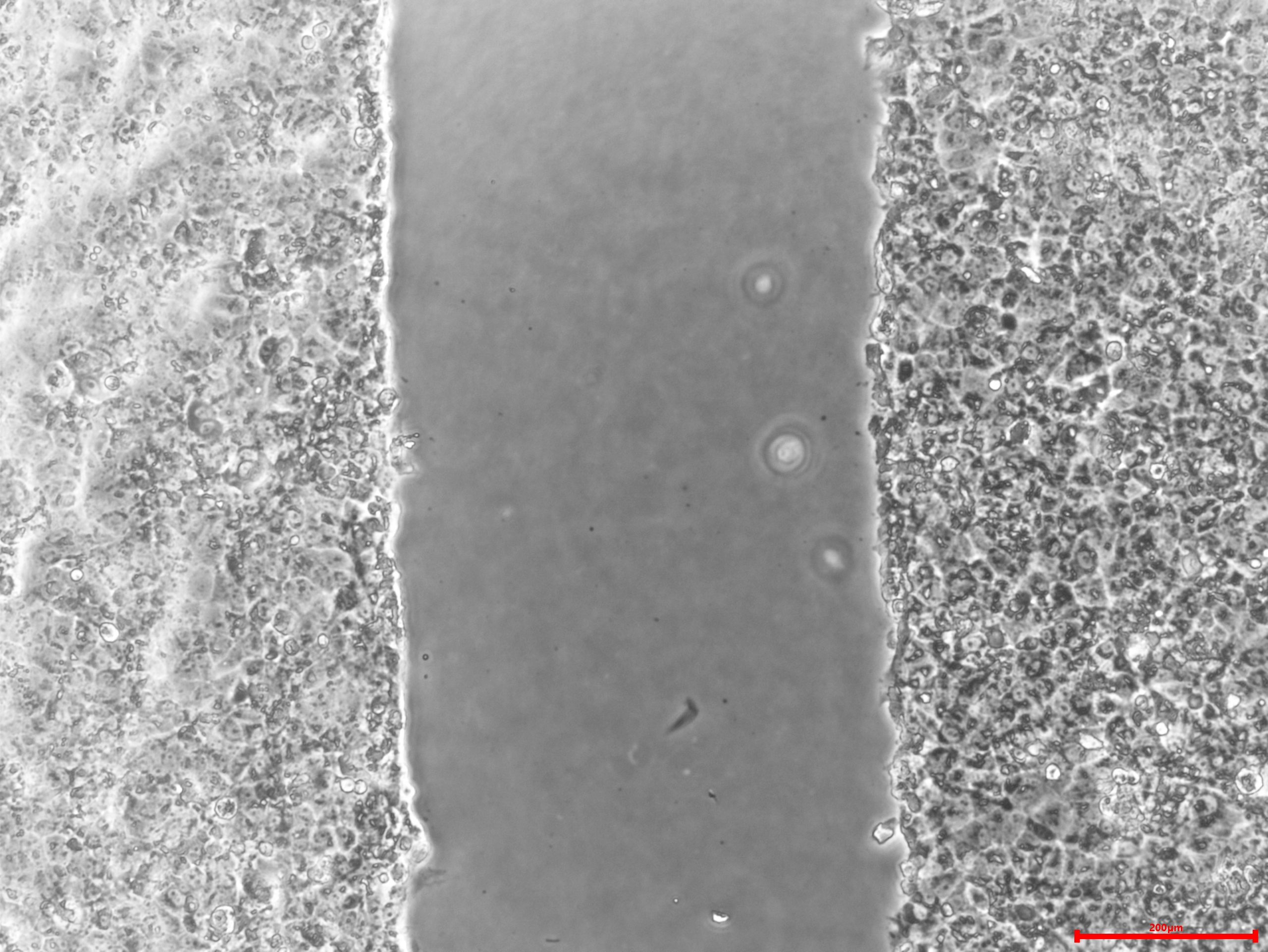

Supplement: Supplementary file 3 — Additional file 2. [file 12964_2023_1355_MOESM2_ESM.zip › raw data/Figure 2/Figure 2D/Figure 2D_Huh7_SOR_0 h.jpg]

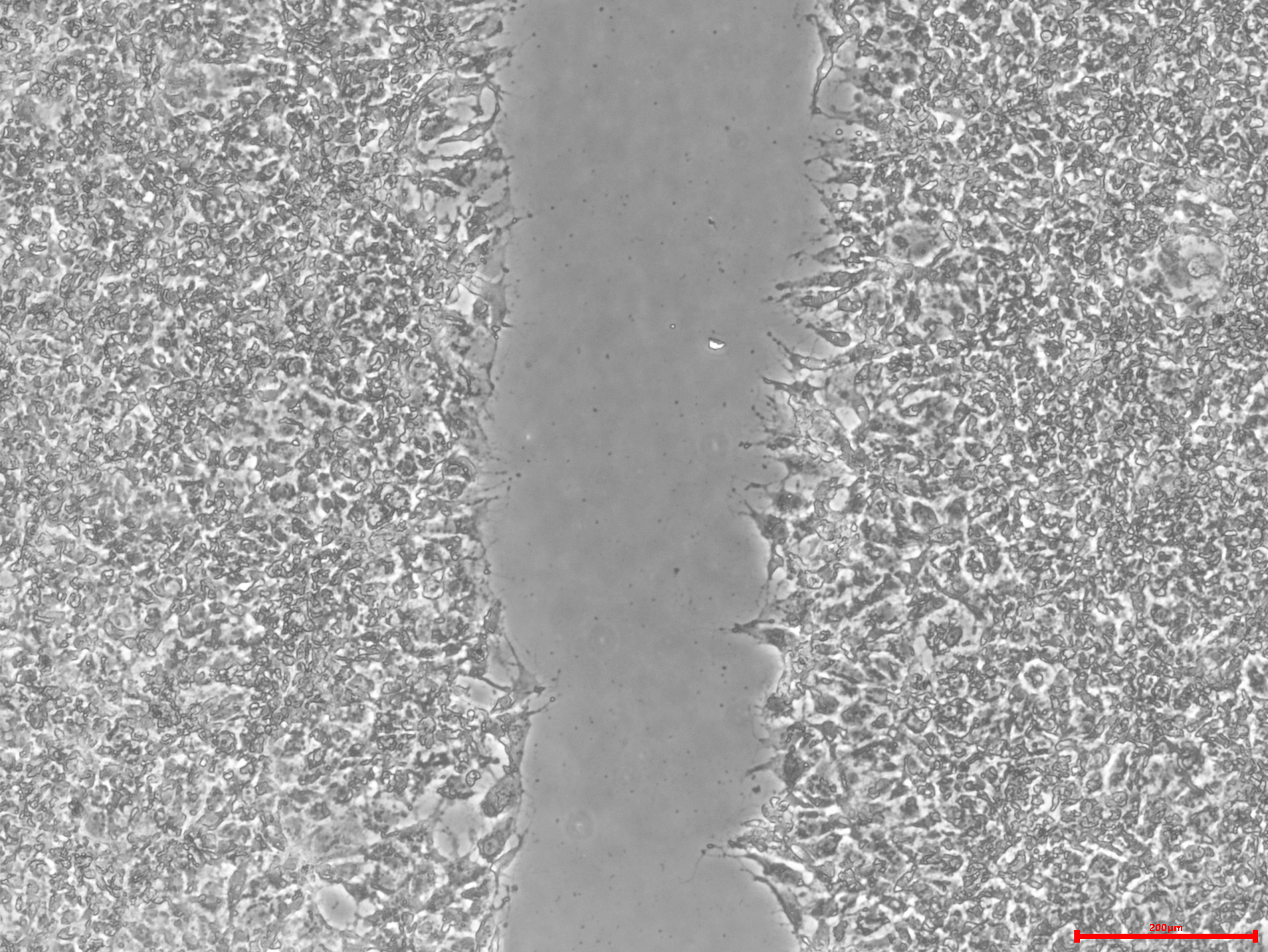

Supplement: Supplementary file 3 — Additional file 2. [file 12964_2023_1355_MOESM2_ESM.zip › raw data/Figure 2/Figure 2D/Figure 2D_Huh7_SOR_48 h.tif]

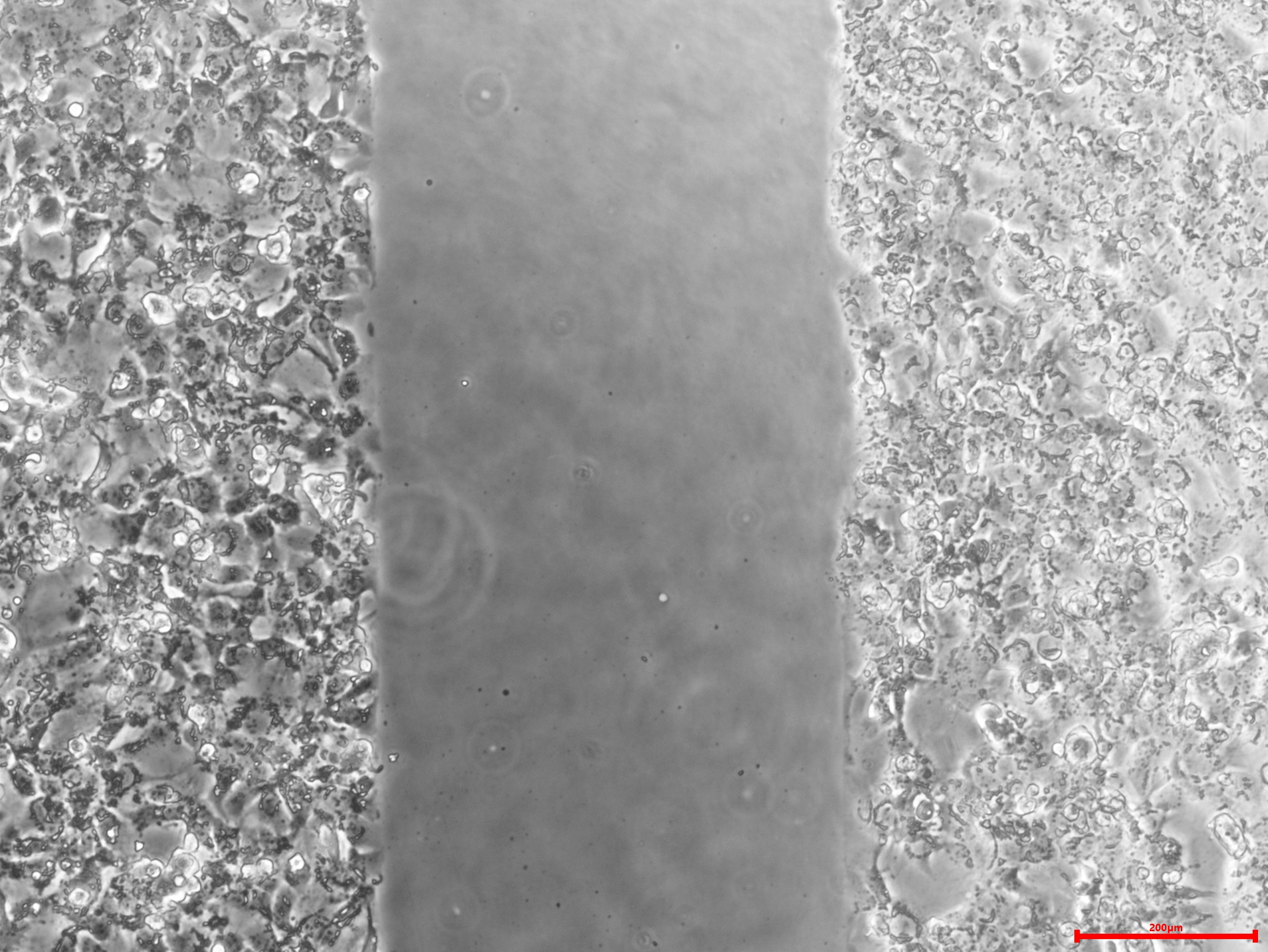

Supplement: Supplementary file 3 — Additional file 2. [file 12964_2023_1355_MOESM2_ESM.zip › raw data/Figure 2/Figure 2D/Figure 2D_Hep3B_SOR_0 h.jpg]

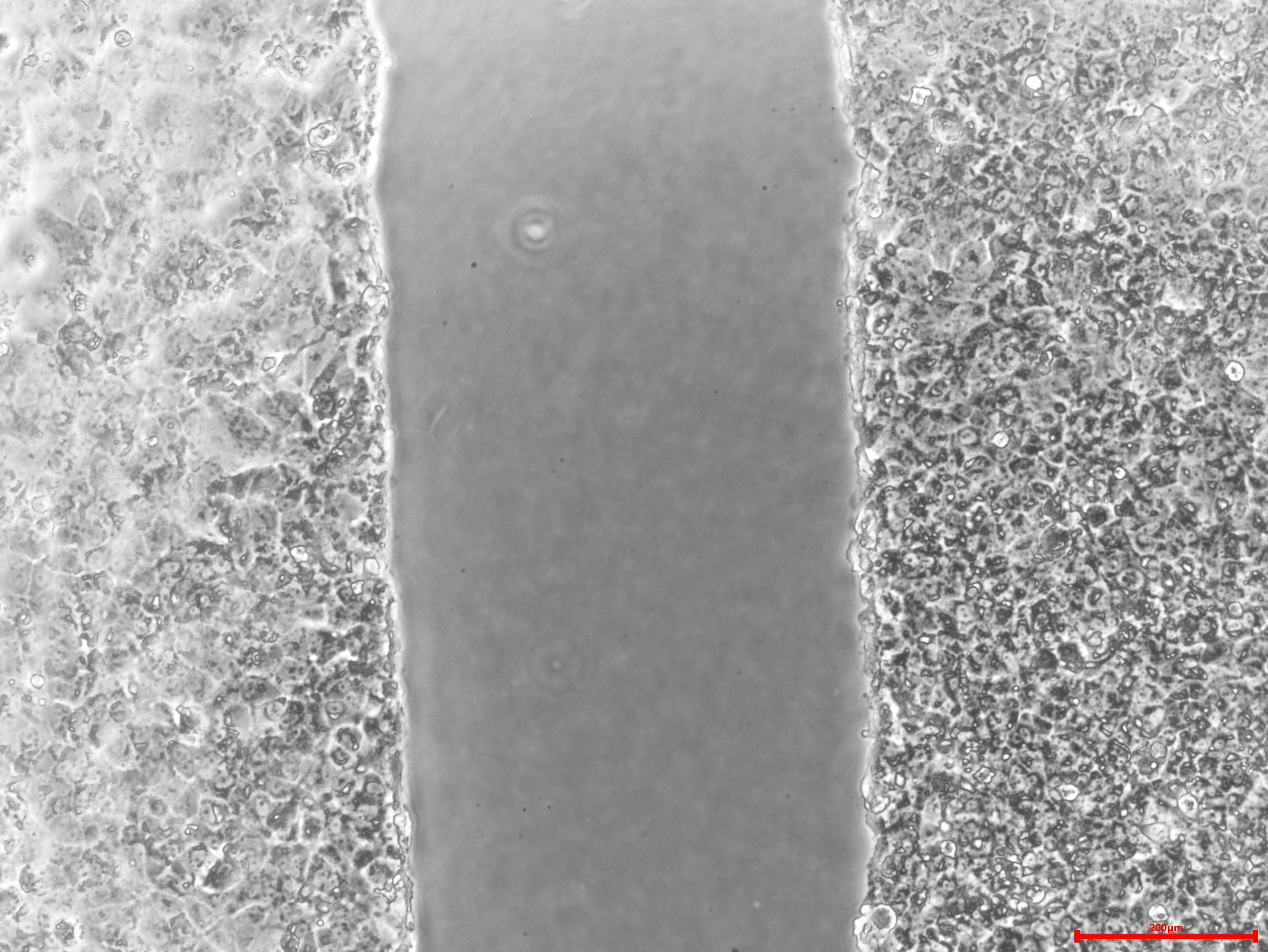

Supplement: Supplementary file 3 — Additional file 2. [file 12964_2023_1355_MOESM2_ESM.zip › raw data/Figure 2/Figure 2D/Figure 2D_Huh7_SOR+WAY_0 h.jpg]

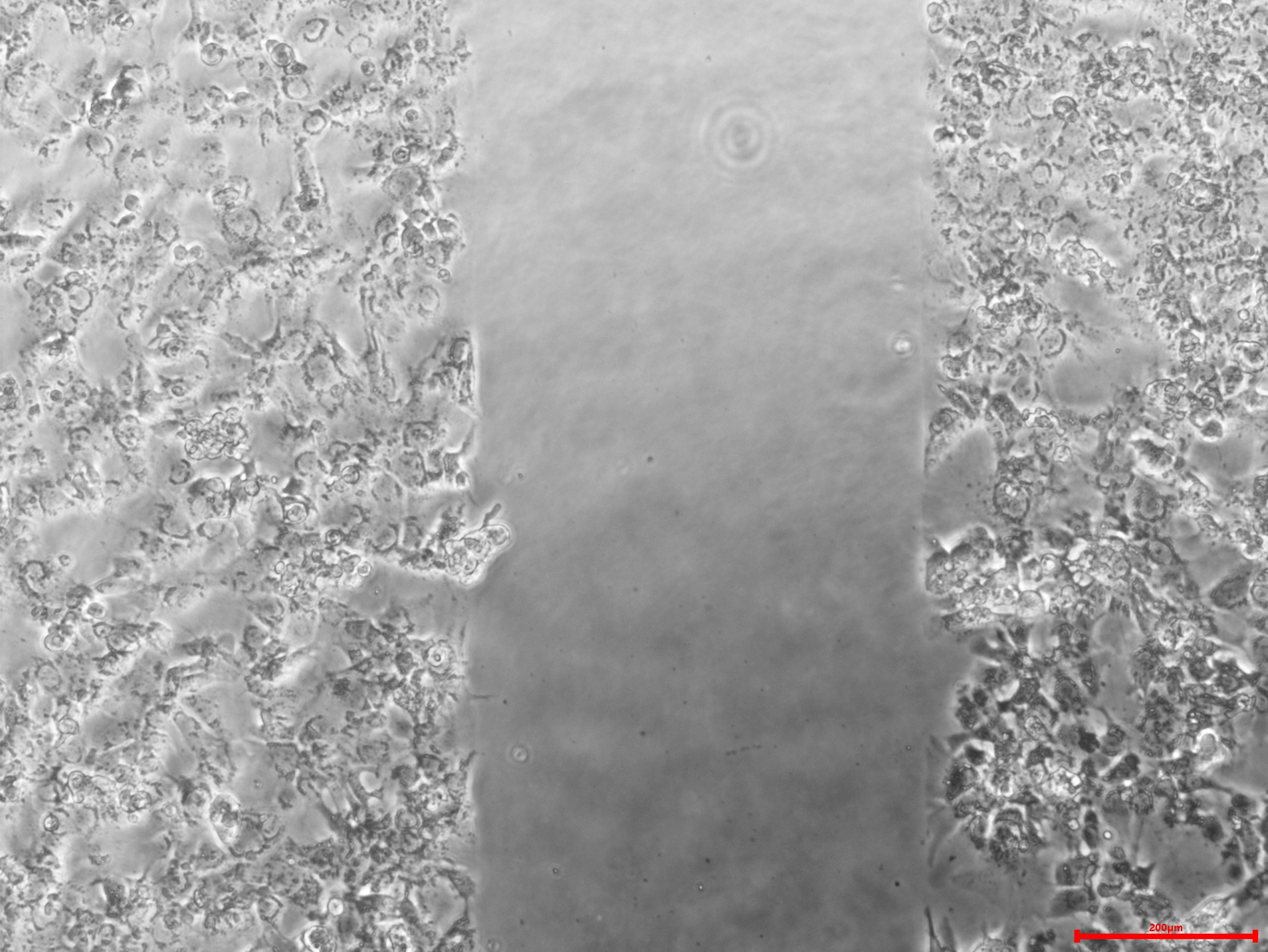

Supplement: Supplementary file 3 — Additional file 2. [file 12964_2023_1355_MOESM2_ESM.zip › raw data/Figure 2/Figure 2D/Figure 2D_Hep3B_Vehicle_0 h.jpg]

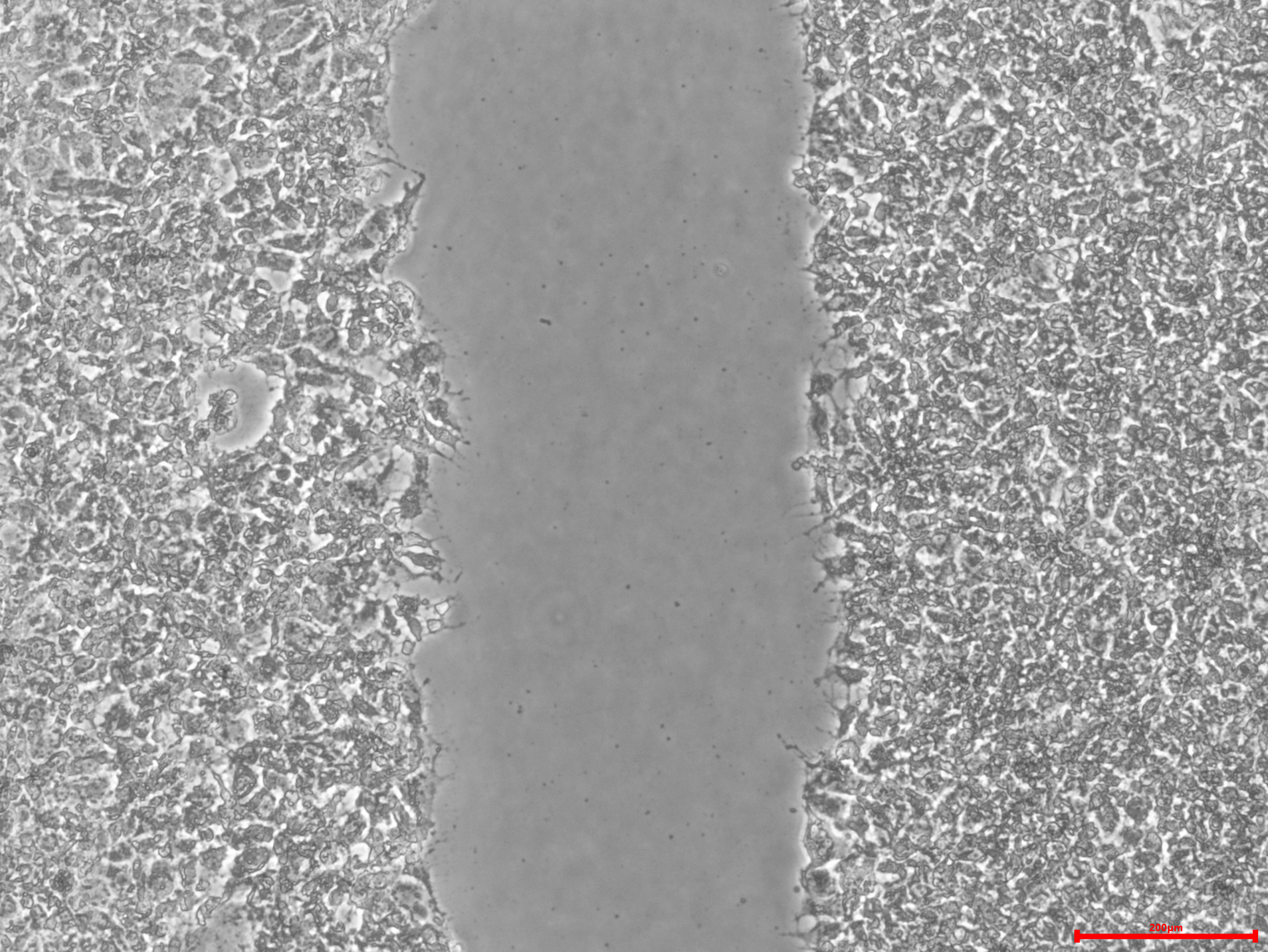

Supplement: Supplementary file 3 — Additional file 2. [file 12964_2023_1355_MOESM2_ESM.zip › raw data/Figure 2/Figure 2D/Figure 2D_Huh7_SOR+WAY_48 h.tif]

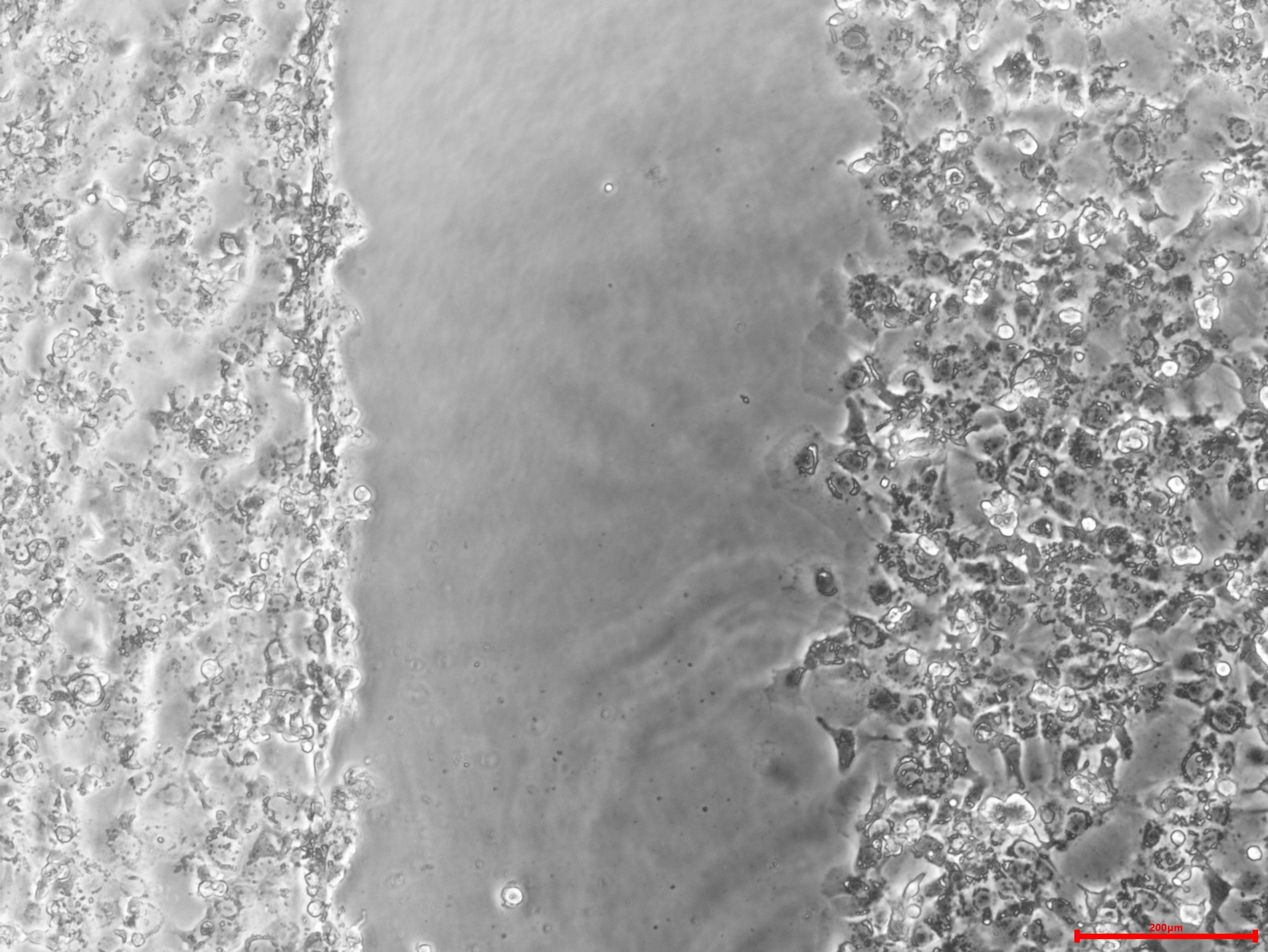

Supplement: Supplementary file 3 — Additional file 2. [file 12964_2023_1355_MOESM2_ESM.zip › raw data/Figure 2/Figure 2D/Figure 2D_Hep3B_WAY_0 h.jpg]

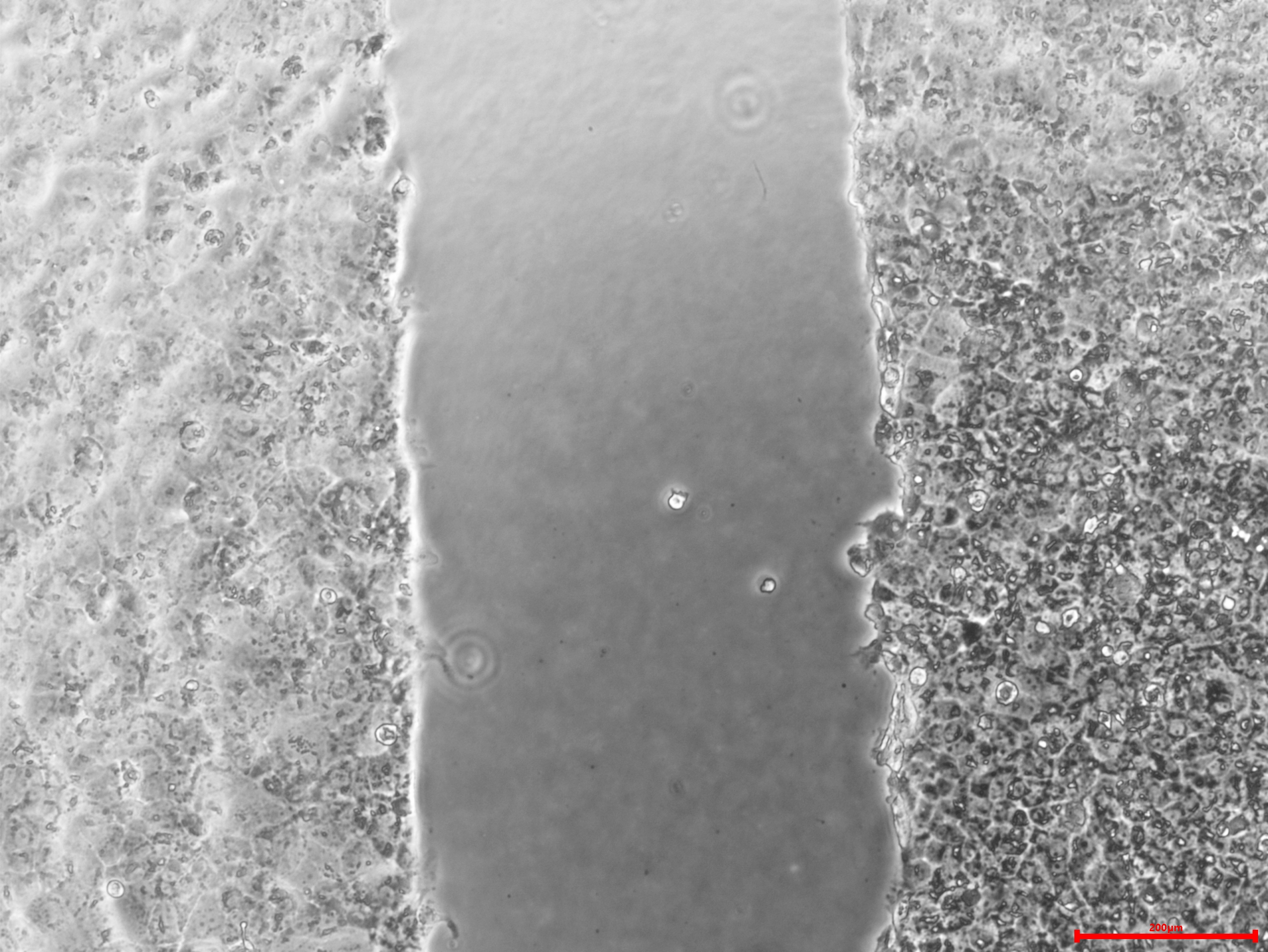

Supplement: Supplementary file 3 — Additional file 2. [file 12964_2023_1355_MOESM2_ESM.zip › raw data/Figure 2/Figure 2D/Figure 2D_Huh7_Vehicle_0 h.jpg]

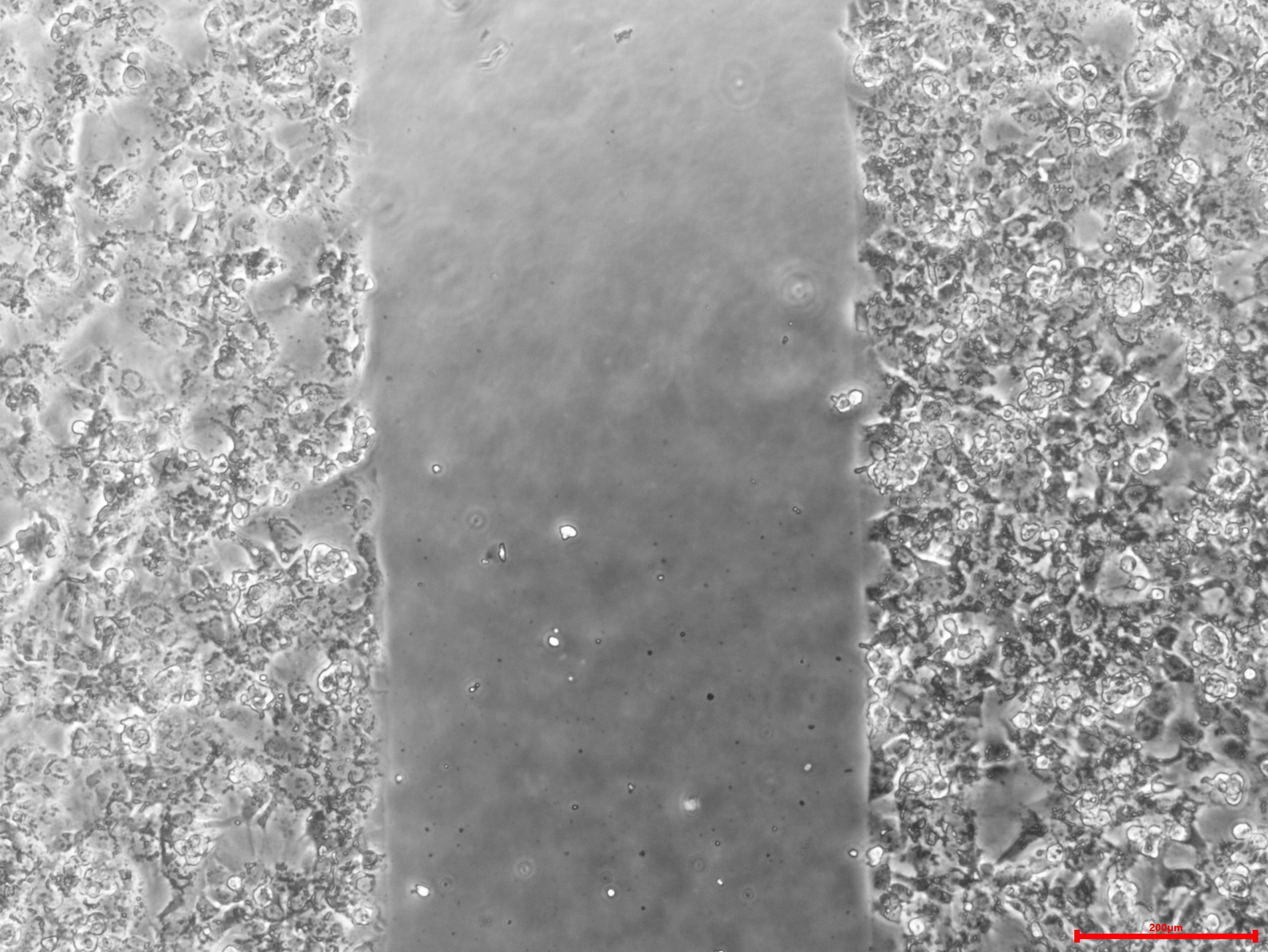

Supplement: Supplementary file 3 — Additional file 2. [file 12964_2023_1355_MOESM2_ESM.zip › raw data/Figure 2/Figure 2D/Figure 2D_Hep3B_SOR+WAY_0 h.jpg]

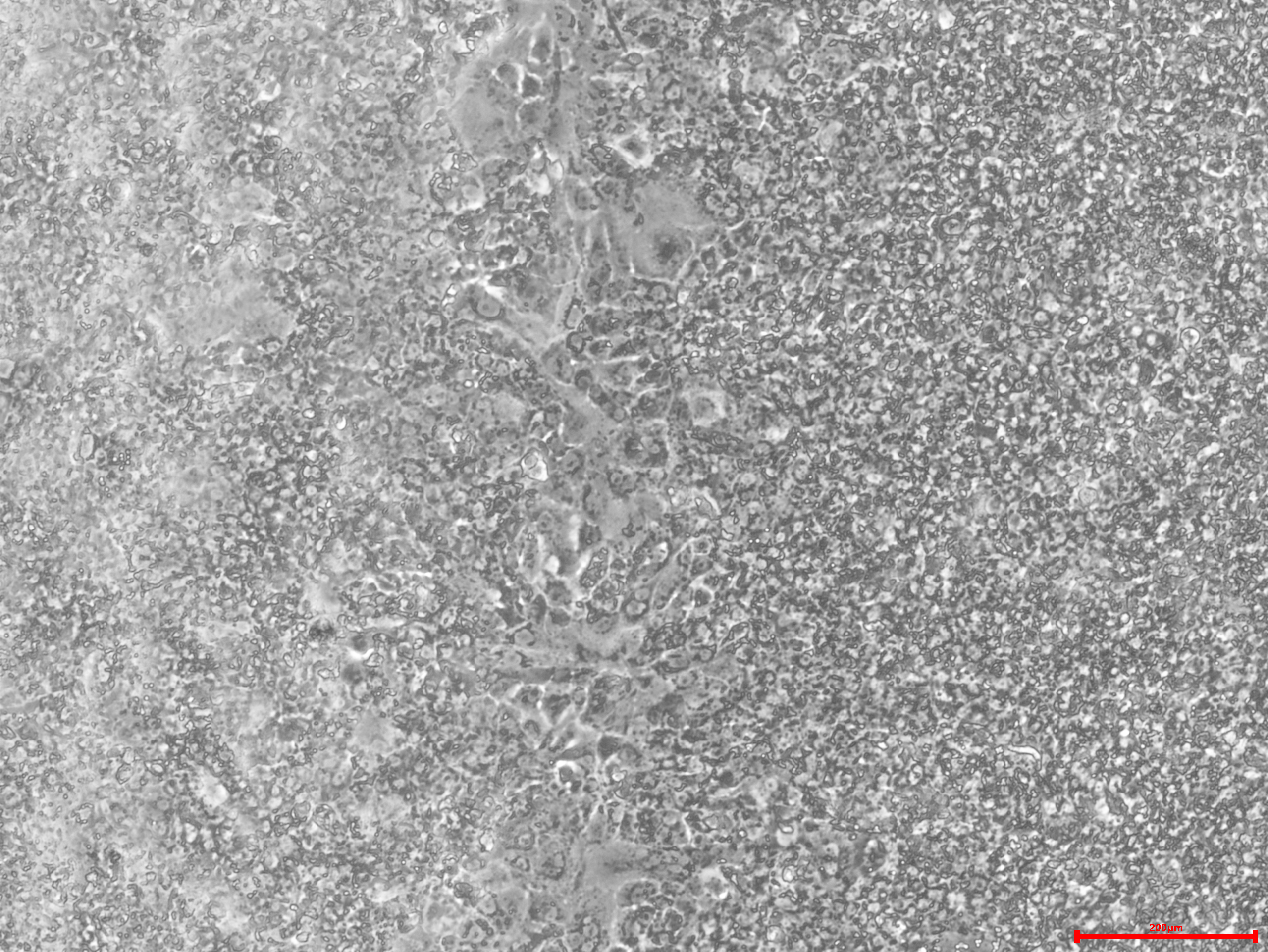

Supplement: Supplementary file 3 — Additional file 2. [file 12964_2023_1355_MOESM2_ESM.zip › raw data/Figure 2/Figure 2D/Figure 2D_Huh7_Vehicle_48 h.tif]

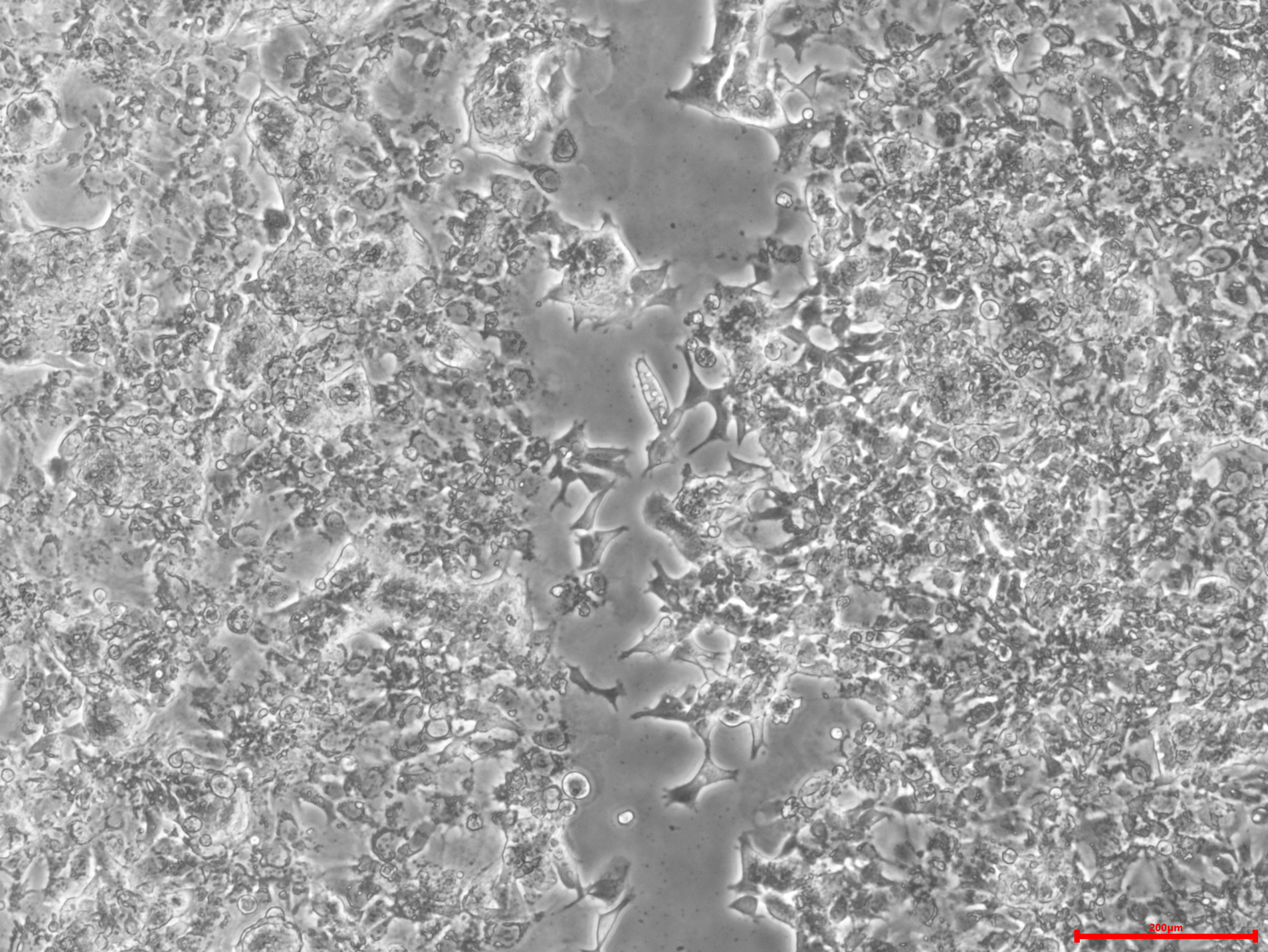

Supplement: Supplementary file 3 — Additional file 2. [file 12964_2023_1355_MOESM2_ESM.zip › raw data/Figure 2/Figure 2D/Figure 2D_Hep3B_Vehicle_48 h.tif]

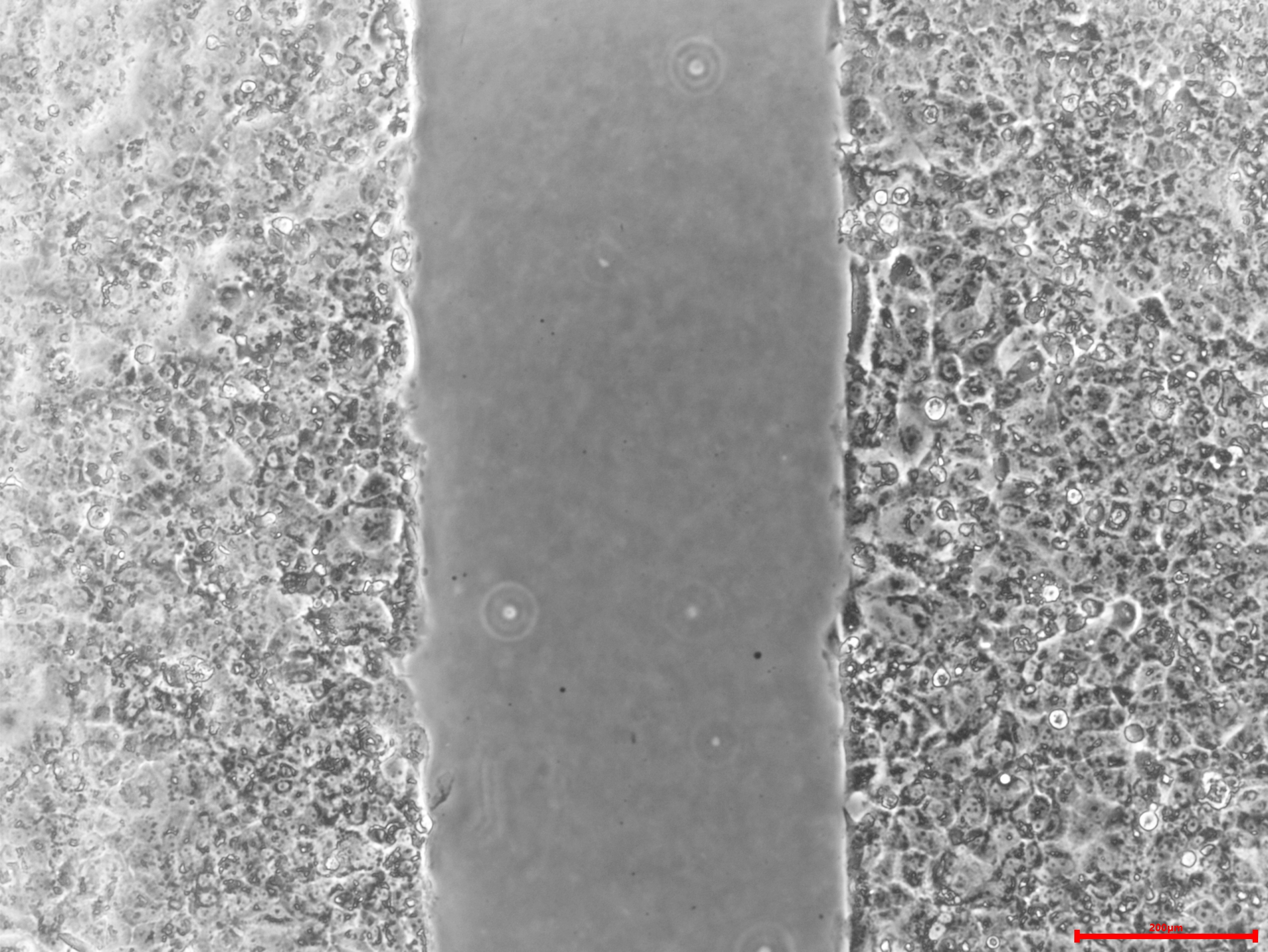

Supplement: Supplementary file 3 — Additional file 2. [file 12964_2023_1355_MOESM2_ESM.zip › raw data/Figure 2/Figure 2D/Figure 2D_Huh7_WAY_0 h.jpg]

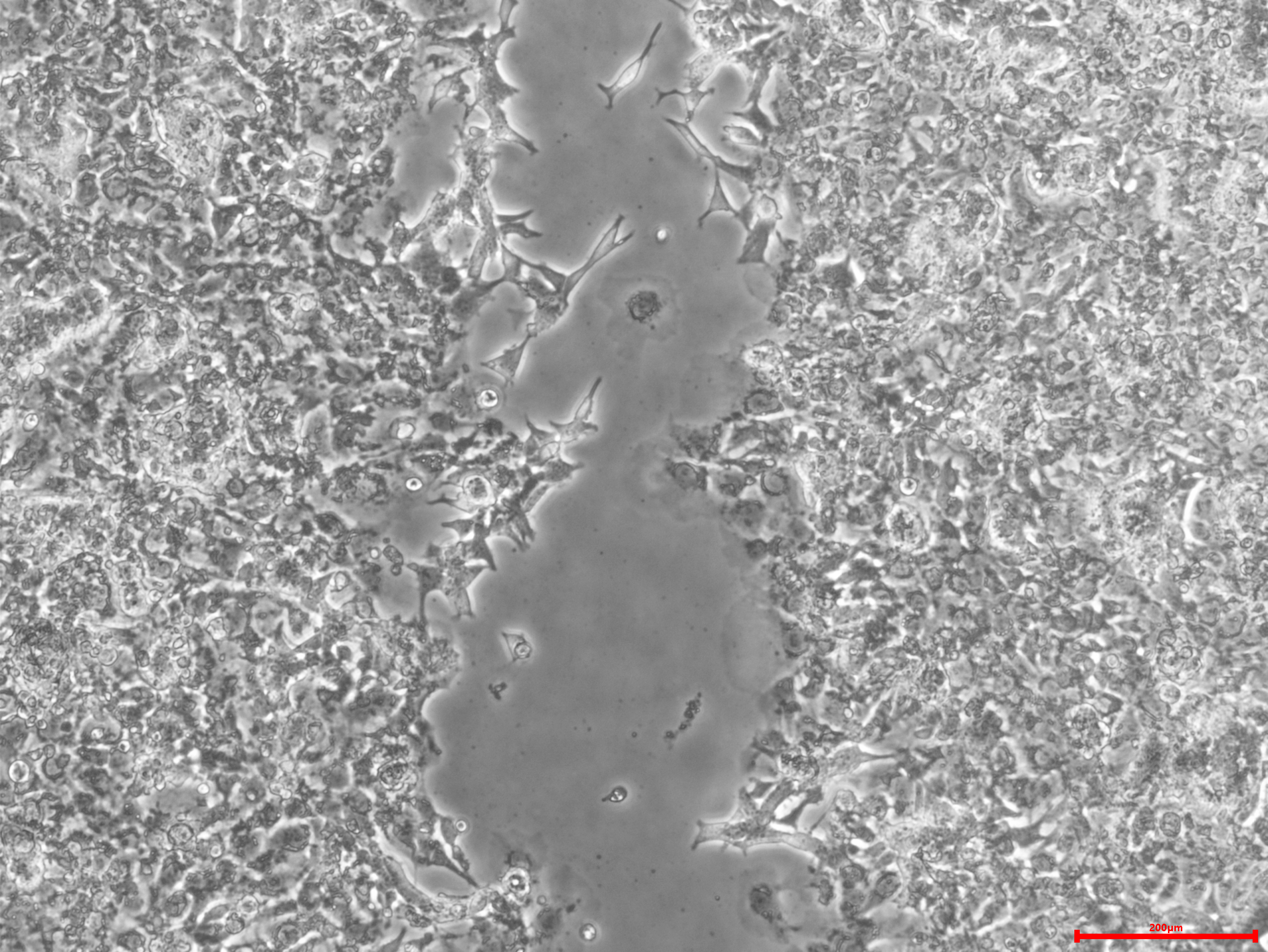

Supplement: Supplementary file 3 — Additional file 2. [file 12964_2023_1355_MOESM2_ESM.zip › raw data/Figure 2/Figure 2D/Figure 2D_Hep3B_SOR_48 h.tif]

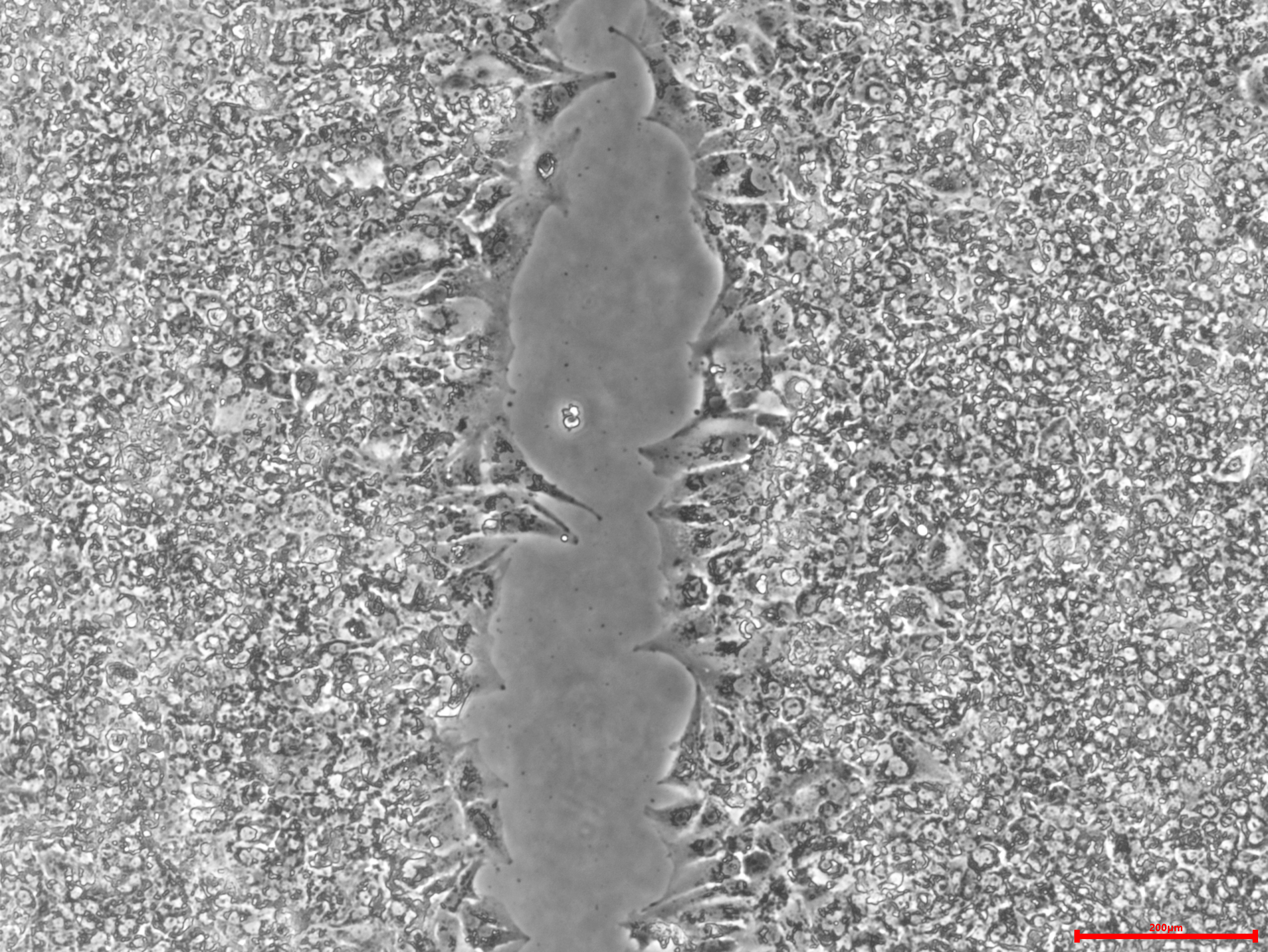

Supplement: Supplementary file 3 — Additional file 2. [file 12964_2023_1355_MOESM2_ESM.zip › raw data/Figure 2/Figure 2D/Figure 2D_Huh7_WAY_48 h.tif]

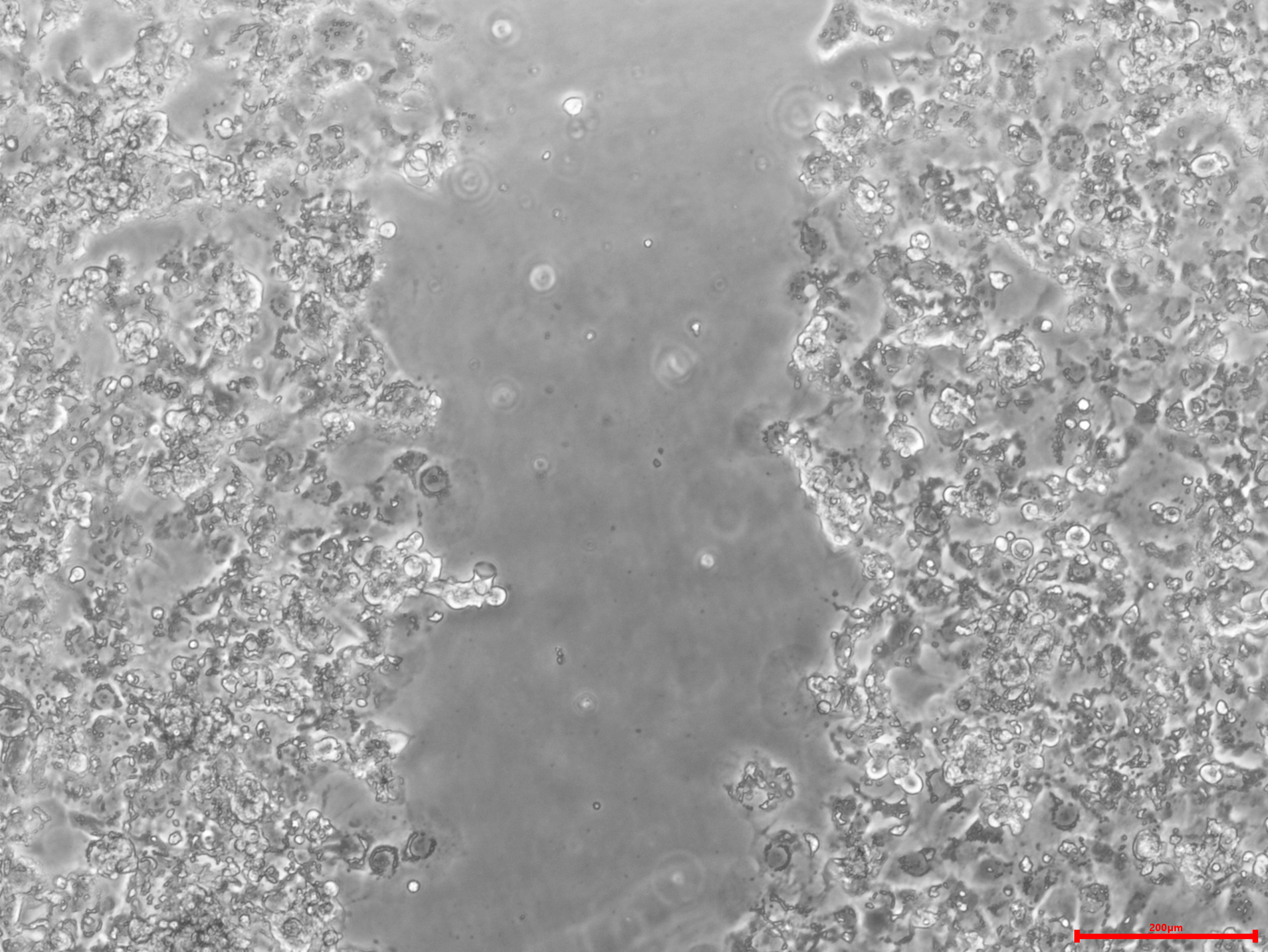

Supplement: Supplementary file 3 — Additional file 2. [file 12964_2023_1355_MOESM2_ESM.zip › raw data/Figure 2/Figure 2D/Figure 2D_Hep3B_WAY_48 h.tif]

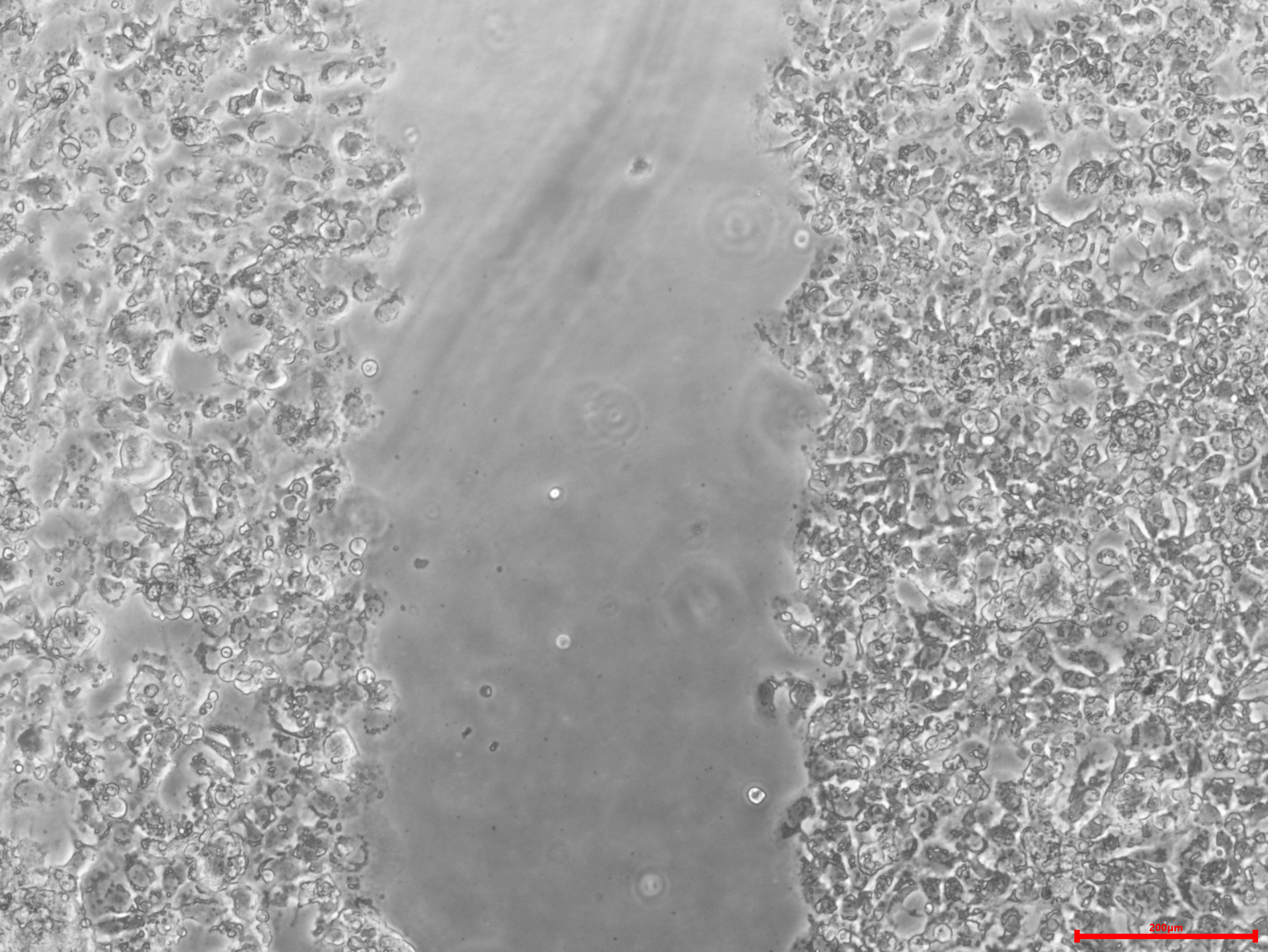

Supplement: Supplementary file 3 — Additional file 2. [file 12964_2023_1355_MOESM2_ESM.zip › raw data/Figure 2/Figure 2D/Figure 2D_Hep3B_SOR+WAY_48 h.tif]

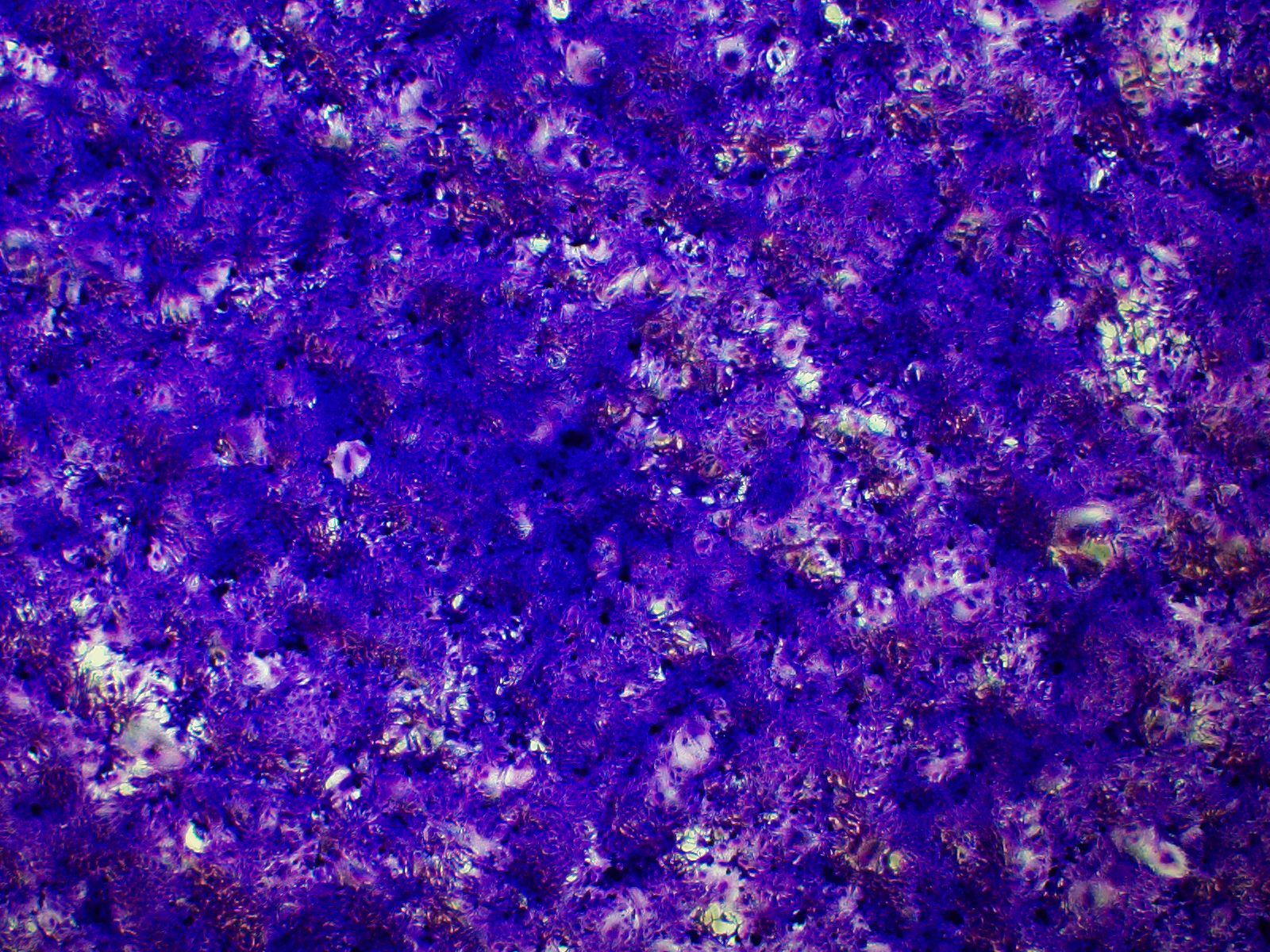

Supplement: Supplementary file 3 — Additional file 2. [file 12964_2023_1355_MOESM2_ESM.zip › raw data/Figure 2/Figure 2E/Figure 2E_Huh7_SOR 1.5 ╬╝M+WAY 0 ╬╝M.tif]

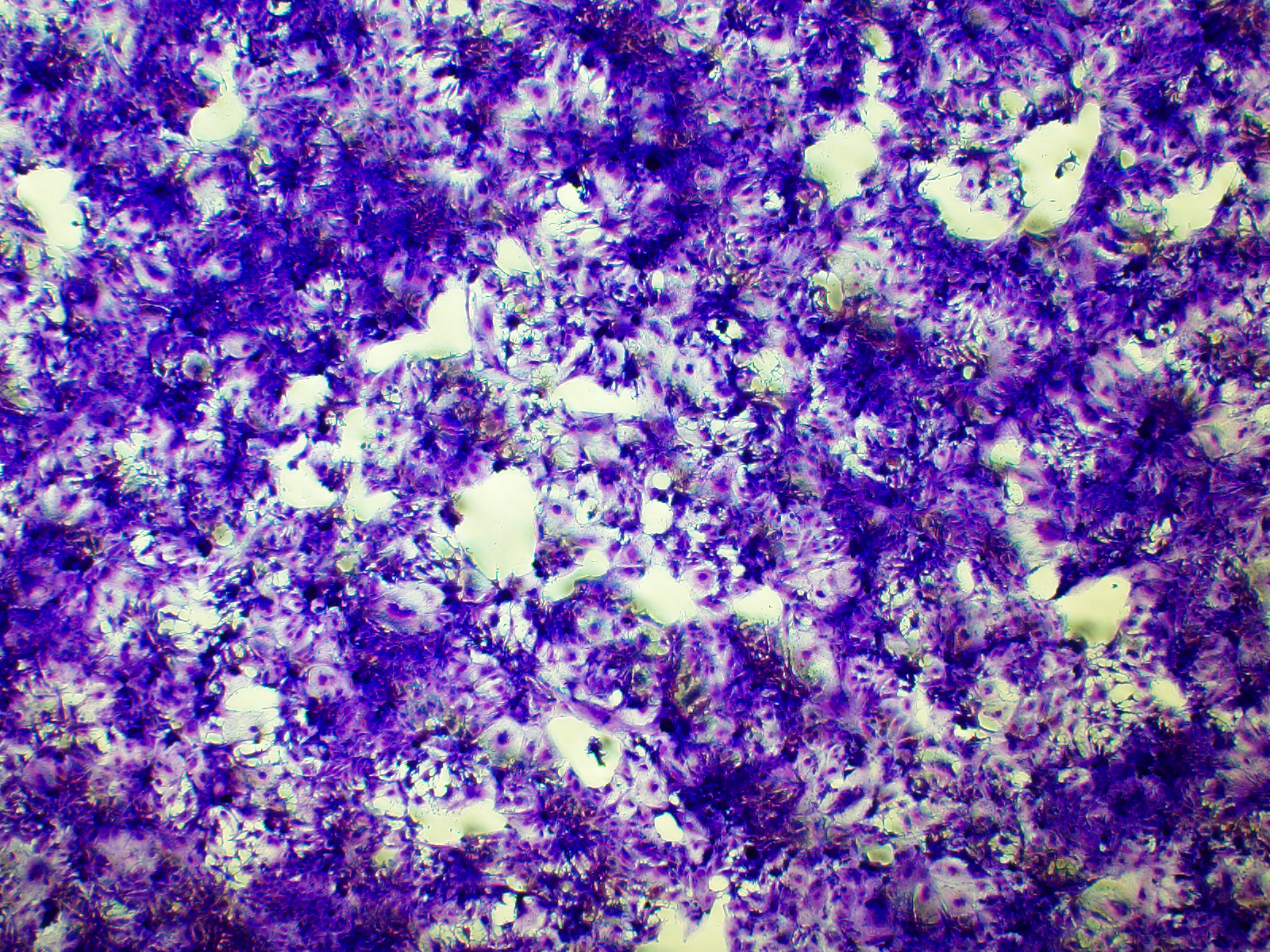

Supplement: Supplementary file 3 — Additional file 2. [file 12964_2023_1355_MOESM2_ESM.zip › raw data/Figure 2/Figure 2E/Figure 2E_Huh7_SOR 3 ╬╝M+WAY 0 ╬╝M.tif]

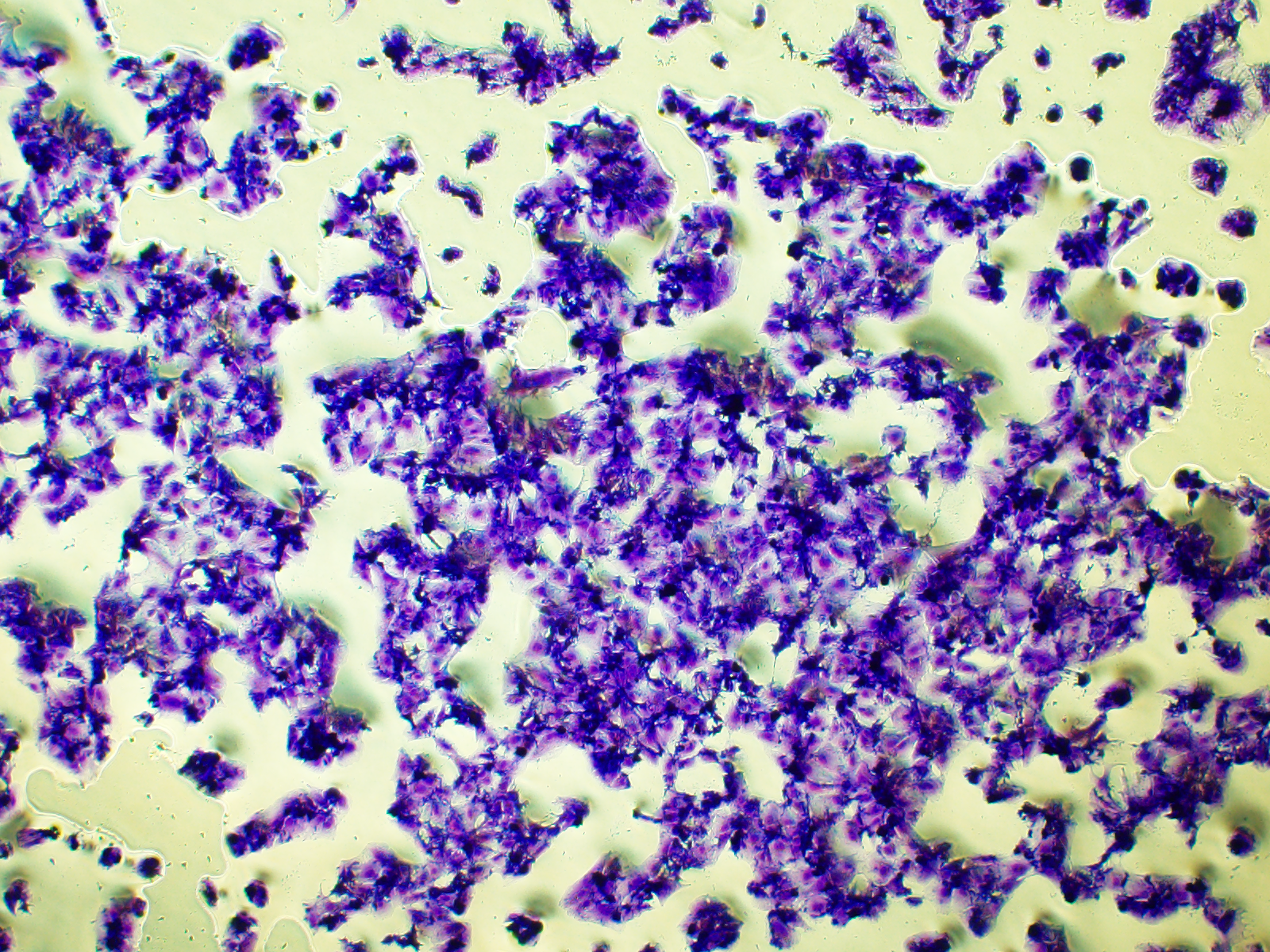

Supplement: Supplementary file 3 — Additional file 2. [file 12964_2023_1355_MOESM2_ESM.zip › raw data/Figure 2/Figure 2E/Figure 2E_Huh7_SOR 6 ╬╝M+WAY 0 ╬╝M.tif]

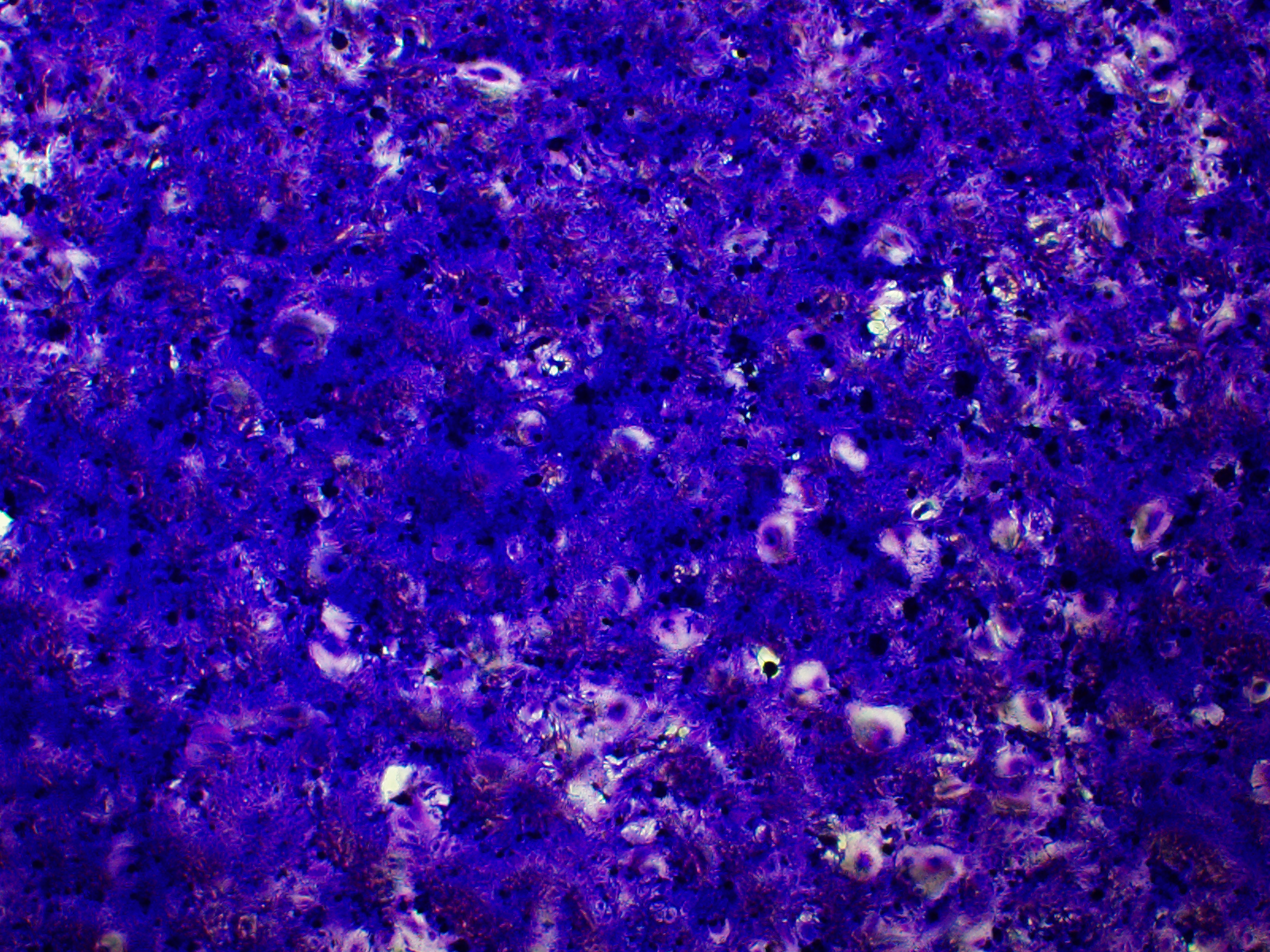

Supplement: Supplementary file 3 — Additional file 2. [file 12964_2023_1355_MOESM2_ESM.zip › raw data/Figure 2/Figure 2E/Figure 2E_Huh7_SOR 0 ╬╝M+WAY 4 ╬╝M.tif]

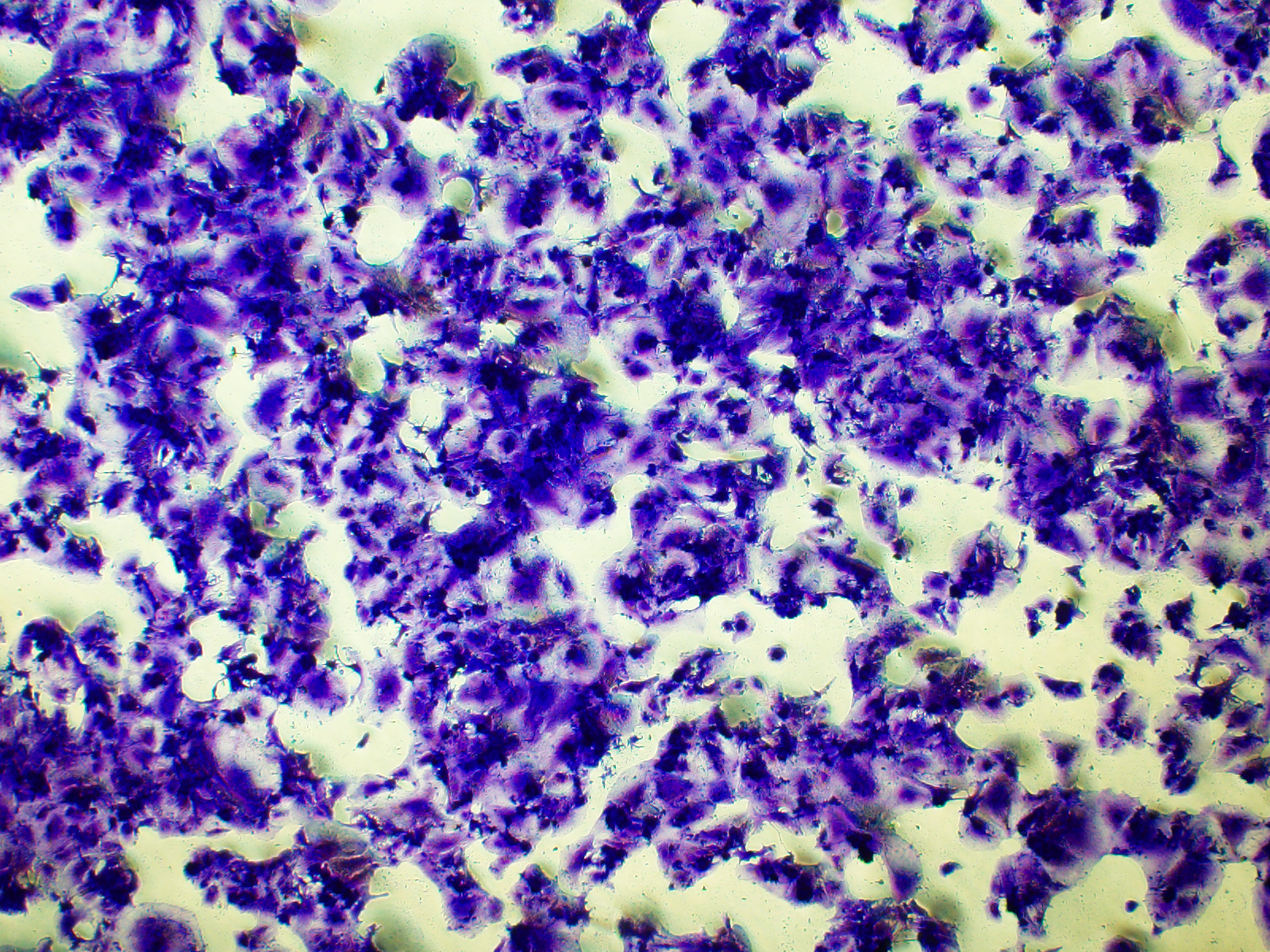

Supplement: Supplementary file 3 — Additional file 2. [file 12964_2023_1355_MOESM2_ESM.zip › raw data/Figure 2/Figure 2E/Figure 2E_Huh7_SOR 0 ╬╝M+WAY 8 ╬╝M.tif]

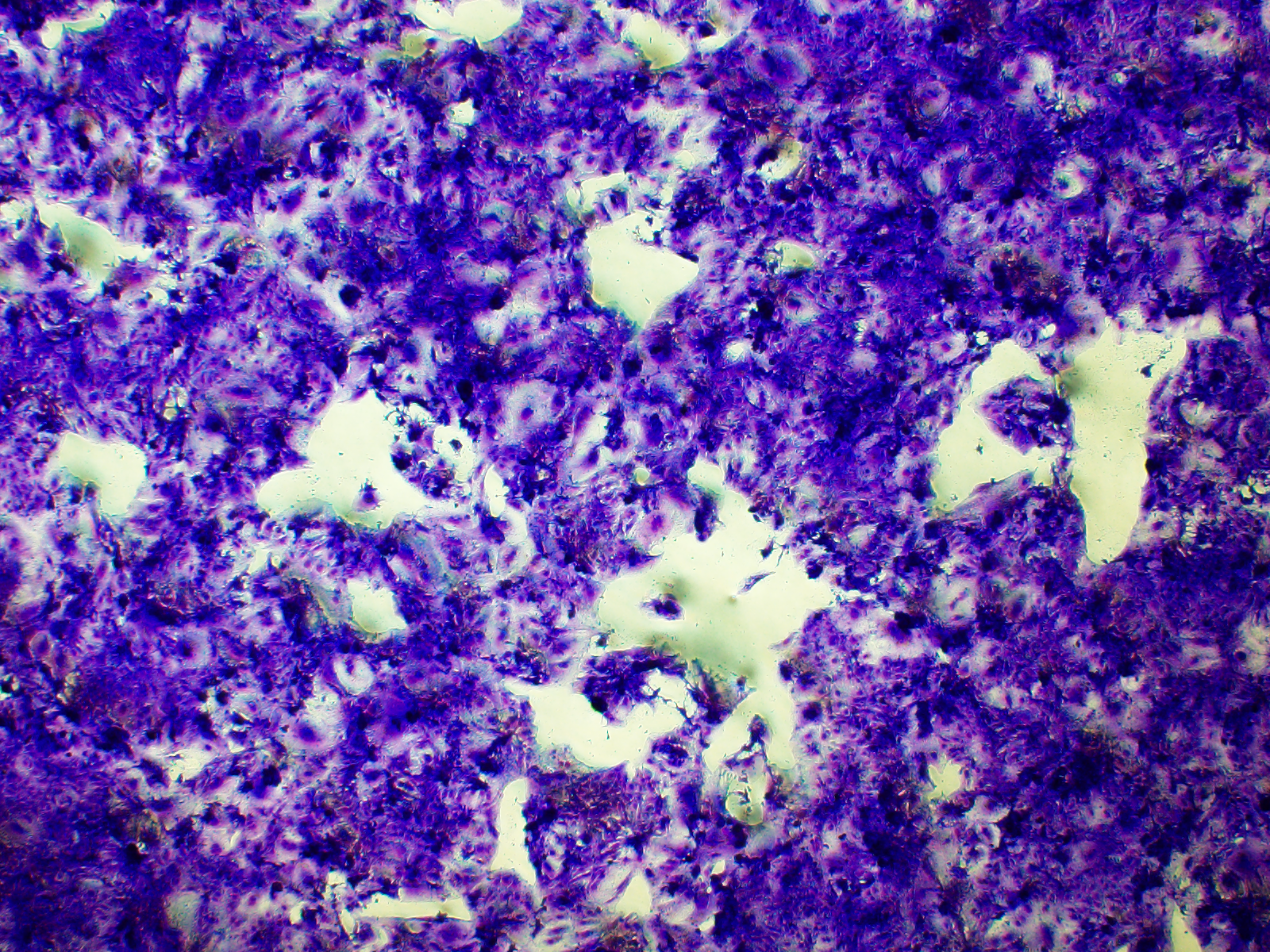

Supplement: Supplementary file 3 — Additional file 2. [file 12964_2023_1355_MOESM2_ESM.zip › raw data/Figure 2/Figure 2E/Figure 2E_Huh7_SOR 1.5 ╬╝M+WAY 4 ╬╝M.tif]

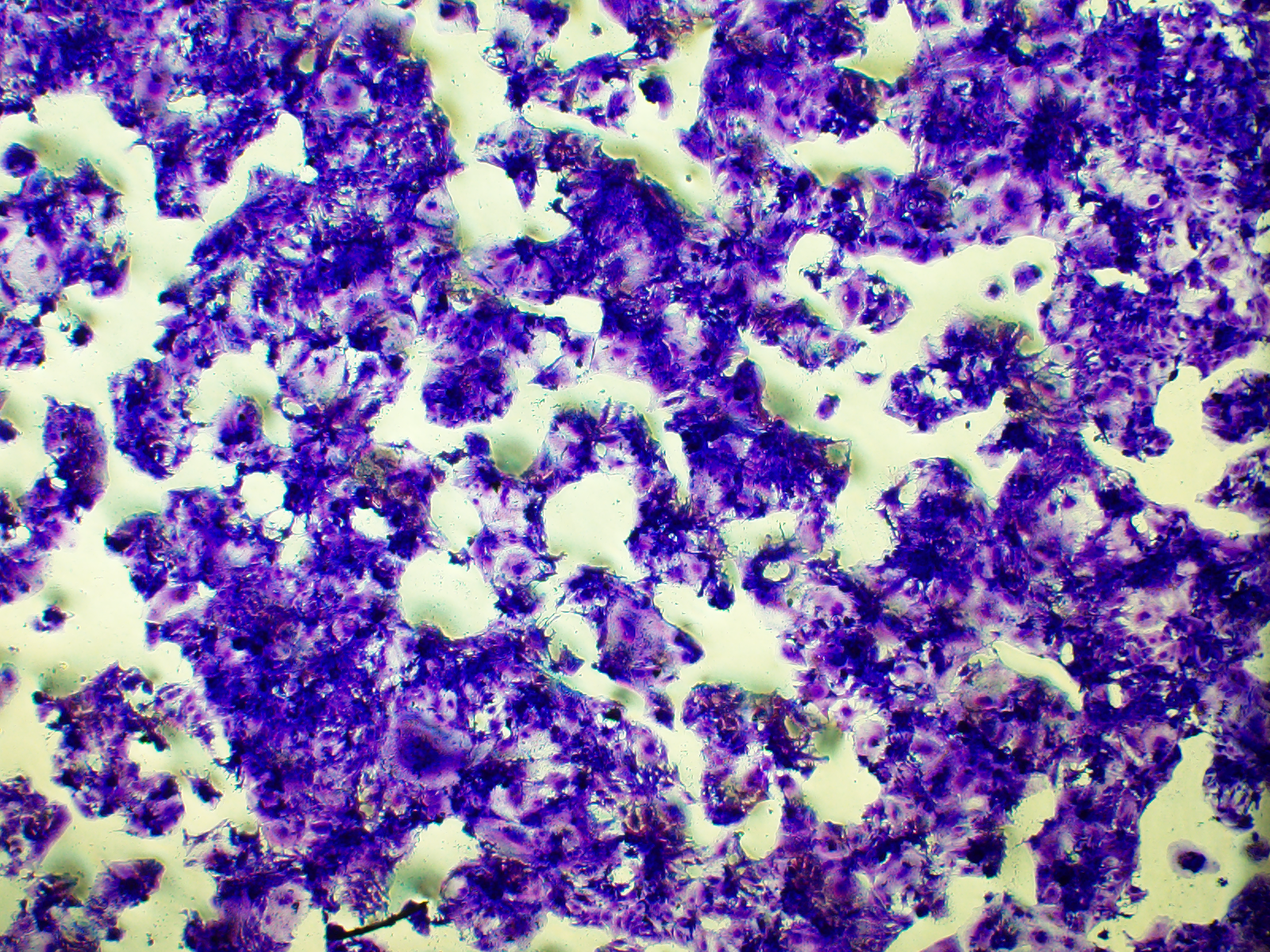

Supplement: Supplementary file 3 — Additional file 2. [file 12964_2023_1355_MOESM2_ESM.zip › raw data/Figure 2/Figure 2E/Figure 2E_Huh7_SOR 3 ╬╝M+WAY 4 ╬╝M.tif]

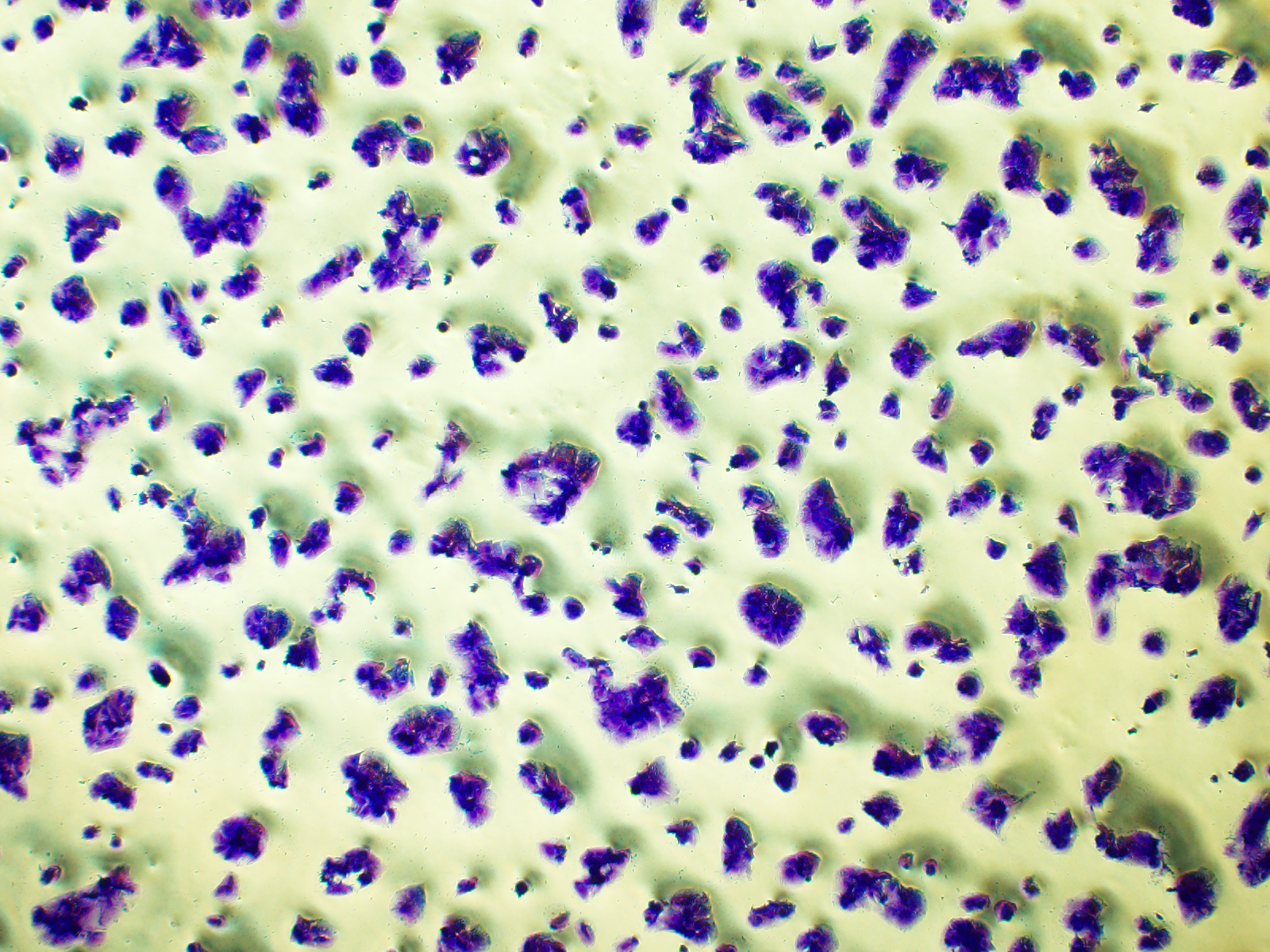

Supplement: Supplementary file 3 — Additional file 2. [file 12964_2023_1355_MOESM2_ESM.zip › raw data/Figure 2/Figure 2E/Figure 2E_Huh7_SOR 6 ╬╝M+WAY 4 ╬╝M.tif]

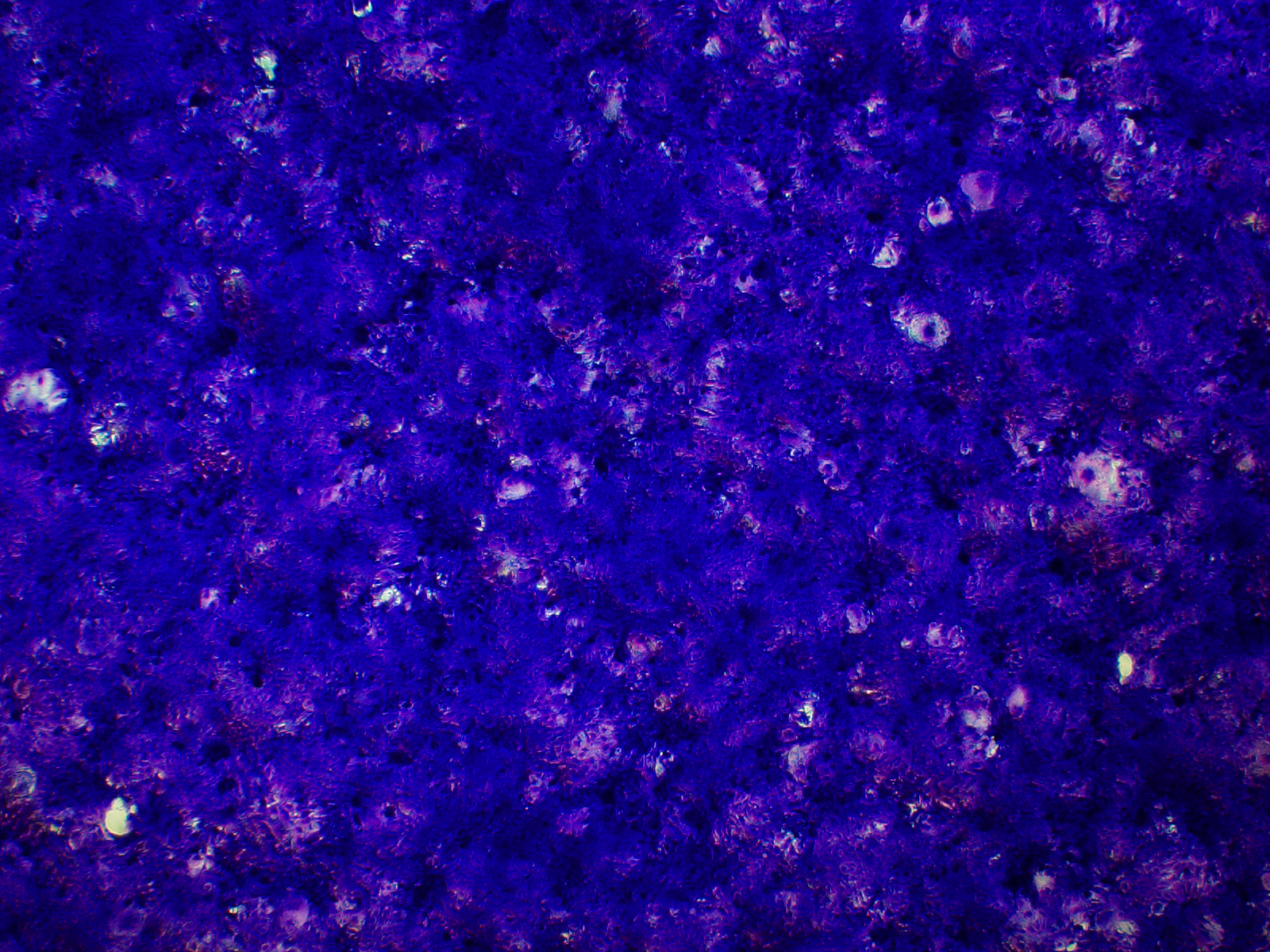

Supplement: Supplementary file 3 — Additional file 2. [file 12964_2023_1355_MOESM2_ESM.zip › raw data/Figure 2/Figure 2E/Figure 2E_Huh7_SOR 0 ╬╝M+WAY 0 ╬╝M.tif]

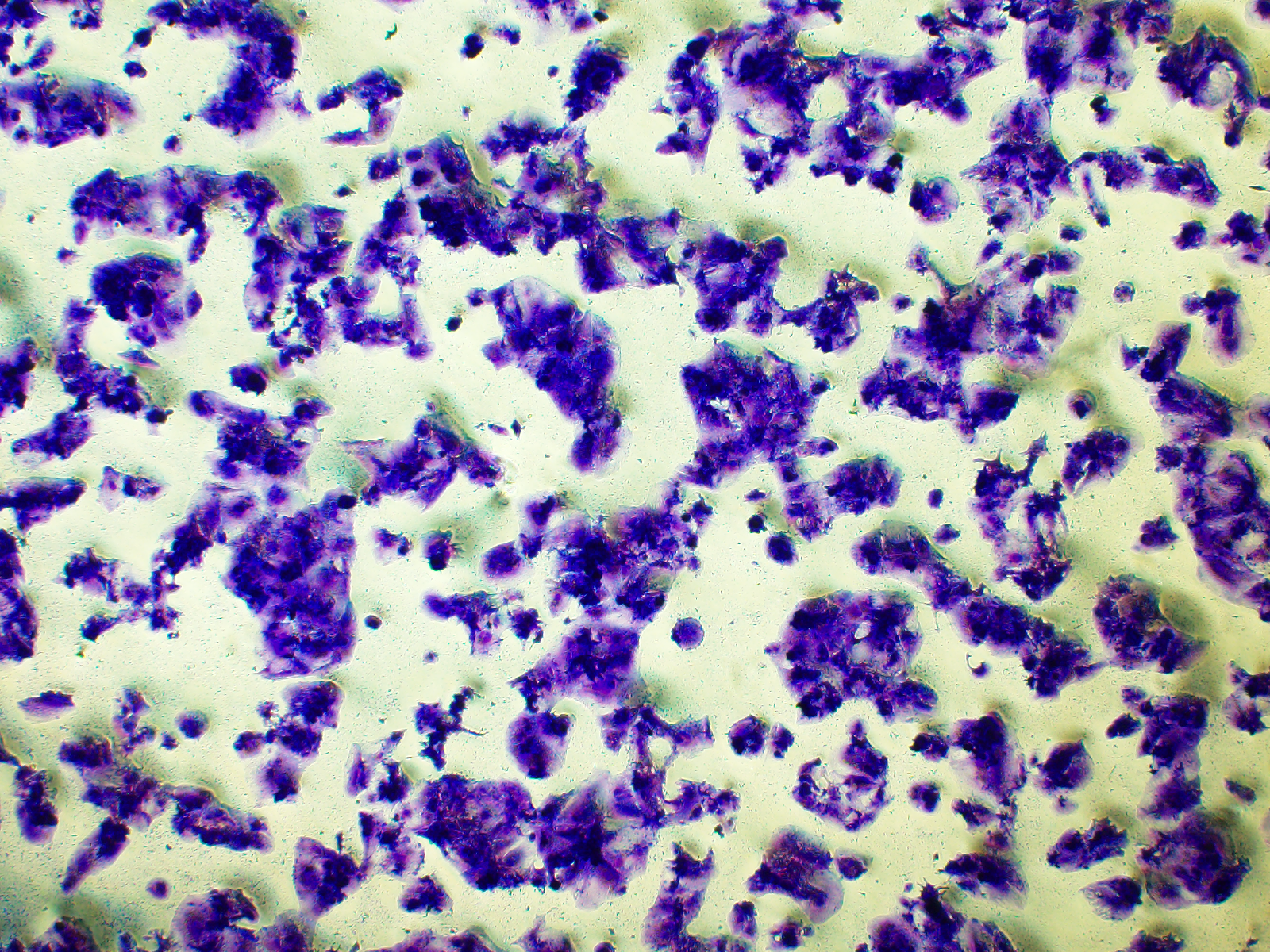

Supplement: Supplementary file 3 — Additional file 2. [file 12964_2023_1355_MOESM2_ESM.zip › raw data/Figure 2/Figure 2E/Figure 2E_Huh7_SOR 1.5 ╬╝M+WAY 8 ╬╝M.tif]

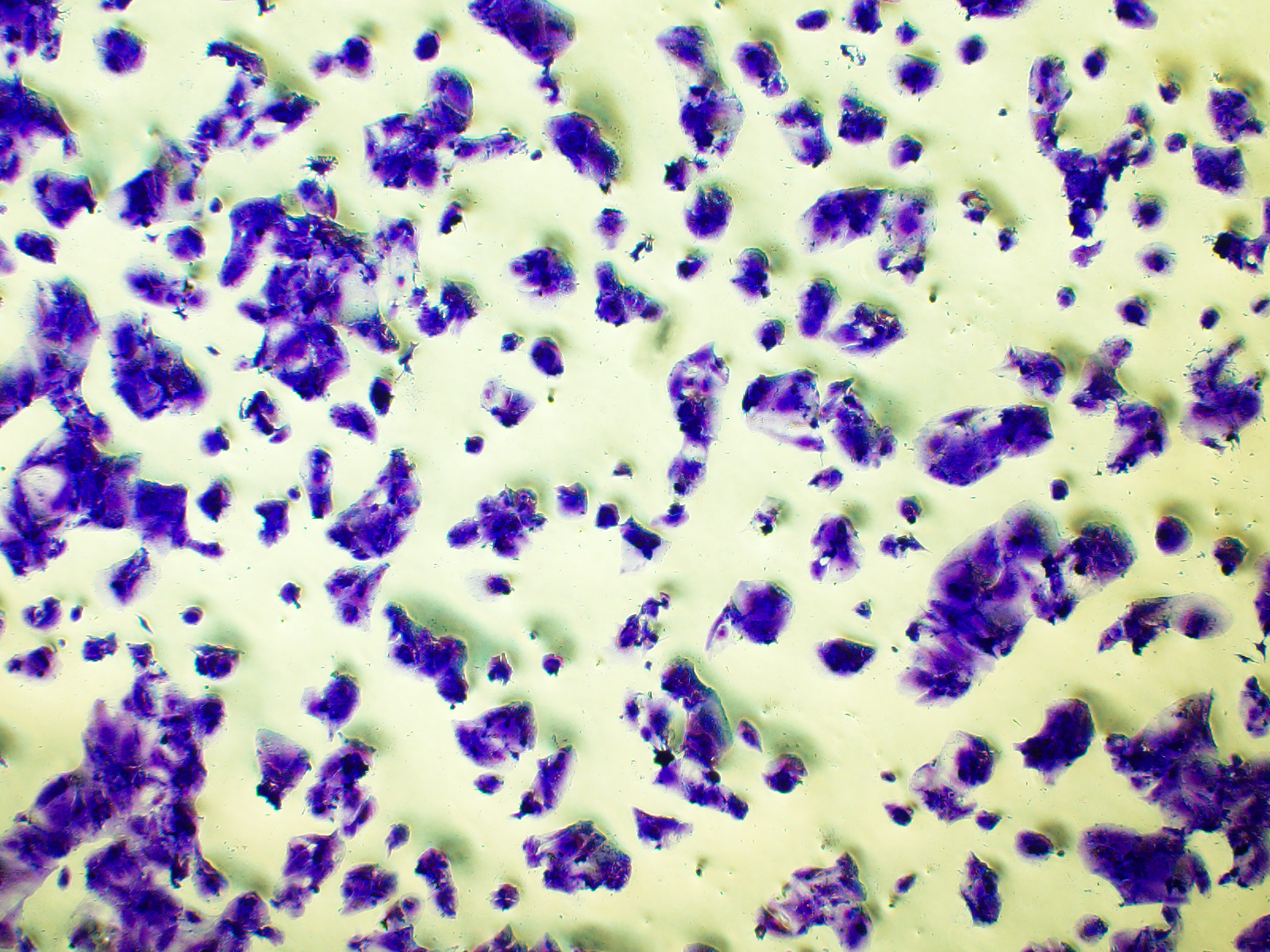

Supplement: Supplementary file 3 — Additional file 2. [file 12964_2023_1355_MOESM2_ESM.zip › raw data/Figure 2/Figure 2E/Figure 2E_Huh7_SOR 3 ╬╝M+WAY 8 ╬╝M.tif]

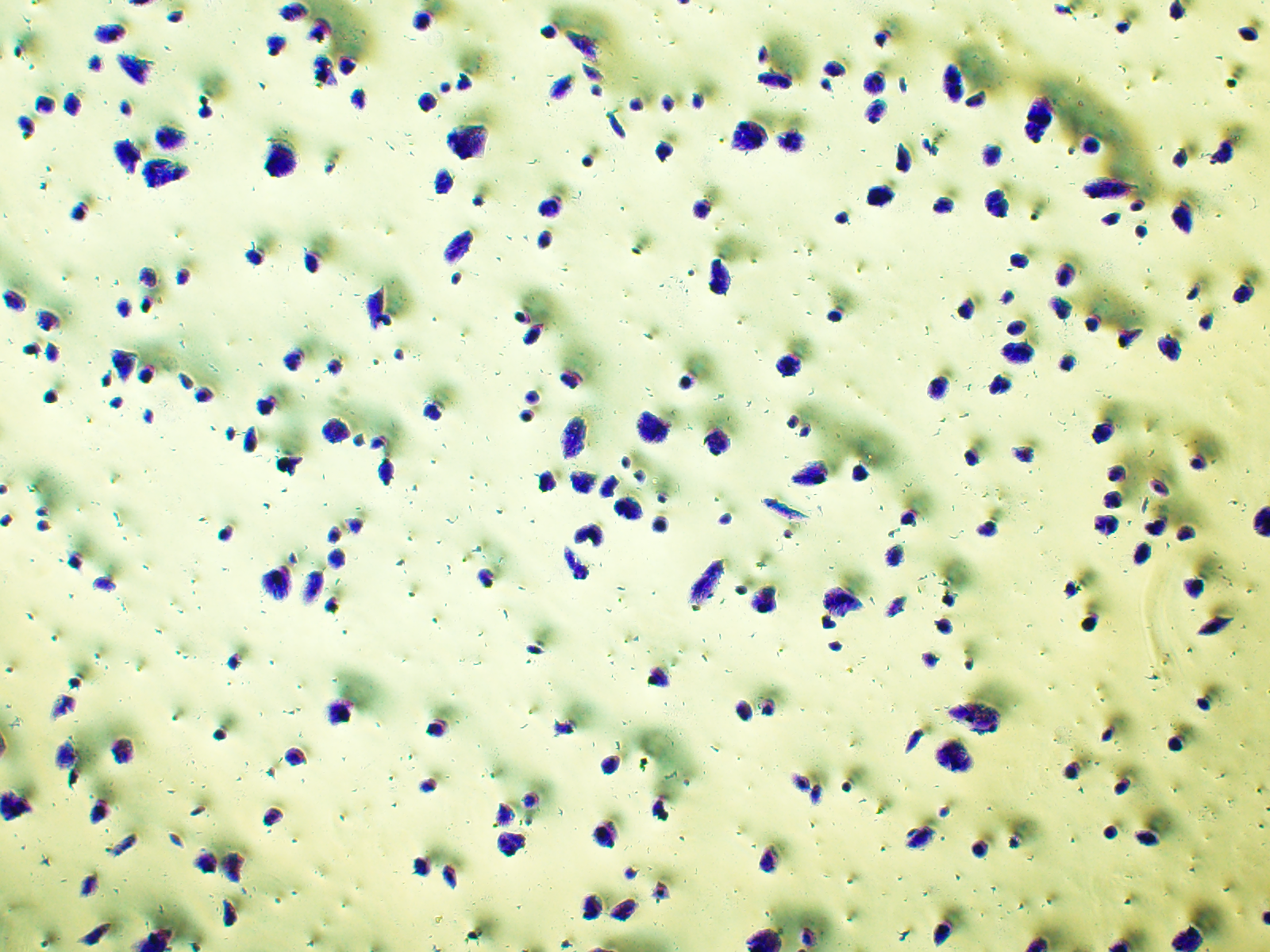

Supplement: Supplementary file 3 — Additional file 2. [file 12964_2023_1355_MOESM2_ESM.zip › raw data/Figure 2/Figure 2E/Figure 2E_Huh7_SOR 6 ╬╝M+WAY 8 ╬╝M.tif]

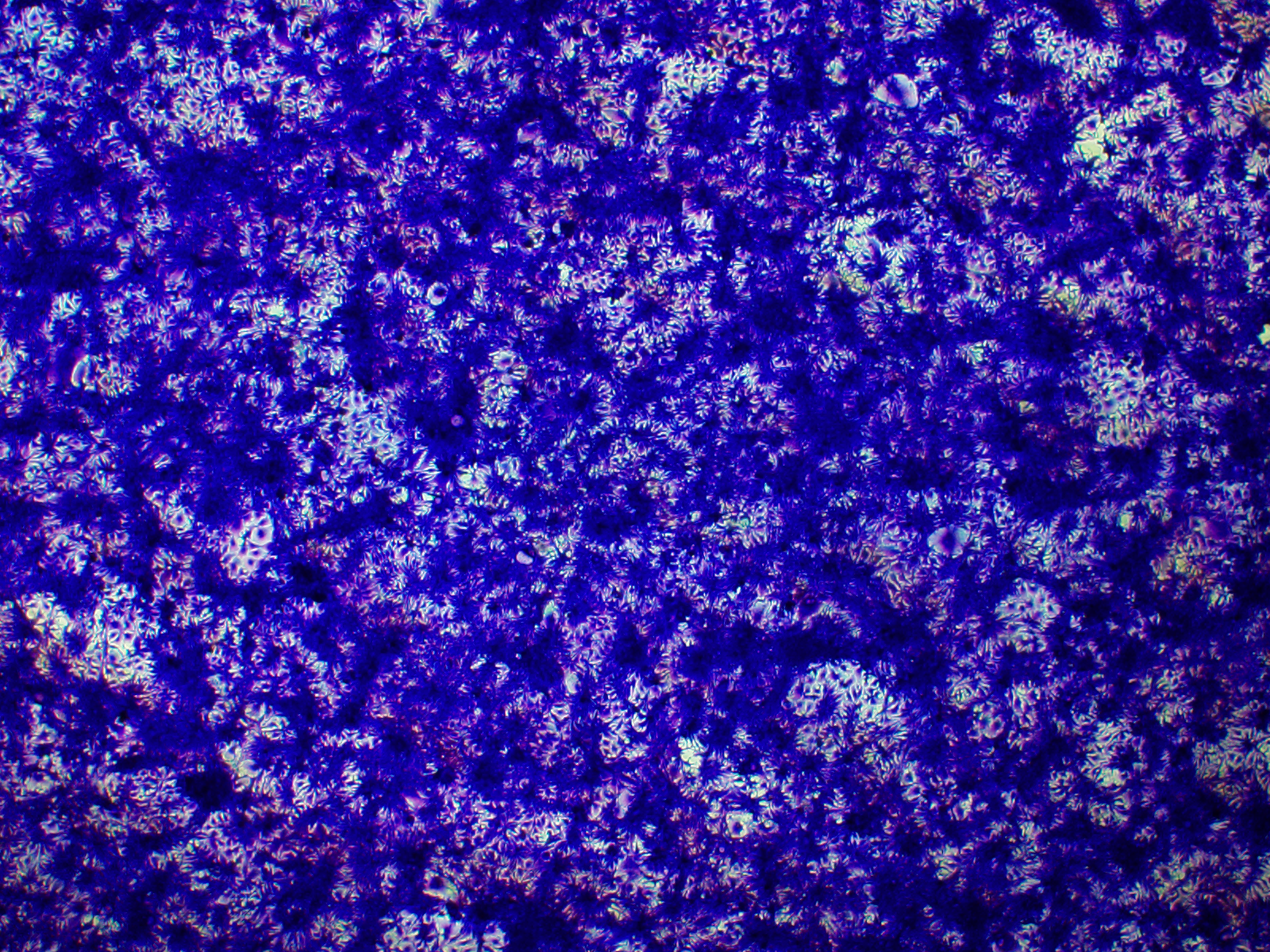

Supplement: Supplementary file 3 — Additional file 2. [file 12964_2023_1355_MOESM2_ESM.zip › raw data/Figure 2/Figure 2E/Figure 2E_Hep3B SOR 0 ╬╝M+WAY 0 ╬╝M.tif]

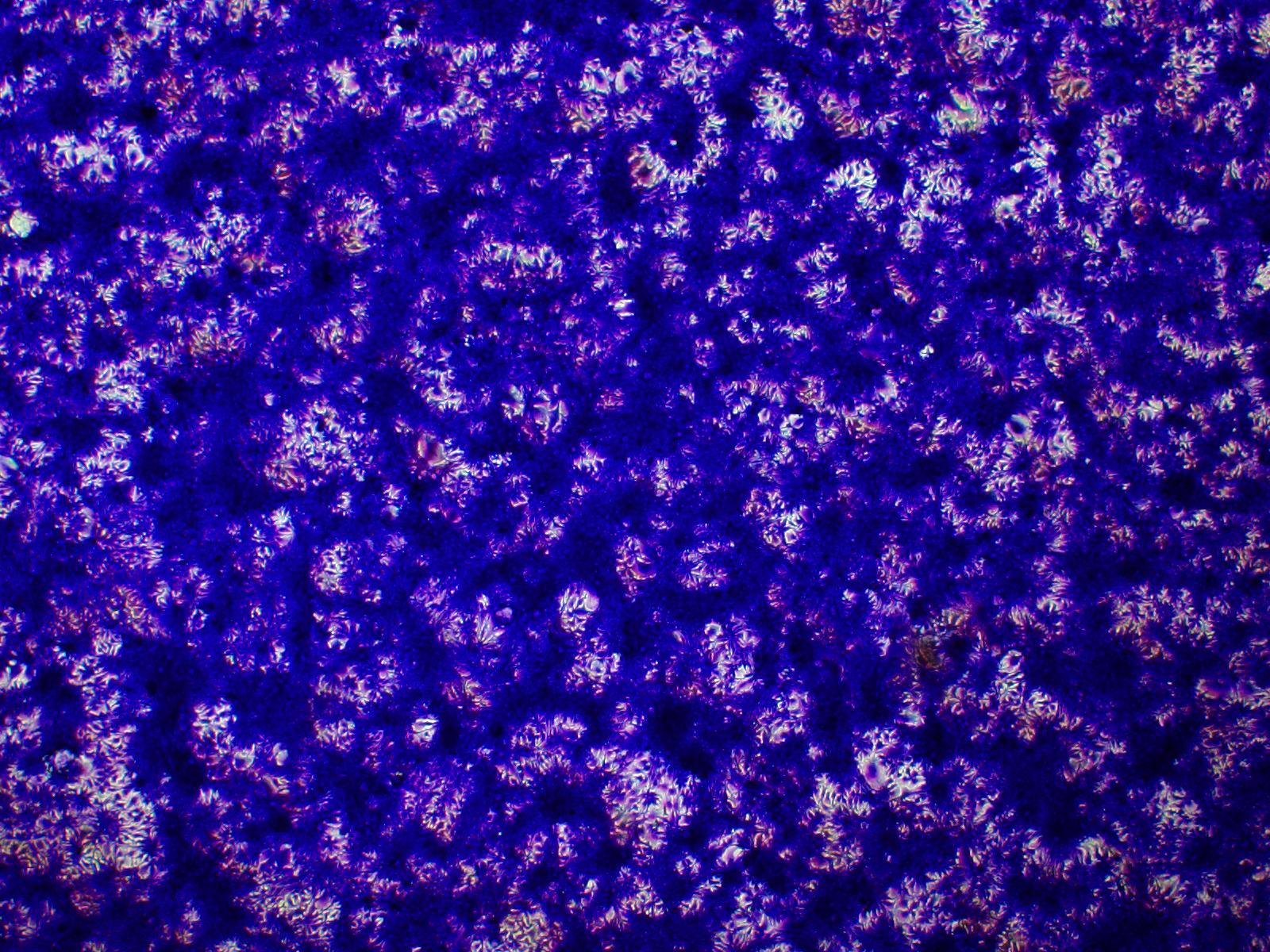

Supplement: Supplementary file 3 — Additional file 2. [file 12964_2023_1355_MOESM2_ESM.zip › raw data/Figure 2/Figure 2E/Figure 2E_Hep3B_SOR 1 ╬╝M+WAY 0 ╬╝M.tif]

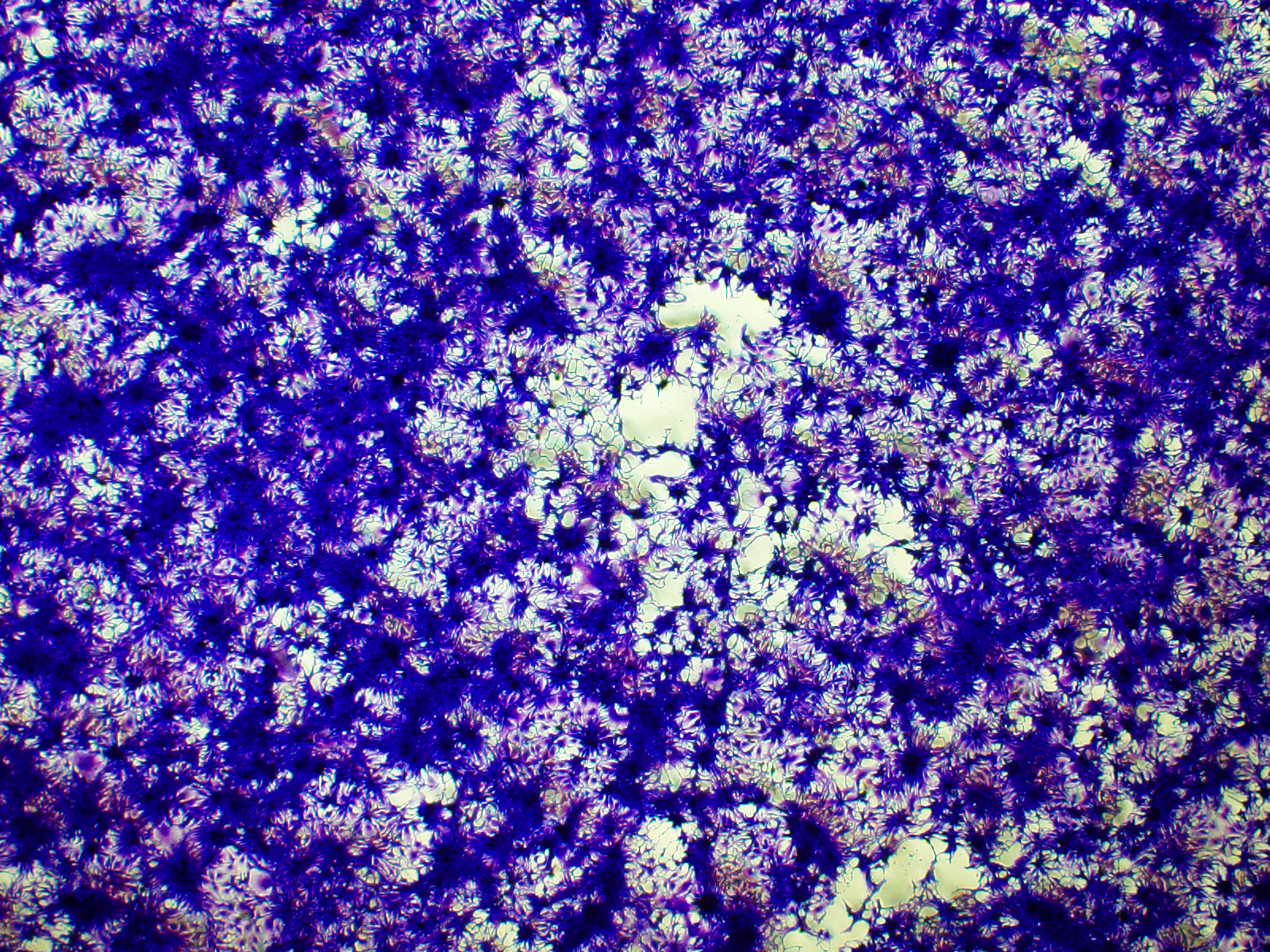

Supplement: Supplementary file 3 — Additional file 2. [file 12964_2023_1355_MOESM2_ESM.zip › raw data/Figure 2/Figure 2E/Figure 2E_Hep3B_SOR 2 ╬╝M+WAY 0 ╬╝M.tif]

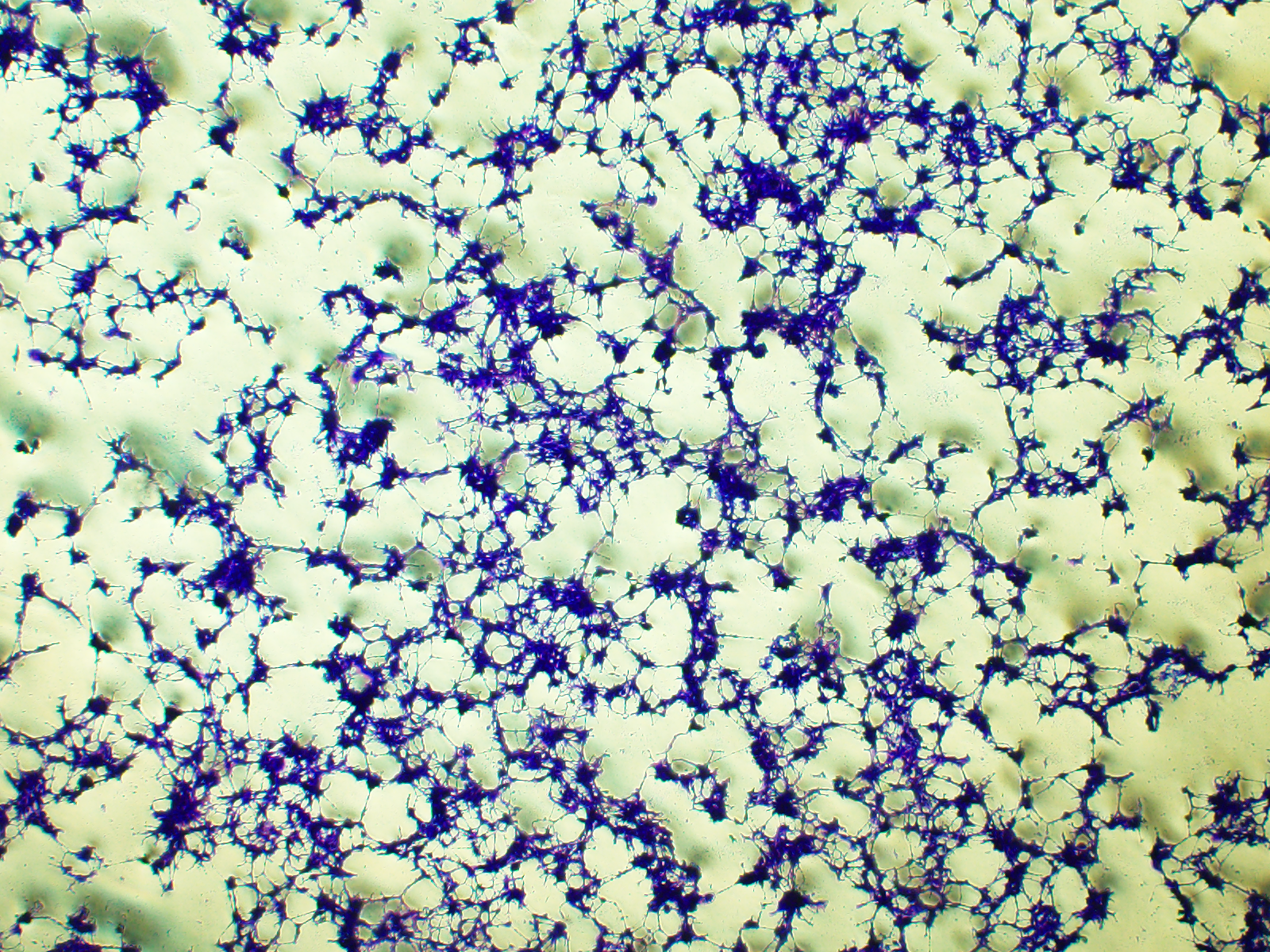

Supplement: Supplementary file 3 — Additional file 2. [file 12964_2023_1355_MOESM2_ESM.zip › raw data/Figure 2/Figure 2E/Figure 2E_Hep3B_SOR 4 ╬╝M+WAY 0 ╬╝M.tif]

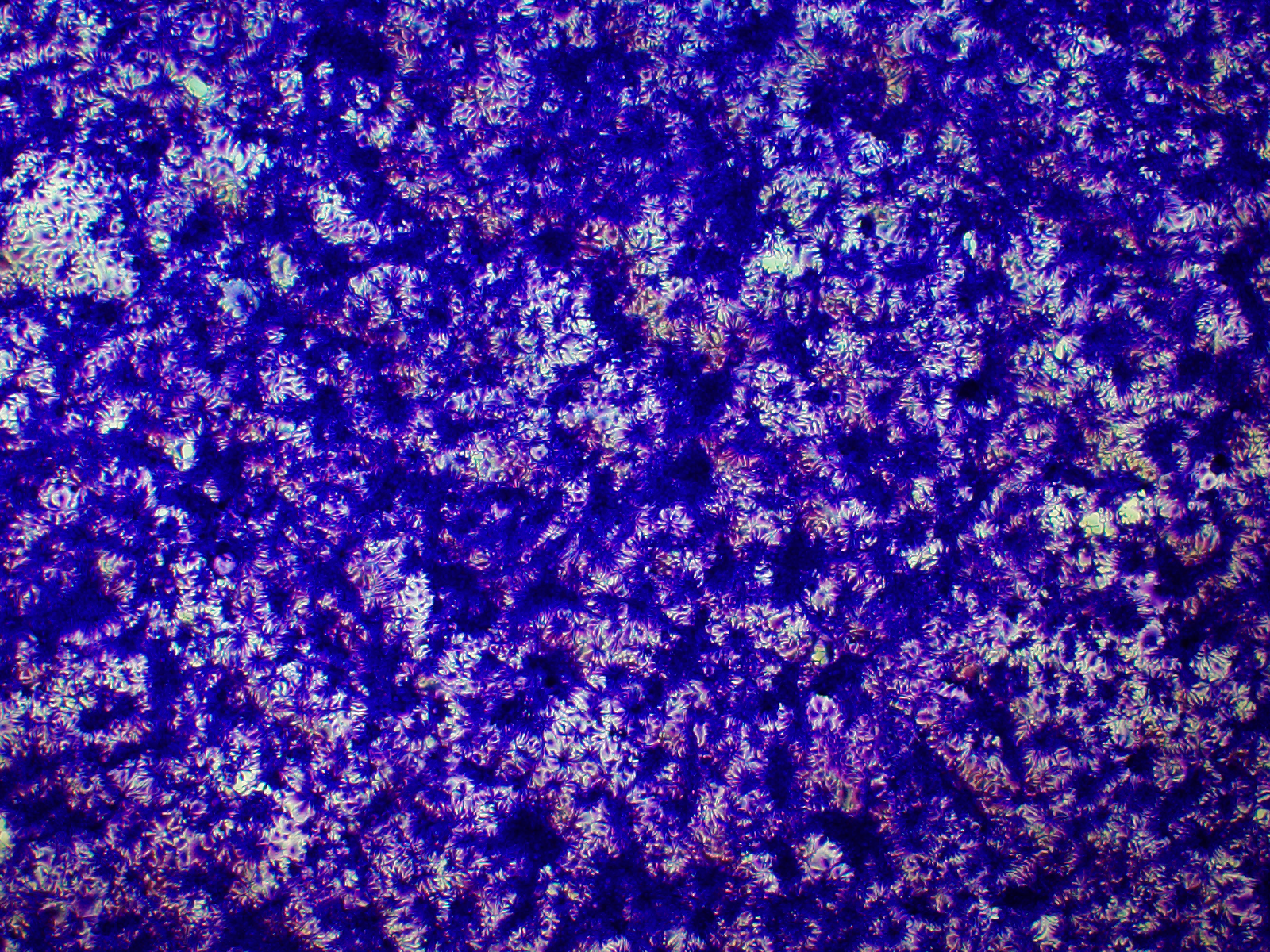

Supplement: Supplementary file 3 — Additional file 2. [file 12964_2023_1355_MOESM2_ESM.zip › raw data/Figure 2/Figure 2E/Figure 2E_Hep3B_SOR 0 ╬╝M+WAY 4.5 ╬╝M.tif]

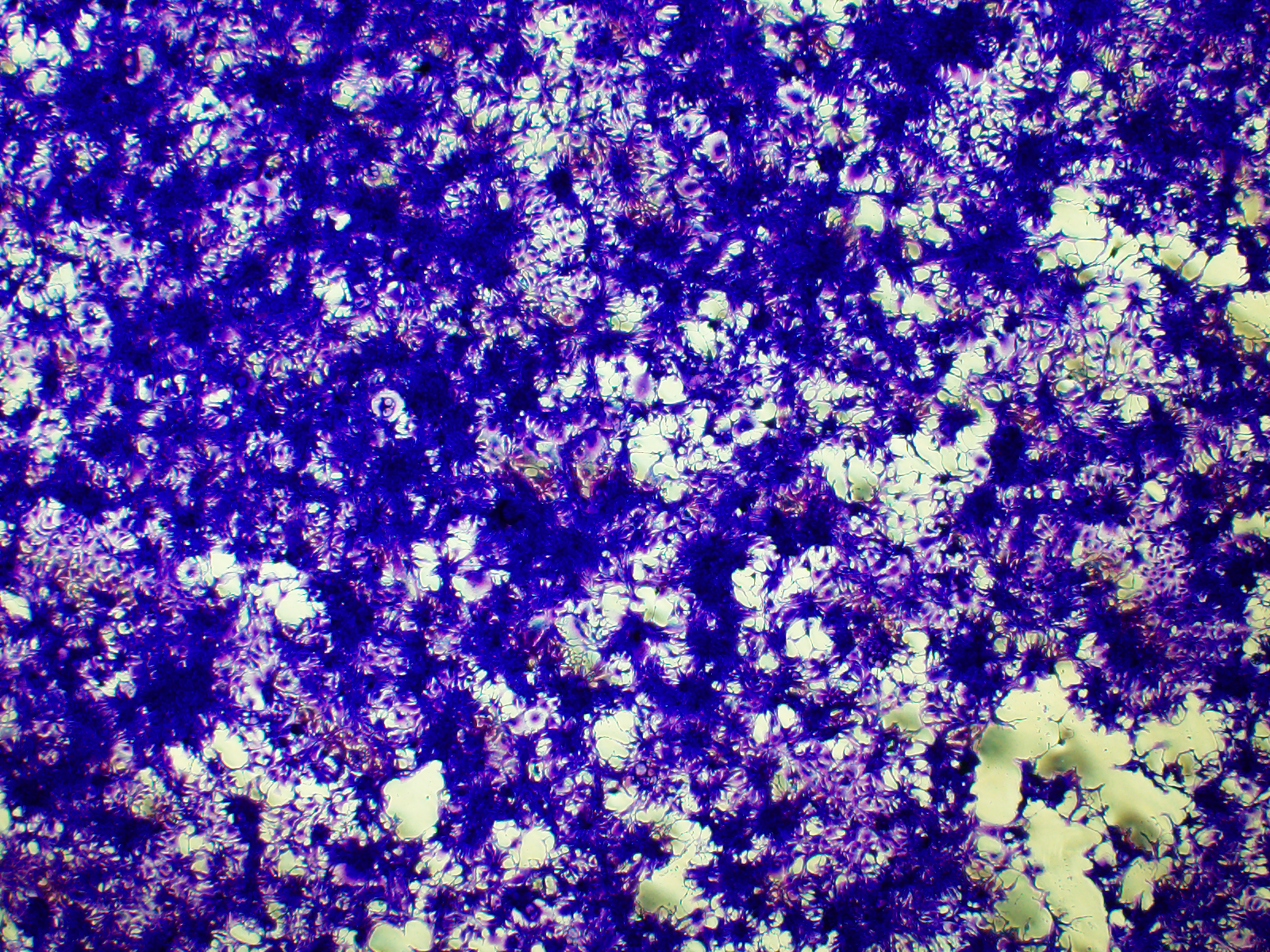

Supplement: Supplementary file 3 — Additional file 2. [file 12964_2023_1355_MOESM2_ESM.zip › raw data/Figure 2/Figure 2E/Figure 2E_Hep3B_SOR 1 ╬╝M+WAY 4.5 ╬╝M.tif]

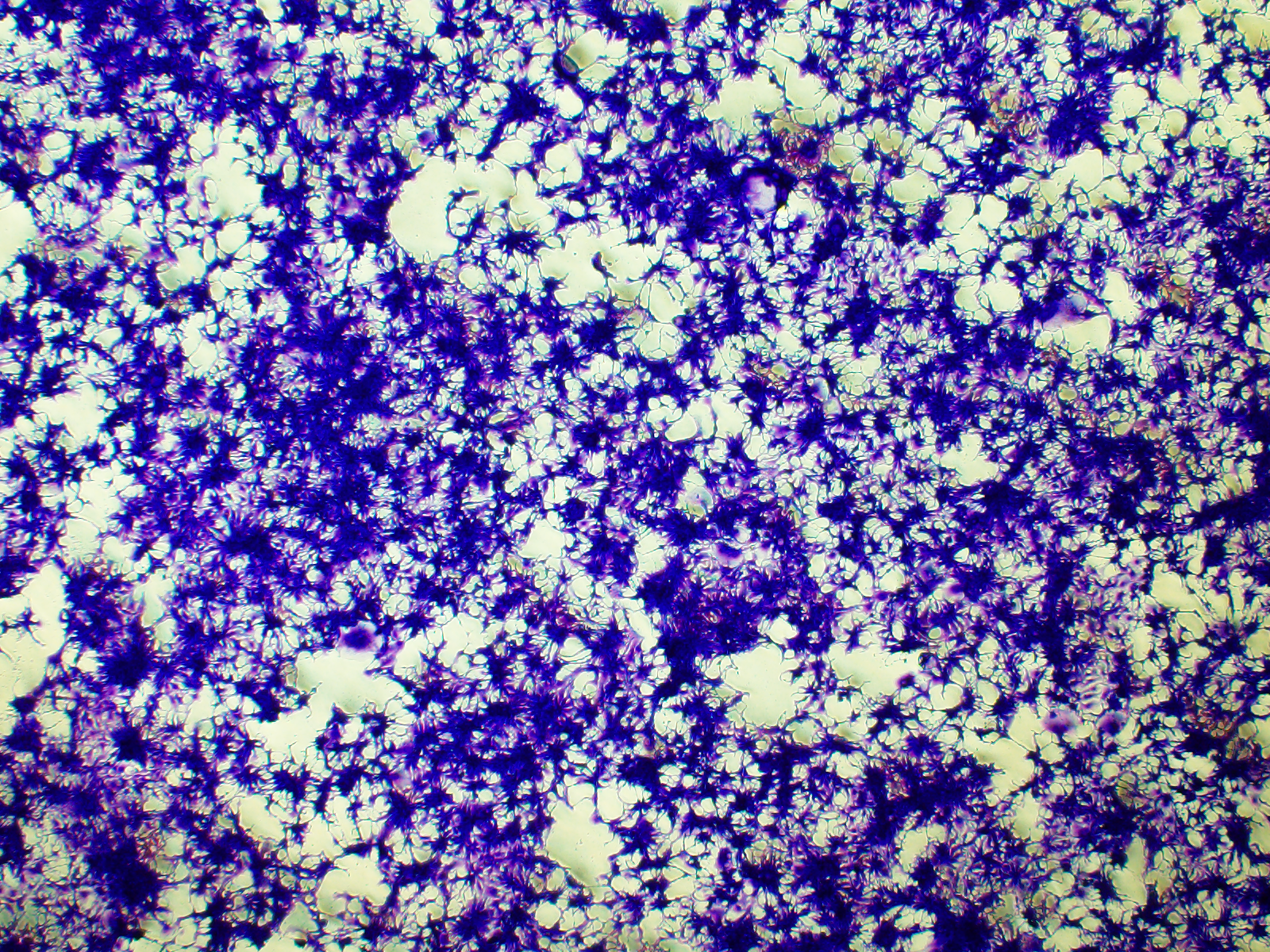

Supplement: Supplementary file 3 — Additional file 2. [file 12964_2023_1355_MOESM2_ESM.zip › raw data/Figure 2/Figure 2E/Figure 2E_Hep3B_SOR 2 ╬╝M+WAY 4.5 ╬╝M.tif]

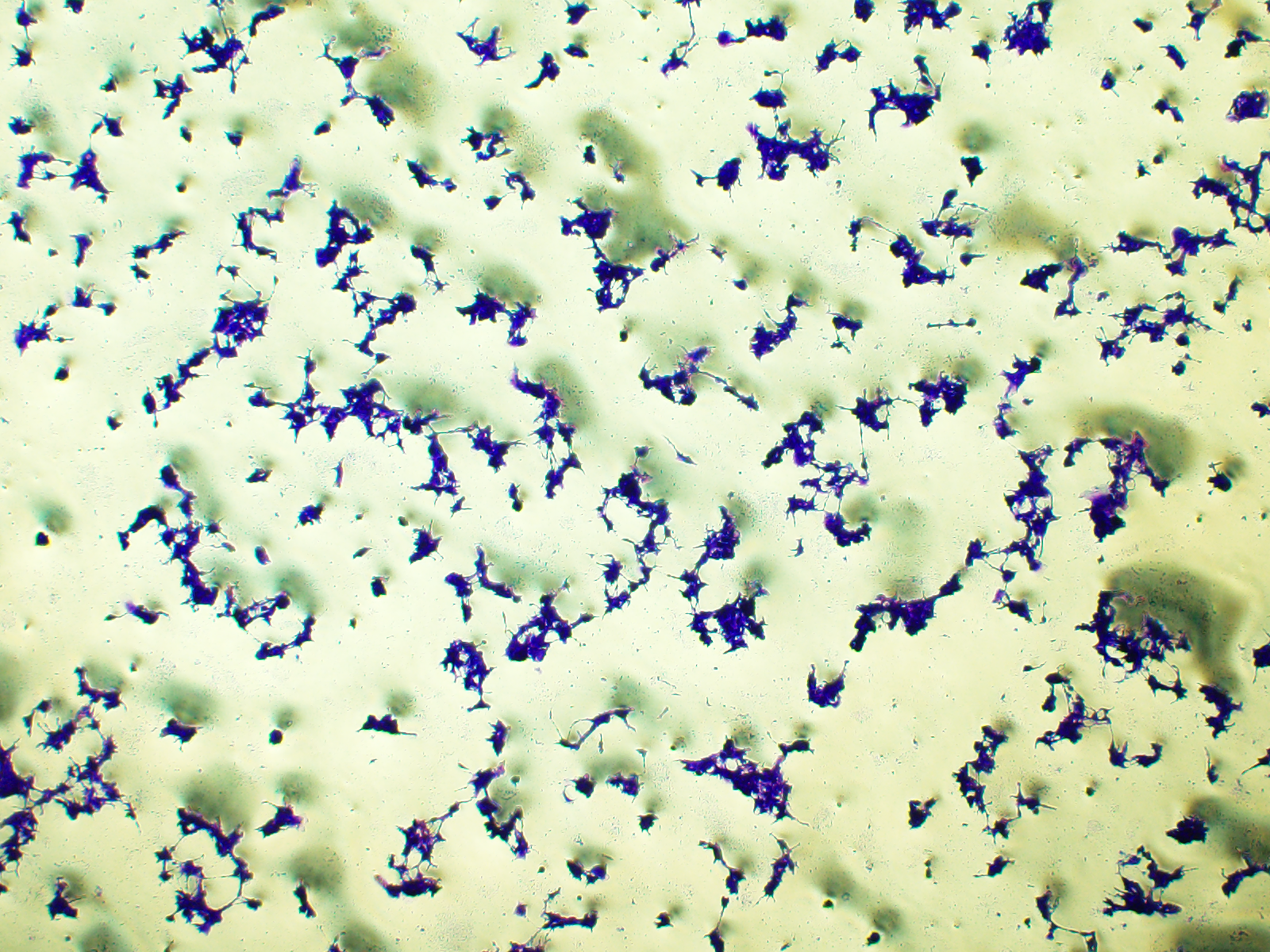

Supplement: Supplementary file 3 — Additional file 2. [file 12964_2023_1355_MOESM2_ESM.zip › raw data/Figure 2/Figure 2E/Figure 2E_Hep3B_SOR 4 ╬╝M+WAY 4.5 ╬╝M.tif]

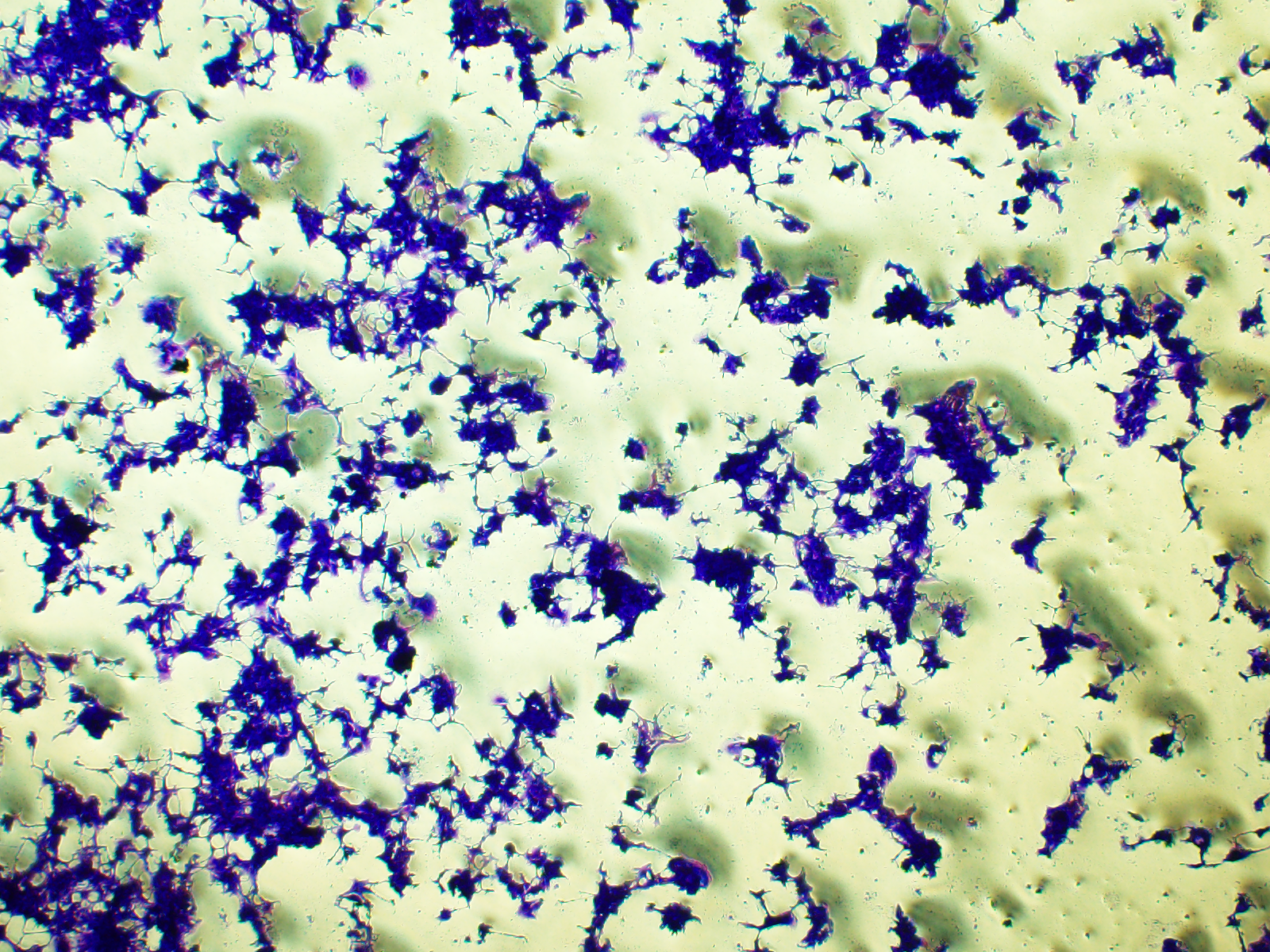

Supplement: Supplementary file 3 — Additional file 2. [file 12964_2023_1355_MOESM2_ESM.zip › raw data/Figure 2/Figure 2E/Figure 2E_Hep3B_SOR 0 ╬╝M+WAY 9 ╬╝M.tif]

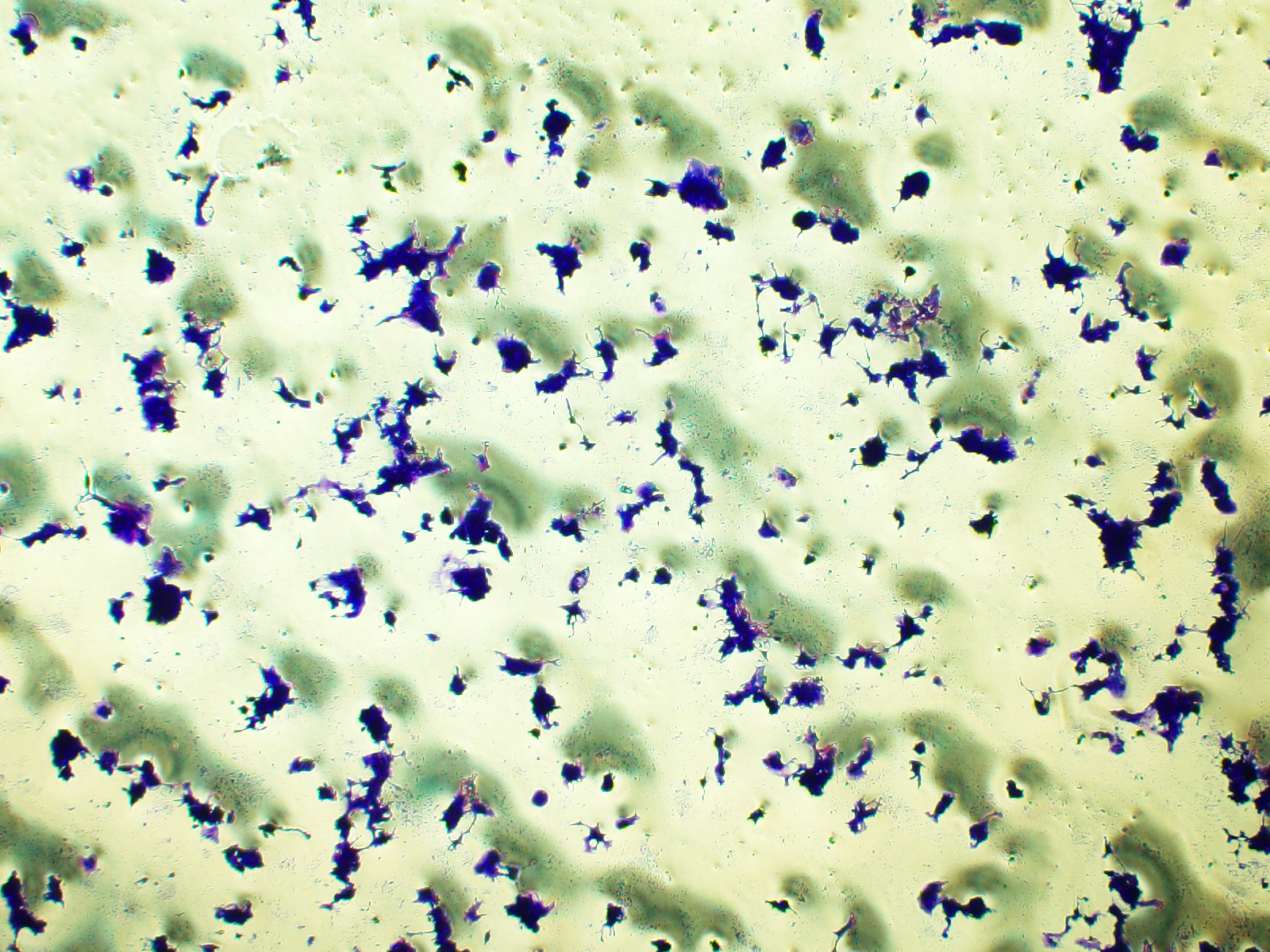

Supplement: Supplementary file 3 — Additional file 2. [file 12964_2023_1355_MOESM2_ESM.zip › raw data/Figure 2/Figure 2E/Figure 2E_Hep3B_SOR 1 ╬╝M+WAY 9 ╬╝M.tif]

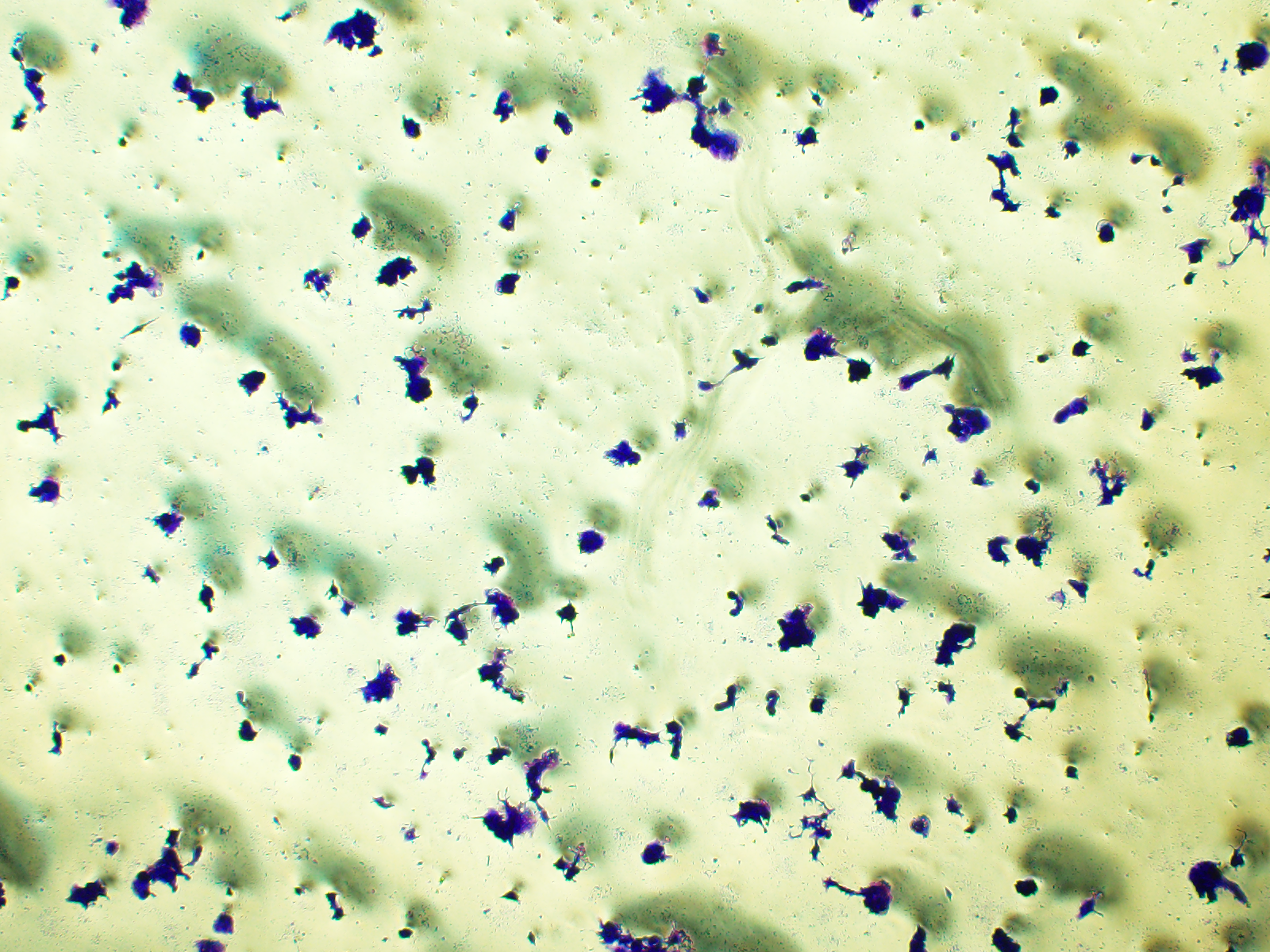

Supplement: Supplementary file 3 — Additional file 2. [file 12964_2023_1355_MOESM2_ESM.zip › raw data/Figure 2/Figure 2E/Figure 2E_Hep3B_SOR 2 ╬╝M+WAY 9 ╬╝M.tif]

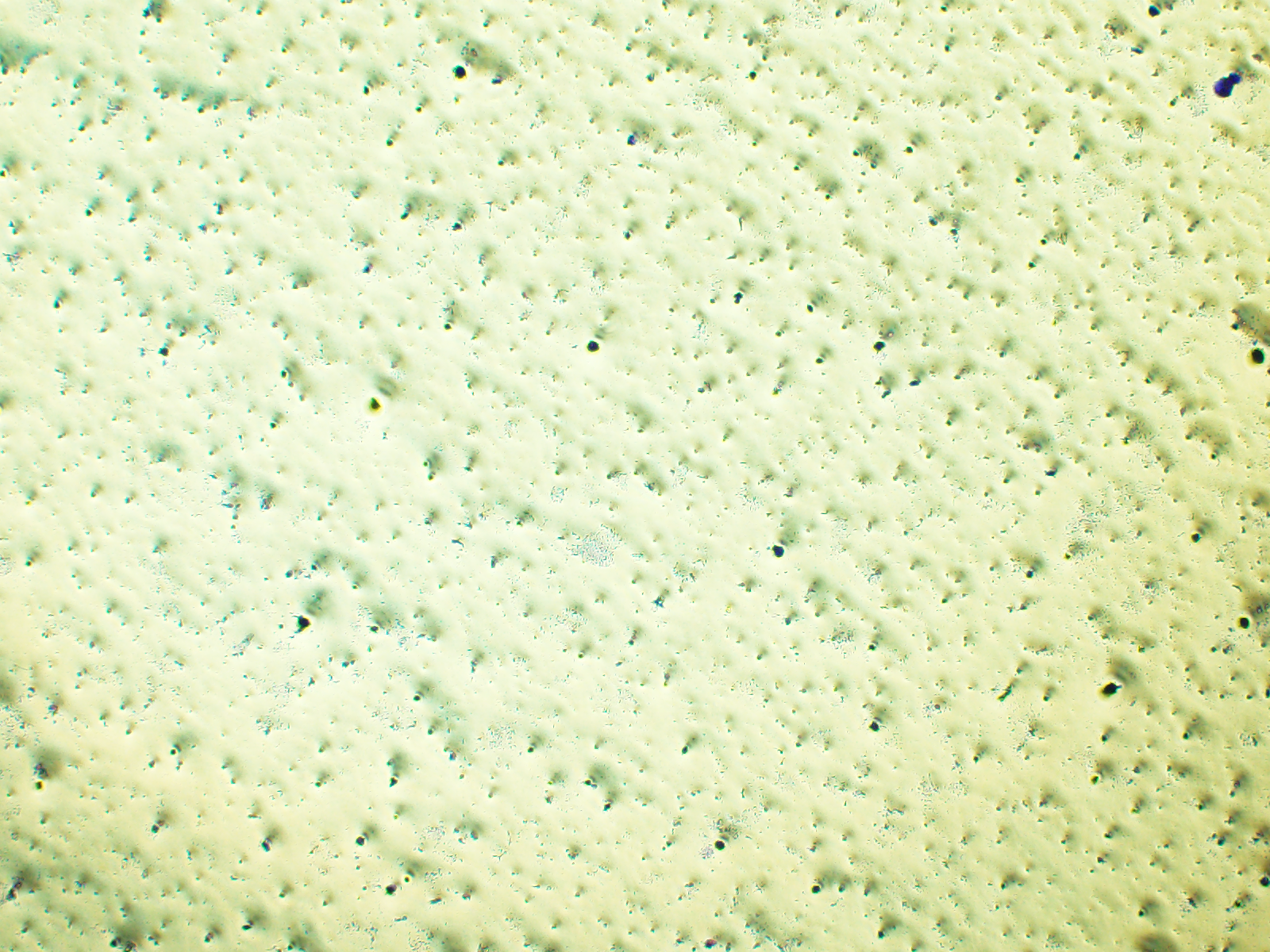

Supplement: Supplementary file 3 — Additional file 2. [file 12964_2023_1355_MOESM2_ESM.zip › raw data/Figure 2/Figure 2E/Figure 2E_Hep3B_SOR 4 ╬╝M+WAY 9 ╬╝M.tif]

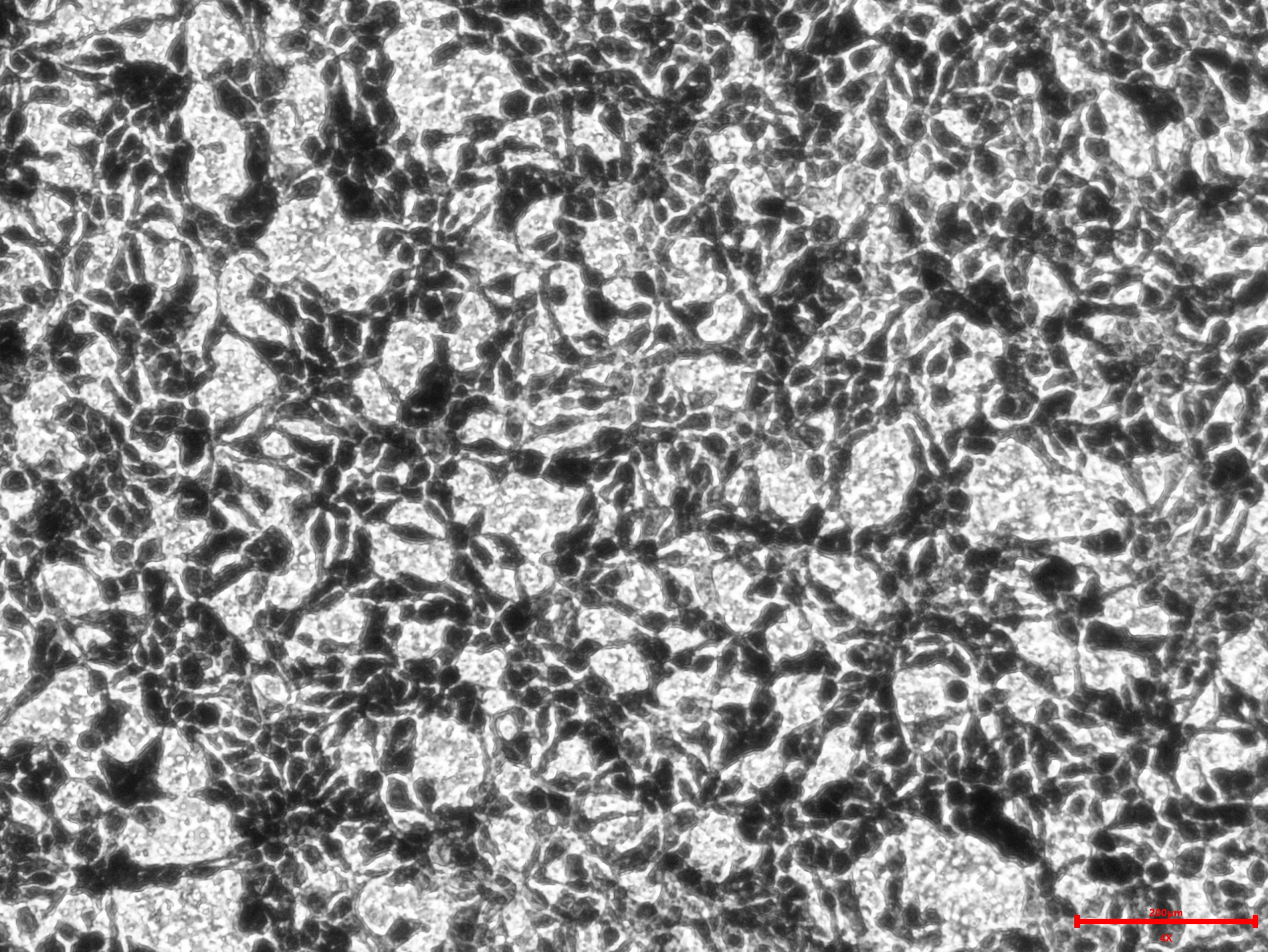

Supplement: Supplementary file 3 — Additional file 2. [file 12964_2023_1355_MOESM2_ESM.zip › raw data/Figure 2/Figure 2C/Figure 2C_Hep3B_SOR.jpg]

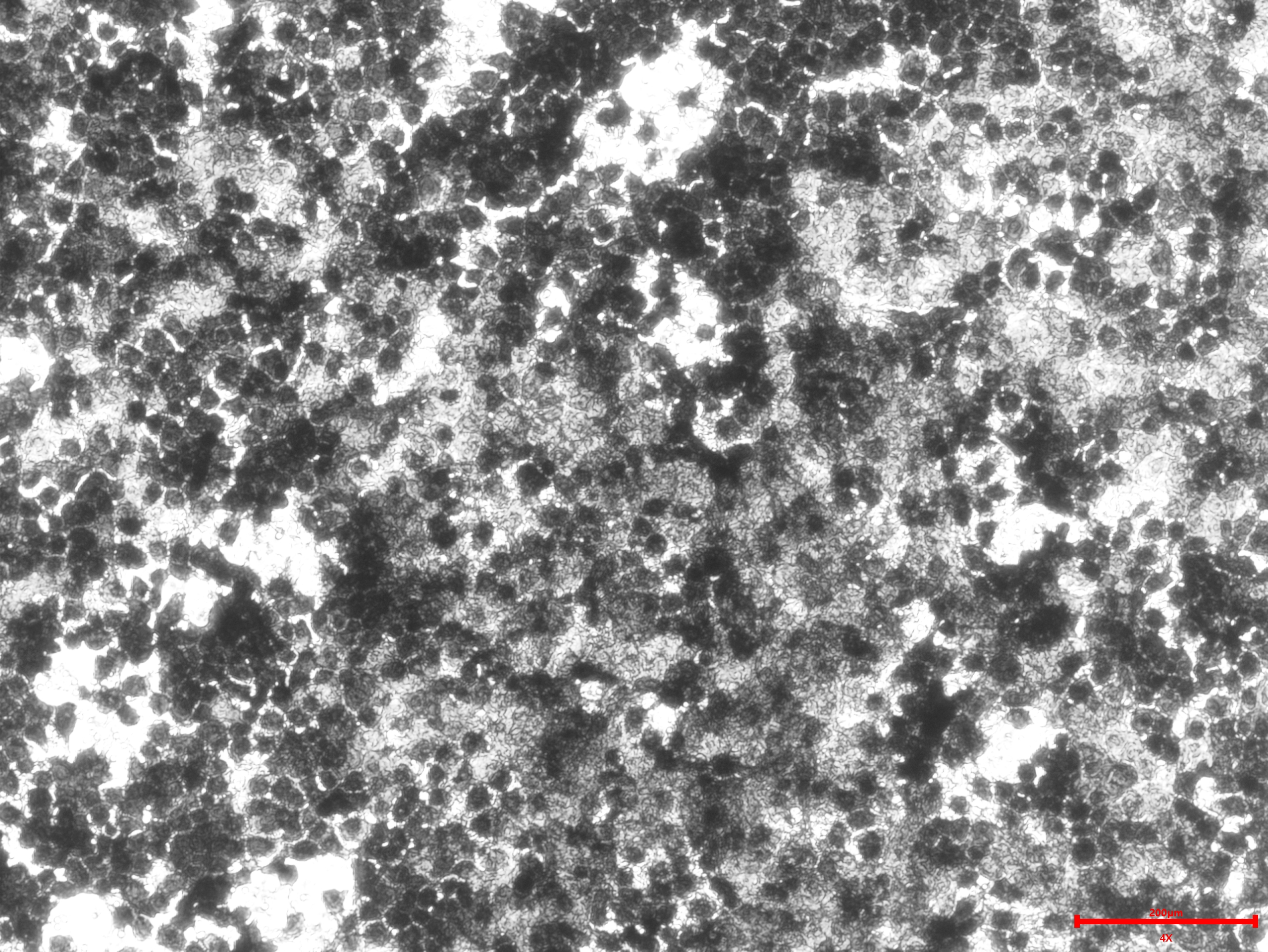

Supplement: Supplementary file 3 — Additional file 2. [file 12964_2023_1355_MOESM2_ESM.zip › raw data/Figure 2/Figure 2C/Figure 2C_Huh7_Vehicle.jpg]

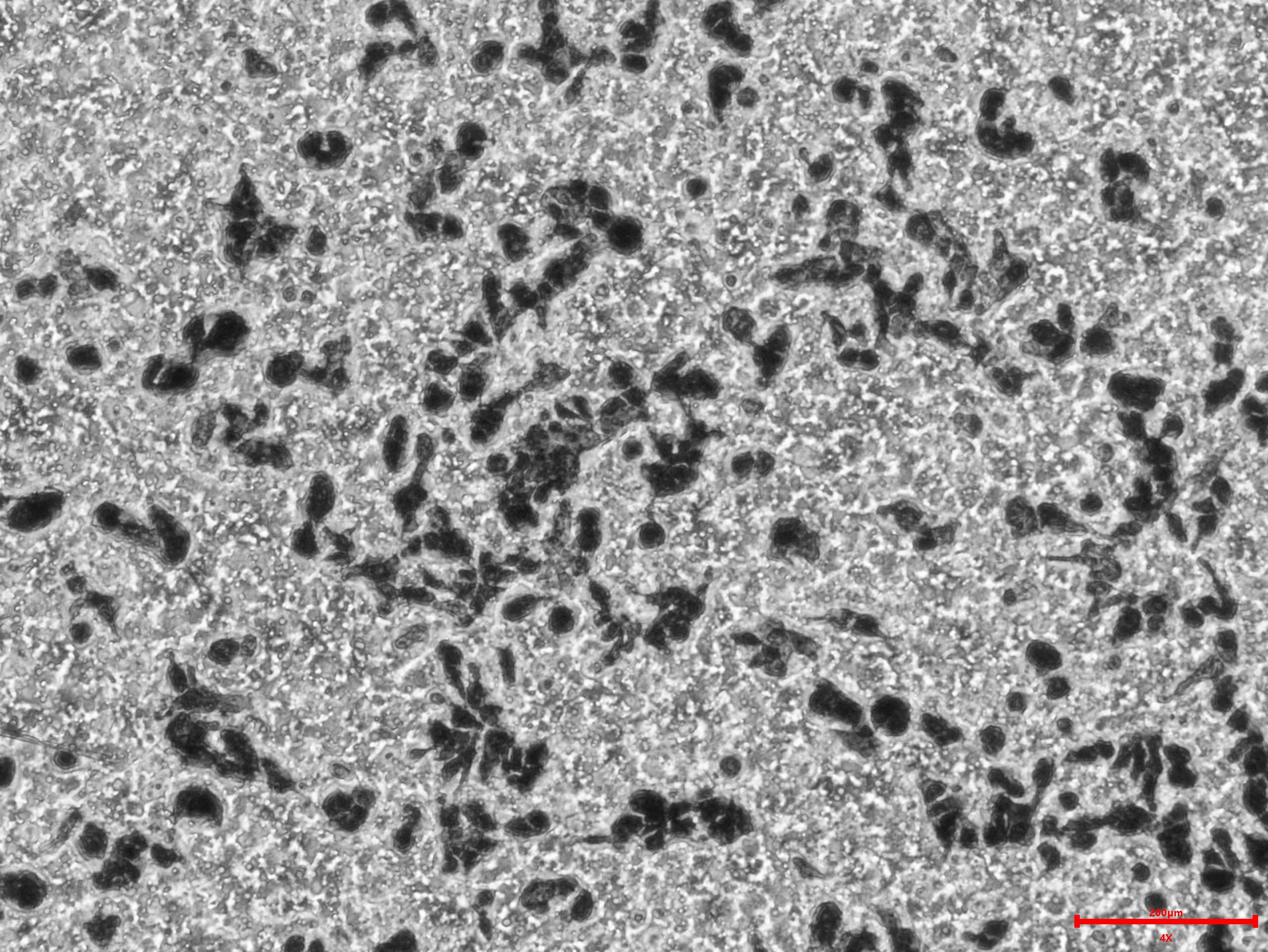

Supplement: Supplementary file 3 — Additional file 2. [file 12964_2023_1355_MOESM2_ESM.zip › raw data/Figure 2/Figure 2C/Figure 2C_Hep3B_SOR+WAY.jpg]

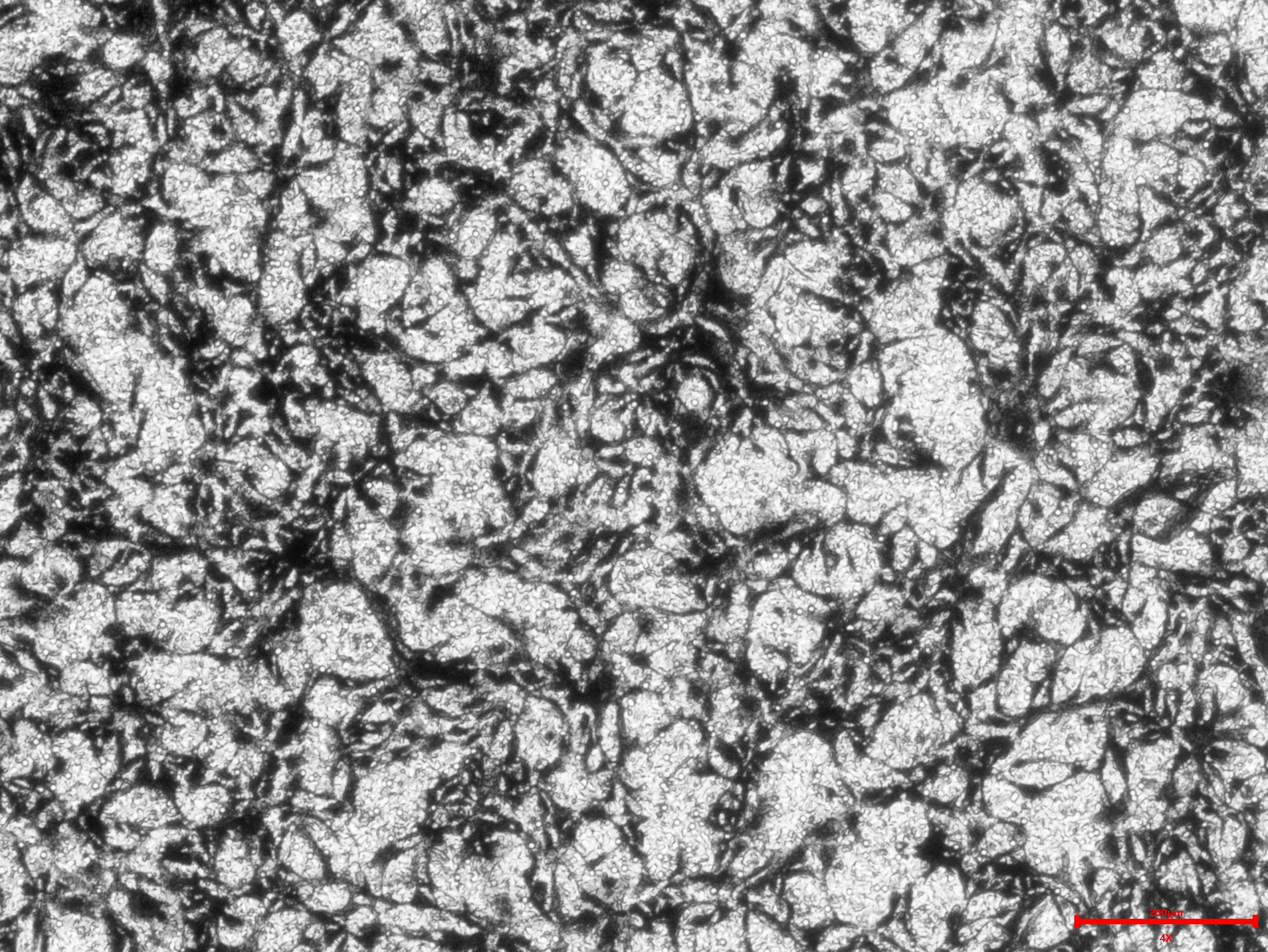

Supplement: Supplementary file 3 — Additional file 2. [file 12964_2023_1355_MOESM2_ESM.zip › raw data/Figure 2/Figure 2C/Figure 2C_Hep3B_Vehicle.jpg]

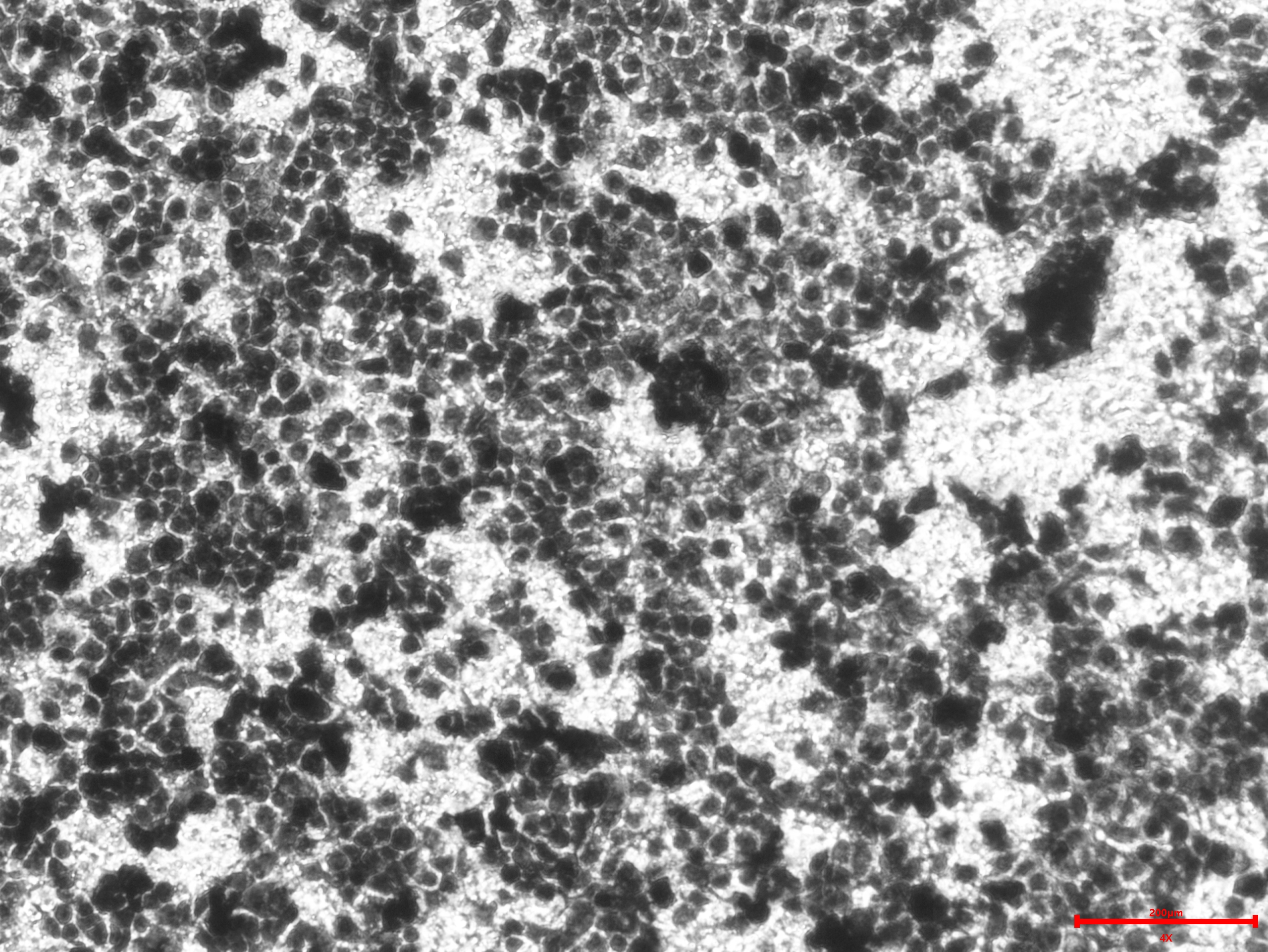

Supplement: Supplementary file 3 — Additional file 2. [file 12964_2023_1355_MOESM2_ESM.zip › raw data/Figure 2/Figure 2C/Figure 2C_Huh7_WAY.jpg]

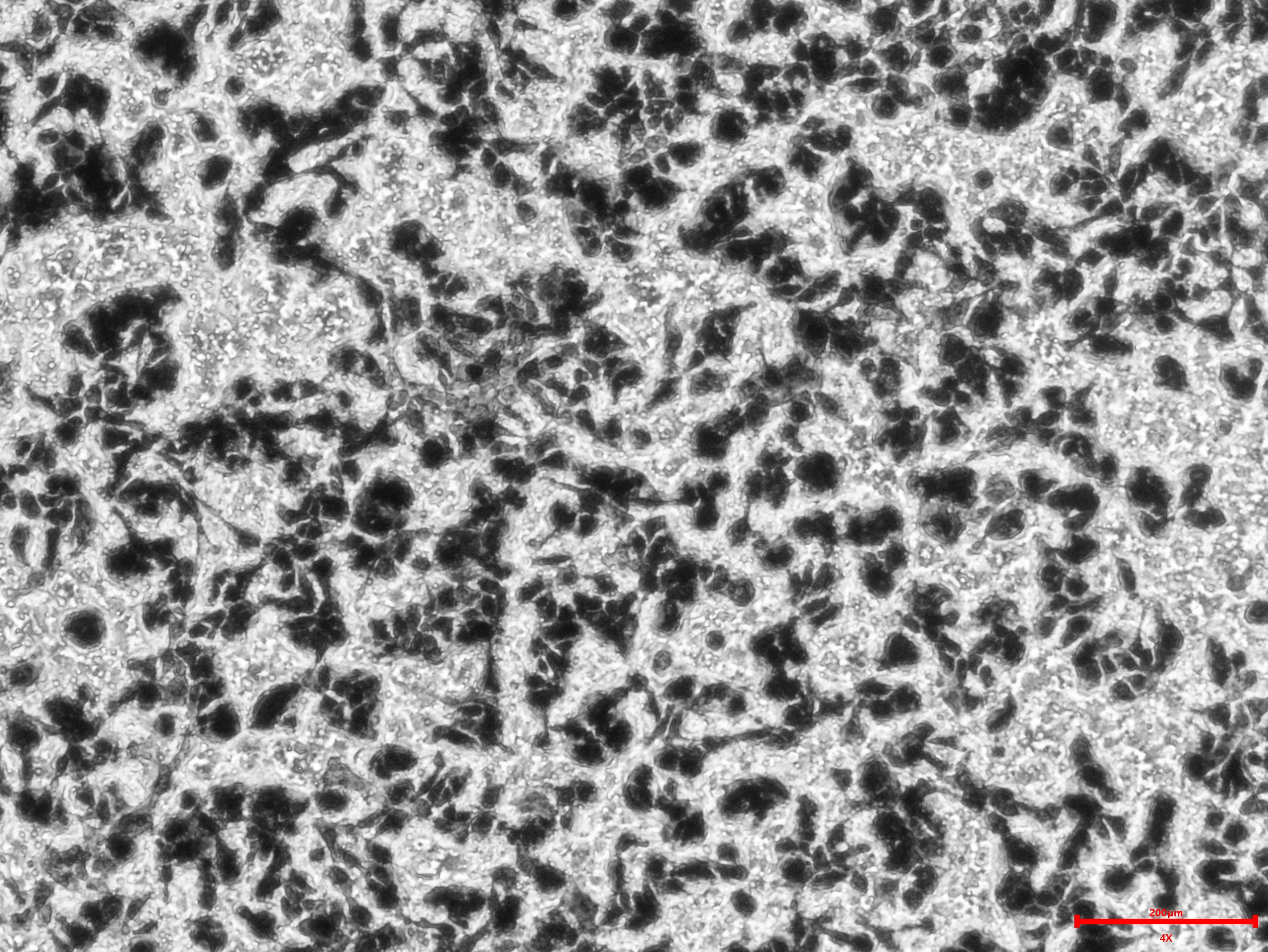

Supplement: Supplementary file 3 — Additional file 2. [file 12964_2023_1355_MOESM2_ESM.zip › raw data/Figure 2/Figure 2C/Figure 2C_Hep3B_WAY.jpg]

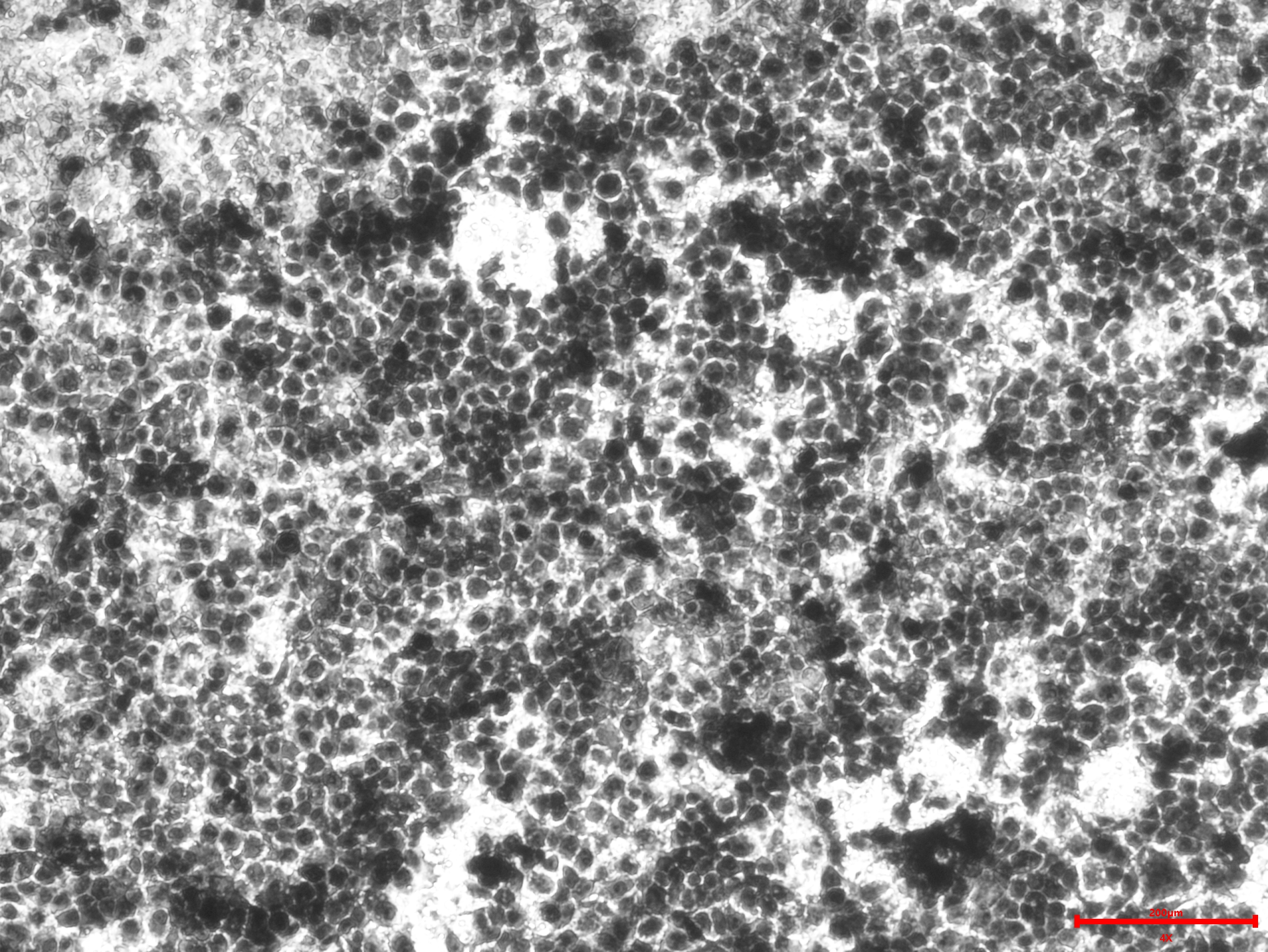

Supplement: Supplementary file 3 — Additional file 2. [file 12964_2023_1355_MOESM2_ESM.zip › raw data/Figure 2/Figure 2C/Figure 2C_Huh7_SOR.jpg]

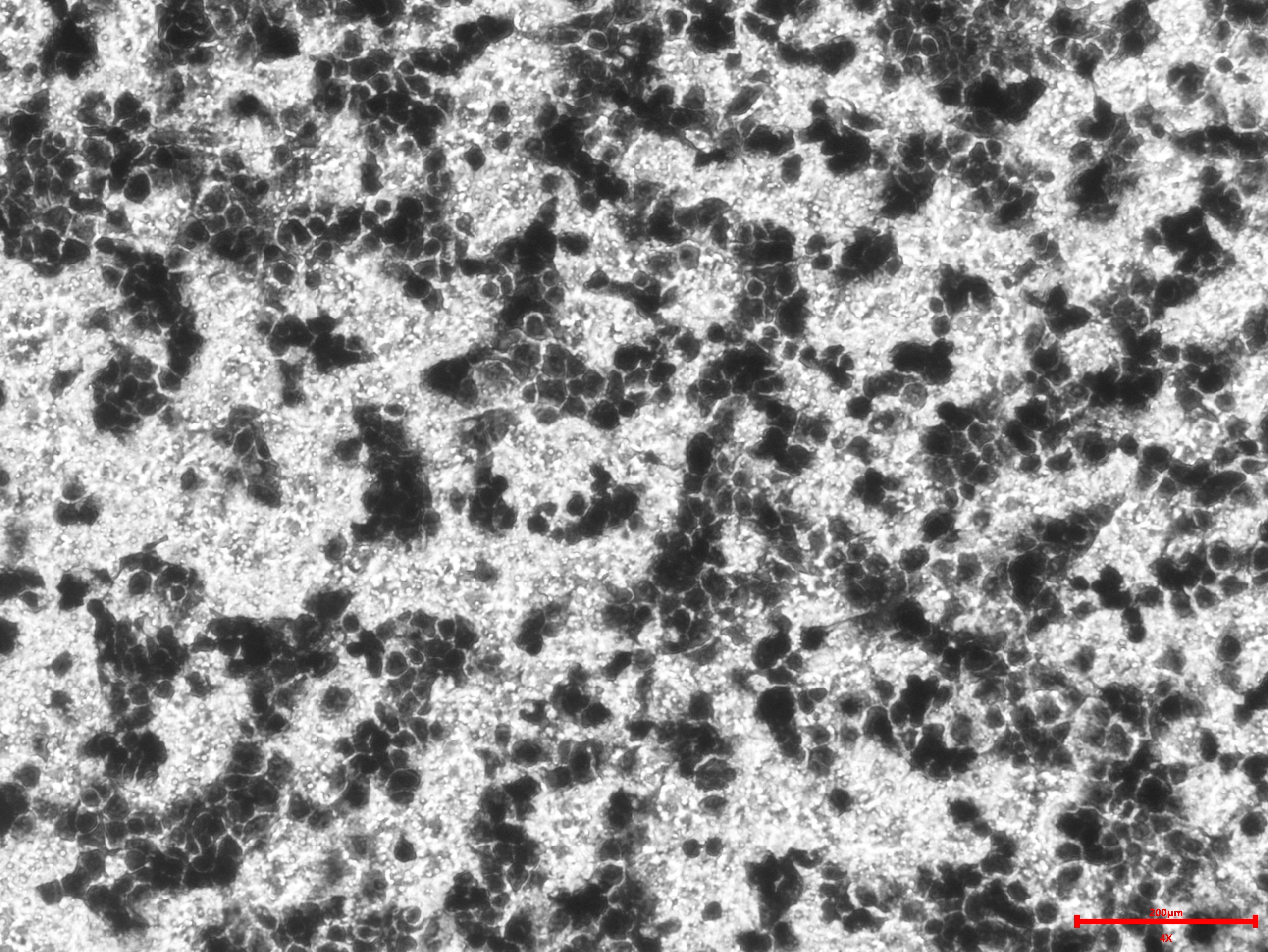

Supplement: Supplementary file 3 — Additional file 2. [file 12964_2023_1355_MOESM2_ESM.zip › raw data/Figure 2/Figure 2C/Figure 2C_Huh7_SOR+WAY.jpg]

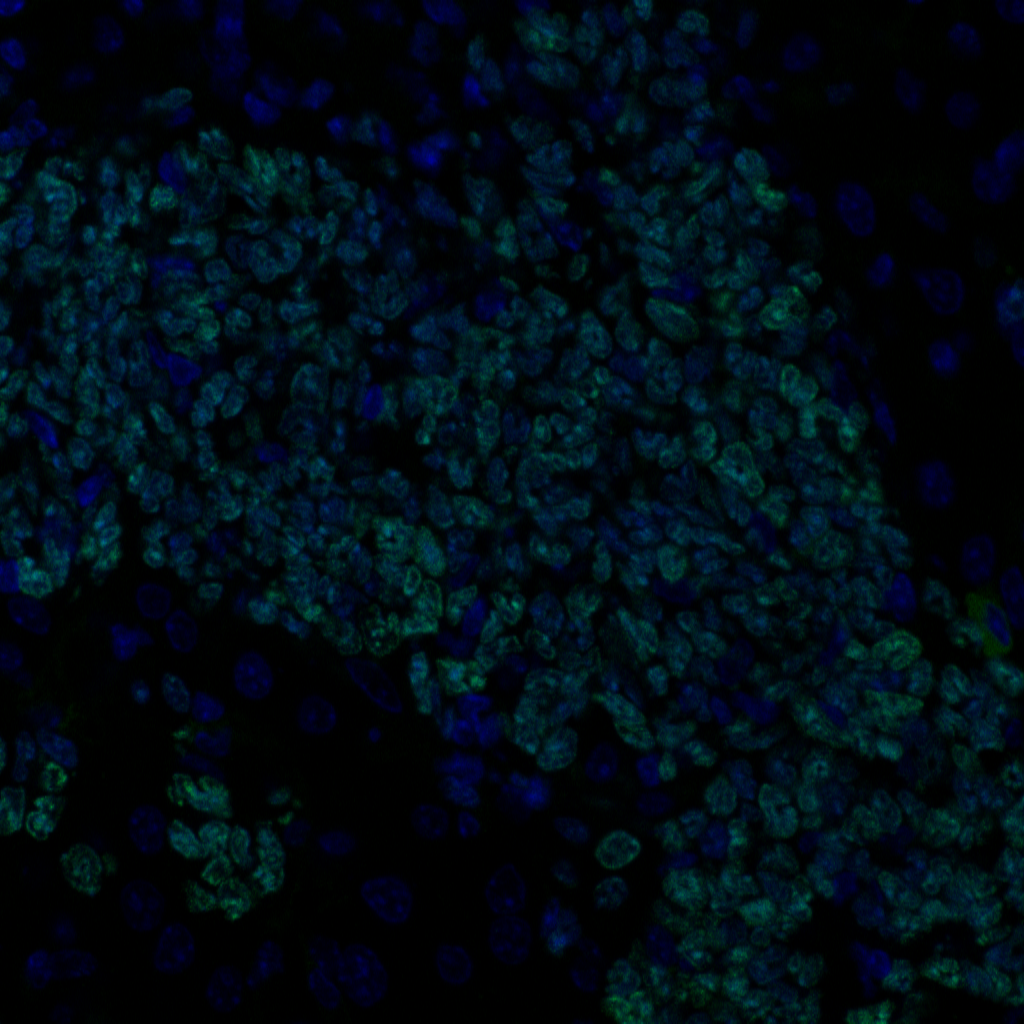

Supplement: Supplementary file 3 — Additional file 2. [file 12964_2023_1355_MOESM2_ESM.zip › raw data/Figure 5/Figure 5E/Figure 5E_Control_Ki-67.tif]

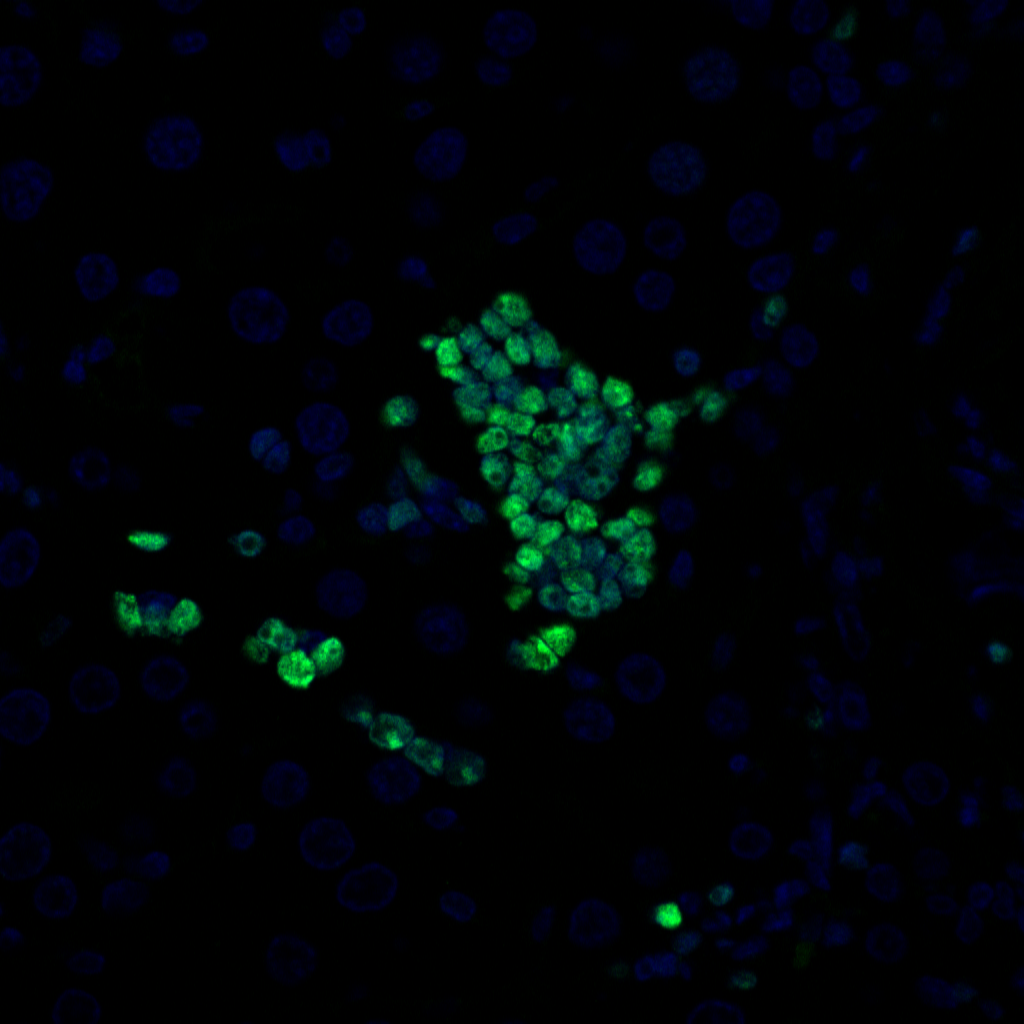

Supplement: Supplementary file 3 — Additional file 2. [file 12964_2023_1355_MOESM2_ESM.zip › raw data/Figure 5/Figure 5E/Figure 5E_SOR_Ki-67.tif]

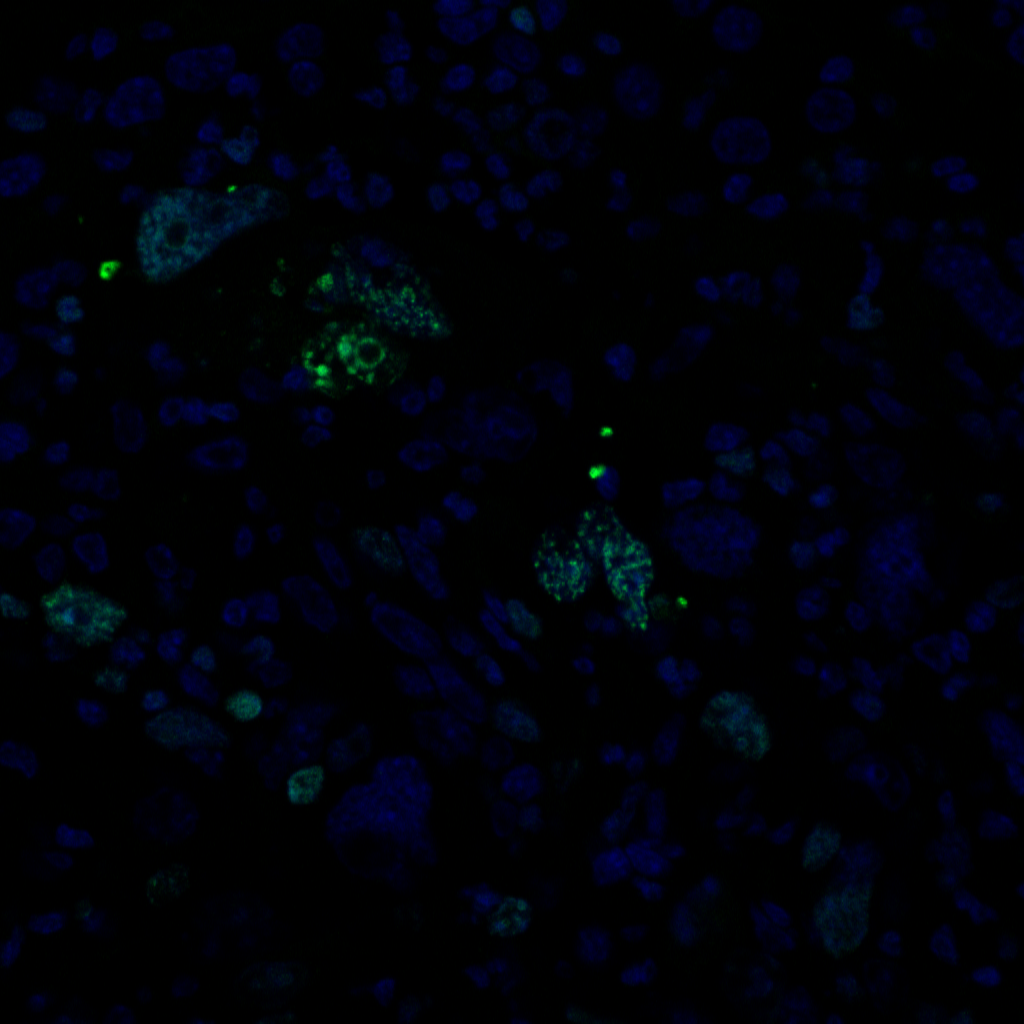

Supplement: Supplementary file 3 — Additional file 2. [file 12964_2023_1355_MOESM2_ESM.zip › raw data/Figure 5/Figure 5E/Figure 5E_WAY_Ki-67.tif]

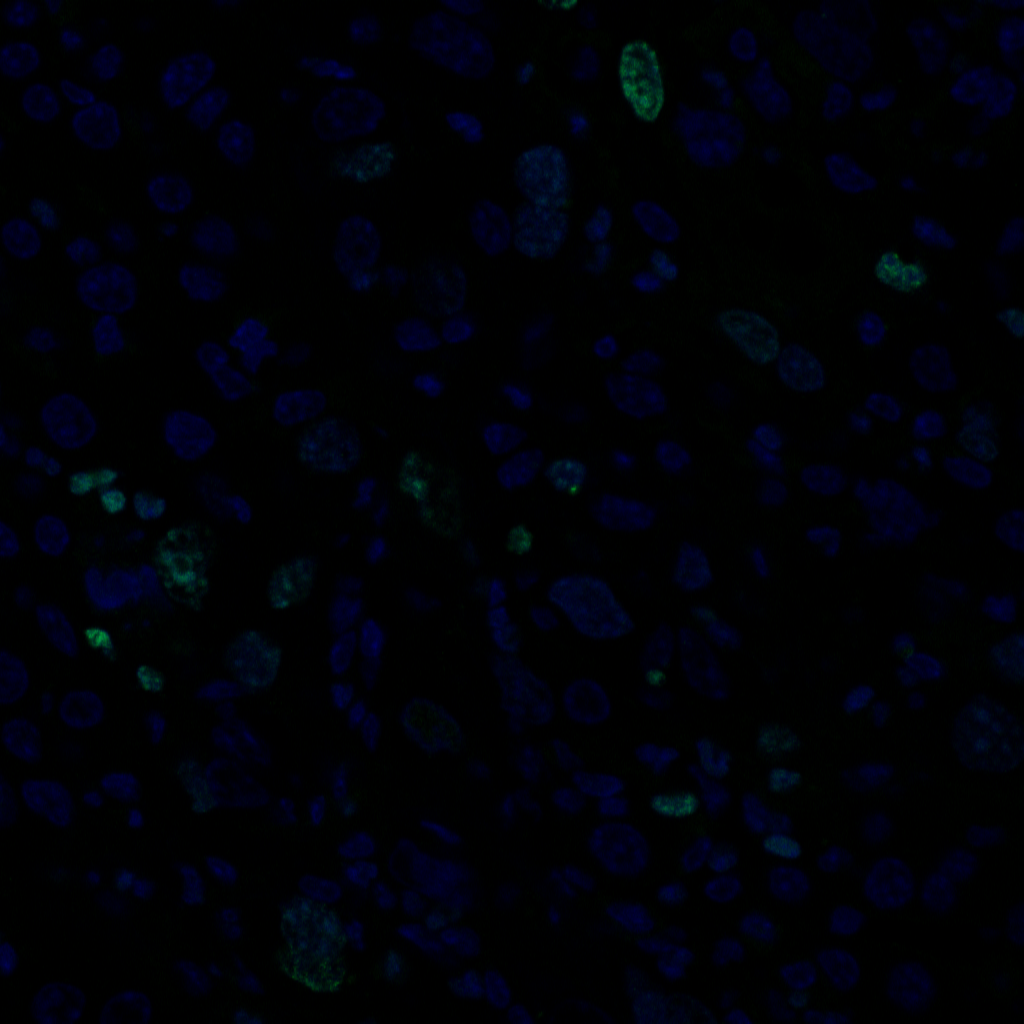

Supplement: Supplementary file 3 — Additional file 2. [file 12964_2023_1355_MOESM2_ESM.zip › raw data/Figure 5/Figure 5E/Figure 5E_SOR + WAY_Ki-67.tif]

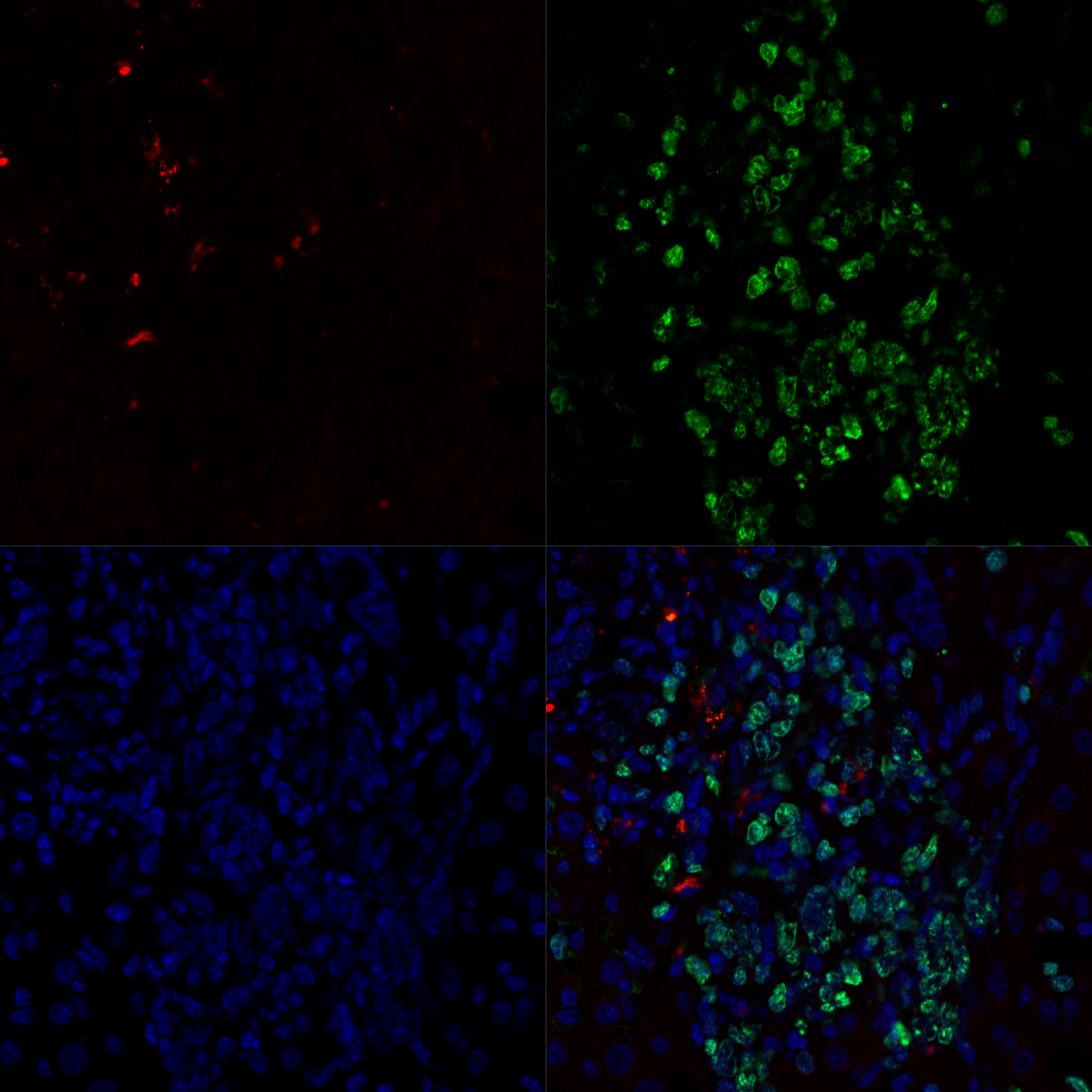

Supplement: Supplementary file 3 — Additional file 2. [file 12964_2023_1355_MOESM2_ESM.zip › raw data/Figure 5/Figure 5E/Figure 5E_Control_cleaved Caspase-3.tif]

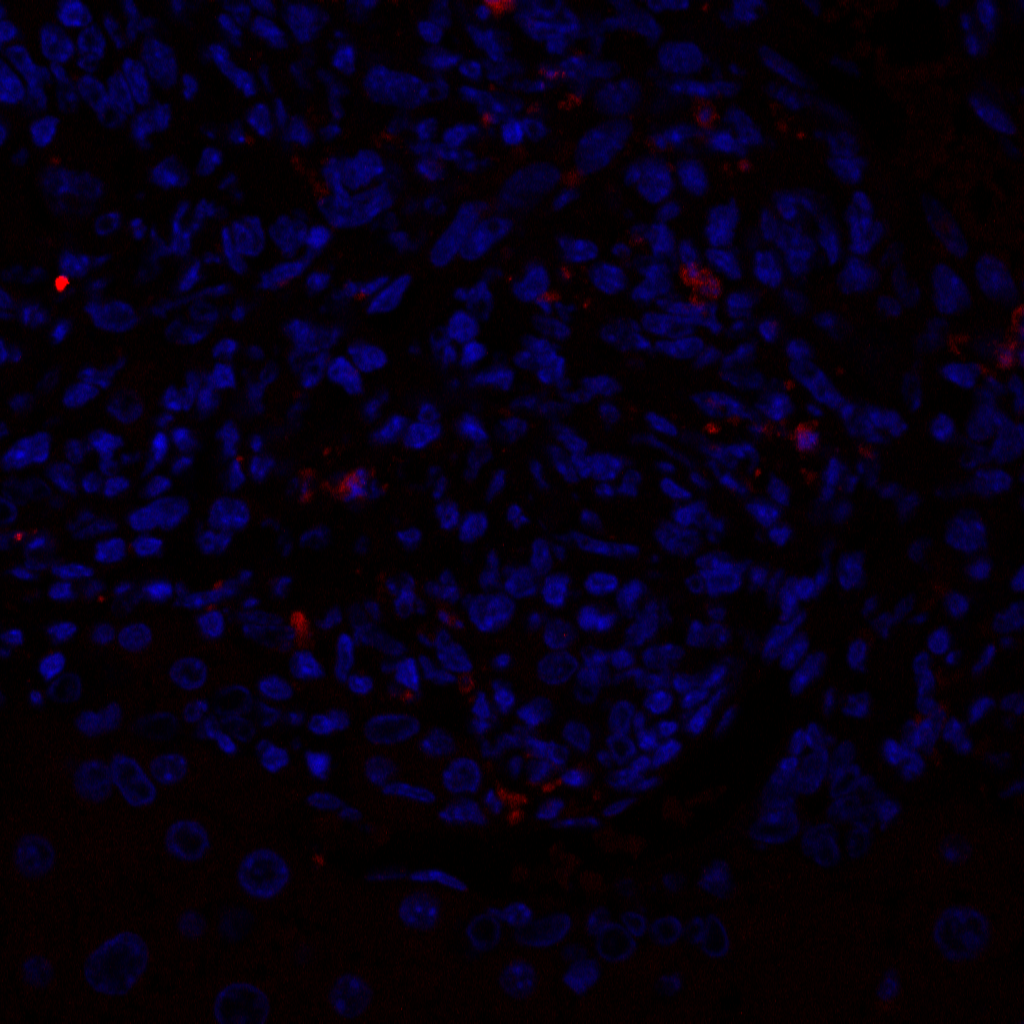

Supplement: Supplementary file 3 — Additional file 2. [file 12964_2023_1355_MOESM2_ESM.zip › raw data/Figure 5/Figure 5E/Figure 5E_SOR_cleaved Caspase-3.tif]

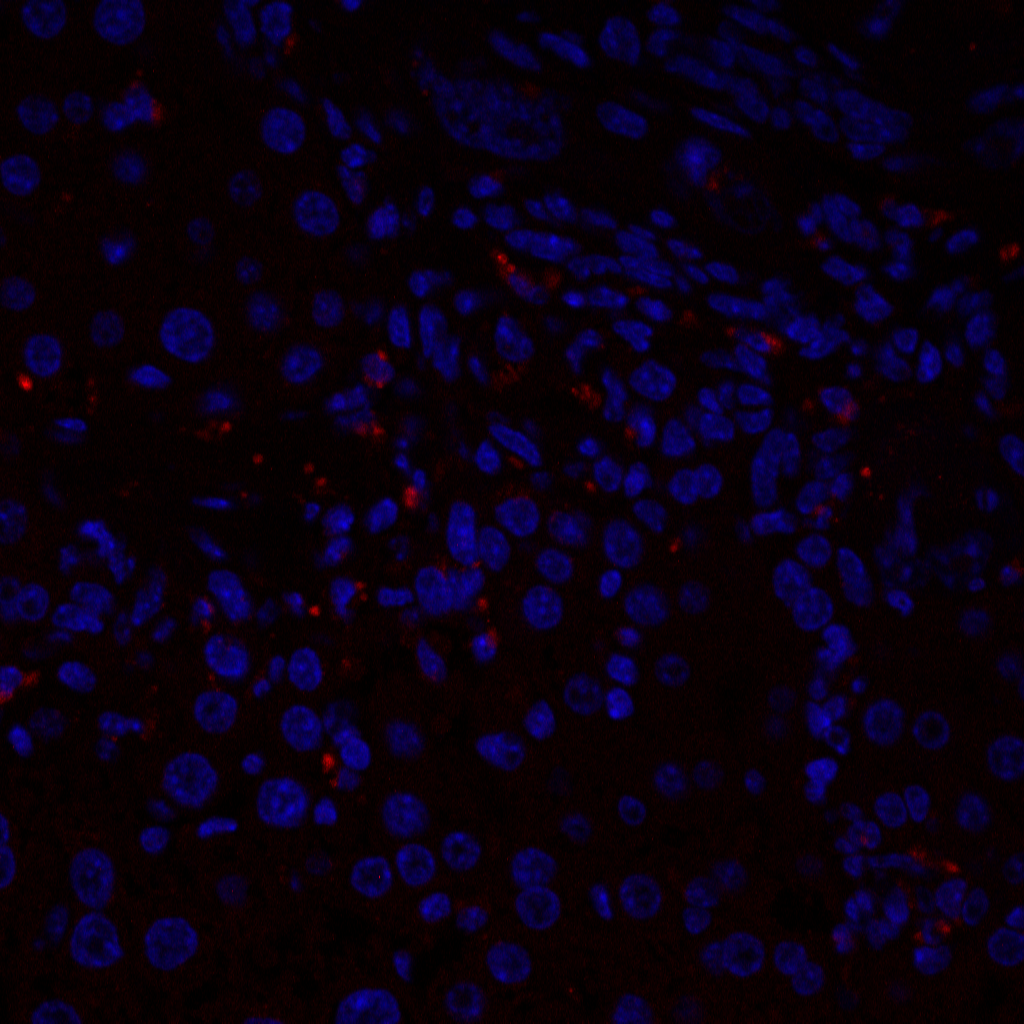

Supplement: Supplementary file 3 — Additional file 2. [file 12964_2023_1355_MOESM2_ESM.zip › raw data/Figure 5/Figure 5E/Figure 5E_SOR + WAY_cleaved Caspase-3.tif]

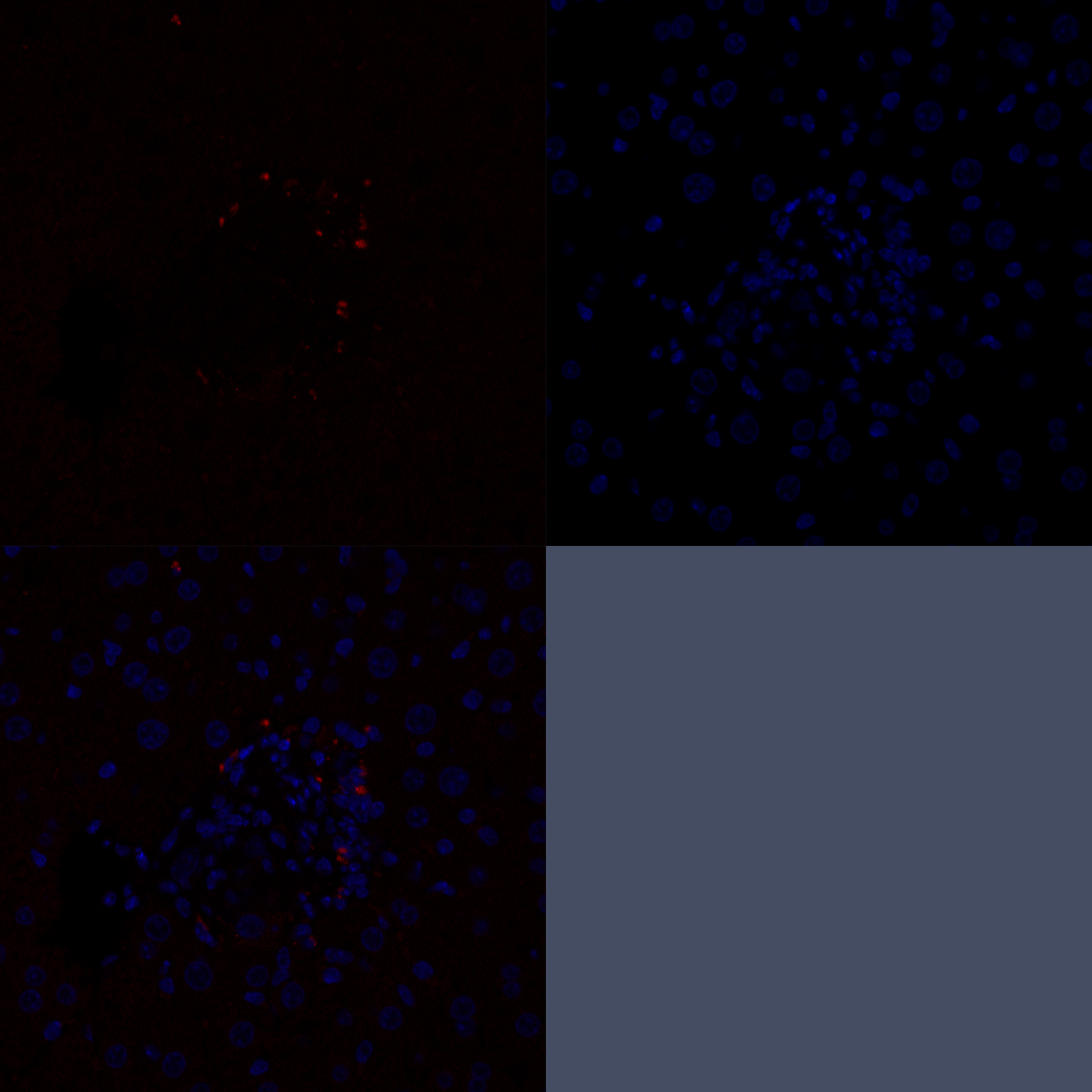

Supplement: Supplementary file 3 — Additional file 2. [file 12964_2023_1355_MOESM2_ESM.zip › raw data/Figure 5/Figure 5E/Figure 5E_WAY_cleaved Caspase-3.tif]

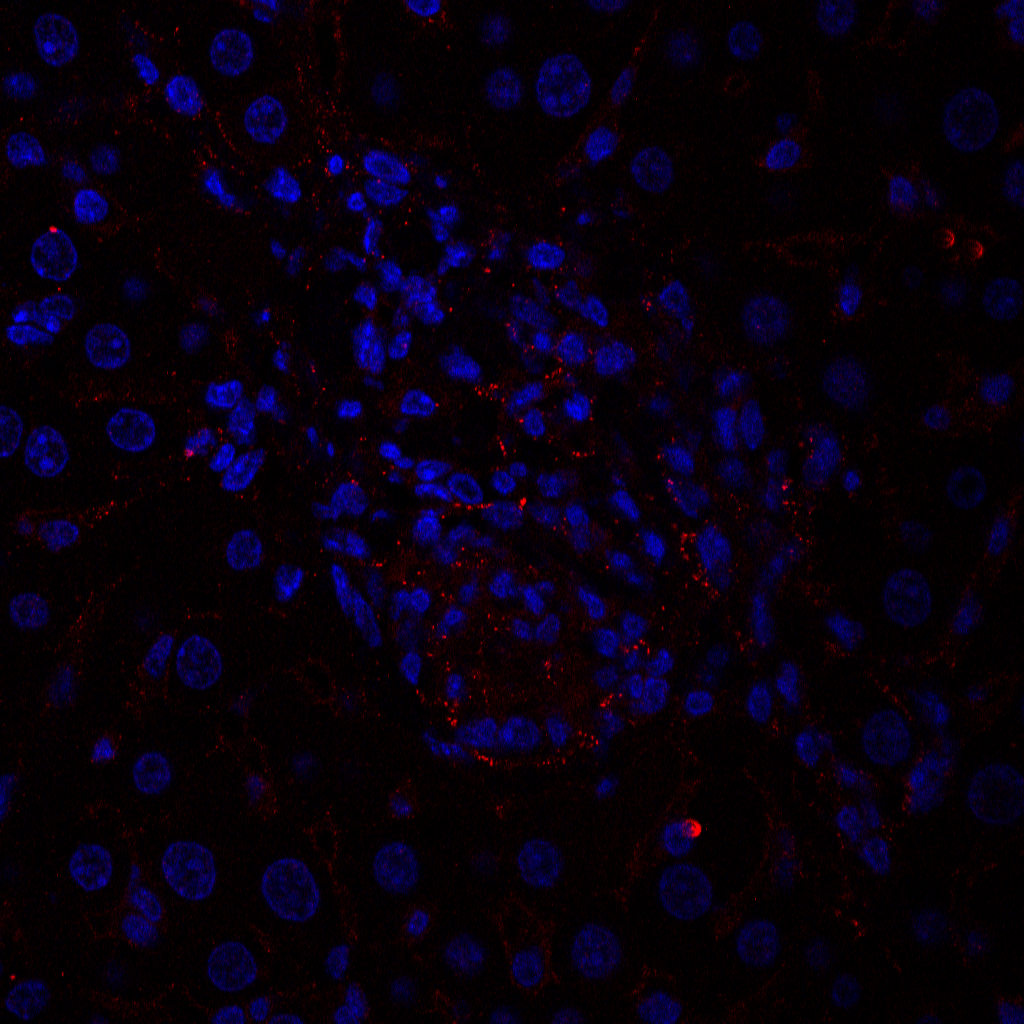

Supplement: Supplementary file 3 — Additional file 2. [file 12964_2023_1355_MOESM2_ESM.zip › raw data/Figure 5/Figure 5D/Figure 5D_Control_DKK1.tif]

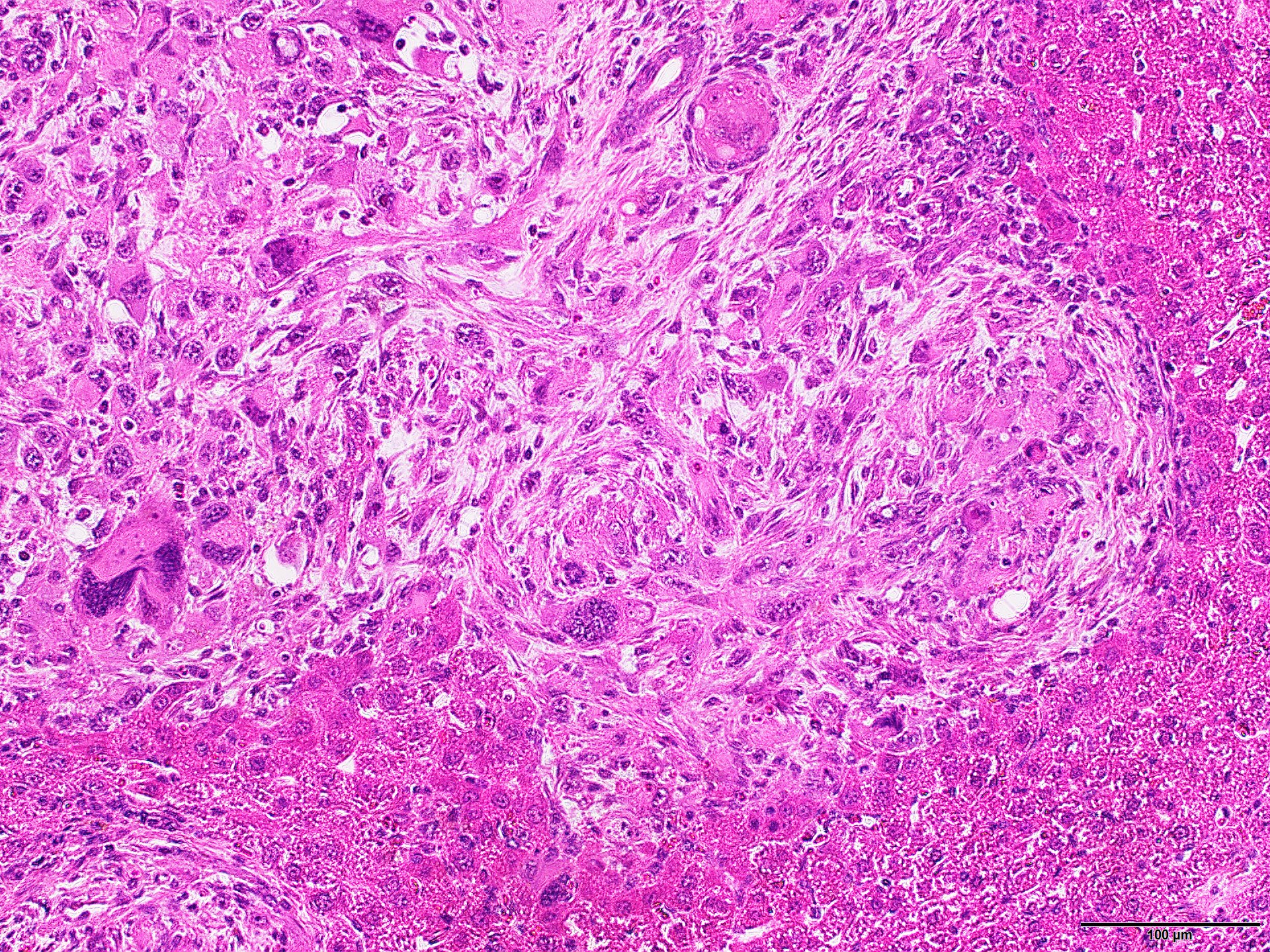

Supplement: Supplementary file 3 — Additional file 2. [file 12964_2023_1355_MOESM2_ESM.zip › raw data/Figure 5/Figure 5D/Figure 5D_Control_H&E.jpg]

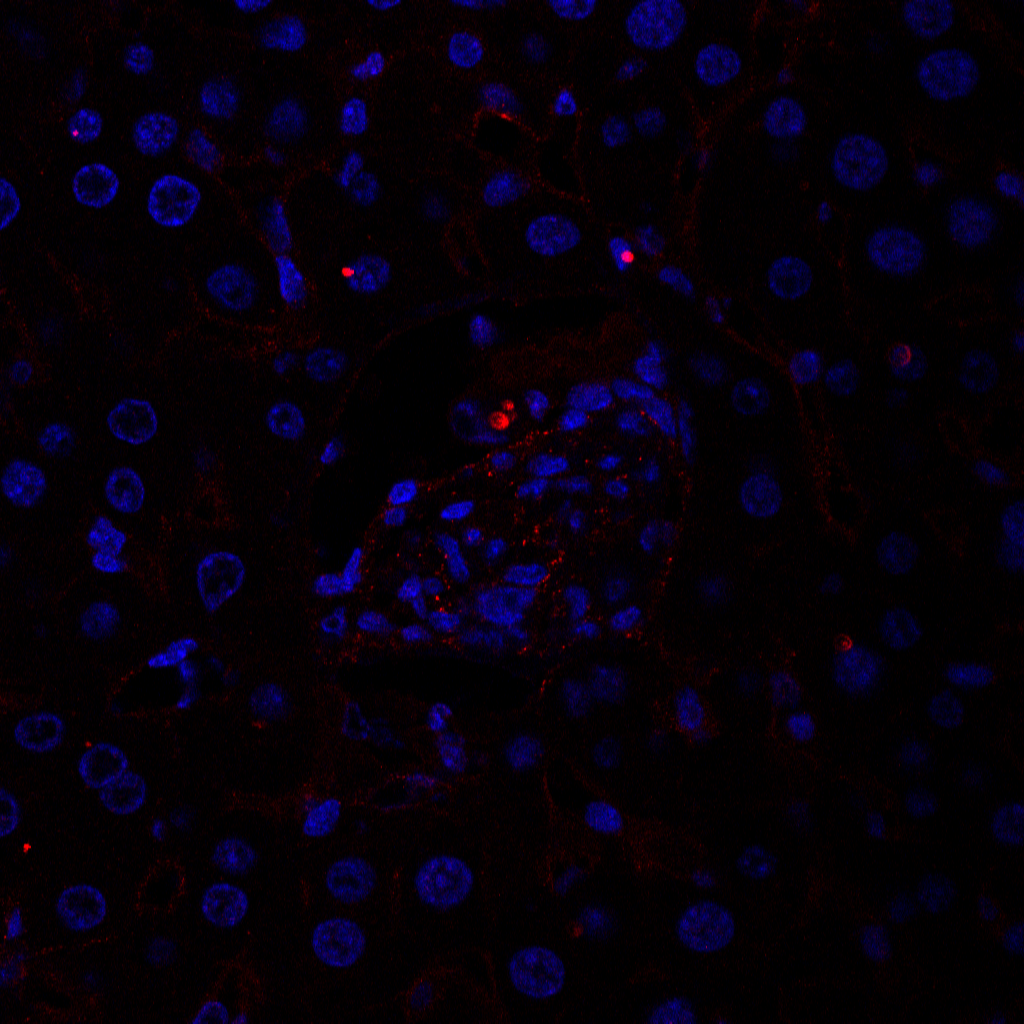

Supplement: Supplementary file 3 — Additional file 2. [file 12964_2023_1355_MOESM2_ESM.zip › raw data/Figure 5/Figure 5D/Figure 5D_SOR + WAY_DKK1.tif]

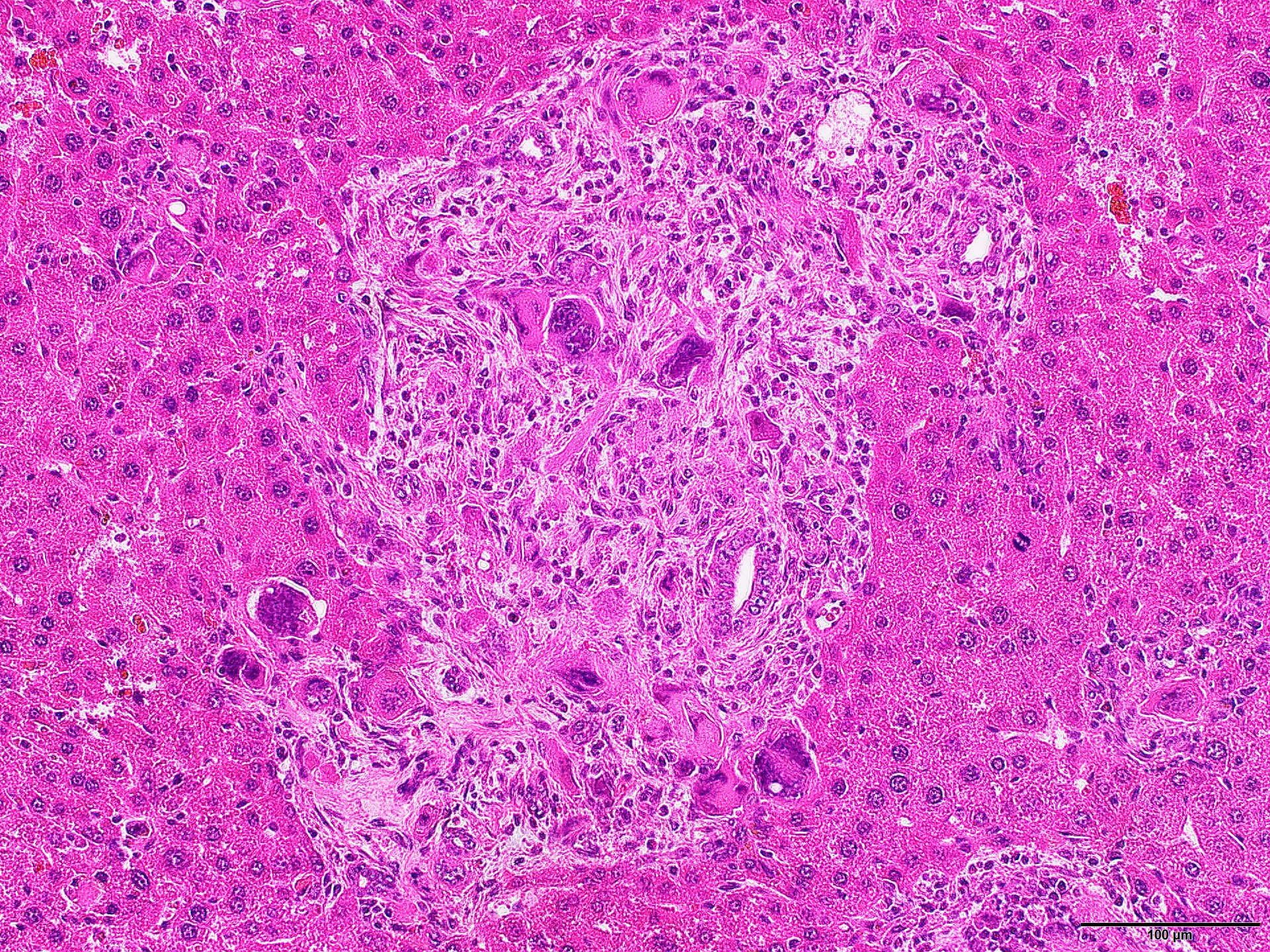

Supplement: Supplementary file 3 — Additional file 2. [file 12964_2023_1355_MOESM2_ESM.zip › raw data/Figure 5/Figure 5D/Figure 5D_SOR + WAY_H&E.jpg]

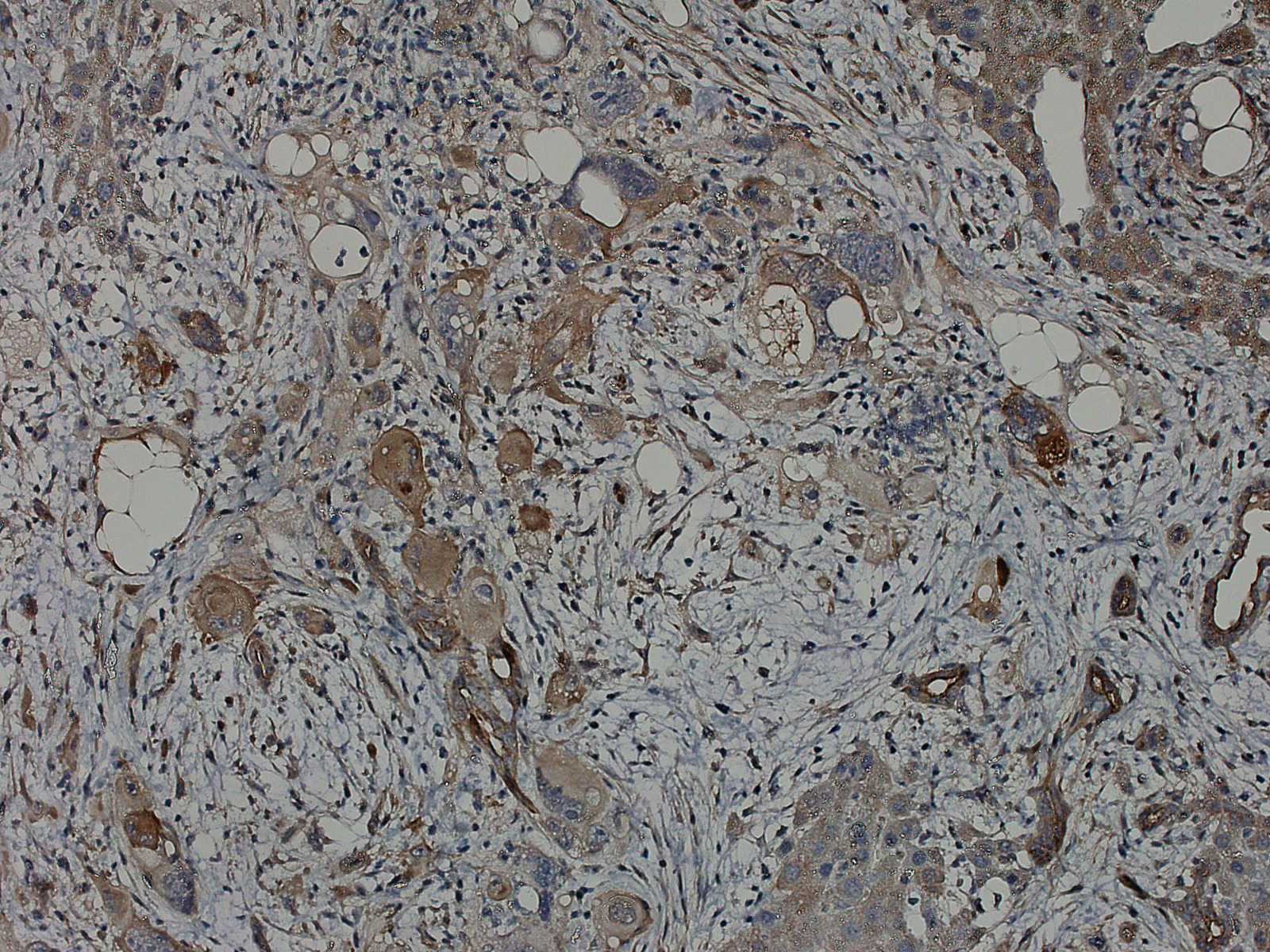

Supplement: Supplementary file 3 — Additional file 2. [file 12964_2023_1355_MOESM2_ESM.zip › raw data/Figure 5/Figure 5D/Figure 5D_SOR_GSK3╬▓ (Ser9).tif]

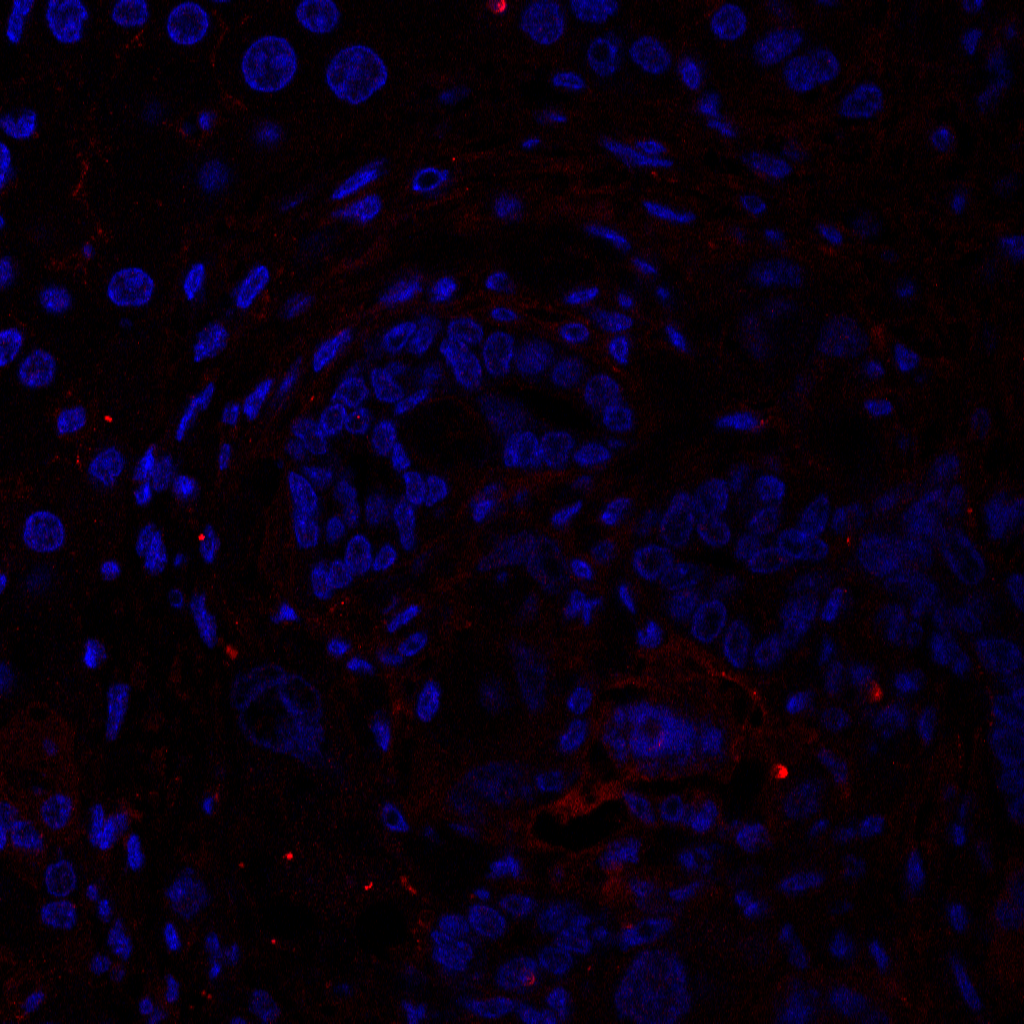

Supplement: Supplementary file 3 — Additional file 2. [file 12964_2023_1355_MOESM2_ESM.zip › raw data/Figure 5/Figure 5D/Figure 5D_SOR_DKK1.tif]

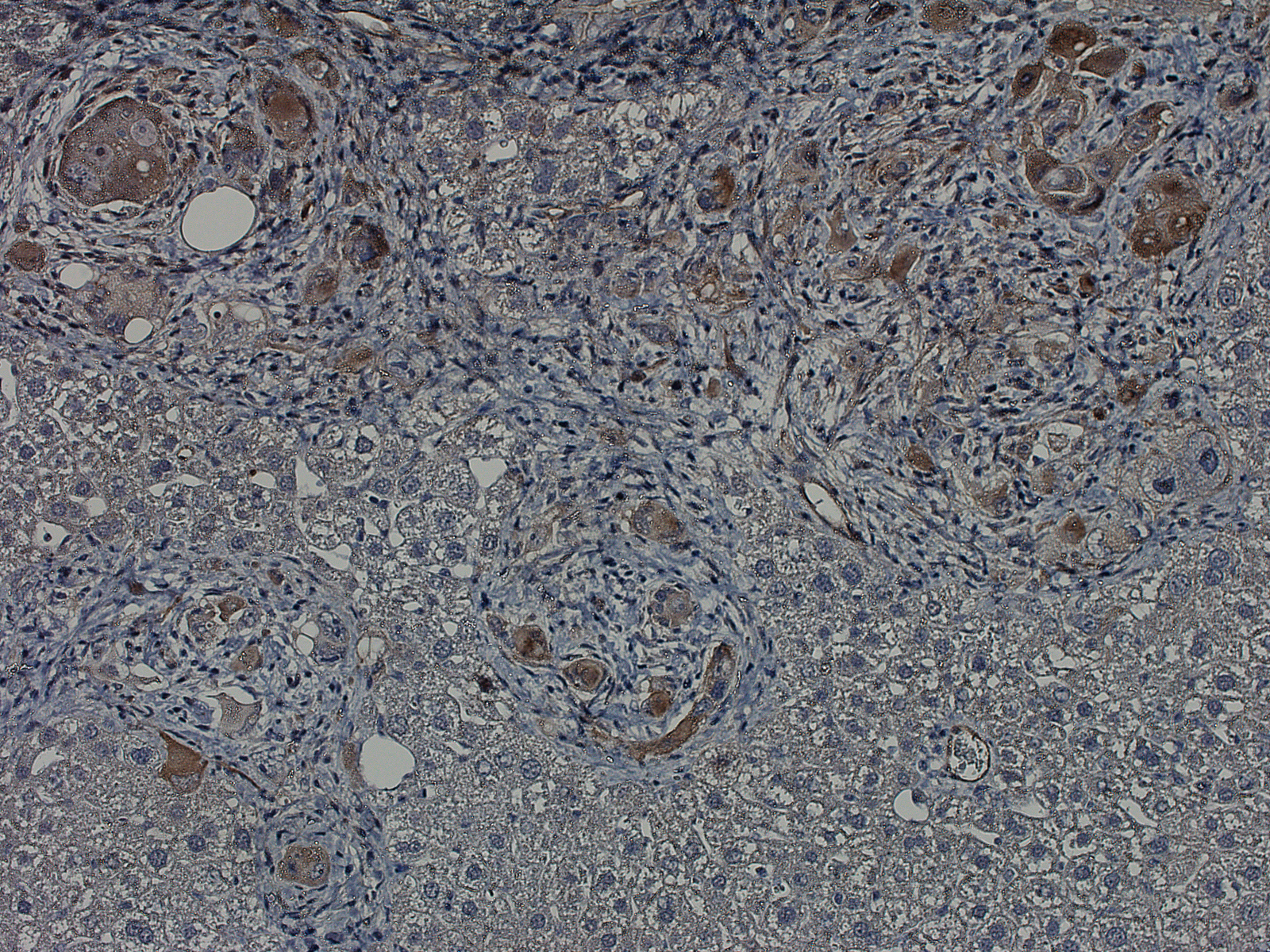

Supplement: Supplementary file 3 — Additional file 2. [file 12964_2023_1355_MOESM2_ESM.zip › raw data/Figure 5/Figure 5D/Figure 5D_WAY_GSK3╬▓ (Ser9).tif]

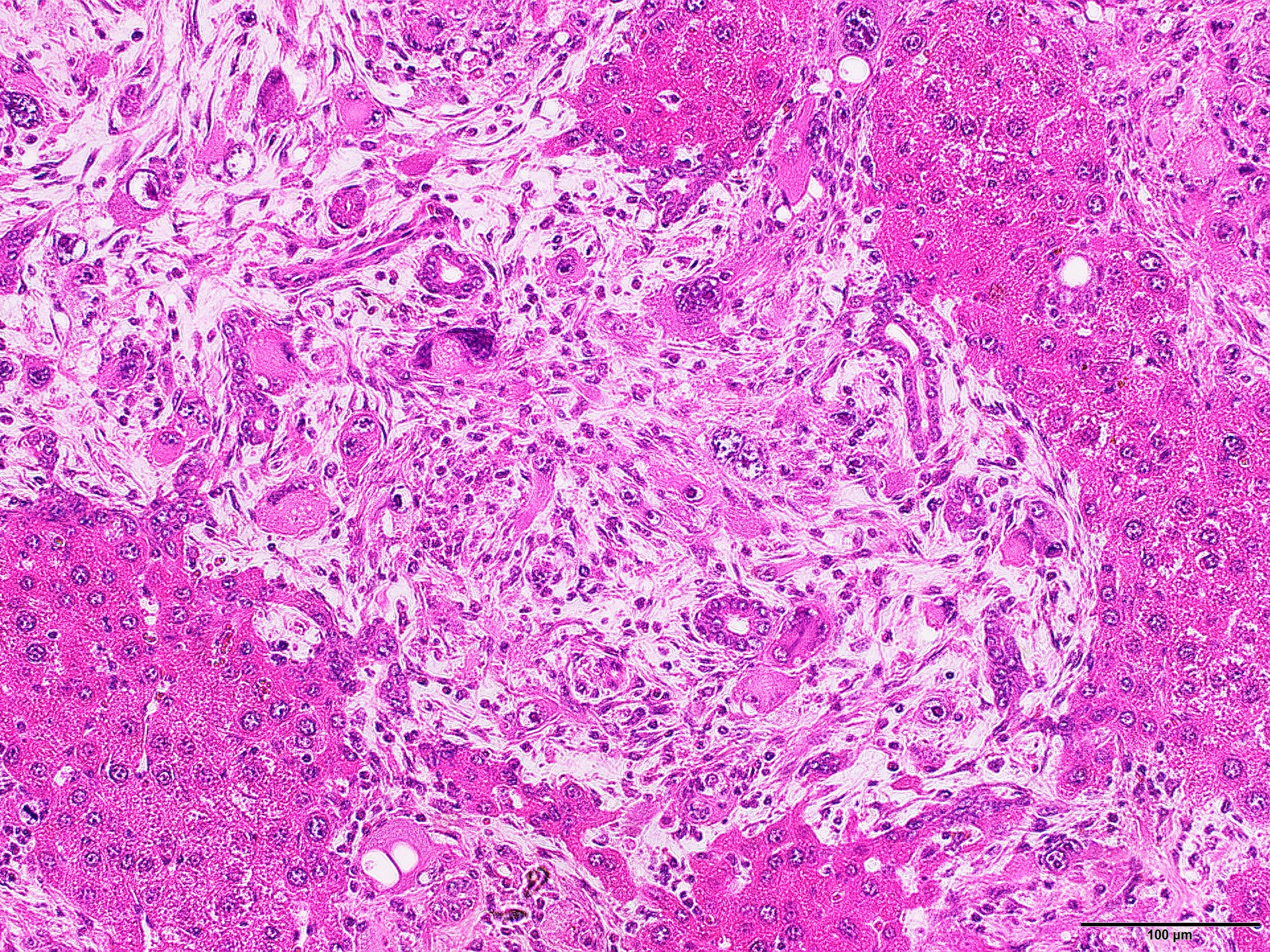

Supplement: Supplementary file 3 — Additional file 2. [file 12964_2023_1355_MOESM2_ESM.zip › raw data/Figure 5/Figure 5D/Figure 5D_SOR_H&E.jpg]

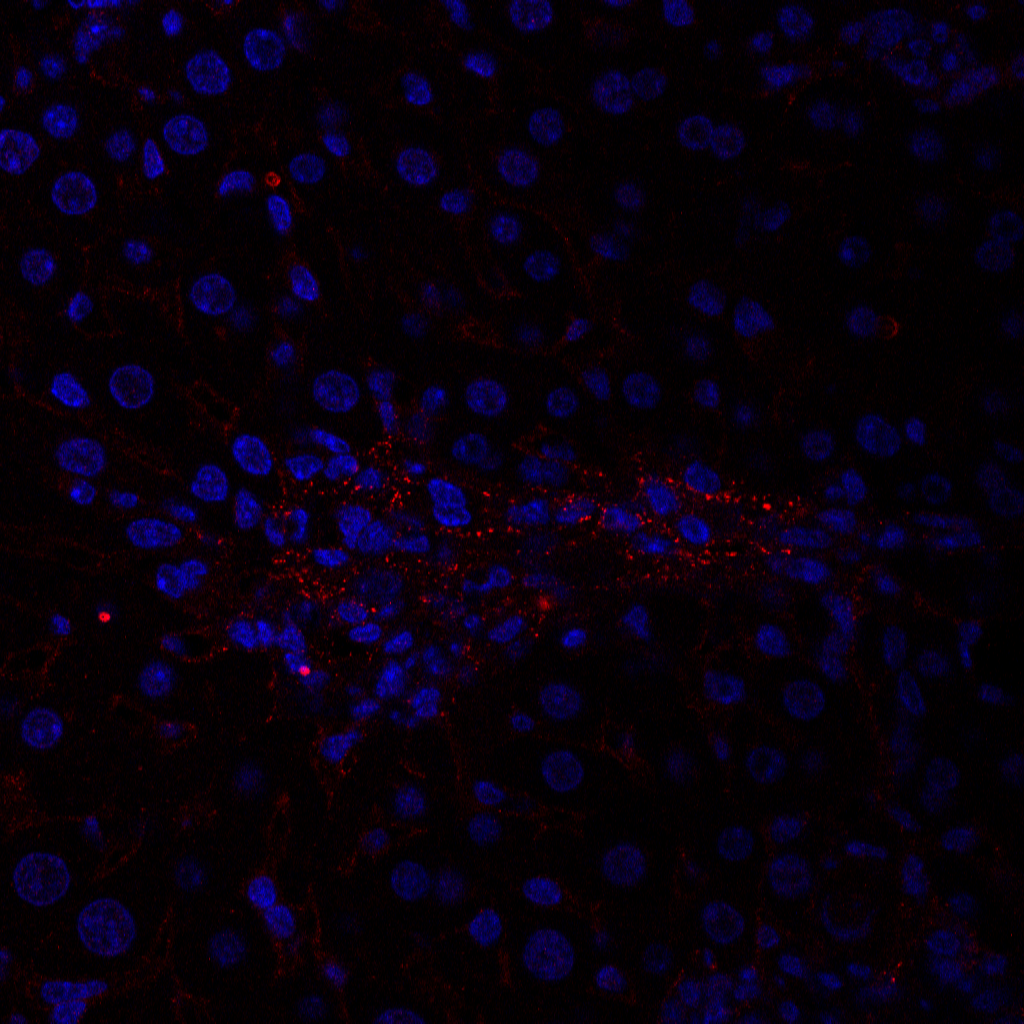

Supplement: Supplementary file 3 — Additional file 2. [file 12964_2023_1355_MOESM2_ESM.zip › raw data/Figure 5/Figure 5D/Figure 5D_WAY_DKK1.tif]

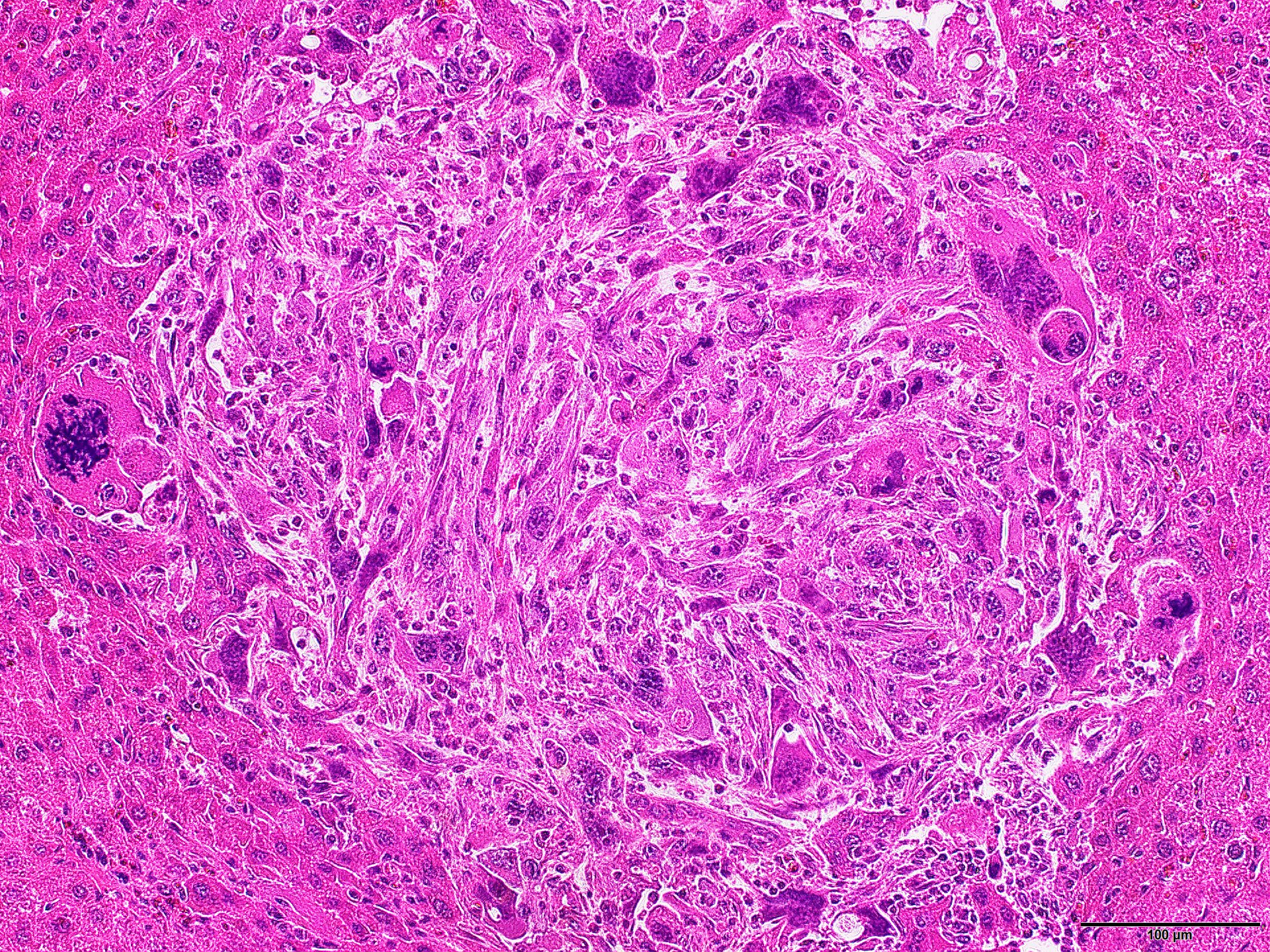

Supplement: Supplementary file 3 — Additional file 2. [file 12964_2023_1355_MOESM2_ESM.zip › raw data/Figure 5/Figure 5D/Figure 5D_WAY_H&E.jpg]

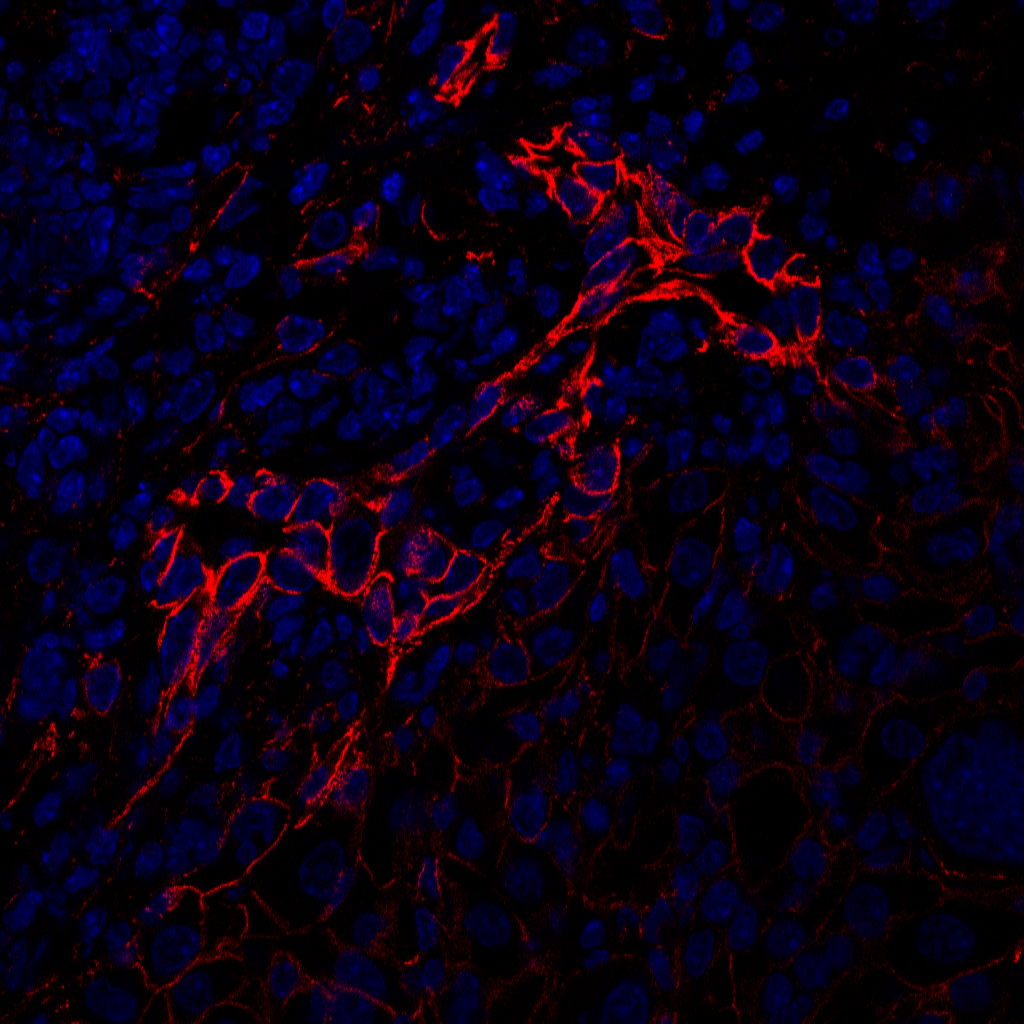

Supplement: Supplementary file 3 — Additional file 2. [file 12964_2023_1355_MOESM2_ESM.zip › raw data/Figure 5/Figure 5D/Figure 5D_Control_Active ╬▓-catenin.tif]

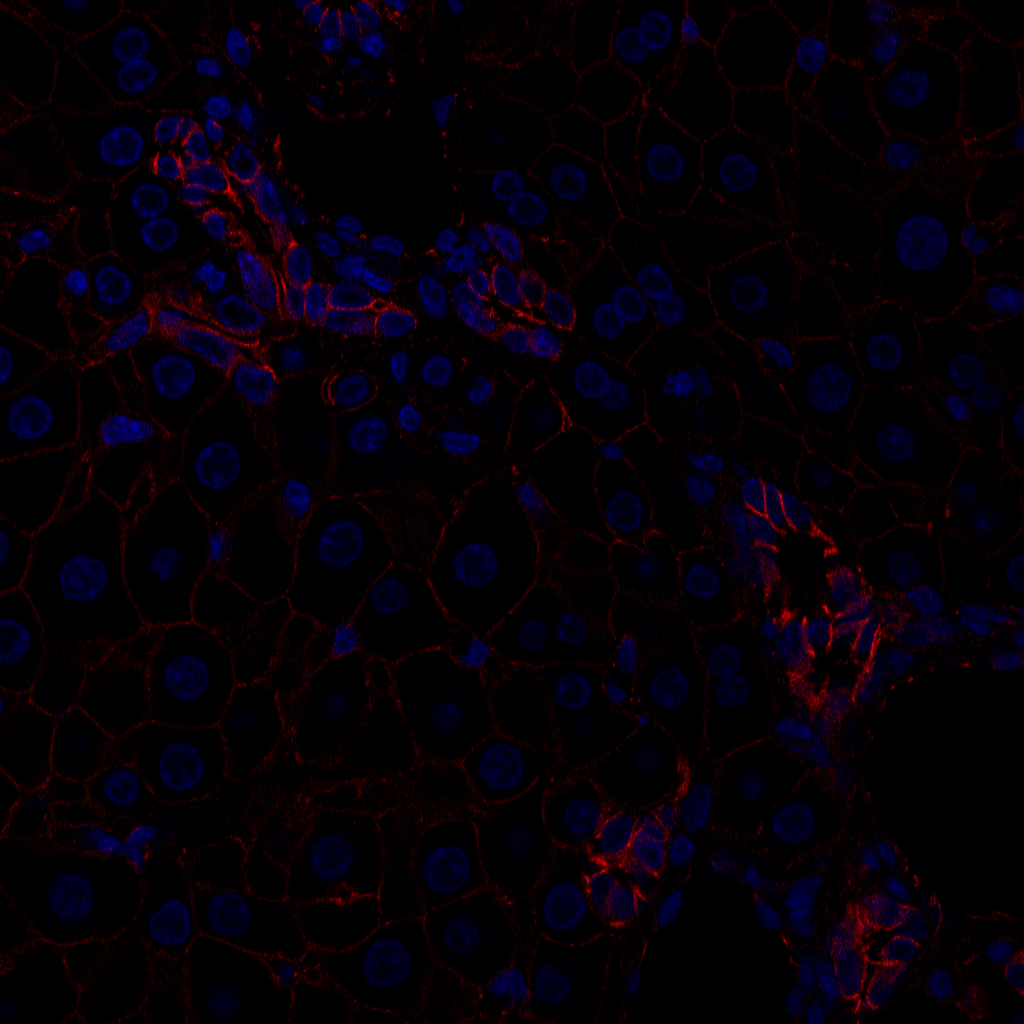

Supplement: Supplementary file 3 — Additional file 2. [file 12964_2023_1355_MOESM2_ESM.zip › raw data/Figure 5/Figure 5D/Figure 5D_SOR_Active ╬▓-catenin.tif]

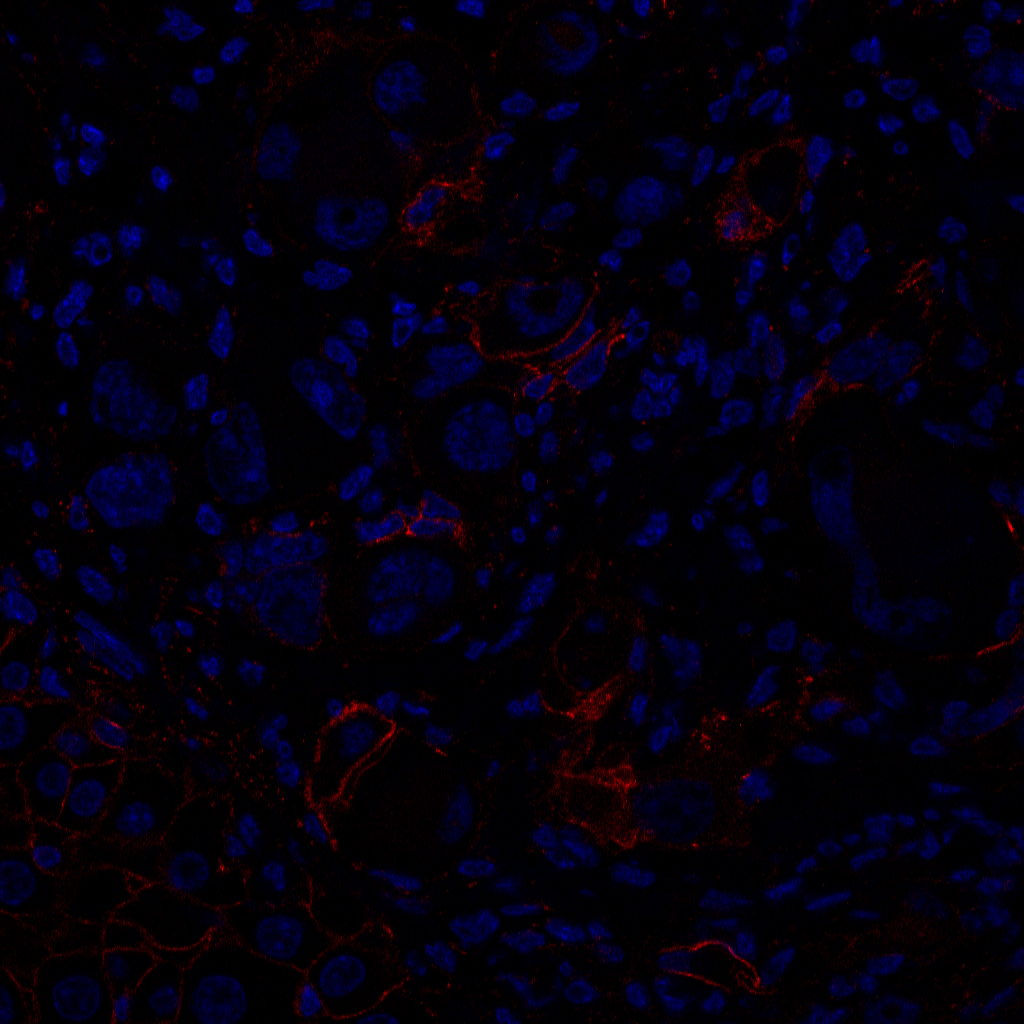

Supplement: Supplementary file 3 — Additional file 2. [file 12964_2023_1355_MOESM2_ESM.zip › raw data/Figure 5/Figure 5D/Figure 5D_WAY_Active ╬▓-catenin.tif]

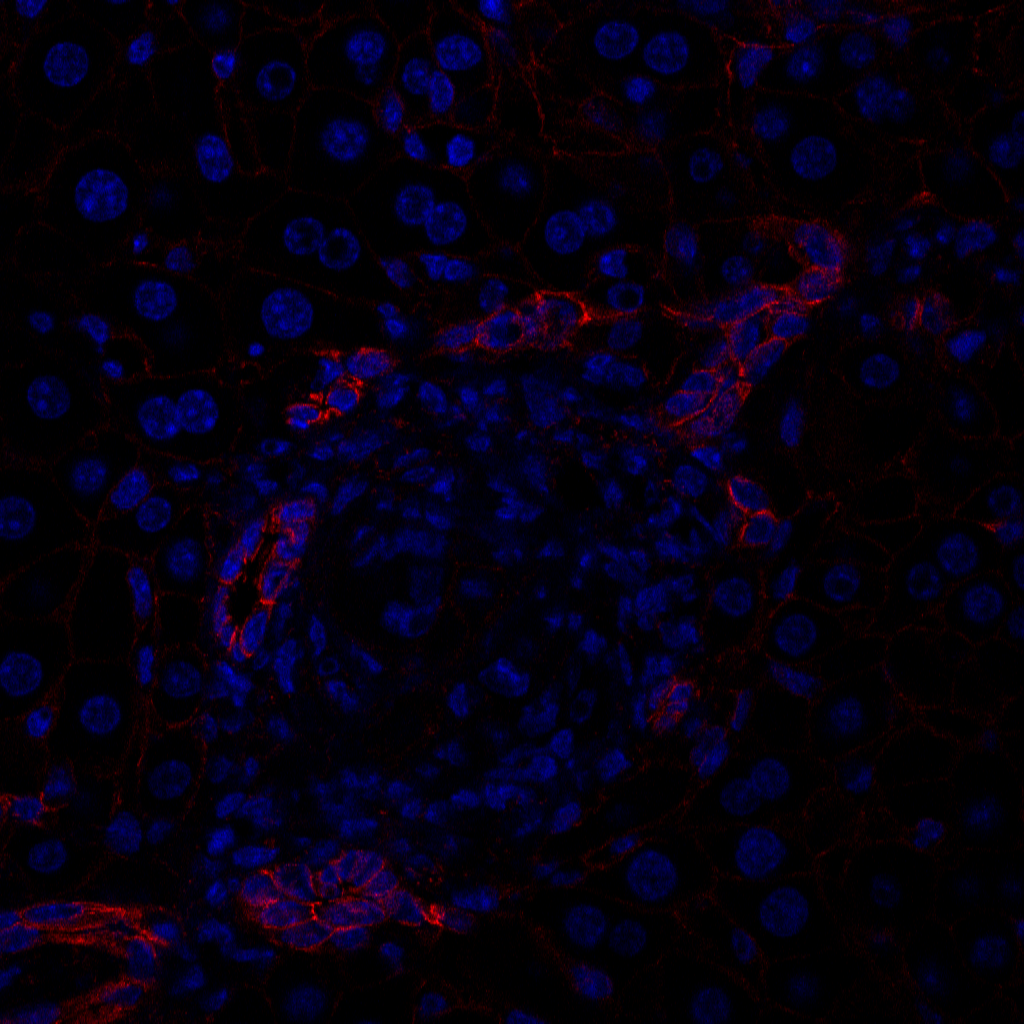

Supplement: Supplementary file 3 — Additional file 2. [file 12964_2023_1355_MOESM2_ESM.zip › raw data/Figure 5/Figure 5D/Figure 5D_SOR + WAY_Active ╬▓-catenin.tif]

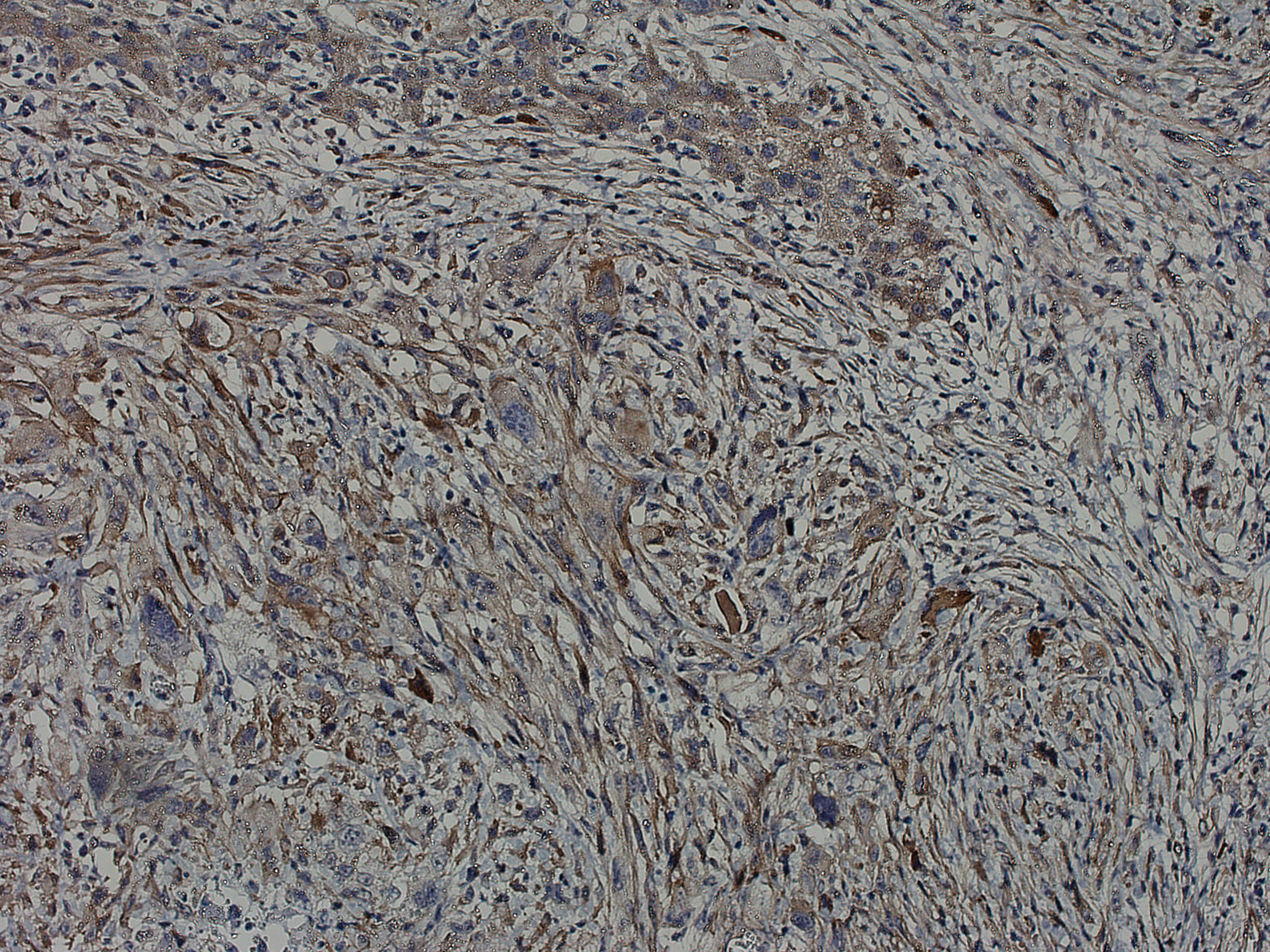

Supplement: Supplementary file 3 — Additional file 2. [file 12964_2023_1355_MOESM2_ESM.zip › raw data/Figure 5/Figure 5D/Figure 5D_Control_p-GSK3╬▓ (Ser9).tif]

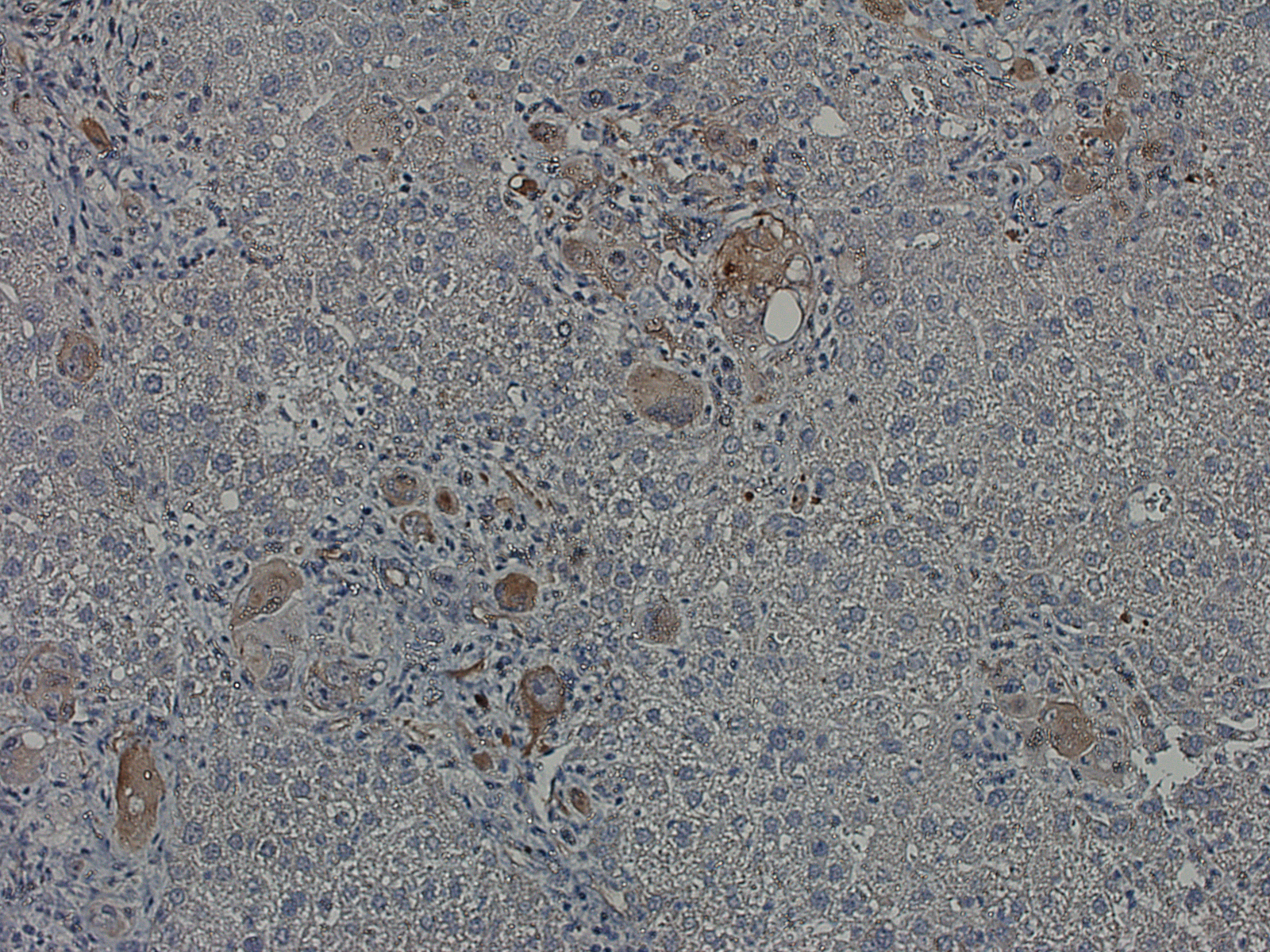

Supplement: Supplementary file 3 — Additional file 2. [file 12964_2023_1355_MOESM2_ESM.zip › raw data/Figure 5/Figure 5D/Figure 5D_SOR + WAY_GSK3╬▓ (Ser9).tif]

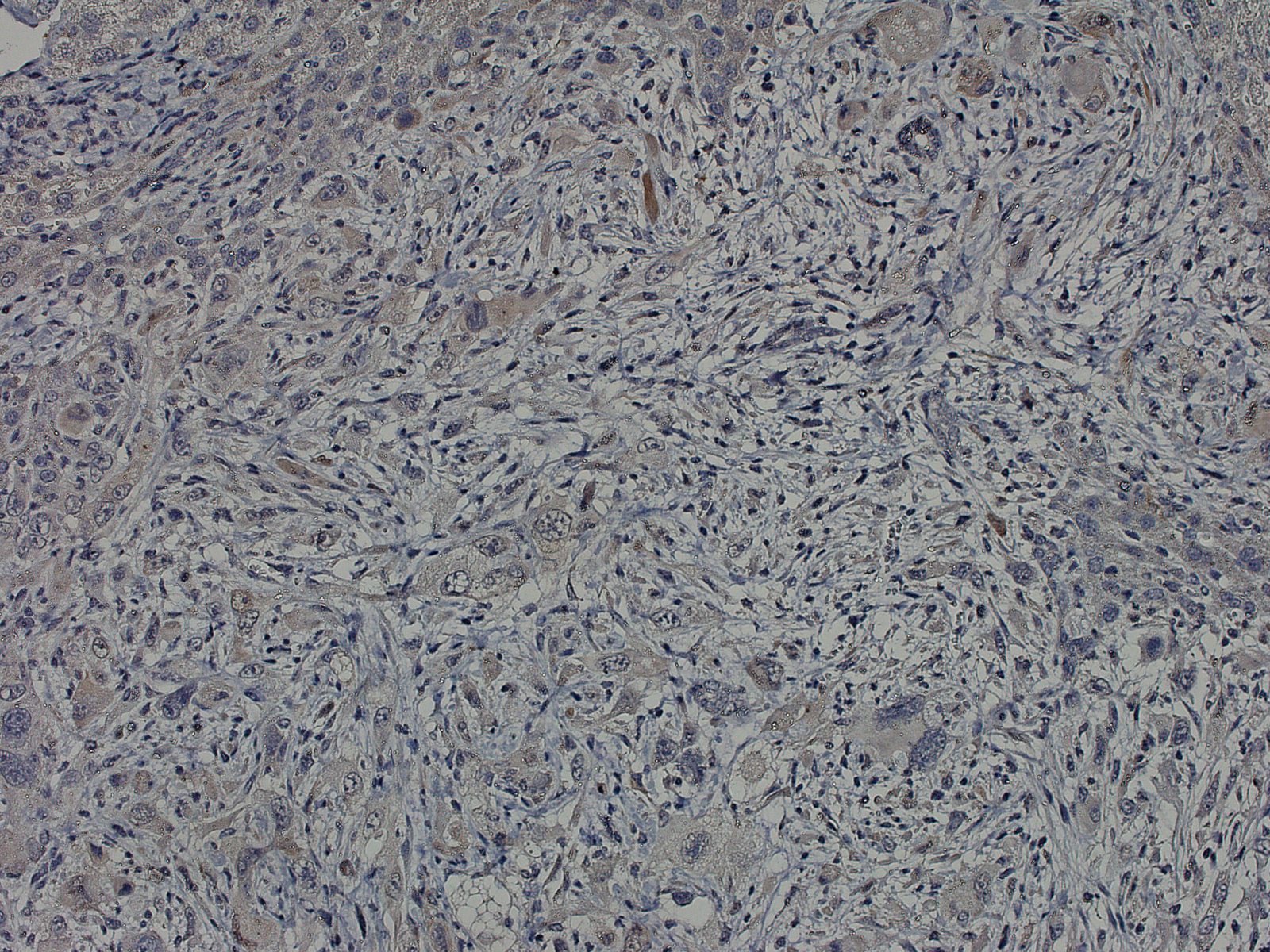

Supplement: Supplementary file 3 — Additional file 2. [file 12964_2023_1355_MOESM2_ESM.zip › raw data/Figure 5/Figure 5D/Figure 5D_Control_GSK3╬▓ (Tyr216).tif]

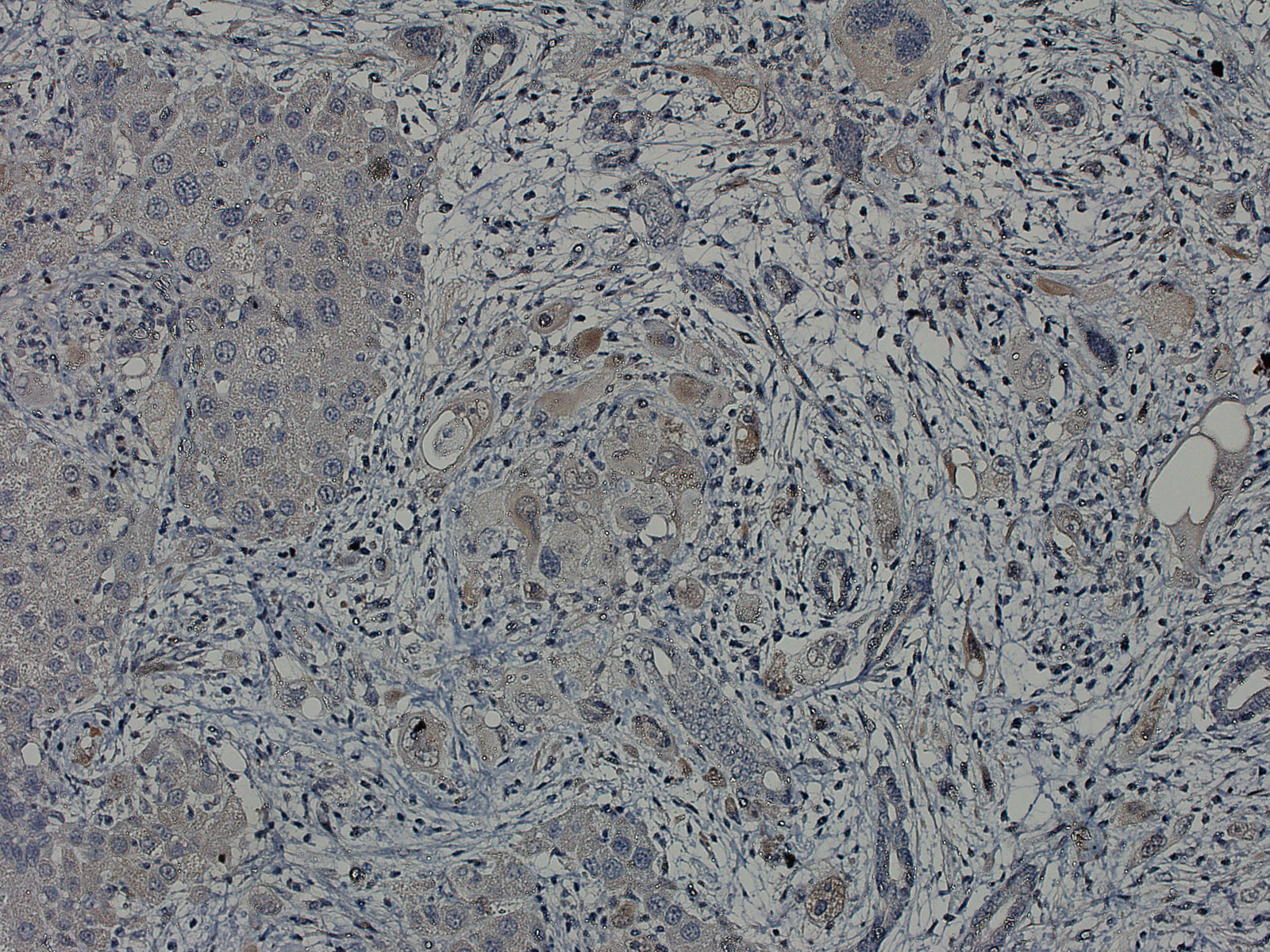

Supplement: Supplementary file 3 — Additional file 2. [file 12964_2023_1355_MOESM2_ESM.zip › raw data/Figure 5/Figure 5D/Figure 5D_SOR_GSK3╬▓ (Tyr216).tif]

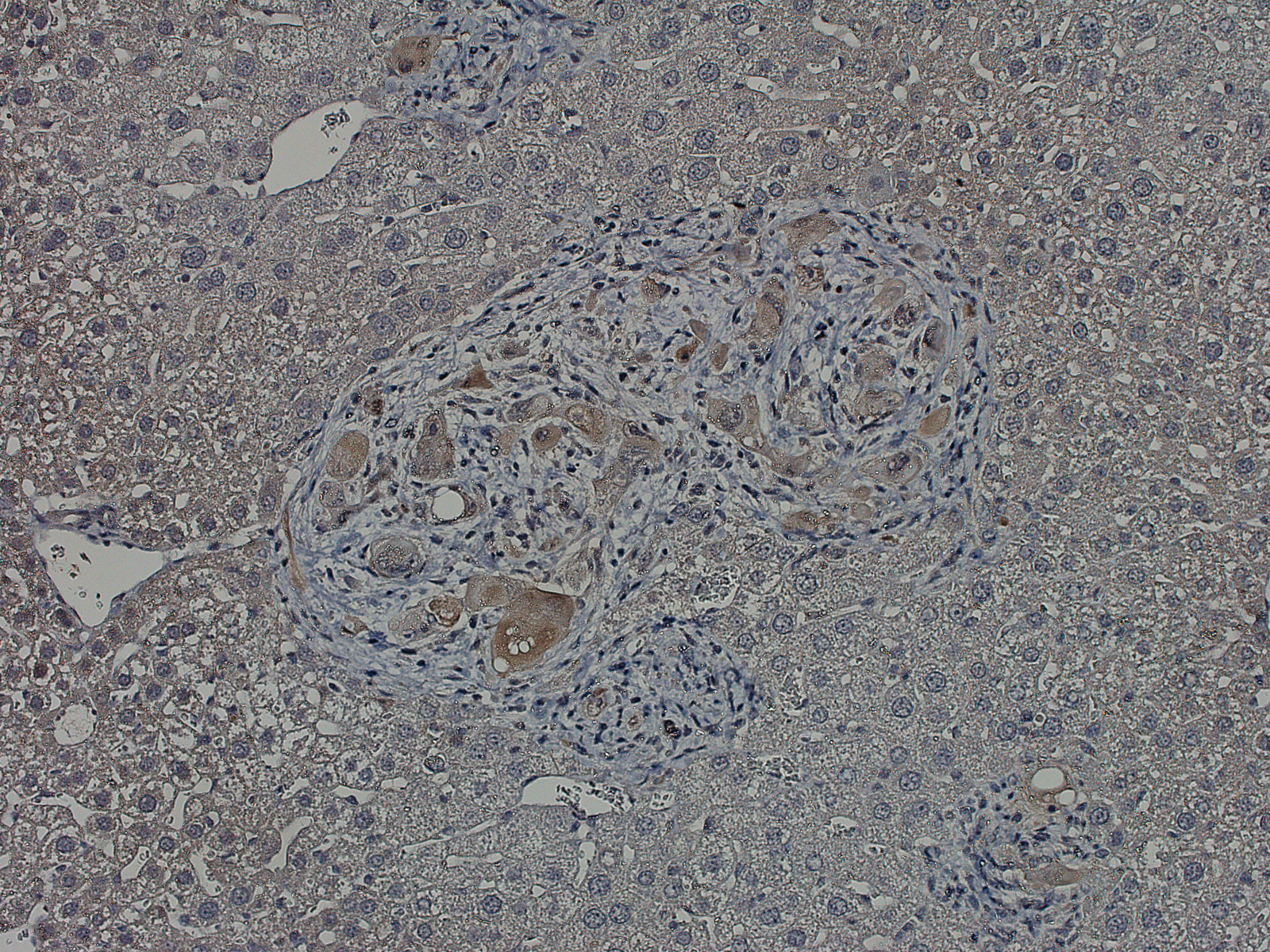

Supplement: Supplementary file 3 — Additional file 2. [file 12964_2023_1355_MOESM2_ESM.zip › raw data/Figure 5/Figure 5D/Figure 5D_WAY_GSK3╬▓ (Tyr216).tif]

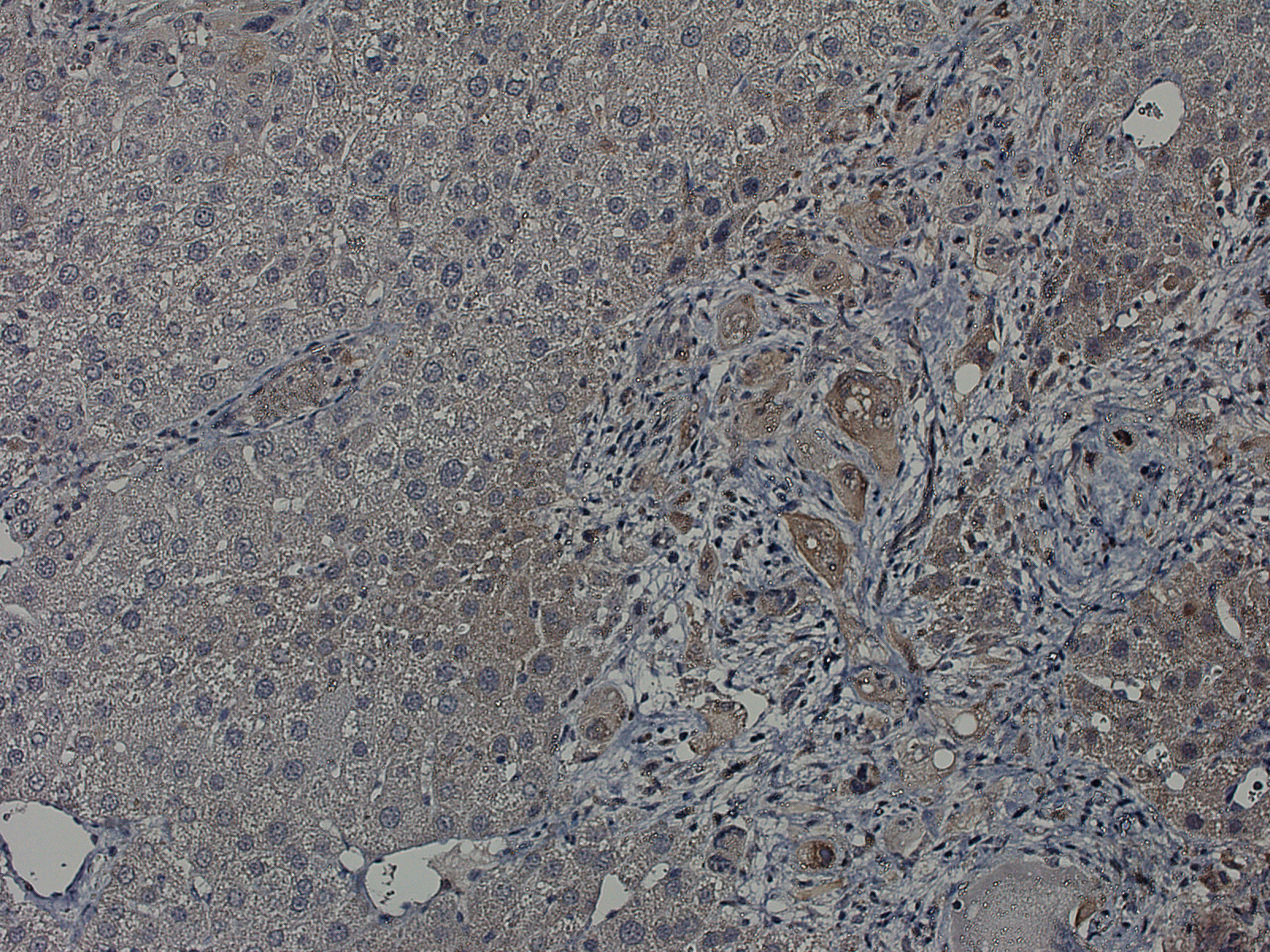

Supplement: Supplementary file 3 — Additional file 2. [file 12964_2023_1355_MOESM2_ESM.zip › raw data/Figure 5/Figure 5D/Figure 5D_SOR + WAY_GSK3╬▓ (Tyr216).tif]

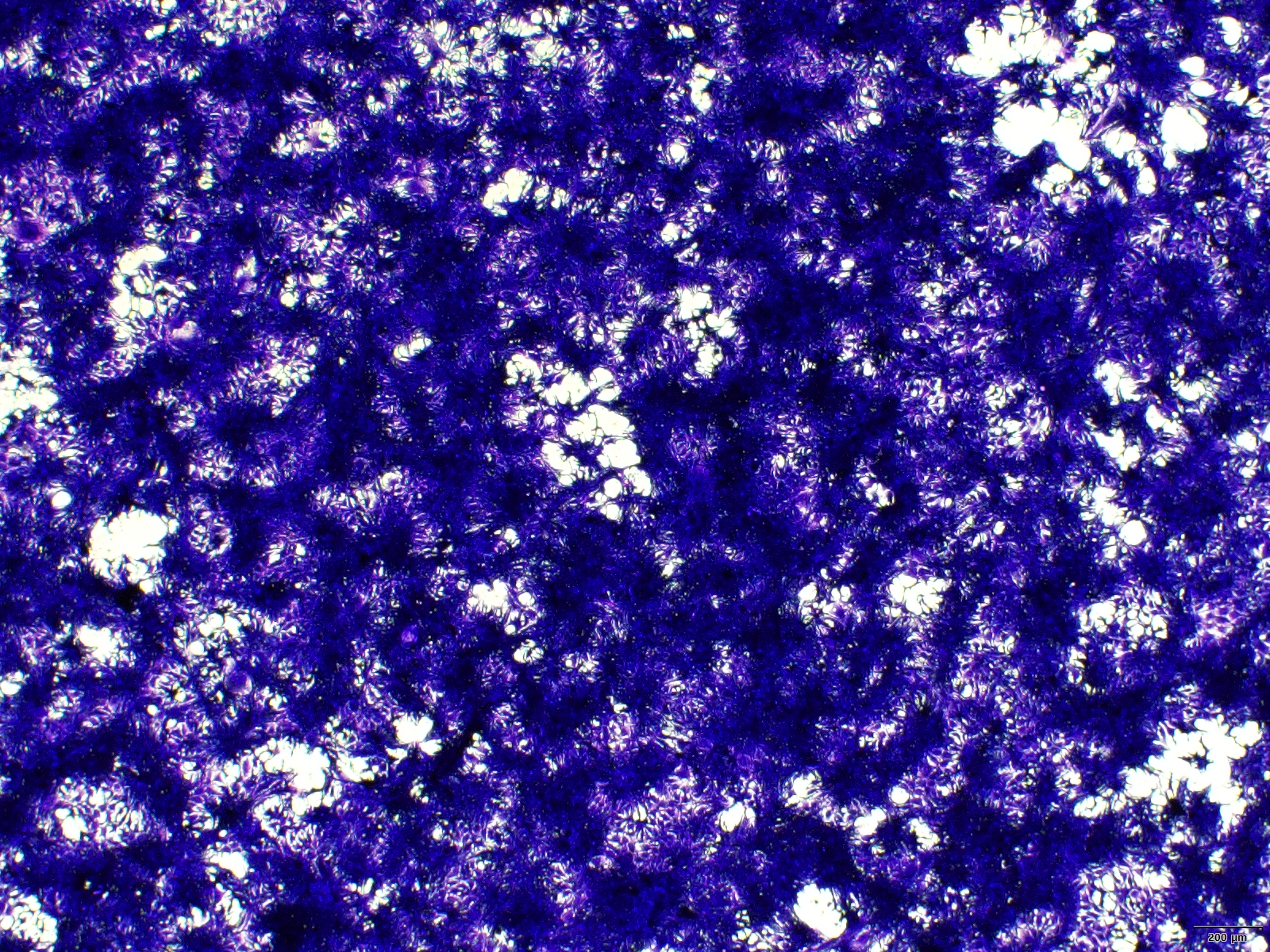

Supplement: Supplementary file 3 — Additional file 2. [file 12964_2023_1355_MOESM2_ESM.zip › raw data/Figure 4/Figure 4E/Hep3B/SOR 0 ╬╝M+LY294002 0 ╬╝M.jpg]

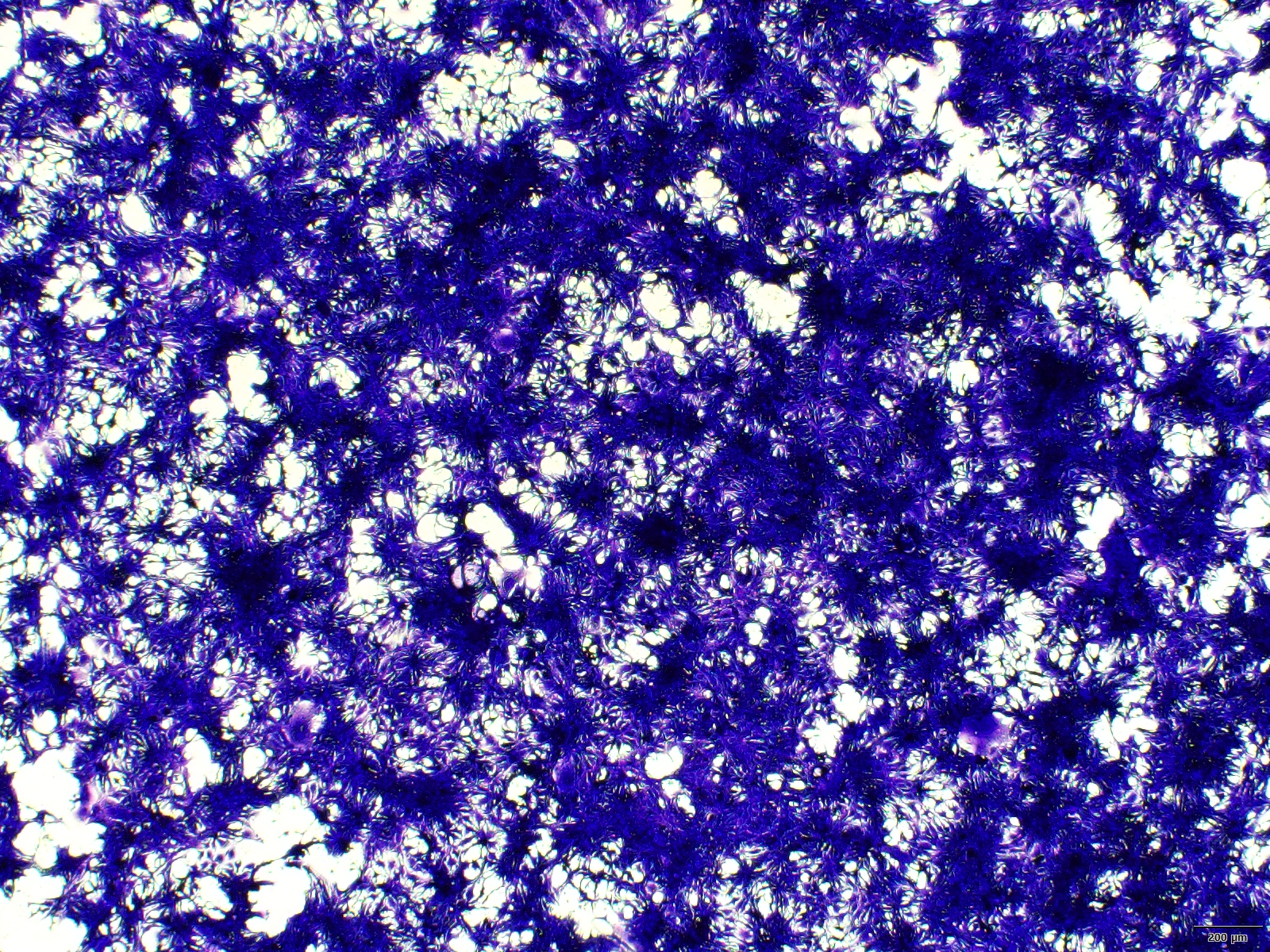

Supplement: Supplementary file 3 — Additional file 2. [file 12964_2023_1355_MOESM2_ESM.zip › raw data/Figure 4/Figure 4E/Hep3B/SOR 1 ╬╝M+LY294002 0 ╬╝M.jpg]

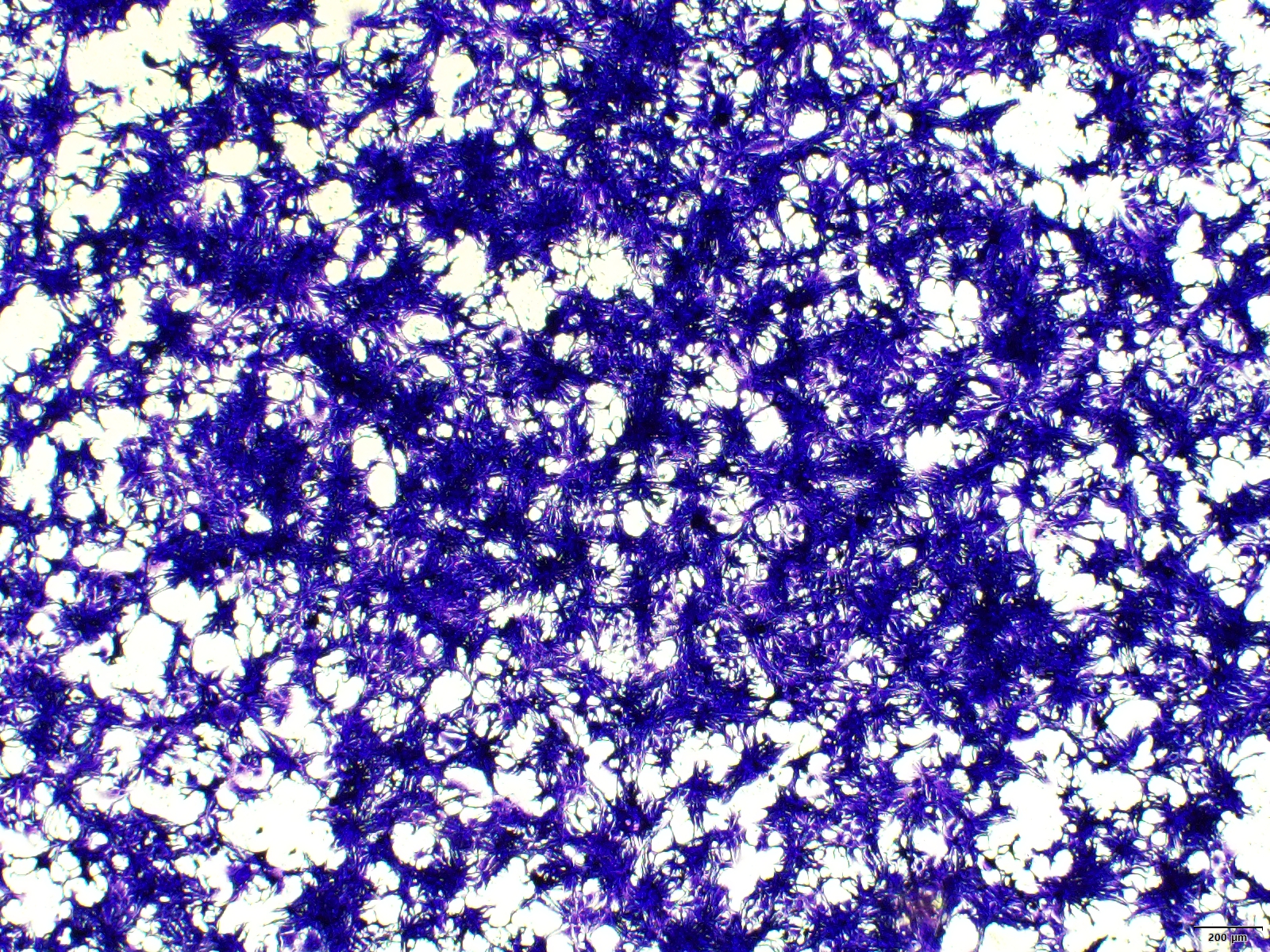

Supplement: Supplementary file 3 — Additional file 2. [file 12964_2023_1355_MOESM2_ESM.zip › raw data/Figure 4/Figure 4E/Hep3B/SOR 2╬╝M+LY294002 0 ╬╝M.jpg]

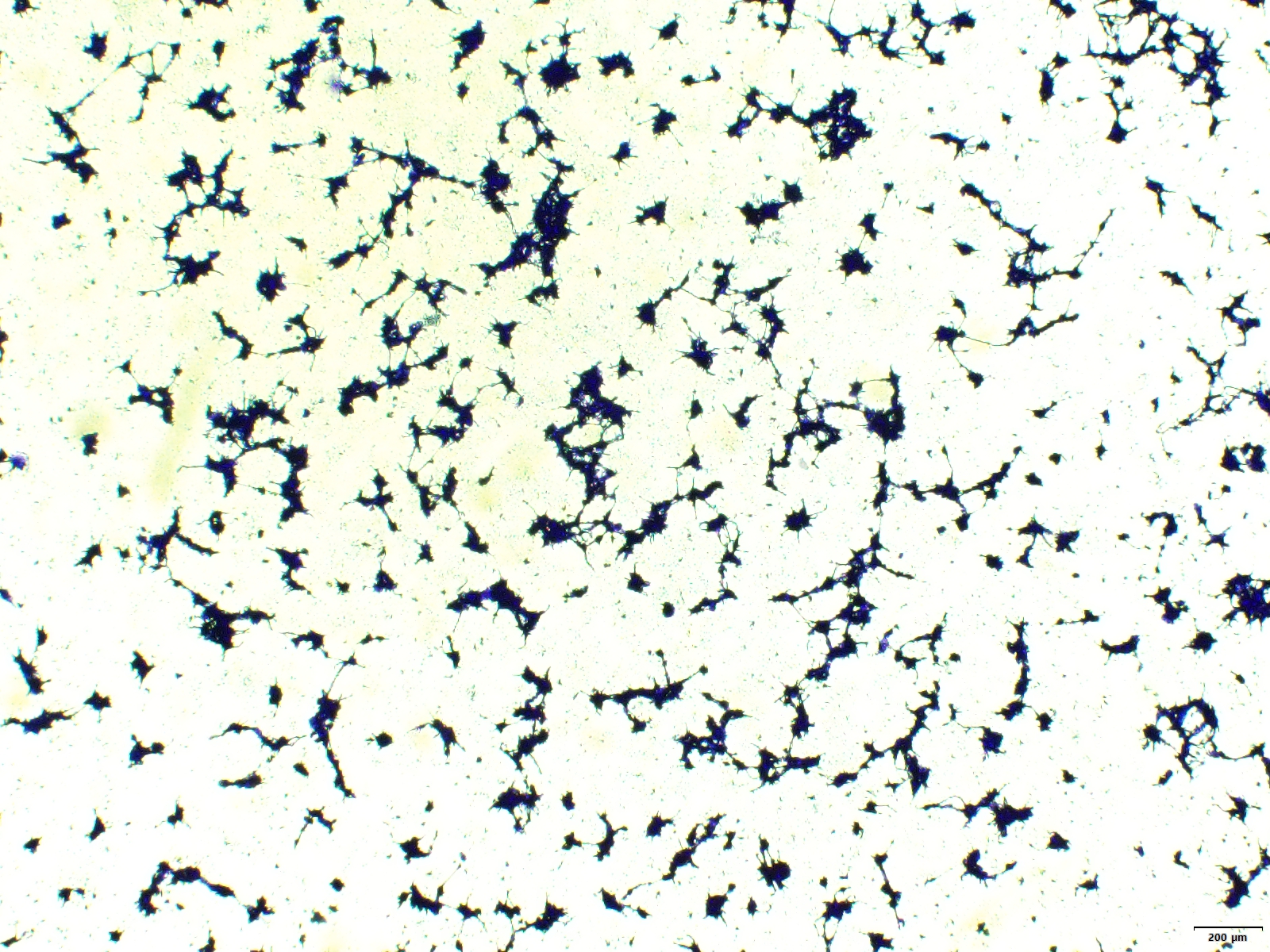

Supplement: Supplementary file 3 — Additional file 2. [file 12964_2023_1355_MOESM2_ESM.zip › raw data/Figure 4/Figure 4E/Hep3B/SOR 4 ╬╝M+LY294002 0 ╬╝M.jpg]

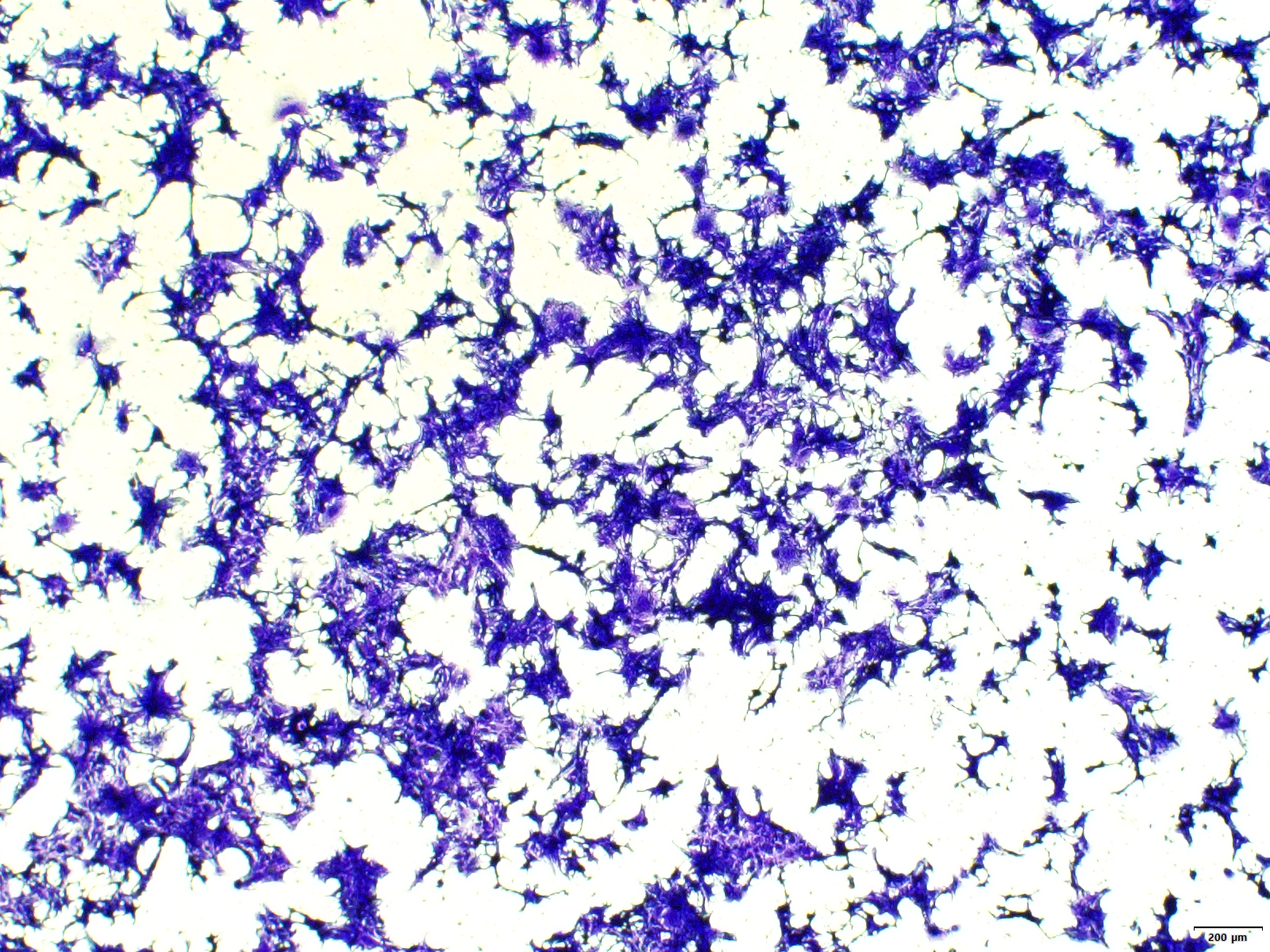

Supplement: Supplementary file 3 — Additional file 2. [file 12964_2023_1355_MOESM2_ESM.zip › raw data/Figure 4/Figure 4E/Hep3B/SOR 0 ╬╝M+LY294002 25 ╬╝M.jpg]
