# Supplementary material for: Inhibition of Dickkopf-1 enhances the anti-tumor efficacy of sorafenib via inhibition of the PI3K/Akt and Wnt/β-catenin pathways in hepatocellular carcinoma
Source: Cell Commun Signal. 2023 Nov 27;21:339. doi: 10.1186/s12964-023-01355-2 (PMC10680194; doi:10.1186/s12964-023-01355-2)

Figure 3.

Figure 3B

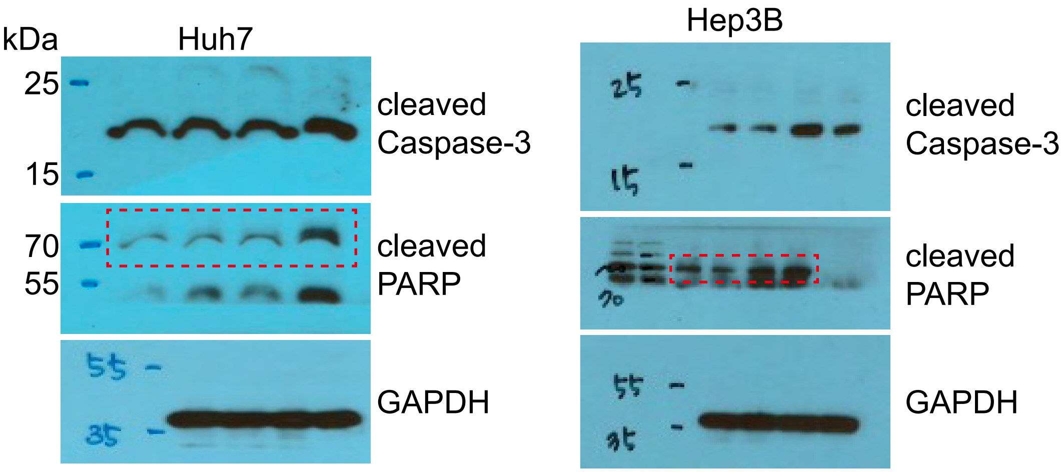

Figure 4.

Figure 4B

Huh7

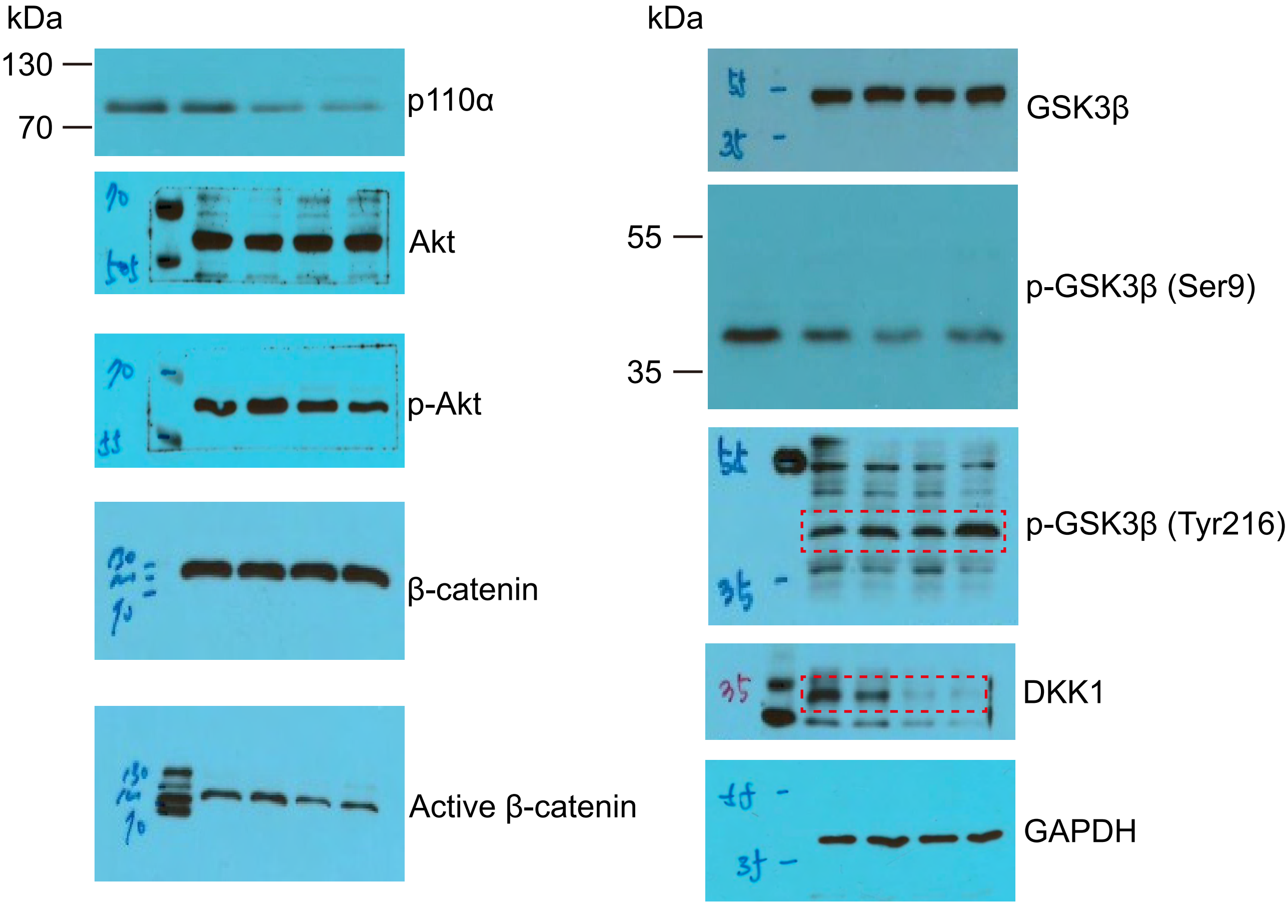

Figure 4.

Figure 4B

Hep3B

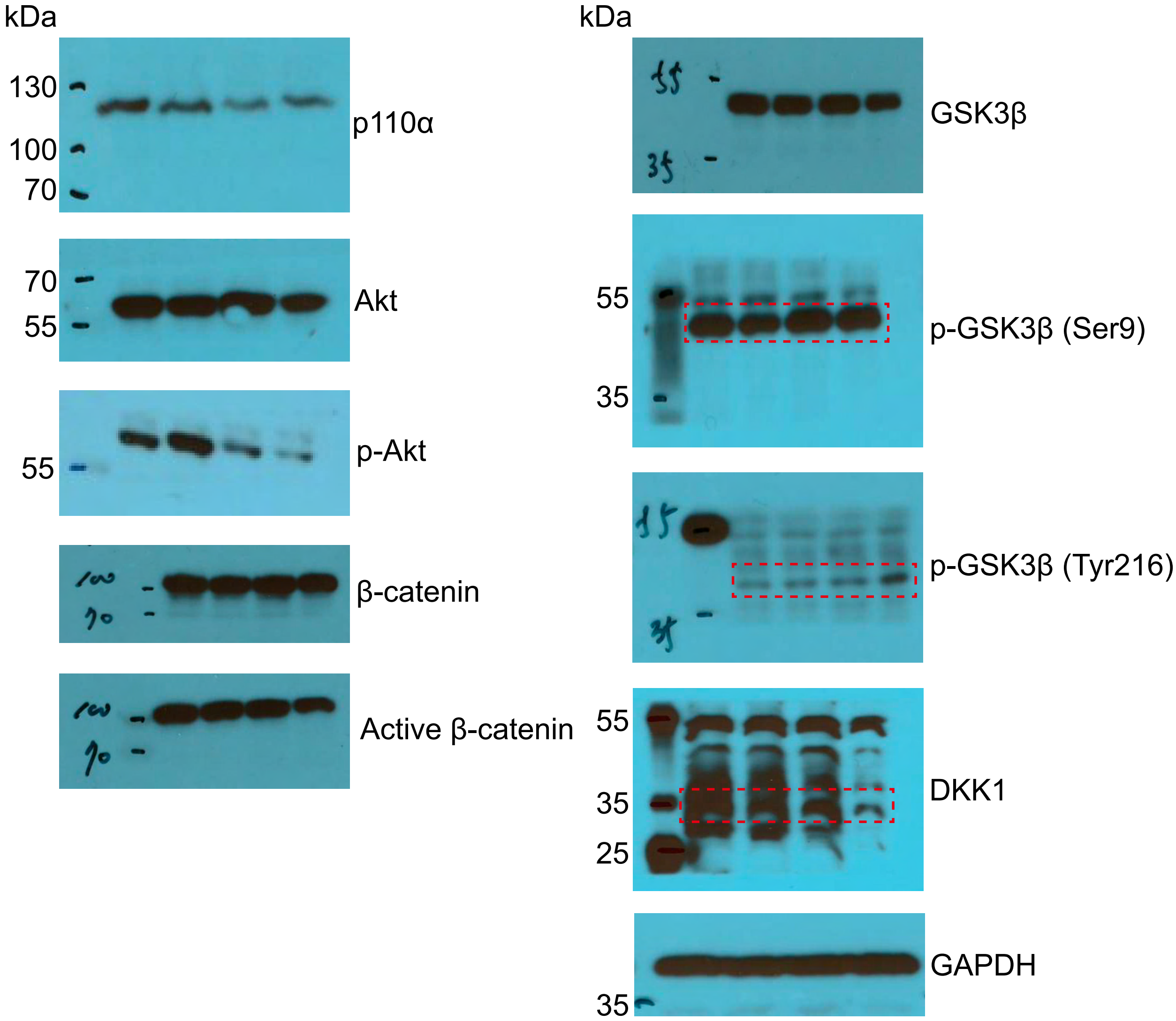

Figure 4.

Figure 4D

Huh7

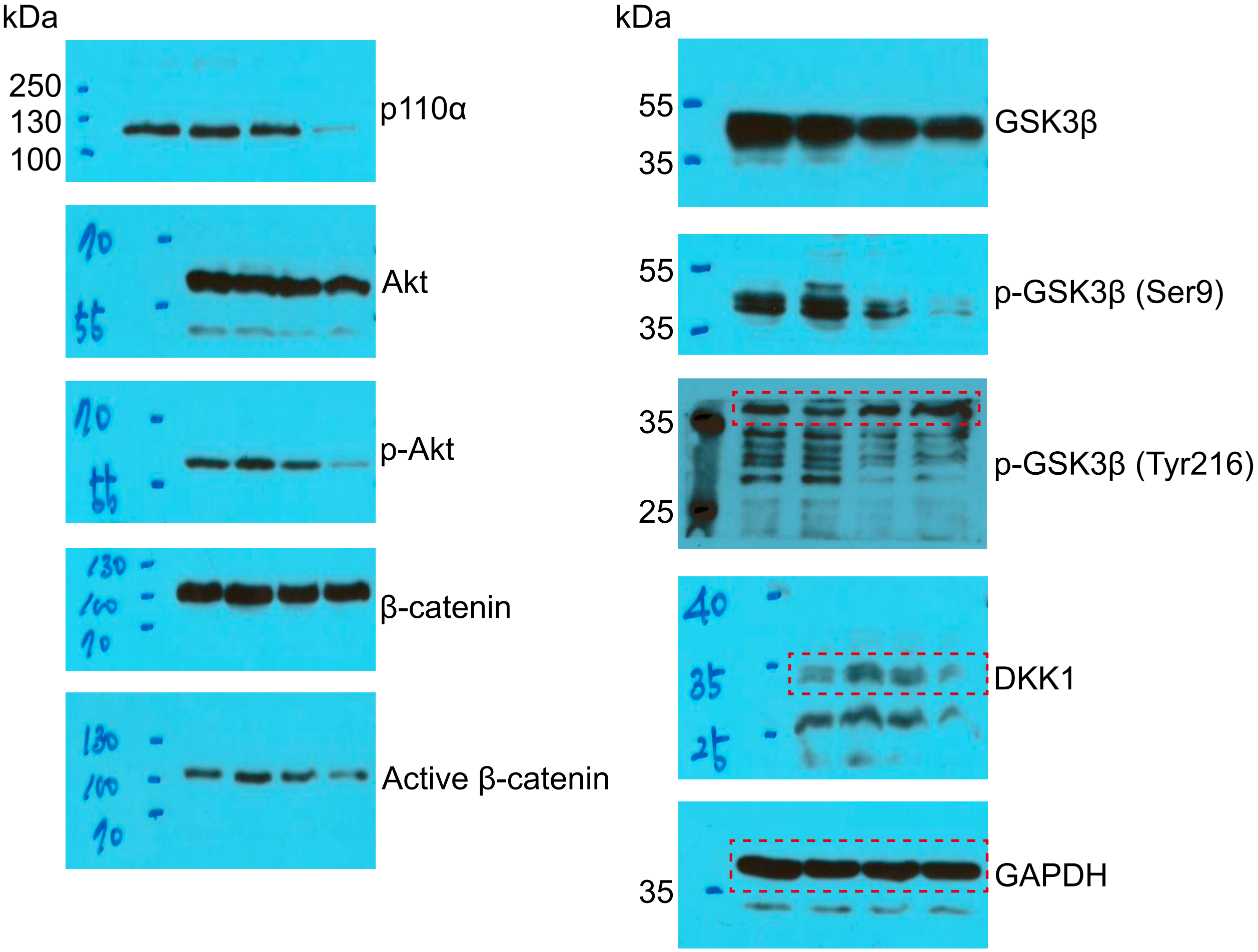

Figure 4.

Figure 4D

Hep3B

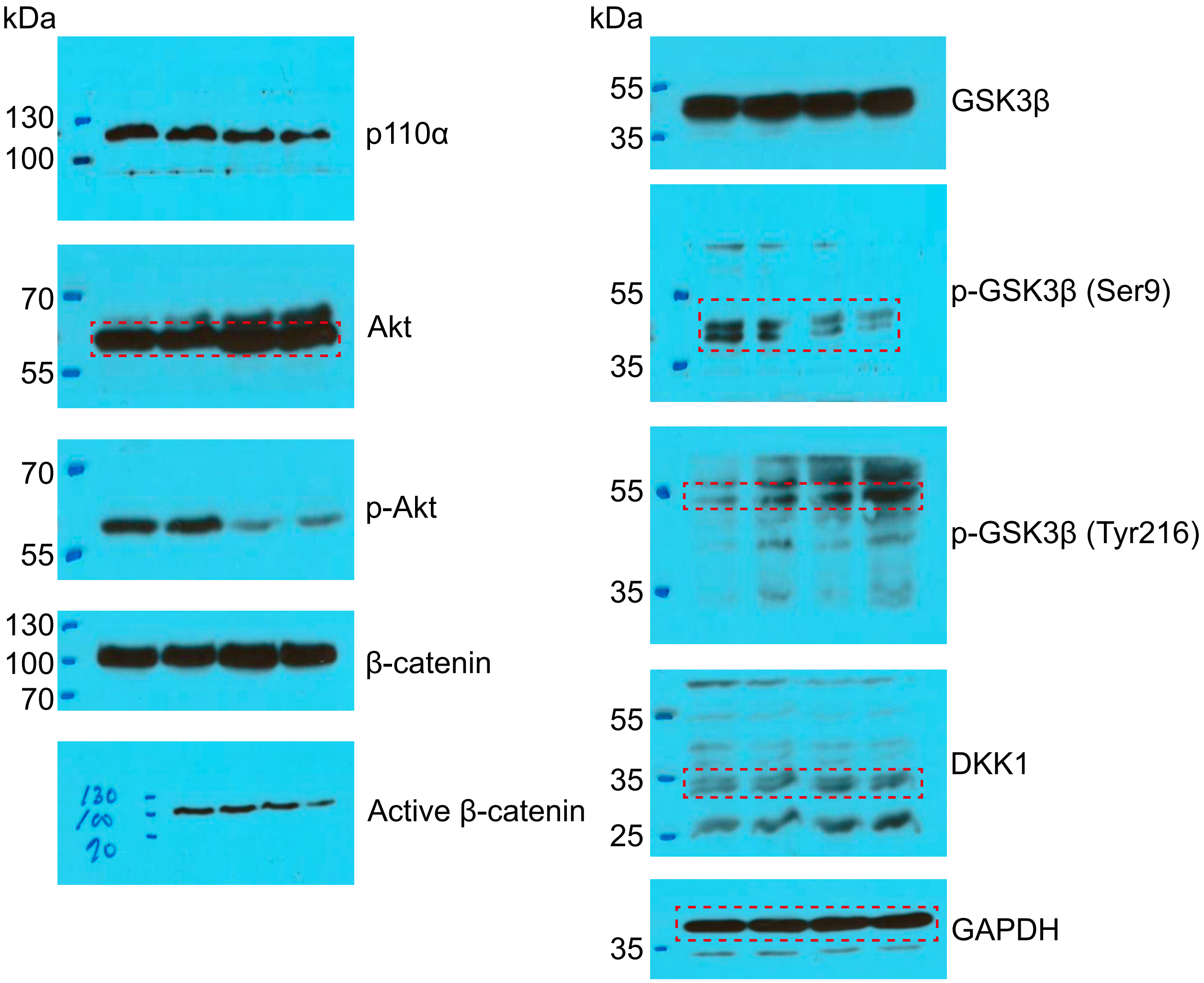

Figure 5.

Figure 5C

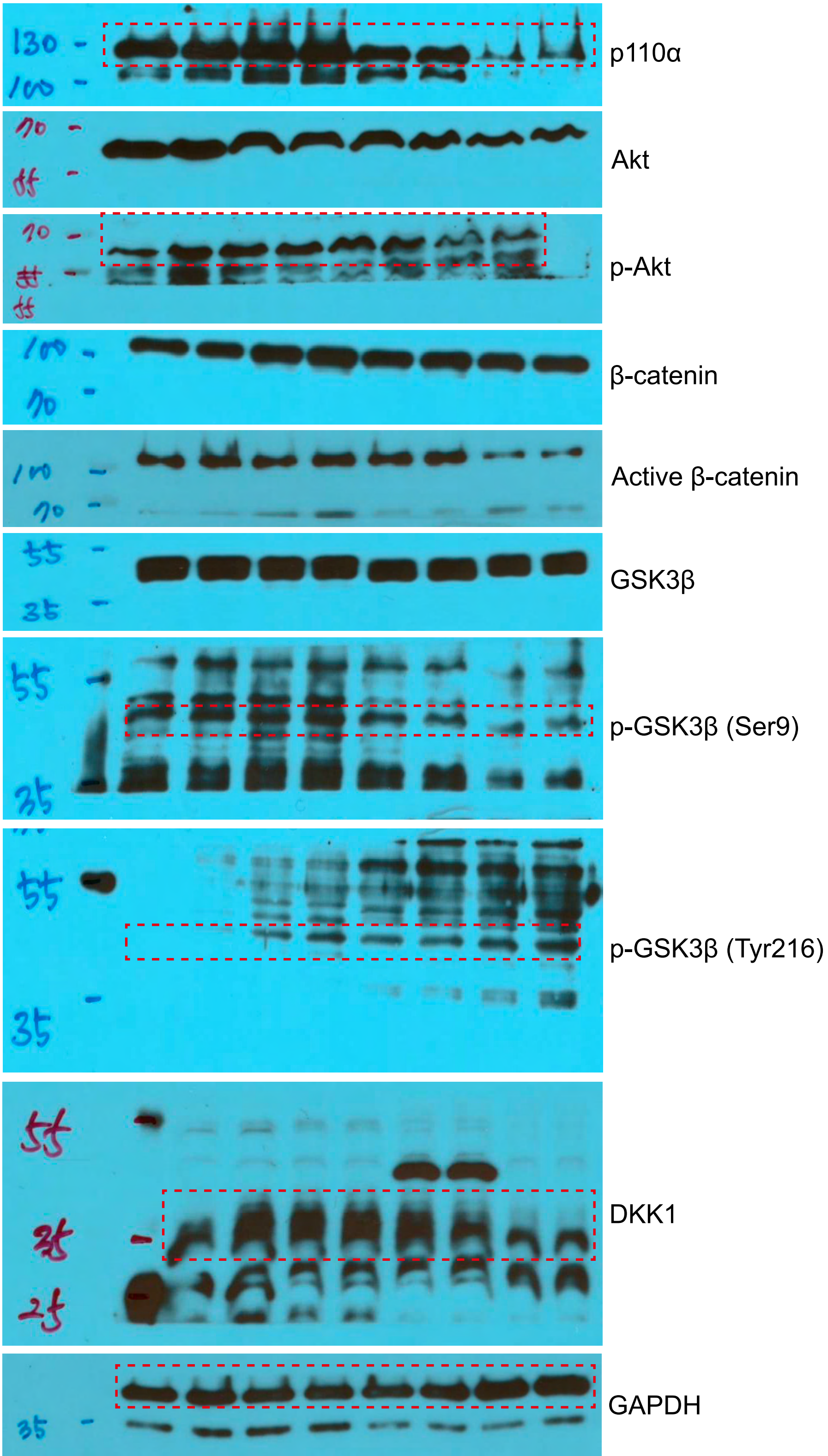

Figure 6.

Figure 6A

Huh7

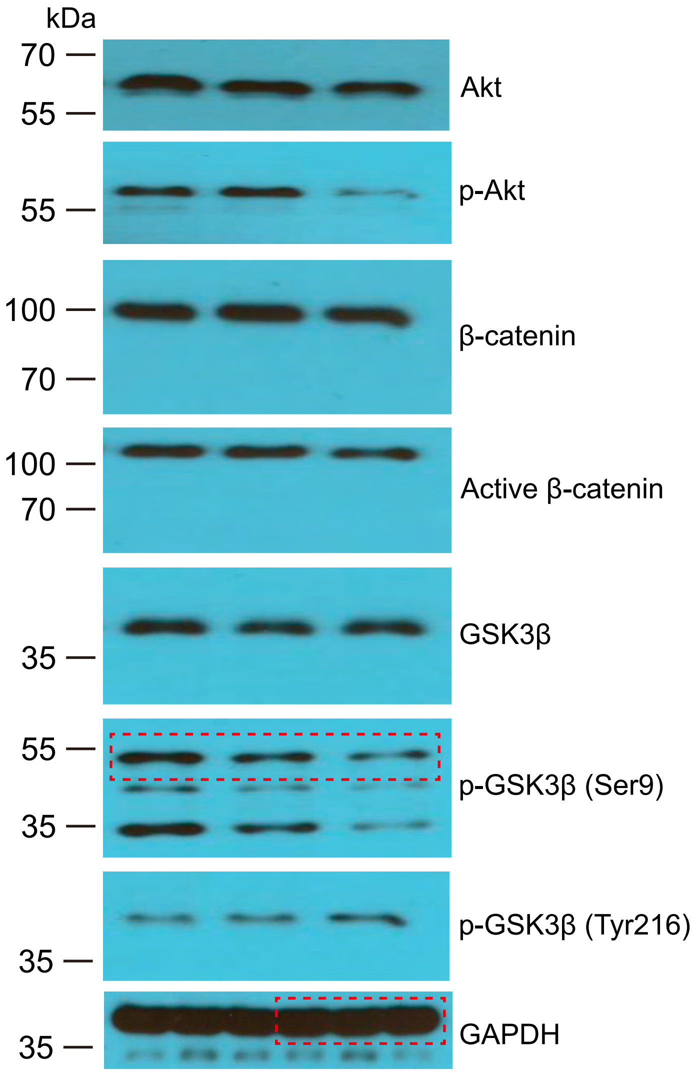

Figure 6.

Figure 6A

Hep3B

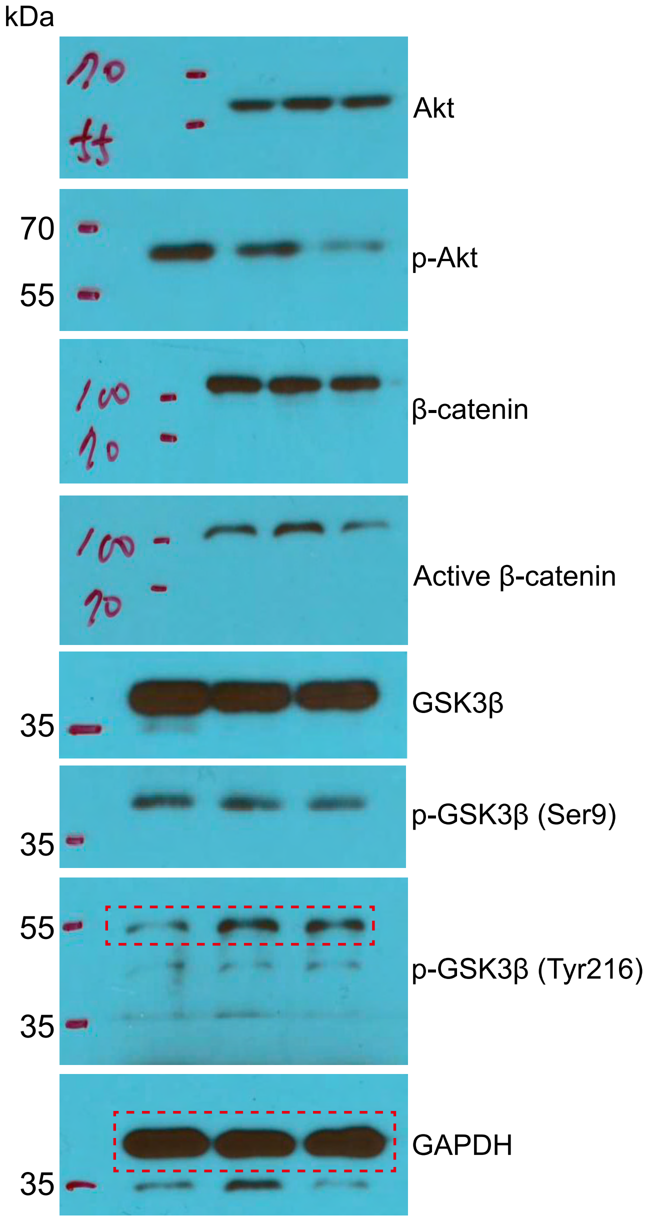

Figure 6.

Figure 6D

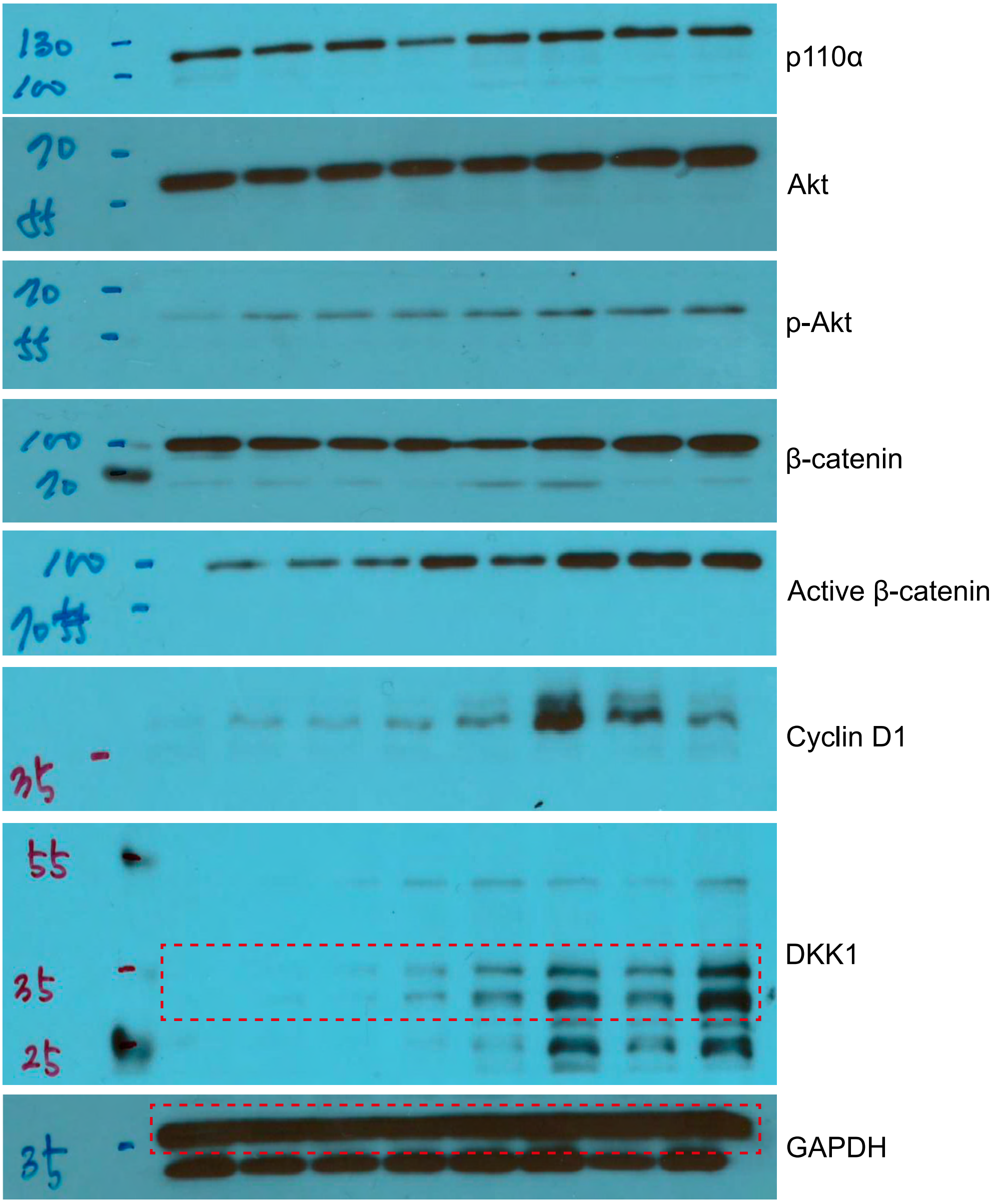

Supplementary Figure 3.

Supple Fig 3C

Huh7

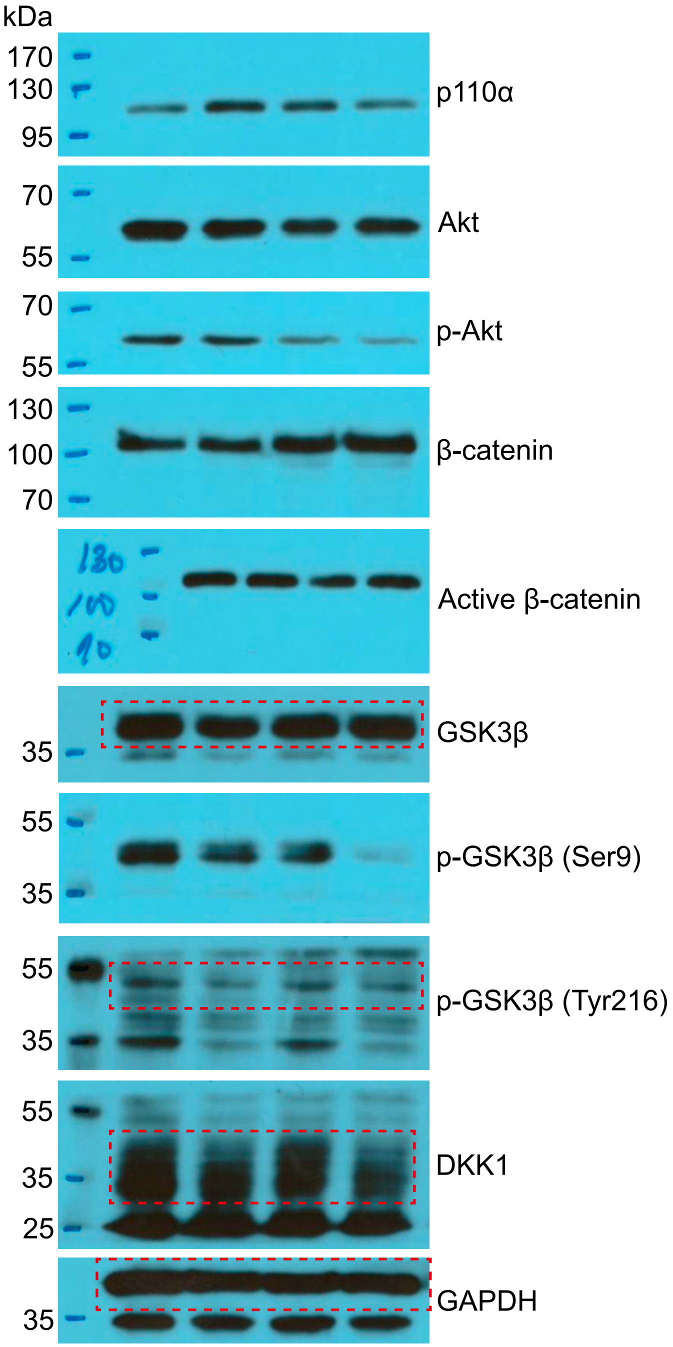

Supplementary Figure 3.

Supple Fig 3C

Hep3B

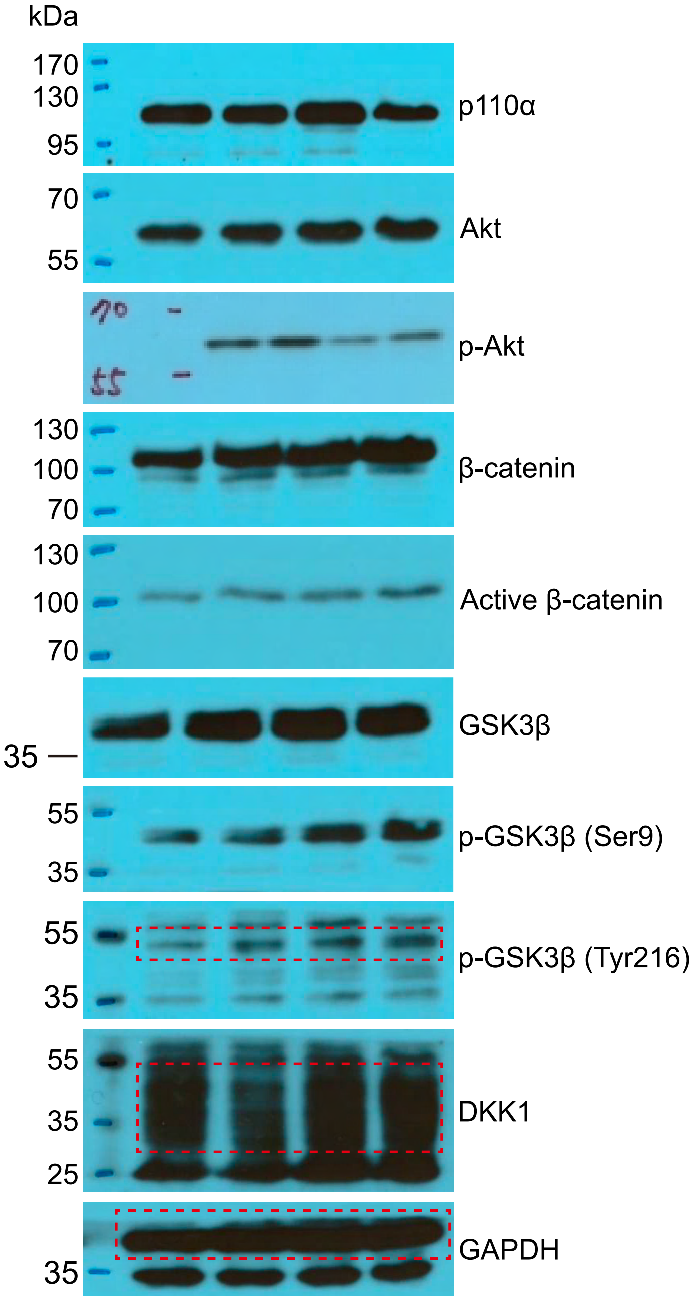

Supplementary Figure 4.

Supple Fig 4D

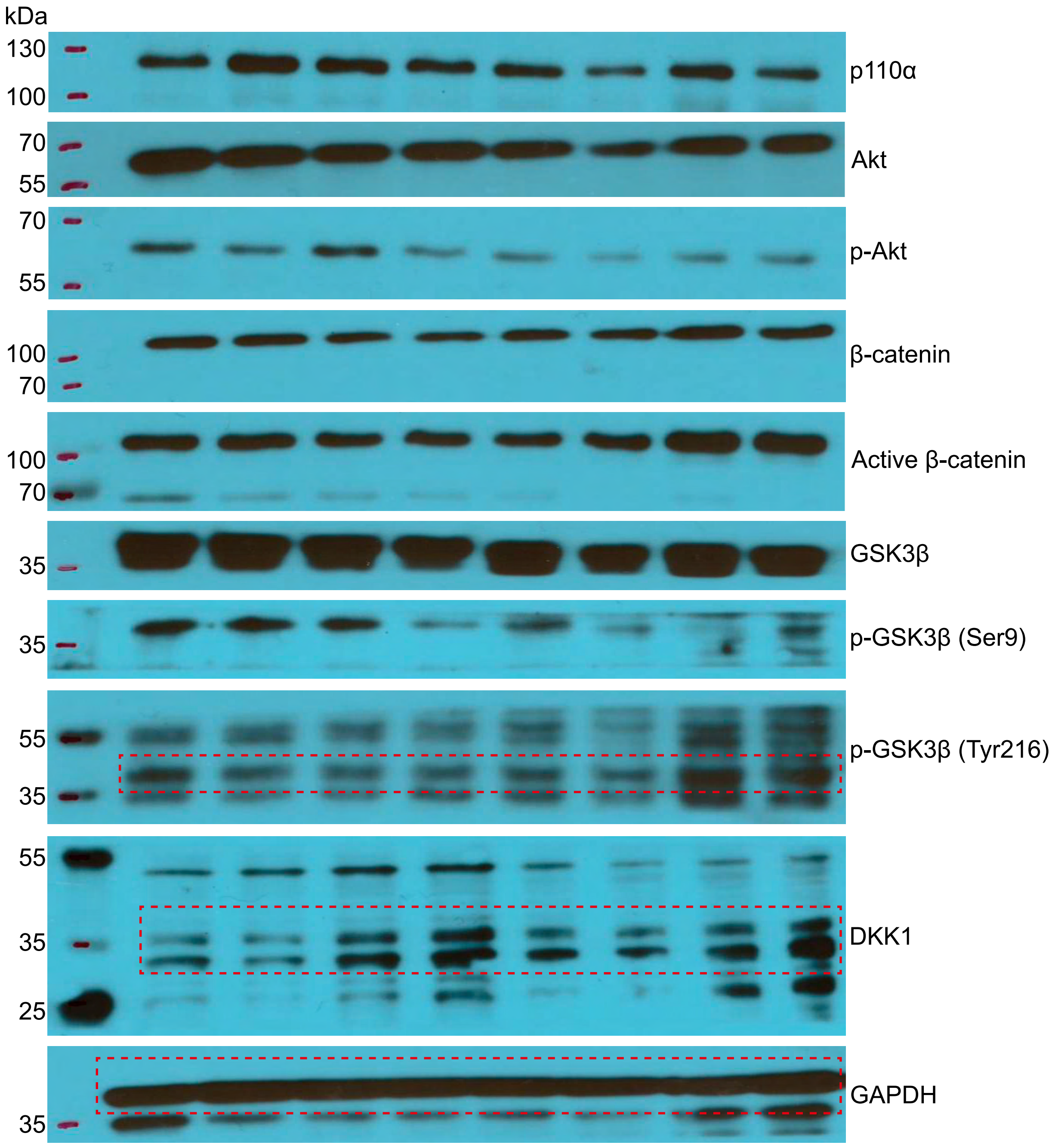

Supplement: Supplementary file 4 — Additional file 3. [file 12964_2023_1355_MOESM3_ESM.pdf]
